# Supplementary material for: Trends in health expectancies: a systematic review of international evidence
Source: BMJ Open. 2021 May 25;11(5):e045567. doi: 10.1136/bmjopen-2020-045567 (PMC8154999; doi:10.1136/bmjopen-2020-045567)
Supplement: Supplementary data [file bmjopen-2020-045567supp001.pdf]

Supplementary Materials

Table 1. Summary of included studies

Study	Country	Data source	Trend years	Trends reported	Disability/health measured from a global (single) item or multiple items?[a]	Stratification	At age	Method used to calculate life expectancy
High income countries combined								
Global Burden of Disease study 2016 [b]	High income countries combined	Global Burden of Disease Study	2005, 2015	LE, HLE	Multiple	Sex	0	Sullivan
Global Burden of Disease study 2017 [b]	High income countries combined	Global Burden of Disease Study	1990, 2016	LE, HLE	Multiple	Sex	0, 65	Sullivan
Global Burden of Disease study 2018 [b]	High income countries combined	Global Burden of Disease Study	1990, 2017	LE, HLE	Multiple	Sex	0	Sullivan
Studies with samples from OECD high-income countries in Europe								
Jagger 2016	England	CFAS I and II	1991, 2011	LE, HLE, DFLE, t % of LE without disability, % of life spent healthy, disability prevalence	HLE: global DFLE: multiple	Sex	65	Sullivan
Kingston 2017	England	CFAS I and II	1991, 2011, and 2015, 2025, 2035 (projection)	LE, DFLE, % of LE without disability, disability prevalence	Multiple	Sex	65	Sullivan

Study	Country	Data source	Trend years	Trends reported	Disability/health measured from a global (single) item or multiple items?[a]	Stratification	At age	Method used to calculate life expectancy
Kingston 2018	England	CFAS II, ELSA, Understanding Society	2015, 2025, 2035 (projection)	LE, DFLE, % of LE without disability, disability prevalence	Multiple	Sex, age	65-74, 75-84, 85	Sullivan
Guzman-Castillo 2017	England, Wales	ELSA	2015, 2025 (projection)	LE, DFLE, disability prevalence	Multiple	None	65	Sullivan
Reus-Pons 2017	England & Wales	Census, national death registries, Permanent Survey on the Living Situation (POLS), Health Survey	2001, 2011	LE, HLE	Global	Sex, migrant/non-migrant	50	Sullivan
ONS 2019	England & Wales	UK Census, Annual Population Survey, Indices of Multiple Deprivation 2015, Welsh Index of Multiple Deprivation 2014	2012/14 – 2015/17	LE, HLE	Global	Sex, deprivation decile[c]	0, 65	Sullivan
ONS 2018	UK, England, Wales, Northern Ireland, Scotland	UK Census, Annual Population Survey	2009/11 – 2015/17	LE, HLE	Global	Sex	0	Sullivan
Bronnum-Hansen 2017a	Denmark	Danish Surveys of Health, Ageing and Retirement in Europe	2006/07, 2010/11,2013/14	LE, DFLE,	Global	Sex, education	65	Sullivan
Deeg 2018	Netherlands	Longitudinal Aging Study Amsterdam	1993, 1996, 1999, 2002, 2006, 2009, 2012, 2016	LE, HLE, % of life spent healthy	Multiple	Sex	65	Sullivan
Gheorghe 2016	Netherlands	Dutch Labour force Survey and National Mortality Registry	2001, 2011	LE, Quality Adjusted Life Expectancy),	Multiple	Sex, education	25, 65	Sullivan

Study	Country	Data source	Trend years	Trends reported	Disability/health measured from a global (single) item or multiple items?[a]	Stratification	At age	Method used to calculate life expectancy
Lagergren 2017	Sweden	National mortality statistics, Nationwide Swedish Surveys of Living Conditions	1980/1985, 1994/95, 2006/2011	LE (graph only), DFLE, disability prevalence	Multiple	Sex	65	Sullivan
Remund 2019	Switzerland	Swiss National Cohort, Swiss Health Interview Survey	1990/94, 1995/99, 2004/04, 2010/14	LE, HLE,	Global	Sex, education	30	Sullivan
Renard 2019	Belgium	Census, National Registry	2001, 2011	LE, DFLE,	Global	Sex, education	25	Sullivan
Reus-Pons 2017	Netherlands	Census, national death registries, Permanent Survey on the Living Situation (POLS) and Health Survey	2001, 2011	LE, HLE,	Global	Sex	50	Sullivan
Storeng 2018	Norway	HUNT Study, Norwegian Education Database	1984/86, 1995/97, 2006/08	LE, HLE, DFLE,	HLE & DFLE: Global	Sex, education	30	Sullivan
Sundberg 2016	Sweden	National mortality statistics, Swedish Panel Study of Living Conditions of the Oldest Old (SWEOLD), Survey of Health, Ageing and Retirement in Europe (SHARE)	1992, 2002, 2004, 2011	LE, DFLE,	Multiple	Sex	77	Sullivan
Yokota 2019	Belgium	Health Interview Survey, National Statistics	2001, 2004, 2008	LE, DFLE,	Multiple	Sex	15	Sullivan
Studies with samples from OECD high-income countries in Asia								
Jo 2019	R. Korea	Korean Statistical Information Service, Korea National Health and Nutrition Examination Survey	2005, 2007, 2008, 2009, 2010, 2011, 2012, 2013	LE, HLE,	Multiple	Sex	0	Sullivan
Lee 2016	R. Korea	Korean Statistical Information Service,	2005, 2008, 2011	LE, HLE,	Multiple	Sex	0-100 at five year	Sullivan

Study	Country	Data source	Trend years	Trends reported	Disability/health measured from a global (single) item or multiple items?[a]	Stratification	At age	Method used to calculate life expectancy
		Korea National Health and Nutrition Examination Survey					intervals; ages 0, 65 and 85 reported here	
Sugawara 2016	Japan	Census, Comprehensive Survey of Living Conditions of the People on Health and Welfare	2000, 2010	LE, DFLE, % life without disability	Multiple	None	0	Sullivan
Tokudome 2016	Japan	Global Burden of Disease Study 2013	1990, 1995, 2000, 2005, 2010, 2013	LE, HLE,	Multiple	Sex	0	Sullivan (based on GBD estimates)
Studies with samples from OECD high-income countries in North America								
Cao 2016	US	Human Mortality Database, US National Health Interview Survey (NHIS)	1982, 2010, 2040 (projection)	LE, DFLE,	Global	Sex	55-85	Multi-state life table
Crimmins 2016	US	Census, US National Vital Statistics, National Health Interview	1970, 1980, 1990, 2000, 2010	LE, DFLE, % life without disability, disability prevalence	Global	Sex	0, 20-64, 65, 85	Sullivan
Freedman 2016a	US	National Long-Term Care Survey, National Health and Aging Trends Study	1982, 2004, 2011	LE (graph only), DFLE, prevalence	Multiple	Sex	65, 85	Sullivan
Freedman 2016b	US	National Long-Term Care Survey, National Health and Aging Trends Study	1982, 2004, 2011	LE (graph only), Active Life Expectancy	Multiple	Sex, Race	65, 85	Sullivan
Steensma 2017	Canada	Statistics Canada, National Population Health Survey, Canadian Community Health Survey	1994, , 1994/95, 1996/97, 1998/99, 2000/01 2003, 2005, 2009/10	LE, HLE	Multiple	Sex	0, 20, 65	Sullivan

[a]Global item measures include self-rated health, the Global Limitation Activity Indicator, or other single item measures about having a limitation; [b]Disability adjusted life years reported as global estimates (i.e. not country specific) and were thus not included here; [c]Healthy life expectancy trends are not reported by deprivation deciles, but as a slope index of inequality between the most and least deprived deciles

Spiers GF, *et al. BMJ Open* 2021; 11:e045567. doi: 10.1136/bmjopen-2020-045567

Table 2. Quality assessment

Study	Comparability of interview methods	Outcome measure	Does the trend consider more than 2 time points?	% Response rate in cross-sectional surveys or loss to follow up in longitudinal studies	Proxy %	Missing data %	Other publications consulted	SUMMARY JUDGMENT
Bronnum-Hansen 2017	FAIR<br>Change in items across waves, but disability question did not change across waves 1-7.	Disability: FAIR<br>(Single item question - Global Activity Limitation Indicator)	GOOD: Yes	FAIR<br><br>Response rates:<br><br>2006/07: 69.0%<br>2011/12: 61.5%<br>2013/14: 61.0%),	UNCLEAR	UNCLEAR	SHARE methods report: http://www.share-project.org/fileadmin/pdf_documentation/Methodology/Methodology_2005.pdf	FAIR
Cao 2016	FAIR<br>Not reported, but in Freedman et al. (2002), the NHIS (which is the survey used in Cao et al.), they note the sampling frame was redesigned in 1995. A change in disability items is reported, but this study used a different set of items to ensure disability is comparable across waves.	Disability: FAIR<br>Although this measure draws upon multiple ADLs, the question is whether the participant has any limitation in any of the ADLs, and so is a single item measure.	GOOD: Yes (third time point is projection)	GOOD<br>Annual response rate approximately 80%	UNCLEAR	UNCLEAR	Freedman et al. (2002)<br><br>https://www.cms.gov/About-CMS/Agency-Information/OMH/resource-center/hcps-and-researchers/data-tools/sgm-clearinghouse/nhis	FAIR
Crimmins 2016	FAIR<br>Not reported, but in Freedman et al. (2002), the NHIS (which is the survey used in Crimmins et al), they note the sampling frame was redesigned in 1995. A change in disability items is	Disability: FAIR<br>Although question draws upon multiple ADLs, the question is whether the participant has any limitation in any of the ADLs,	GOOD: Yes	GOOD<br>Annual response rate approximately 80%	UNCLEAR	UNCLEAR	Freedman et al. (2002)	FAIR

Study	Comparability of interview methods	Outcome measure	Does the trend consider more than 2 time points?	% Response rate in cross-sectional surveys or loss to follow up in longitudinal studies	Proxy %	Missing data %	Other publications consulted	SUMMARY JUDGMENT
	reported, but study uses different set of items to ensure disability is comparable across waves.	and so is a single item measure.						
Deeg 2018	GOOD	Disability: GOOD	GOOD: Yes	FAIR<br><br>Response rates:<br><br>1992/93: 81.7%<br>1995/96: 66.9%<br>1998/99: 54.6%<br>2001/02: 44.4% (cohort 1 only, replenished in 2002/2003)<br>2005/06: 33.0% (cohort 1) and : 90.6% (cohort 2)<br>2008/09: 25.9% (cohort 1) and 83.1% (cohort 2)<br>2011/12: 20.1% (cohort 1) and 75.7% (cohort 2)<br>2015/16: 13.1% (cohort 1) and 67.0% (cohort 2) and 83.4% (cohort 3)	GOOD<br>From publication appendix: "Across the study period, the average use of proxies was 2% for respondents in good physical health, 3% for those in fair physical health, and 8% for those in poor physical health."	GOOD<br>Across waves:<br><br>Men: 3.3%<br>Women: 4.4%	https://www.ncbi.nlm.nih.gov/pmc/articles/PMC5010587/<br><br>https://link.springer.com/article/10.1007/s10654-019-00541-2	FAIR
Freedman 2016a	GOOD	Disability: GOOD	GOOD: Yes	GOOD<br><br>Response rates:<br><br>1982: 87.3%<br>2004: 80.6%<br>2011: (NHATS round 1) 70.9%	POOR<br>1982: 20.2%<br>2011: 7.2%	GOOD<br>1982: 0%<br>2011: 2.1%	https://aspe.hhs.gov/basic-report/introduction-national-long-term-care-survey<br><br>https://www.nhats.org/sites/default/files/2021-01/NHATS_User_Guide_R9_Final_Release_0.pdf<br><br>https://www.ncbi.nlm.nih.gov/pmc/articles/PMC6934030/	GOOD

Study	Comparability of interview methods	Outcome measure	Does the trend consider more than 2 time points?	% Response rate in cross-sectional surveys or loss to follow up in longitudinal studies	Proxy %	Missing data %	Other publications consulted	SUMMARY JUDGMENT
Freedman 2016b	GOOD	Disability: GOOD	GOOD: Yes	GOOD<br><br>Response rates:<br><br>1982: 87.3%<br>2004: 80.6%<br>2011: (NHATS round 1) 70.9%	POOR<br>1982: 20.2%<br>2011: 7.2%	GOOD<br>1982: 0%<br>2011: 2.1%	https://aspe.hhs.gov/basic-report/introduction-national-long-term-care-survey<br><br>https://www.nhats.org/sites/default/files/2021-01/NHATS_User_Guide_R9_Final_Release_0.pdf<br><br>https://www.ncbi.nlm.nih.gov/pmc/articles/PMC6934030/	GOOD
GBD 2016 DALYs and HALE Collaborators	UNCLEAR<br>Not reported.	Disability: GOOD	FAIR: No	UNCLEAR	UNCLEAR	UNCLEAR		UNCLEAR
GBD 2017 DALYs and HALE Collaborators	UNCLEAR<br>Not reported.	Disability: GOOD	FAIR: No	UNCLEAR	UNCLEAR	UNCLEAR		UNCLEAR
GBD 2018 DALYs and HALE Collaborators	UNCLEAR<br>Not reported.	Disability: GOOD	FAIR: No	UNCLEAR	UNCLEAR	UNCLEAR		UNCLEAR
Gheorghe 2016	FAIR<br>Mode of collection differed but health questions remained the same.	Health: GOOD	FAIR: No	UNCLEAR	UNCLEAR	UNCLEAR		UNCLEAR
Guzman-Castillo 2017	NA as forecasting from baseline data	Disability: GOOD	FAIR: No	NA (forecasts)	GOOD<br>Full interview by proxy 4%	UNCLEAR	Rogers et al. (2016) DOI 10.1007/978-981-287-080-3_52-1	FAIR

Study	Comparability of interview methods	Outcome measure	Does the trend consider more than 2 time points?	% Response rate in cross-sectional surveys or loss to follow up in longitudinal studies	Proxy %	Missing data %	Other publications consulted	SUMMARY JUDGMENT
					Partial interview or institute proxy <1%		https://www.ifs.org.uk/uploads/elsa/docs_w7/ELSA%20Wave%207%20report.pdf	
Jagger 2016	GOOD	SRH: FAIR<br>Disability: GOOD	FAIR: No	FAIR<br><br>Response rates:<br><br>CFAS I: 80%<br>CFAS II: 56%	GOOD<br><br>CFAS I: 0.1%<br>CFAS II: 0.1%	GOOD<br>SRH: 2·9% (1991) and 4·2% (2011)<br>Disability: 1·1% (1991) and 4·2% (2011)	https://www.sciencedirect.com/science/article/pii/S0140673613615706<br><br>https://ars.els-cdn.com/content/image/1-s2.0-S0140673613615706-mmc1.pdf	FAIR/GOOD depending on outcome
Jo 2019	GOOD<br>Although there were changes to the survey, only waves 3-5 were used where the health question used was the same.	QALE: GOOD	GOOD: Yes	UNCLEAR<br>Not reported	GOOD<br>3-4%	GOOD<br>3-4%	https://www.ncbi.nlm.nih.gov/pmc/articles/PMC3937975/	UNCLEAR
Kingston 2017	GOOD	Disability: GOOD	GOOD: Yes (fourth and fifth time points are projections)	FAIR<br><br>Response rates:<br><br>CFAS I: 80%<br>CFAS II: 56%	GOOD<br><br>CFAS I: https://ars.els-cdn.com/content/image/1-s2.0-S0140673613615706-mmc1.pdf<br>CFAS II: 0.1%	GOOD<br>Not reported but assumed to be same as Jagger et al. as uses same data.	https://www.sciencedirect.com/science/article/pii/S0140673613615706<br><br>https://ars.els-cdn.com/content/image/1-s2.0-S0140673613615706-mmc1.pdf	FAIR
Kingston 2018	NA as forecasting from baseline data	Disability: GOOD	GOOD: Yes (third time point is projection)	FAIR<br><br>Response rates:<br><br>CFAS II: 56%<br>ELSA Wave 5: 80%<br>Understanding society wave 1: 57% (General population)	UNCLEAR/GOOD depending on dataset<br><br>CFAS II: 0.1% (GOOD)<br><br>No information on % proxy	UNCLEAR<br>Not reported, but missing values imputed.	https://www.understandingsociety.ac.uk/sites/default/files/downloads/documentation/mainstage/technical-reports/Wave_1_Technical_Report.pdf	FAIR

Study	Comparability of interview methods	Outcome measure	Does the trend consider more than 2 time points?	% Response rate in cross-sectional surveys or loss to follow up in longitudinal studies	Proxy %	Missing data %	Other publications consulted	SUMMARY JUDGMENT
				and 40% (Ethnic Minority Boost)	interviews for ELSA (UNCLEAR).			
Lagergren 2017	POOR<br>"In the years 1996–2000, a filter was introduced into the questionnaire to the effect that only people who were dependent for all instrumental ADL (IADL) were asked the questions about ADL." (p.552)<br><br>"From 1980 to 2001 there was an upper age limit of 84 years, with the exception of 1988/89." (p.56)	Disability: GOOD	GOOD: Yes	UNCLEAR<br>Response rate reported to be 70-75% with slight decrease up to 2006, but 'lower' after 2006 (actual rates not reported).	FAIR<br>From methods paper on dataset: "The proportion of proxy interviews varies from 2–3% among those aged 65–79 years to 14–16% among individuals aged 80 years and older." (p.56)	UNCLEAR	https://journals.sagepub.com/doi/full/10.1177/1403494815605195	POOR
Lee 2016	GOOD<br>Same criteria used across the waves used to identify diseases.	GOOD<br>Health adjusted life expectancy was calculated using disability weights attached to self-reported morbidity for 45 diseases. This was converted into a score of disability measured on a scale from 0 (full health) to 1 (being dead). This would suggest it	GOOD: Yes	UNCLEAR<br>Not reported, but 2011 KNHANES wave known to be 76.1% for health interview and examination (Jo et al., 2019)	GOOD<br>3-4%<br>Not reported, but assumed to be the same as for Lee et al., 2016, which uses the same data.	GOOD<br>3-4%<br>Not reported, but assumed to be the same as for Lee et al., 2016, which uses the same data.		UNCLEAR

Study	Comparability of interview methods	Outcome measure	Does the trend consider more than 2 time points?	% Response rate in cross-sectional surveys or loss to follow up in longitudinal studies	Proxy %	Missing data %	Other publications consulted	SUMMARY JUDGMENT
		is a multiple item measure.						
ONS 2018	GOOD<br>No change in health question/measure, but items used to measure disability changed from the 2013 APS survey (p.4). However, the ONS analysis does not report trends in DFLE (HLE and LE only).	Health: FAIR	FAIR: No	UNCLEAR	UNCLEAR	UNCLEAR<br>Methods report states that missing data are deleted from the survey, but there is no detail on what proportion of data is missing.	https://www.ons.gov.uk/peoplepopulationandcommunity/healthandsocialcare/healthandlifeexpectancies/methodologies/healthstatelifeexpectanciesukqmi#_blank	UNCLEAR
ONS 2019	GOOD<br>No change in health question/measure, but items used to measure disability changed from the 2013 APS survey (p.4). However, the ONS analysis does not report trends in DFLE (HLE and LE only).	Health: FAIR	FAIR: No	UNCLEAR	UNCLEAR	UNCLEAR<br>Methods report states that missing data are deleted from the survey, but there is no detail on what proportion of data is missing.	https://www.ons.gov.uk/peoplepopulationandcommunity/healthandsocialcare/healthandlifeexpectancies/methodologies/healthstatelifeexpectanciesukqmi#_blank	UNCLEAR
Remund 2019	POOR<br>The phrasing of the SRH question and response items were not identical in each survey wave. However, additional analysis supported the use of	SRH: FAIR	GOOD: Yes	GOOD<br><br>Response rates:<br><br>1992: 70.8%<br>1997: 68.8%<br>2002: 63.9%<br>2007: 66.3%	GOOD<br>(% cases, % weights)<br>1992: 0.67%, 0.86%<br>1997: 0.74%, 0.79%	GOOD<br>(% cases, % weights)<br>1992: 0.0%, 0.0%<br>1997: 0.03%, 0.04%	Supplementary materials	POOR

Study	Comparability of interview methods	Outcome measure	Does the trend consider more than 2 time points?	% Response rate in cross-sectional surveys or loss to follow up in longitudinal studies	Proxy %	Missing data %	Other publications consulted	SUMMARY JUDGMENT
	dichotomisation to maximise the comparability of the questions between waves.			2012: 53.1	2002: 1.05%, 0.86%<br>2007: 4.41%, 6.23%<br>2012: 3.32%, 4.26%	2002: 0.03%, 0.02%<br>2007: 0.05%, 0.05%<br>2012: 0.12%, 0.12%		
Renard 2019	GOOD<br>Methods paper states no major methodological changes have been implemented between waves, except oversampling from 1997.	Disability: FAIR (Single item question - Global Activity Limitation Indicator)	FAIR: No	FAIR<br><br>Response rates:<br><br>2001: 49%<br>2004: 49.9%<br>2008: 40.2%<br>2013: Not reported.	UNCLEAR	UNCLEAR	https://archpublichealth.biomedcentral.com/articles/10.1186/0778-7367-71-24	FAIR
Reus-Pons 2017	GOOD<br>For England & Wales data, paper indicates SRH question changed but an adjustment was made for comparability. For Netherlands data, footnote to table states no major changes were made to SRH question and choices.	SRH: FAIR	FAIR: No	UNCLEAR<br><br>Response rates:<br><br>POLS: UNCLEAR<br>Census 2011: 94% (person response rate)<br>Census 2001: 98%	UNCLEAR	UNCLEAR<br>For Belgium sample: data on self-rated health were missing for 'around' 5% of the non-migrant population and 'around' 10% of the migrant population. Exact % not reported, thus could be >10%.<br>Not reported for England	https://www.mzes.uni-mannheim.de/projekte/mikrodaten/wp_pdf/wp_30_NL-ro.pdf<br><br>https://www.ons.gov.uk/census/2001censusandearlier/dataandproducts/qualityofthecensusdata/responserates<br><br>https://www.ons.gov.uk/peoplepopulationandcommunity/populationandmigration/populationestimates/bulletins/2011censuspopulationandhouseholdestimatesforenglandandwales/2012-07-16	UNCLEAR

Study	Comparability of interview methods	Outcome measure	Does the trend consider more than 2 time points?	% Response rate in cross-sectional surveys or loss to follow up in longitudinal studies	Proxy %	Missing data %	Other publications consulted	SUMMARY JUDGMENT
						and Wales sample.		
Steensma 2017	GOOD<br>Different surveys used between 1994-1999 and 2000-2010, but same question for health used in both.	Health: GOOD (multiple item health utilities index)	GOOD: Yes	GOOD<br><br>Response rates:<br><br>NPHS:<br>1994/95: 88.7%,<br>1996/97: 82.6%<br>1998/99: 89.7%<br><br>CCHS:<br>2000/01: 84.7%<br>2009/10: 72.3%	POOR (NPHS), GOOD (CCHS)<br><br>NPHS: 27.5% (for health data)<br><br>CCHS: 6.3%	NPHS: GOOD 1994/1995: between 1% and 9% depending on variable.<br>CCHS: UNCLEAR	https://www23.statcan.gc.ca/imdb/p2SV.pl?Function=getSurvey&Id=3359<br>https://pubmed.ncbi.nlm.nih.gov/10607414/<br>https://pubmed.ncbi.nlm.nih.gov/11565112/	GOOD
Storeng 2018	POOR<br>Self-rated health was measured the same at all three HUNT studies. The question for longstanding limiting illness differed between HUNT1 and HUNT 2 and 3 (that is, HUNT 2 and 3 asked about limiting illness lasting for at least one year, whereas HUNT 1 did not).	Health: FAIR<br>Disability: FAIR	GOOD: Yes	FAIR<br><br>Response rates:<br><br>HUNT 1: 90%<br>HUNT 3: 54%	UNCLEAR	GOOD<br>Missing health data < 5%	https://academic.oup.com/ije/article/42/4/968/655743#11331793	POOR
Sugawara 2016	GOOD	FAIR<br>Disability was measured using the following two survey questions: "Are you currently institutionalized in hospitals,	FAIR: No	GOOD<br><br>Response rates:<br><br>2001: 87.4%<br>2010: 79.4%	UNCLEAR	UNCLEAR		FAIR

Study	Comparability of interview methods	Outcome measure	Does the trend consider more than 2 time points?	% Response rate in cross-sectional surveys or loss to follow up in longitudinal studies	Proxy %	Missing data %	Other publications consulted	SUMMARY JUDGMENT
		clinics, or long-term care facilities?" and "Do you have any limitations in carrying out normal activities due to health problems?" Those who answered "yes" to **either** question are considered to have disability.						
Sundberg 2016	FAIR<br>Mode of data collection changed but health question remain the same.	Disability: GOOD	GOOD: Yes	GOOD/FAIR depending on dataset<br><br>Response rates:<br><br>SWEOLD<br>1992: 95.4%<br>2002: 84.4%<br>2004: 46.9%<br>2011: 86.2%<br><br>SHARE (retention plus recovery):<br>2004: 70.6%<br>2011:108.4%	FAIR/POOR depending on dataset<br><br>SHARE<br>2004: 14.4%<br>2011: 9 %<br><br>SWEOLD<br>1992: 15%<br>2002: 19.8%<br>2004: 21.5%<br>2011: 20.9%	GOOD/UNCLEAR depending on dataset<br><br>SHARE<br>Not reported.<br><br>SWEOLD (severe disability, mild disability, mobility)<br>1992: 1.7%, 0.4%, 0%<br>2002: 1.0%, 1.1%, 1.3%<br>2004: 2.0%, 0.9%, 0.9%<br>2011: 2.4%, 1.3%, 1.4%	https://link.springer.com/article/10.1007%2Fs10433-013-0275-7 and https://academic.oup.com/ije/article/43/3/731/2949546#57616062<br><br>https://academic.oup.com/ije/article/43/3/731/2949546<br><br>https://academic.oup.com/ije/article/42/4/992/657275<br><br>Borsch-Supan, A & Scherpenzeel , A. The Survey of Health, Ageing and Retirement in Europe. Munich Center for the Economics of Aging,	FAIR

Study	Comparability of interview methods	Outcome measure	Does the trend consider more than 2 time points?	% Response rate in cross-sectional surveys or loss to follow up in longitudinal studies	Proxy %	Missing data %	Other publications consulted	SUMMARY JUDGMENT
Tokudome 2016	GOOD	Disability: GOOD	GOOD: Yes	UNCLEAR<br>(Based on HALE values from GBD 2013)	UNCLEAR	UNCLEAR<br>But reported to be rare by author.	http://www.healthdata.org/sites/default/files/files/Projects/GBD/GBD_Protocol.pdf	UNCLEAR
Yokota 2019	GOOD<br>Methods paper states no major methodological changes have been implemented between waves, except oversampling from 1997.	Disability: GOOD	GOOD: Yes	FAIR<br><br>2001: 49%<br>2004: 49.9%<br>2008: 40.2%	FAIR<br>Time 1: 4.3%,<br>time 2: 10.2%,<br>time 3: 13%	UNCLEAR	https://archpublichealth.biomedcentral.com/articles/10.1186/0778-7367-71-24	FAIR

For tables 3a to 7b, the change in expectancy from the first to the last time point represents the final estimated expectancy minus the first estimated expectancy.

Table 3a. Summary of life expectancy trends data for studies from the US and Canada (years)[a]

Study	At age	Time points	T1		T2		T3		T4		T5		T6		T7		Change between first and last time point
			M	F	M	F	M	F	M	F	M	F	M	F	M	F	
Cao 2016	55-85	T1: 1982<br>T2: 1990<br>T3: 2000<br>T4: 2010<br>T5: 2020<br>T6: 2030<br>T7: 2040	19.96	23.40	20.70	23.64	21.75	23.92	22.82	24.69	23.83 (23.5 - 24.05)	25.11 (24.9 - 25.24)	24.63 (24.2 - 24.98)	25.49 (25.2 - 25.69)	25.24 (24.7 - 25.67)	25.74 (25.4 - 26.00)	Men: 5.28[b]<br>Women: 2.34[b]
Crimmins 2016	0	T1: 1970<br>T2: 1980<br>T3: 1990<br>T4: 2000<br>T5: 2010	67.0	74.6	70.1	77.6	71.8	78.8	74.1	79.5	76.2	81.0					Men: 9.2<br>Women: 6.4
	20-64		40.8	42.8	41.6	43.3	41.8	43.5	42.4	43.6	42.6	43.7					Men: 1.8<br>Women: 0.9
	65		13.0	16.8	14.2	18.4	15.1	19.0	16.1	19.1	17.7	20.3					Men: 4.7<br>Women: 3.5
	85		4.7	5.6	5.1	6.4	5.3	6.7	5.5	6.6	5.8	6.9					Men: 1.1<br>Women: 1.3
Steensma 2017	0	T1: 1994/95<br>T2: 96/97<br>T3: 98/99<br>T4: 00/01<br>T5: 03<br>T6: 05<br>T7: 09/10	75.2	81.3	75.7	81.5	76.2	81.8	77.2	82.5	77.7	82.8	78.3	83.2	79.6	84.1	Men: 4.4[b]<br>Women: 2.8[b]
	20		55.7	61.7	56.2	61.8	56.7	62.1	57.7	62.8	58.1	63.0	58.7	63.4	59.9	64.4	Men: 4.2[b]<br>Women: 2.7[b]
	65		15.8	19.9	15.9	19.9	16.2	20.2	17.0	20.7	17.4	20.9	19.9	21.3	18.9	22.1	Men: 3.1[b]<br>Women: 2.2[b]

[a]95% confidence intervals in brackets where reported; [b]Change estimate not reported, and thus calculated by us (T^2-T^1)

Table 3b. Summary of healthy life expectancy trends data for studies from the US and Canada (years)[a]

Study	At age	Time points	T1		T2		T3		T4		T5		T6		T7		Average annual change (%)
			M	F	M	F	M	F	M	F	M	F	M	F	M	F	
Steensma 2017	0	T1: 1994/95<br>T2: 96/97<br>T3: 98/99<br>T4: 00/01<br>T5: 03<br>T6: 05<br>T7: 09/10	66.4 (66.0 - 66.8)	70.0 (69.5 - 70.4)	69.0 (68.7 - 69.2)	72.9 (72.5 - 73.2)	68.9 (68.6 - 69.3)	72.1 (71.7 - 72.5)	68.5 (68.3 - 68.7)	71.5 (71.3 - 71.7)	69.5 (69.1 - 69.8)	71.7 (71.3 - 72.1)	69.3 (69.0 - 69.7)	72.3 (71.9 - 72.6)	70.7 (70.5 - 70.9)	73.3 (73.1 - 73.5)	Men: 0.3<br>Women: 0.2
	20		48.0 (47.6 - 48.3)	51.4 (51.0 - 51.9)	50.1 (49.8 - 50.3)	54.0 (53.7 - 54.3)	50.1 (49.7 - 50.4)	53.1 (52.7 - 53.5)	49.8 (49.6 - 50.0)	52.7 (52.5 - 52.9)	50.9 (50.5 - 51.2)	53.1 (52.7 - 53.5)	50.8 (50.4 - 51.2)	55.3 (53.1 - 53.9)	50.2 (51.8 - 52.2)	54.5 (54.3 - 54.8)	Men: 0.4<br>Women: 0.2
	65		12.3 (12.0 - 12.6)	14.8 (14.4 - 15.1)	12.8 (12.6 - 13.1)	15.6 (15.3 - 15.9)	12.8 (12.5 - 13.1)	15.3 (15.0 - 15.7)	13.1 (13.0 - 13.3)	15.4 (15.2 - 15.6)	14.0 (13.7 - 14.3)	15.7 (15.4 - 16.1)	14.2 (13.9 - 14.5)	16.0 (15.6 - 16.3)	14.9 (14.7 - 15.1)	16.8 (16.7 - 17.0)	Men: 1.2<br>Women: 0.7

[a]95% confidence intervals in brackets where reported;

Table 3c. Summary of disability-free life expectancy trends data for studies from the US and Canada[a]

Study	Trend	At age	Time points	T1		T2		T3		T4		T5		Change between first and last time point
				Male	Female	Male	Female	Male	Female	Male	Female	Male	Female	
Crimmins (2016)	DFLE (years)	0	T1: 1970<br>T2: 1980<br>T3: 1990<br>T4: 2000<br>T5: 2010	56.5 (56.4 - 56.6)	62.7 (62.6 - 62.8)	56.5 (56.4 - 56.6)	62.8 (62.6 - 63.0)	58.8 (58.6 - 58.9)	63.9 (63.8 - 64.0)	60.0 (59.9 - 60.2)	64.6 (64.4 - 64.7)	61.0 (60.9 - 61.2)	65.4 (65.3 - 65.6)	Men: 4.5<br>Women: 2.7
		20-64		34.9 (34.8 - 35.0)	37.4 (37.3 - 37.5)	34.7 (34.6 - 34.8)	36.7 (36.6 - 36.8)	35.3 (35.2 - 35.4)	37.0 (36.9 - 37.1)	35.6 (35.5 - 35.7)	36.9 (36.8 - 37.0)	35.8 (35.7 - 35.9)	36.8 (36.7 - 37.0)	Men: 0.9<br>Women: -0.6
		65		6.6 (6.6 - 6.8)	9.1 (9.0 - 9.2)	6.8 (6.7 - 6.9)	9.3 (9.2 - 9.4)	7.4 (7.3 - 7.5)	9.9 (9.8 - 10.0)	8.2 (8.0 - 8.3)	10.5 (10.3 - 10.6)	9.3 (9.1 - 9.4)	11.5 (11.4 - 11.6)	Men: 2.7<br>Women: 2.4
		85		1.4 (1.3 - 1.6)	1.4 (1.3 - 1.6)	1.5 (1.3 - 1.7)	1.7 (1.5 - 1.8)	1.6 (1.5 - 1.8)	1.7 (1.6 - 1.8)	1.8 (1.6 - 1.9)	1.9 (1.8 - 2.0)	1.9 (1.8 - 2.1)	2.2 (2.1 - 2.4)	Men: 0.5<br>Women: 0.8
	% of life spent disability-free	0		84.2	84.0	81.6	80.9	81.8	81.1	81.0	81.3	80.1	80.7	Men: -4.1<br>Women: -3.3
		20-64		85.5	87.4	83.4	84.8	84.4	85.0	84.0	84.6	84.0	84.2	Men: -1.5<br>Women: -3.2
		65		51.2	54.2	47.8	50.5	49.1	52	50.9	55	52.5	56.7	Men: 1.3<br>Women: 2.5
		85		30.4	25.2	29	26	30.8	25.5	32.7	28.8	32.8	31.9	Men: 2.4<br>Women: 6.7
	LE for those in an institution	0		0.6 (0.6 - 0.6)	1.1 (1.0 - 1.2)	0.6 (0.6 - 0.7)	1.4 (1.3 - 1.5)	0.7 (0.6 - 0.7)	1.5 (1.4 - 1.5)	0.5 (0.5 - 0.6)	1.1 (1.0 - 1.2)	0.4 (0.4 - 0.5)	0.8 (0.8 - 0.9)	Men: -0.2<br>Women: -0.3
		20-64		0.3 (0.2 - 0.3)	0.2 (0.2 - 0.2)	0.2 (0.2 - 0.2)	0.2 (0.1 - 0.2)	0.2 (0.2 - 0.2)	0.1 (0.1 - 0.1)	0.1 (0.1 - 0.1)	0.1 (0.1 - 0.1)	0.1 (0.1 - 0.1)	0.1 (0.0 - 0.1)	Men: -0.2<br>Women: -0.1

Study	Trend	At age	Time points	T1		T2		T3		T4		T5		Change between first and last time point
				Male	Female	Male	Female	Male	Female	Male	Female	Male	Female	
		65		0.5 (0.5 - 0.5)	1.1 (1.0 - 1.2)	0.6 (0.5 - 0.6)	1.5 (1.4 - 1.6)	0.7 (0.6 - 0.7)	1.6 (1.5 - 1.6)	0.5 (0.5 - 0.6)	1.2 (1.1 - 1.3)	0.4 (0.4 - 0.5)	0.9 (0.8 - 0.9)	Men: -0.1 Women: -0.2
		85		0.6 (0.5 - 0.7)	1.2 (1.1 - 1.3)	0.8 (0.7 - 1.0)	1.7 (1.5 - 1.8)	0.9 (0.8 - 1.0)	1.9 (1.8 - 2.0)	0.7 (0.6 - 0.8)	1.4 (1.3, 1.5 -	0.4 (0.4 - 0.5)	0.9 (0.8 - 1.0)	Men: -0.2 Women: -0.3
Cao 2016	DFLE (years)	55-85	T1: 1982 T2: 1990 T3: 2020 T4: 2030 T5: 2040	12.98	14.9	16.92	17.53	17.86 (17.70 - 18.03)	18.00 (17.89 - 18.13)	18.99 (18.68 - 19.36	18.67 (18.41 - 18.97)	20.10 (19.60 - 20.64)	19.30 (18.88 - 19.77)	Men: 7.12[b] Women: 4.4[b]
Freedman (2016)a	DFLE (years)	65	T1: 1982 T2: 2004 T3: 2011	**data only reported in graphs, unable to extract**										Men: 4.5 Women: 1.4
		85		2.5	2.5	4.4	2.6							Men: 1.9 Women: 0.1[b]
Freedman (2016)b	Active Life Expectancy (years)	65	T1: 1982 T2: 2011	White: 12.2 Black: 9.8		White: 15.9 Black: 12.0								White: 2.8 Black: 2.2
		85		White: 2.5 Black: 2.25		White: 3.0 Black: 2.0								White: 0.5 Black: -0.25

[a]95% confidence intervals in brackets where reported; [b]Change estimate not reported, and thus calculated by us (T^2-T^1)

Table 3d. Summary of disability prevalence trends data for studies from the US and Canada[a]

Study	Trend	Time points	Change between first and last time point
Crimmins (2016)	Proportion (%) of individuals with disability in the community (age 65-84)	T1: 1970<br>T2: 1980<br>T3: 1990<br>T4: 2000<br>T5: 2010	Men: -2.5<br>Women: -2.2
	Proportion (%) of individuals with disability in the community (age 85+)		Men: -0.5<br>Women: -5.8
Freedman (2016)	Proportion of those with a severe limitation (limited in 3+ personal care activities or living in a nursing home)	T1: 1982<br>T2: 2004<br>T3: 2011	Men: -3.4[b]<br>Women: -3.0[b]

[a]Estimates for each time point were not extracted here due to volume. Reader is referred to publications and associated supplementary materials; [b]Change estimate not reported, and thus calculated by us (T^2-T^1)

Table 4a. Summary of life expectancy trends data for UK studies and studies with UK samples (years)[a]

Study author and date	At age	Time points	T1		T2		T3		Change between first and last time point
			Male	Female	Male	Female	Male	Female	
Reus-Pons 2017	50	T1: 2001<br>T2: 2011	28.54<br><br>Immigrant: 27.52<br>Non-immigrant: 28.66	32.32<br><br>Immigrant: 31.82<br>Non-immigrant: 32.39	31.29<br><br>Immigrant: 31.01<br>Non-immigrant: 31.32	34.5<br><br>Immigrant: 34.84<br>Non-immigrant: 34.47			Men: 2.8[b]<br>Women: 2.2[b]
Jagger 2016	65	T1: 1991<br>T2: 2011	13·0	16·7	17·5	20·3			Men: 4·5<br>Women: 3·6
Kingston 2018	65	T1: 2015<br>T2: 2025<br>T3: 2035	18·7 (18·3 to 19·0)	21·1 (20·8 to 21·1)	20·7 (20·5 to 21·0)	22·7 (22·5 to 23·3)	22·2 (21·7 to 22·4)	24·1 (23·9 to 24·4)	Men: 3·5 (3·1 to 4·1)<br>Women: 3·0 (3·0 to 3·6)
Kingston 2017	65	T1: 1991<br>T2: 2011	12.9	16.5	17.6	20.6			Men: 4.7<br>Women: 4.1
ONS 2018	0	T1: 2009/12<br>T2: 2015/17	**Not reported**						UK<br>Men: 0.8<br>Women: 0.4<br>England<br>Men: 0.8<br>Women: 0.4<br>Northern Ireland<br>Men: 1.0<br>Women:0.5<br>Scotland<br>Men: 0.8<br>Women: 0.5

Spiers GF, *et al. BMJ Open* 2021; 11:e045567. doi: 10.1136/bmjopen-2020-045567

Study author and date	At age	Time points	T1		T2		T3		Change between first and last time point
			Male	Female	Male	Female	Male	Female	
									Wales<br>Men: 0.5<br>Women: 0.2

[a]95% confidence intervals in brackets where reported; [b]Change estimate not reported in publication, and calculated by us (T^2-T^1);

Table 4b. Summary of healthy life expectancy trends data for UK studies and studies with UK samples[a]

Study	Trend	At age	Time points	T1		T2		Change between first and last time point
				Male	Female	Male	Female	
Reus-Pons 2017	HLE (years)	50	T1: 2001 T2: 2011	18.47 (18.46-18.48)	19.82 (19.81-19.83)	18.71 (18.70-18.72)	19.67 (19.66-, 19.68)	Men: 0.25 Women: -0.15
Jagger 2016	HLE (years)	65	T1: 1991 T2: 2011	8·8 (8·6-9·1)	11·2 (11·0 - 11·5)	12·6 (12·4-12·9)	14·3 (14·0 - 14·6)	Men: 3·8 (3·5 to 4·1) Women: 3·1 (2·7 to 3·4)
	Proportion (%) of life spent healthy			68·2% (66·5 - 69·9)	67·3% (65·9 - 68·7)	72·4% (70·9 - 73·9)	70·3% (68·8 - 71·7)	Men: 4·2% (2·0 to 6·5) Women: 3·0% (1·0 to 4·9)
ONS 2018	HLE (years) - UK	0	T1: 2009/11 T2: 2015/17	62.7	63.8	63.1	63.6	Men: 0.4 [b] Women: -0.2 [b]

[a]95% confidence intervals in brackets where reported; [b]Change estimate not reported in publication, and calculated by us (T^2-T^1);

Table 4c. Summary of disability-free life expectancy trends data for UK studies and studies with UK samples[a]

Study	Trend	At age	Time points	T1		T2		T3		Change between first and last time point
				Male	Female	Male	Female	Male	Female	
Jagger 2016	DFLE (years)	65	T1: 1991 T2: 2011	10.3 (10.2-10.5)	11.0 (10.8-11.2)	12.9 (12.7-13.2)	11.5 (11.3-11.8)			Men: 2.6 (2.3, 2.9) Women: 0.5 (0.2, 0.9)
	% of life disability-free			79.7 (78.3-81.0)	66.1 (64.9-67.4)	74.4 (73.0-75.8)	56.8 (55.5-58.2)			Men: -5.3 (-7.2, -3.4) Women: -9.3 (-11.1, -7.5)
Kingston 2017	DFLE (years)	65	T1: 1991 T2: 2011	9.5 (9.3-9.7)	9.5 (9.2-9.8)	11.2 (10.8-11.5)	9.7 (9.3-10.2)			Men: 1.7 (1.2, 2.1) Women: 0.2 (-0.4, 0.7)
	% of life disability-free			73.6 (71.8-75.4)	58.0 (56.2-59.9)	63.5 (61.4-65.6)	47.3 (45.0-49.5)			Men: -10.1 (-12.9, -7.3) Women: -10.7 (-13.6, -7.8)
Kingston 2018	DFLE (years)	65	T1: 2015 T2: 2025 T3: 2035	11.1 (10.9-11.3)	10.7 (10.5-10.7)	14.5 (14.4-14.6)	11.4 (11.3-11.5)	15.2 (15.1-15.2)	11.6 (11.6-11.8)	Men: 4.2 (3.9, 4.2) Women: 0.9 (0.9, 1.2)
	% of life disability-free			59.3 (59.3-60.2)	50.6 (50.1-50.7)	70.2 (69.7-70.4)	49.9 (49.4-50.3)	68.7 (67.9-69.5)	48.0 (48.0-48.6)	Men: 9.4 (8.0, 9.7) Women: -2.6%
Guzman-Castillo 2017	DFLE (years)	65	T1: 2015 T2: 2025	14.9 (14.7-15.1)	15.8 (15.7-15.9)	16.5 (15.4-17.6)	16.4 (15.1-17.7)			Men: 1.6 (0.5, 2.7) Women: 0.6 (-0.7, 1.9)

[a]95% confidence intervals in brackets where reported;

Spiers GF, *et al. BMJ Open* 2021; 11:e045567. doi: 10.1136/bmjopen-2020-045567

Table 4d. Summary of disability prevalence trends data for UK studies and studies with UK samples[a,b]

Study	Trend	Time points	Change between first and last time point
Jagger 2016	Odds of any disability at time 2 compared to time 1	T1: 1991	1.22 (1.14-1.30)
	Odds of moderate-severe disability at time 2 compared to time 1	T2: 2011	0.76 (0.70-0.82)
	Odds of fair or poor self-rated health at time 2 compared to time 1		0.83 (0.78–0.88)
Kingston 2017	% increase in projected number of people with high dependency	T1: 2015	61.5
	% increase in projected number of people with medium dependency	T2: 2025	66.6
	% increase in projected number of care home places needed for those with high dependency	T3: 2035	84.5
	% increase in projected number of care home places needed for those with medium dependency		89.3
Kingston 2018	Relative change in projected proportion (%) of sample with high dependency (65-74 years)	T1: 2015	−15.0 (−20.0 to −13.0)
	Relative change in projected proportion (%) of sample with high dependency (75 - 84 years)	T2: 2025	42.0 (36.6- 42.7)
	Relative change in projected proportion (%) of sample with high dependency (85+ years)	T3: 2035	91.8 (87.3- 94.1)
	Relative change in projected proportion (%) of sample with high dependency (65+)		36.0 (32.6- 36.0)
Guzman-Castillo 2017	Relative change in projected number of disability cases 2015-2025, 65+	T1: 2015	25.0 (21.3–28.2)
	Relative change in projected number of disability cases 2015-2025, 65-84	T2: 2025	18.9 (16.6–20.9)
	Relative change in projected number of disability cases 2015-2025, 85		43.2 (34.2–52.1)

[a]Estimates for each time point were not extracted here due to volume. Reader is referred to publications and associated supplementary materials; [b]95% confidence intervals in brackets where reported;

Spiers GF, *et al. BMJ Open* 2021; 11:e045567. doi: 10.1136/bmjopen-2020-045567

Table 4e. Summary of inequalities in life expectancy trends, reported as the slope index of inequality between those living in the most and least deprived decile of area deprivation

Study	Trend	Time points	Change between first and last time point (range)
ONS 2019	Change in SSI in life expectancy at birth, England	T1: 2012/14<br>T2: 2015/17	Men: 0.3 (0.2)<br>Women: 0.5 (0.5)
	Change in SSI in life expectancy at age 65, England		Men: 0.3 (0.2)<br>Women: 0.5 (0.5)
	Change in SSI in life expectancy at birth, Wales		Men: 0.5 (0.3)<br>Women: 0.6 (0.7)
	Change in SSI in life expectancy at age 65, Wales		Men: 0.3 (0.2)<br>Women: 0.1 (0.3)

Spiers GF, *et al. BMJ Open* 2021; 11:e045567. doi: 10.1136/bmjopen-2020-045567

Table 4f. Summary of inequalities in healthy life expectancy trends, reported as the slope index of inequality between those living in the most and least deprived decile of area deprivation

Study	Trend	Time points	Change between first and last time point (range)
ONS 2019	Change in SSI in healthy life expectancy at birth, England	T1: 2012/14<br>T2: 2015/17	Men: 0.2 (0.2)<br>Women: -0.9 (-0.8)
	Change in SSI in healthy life expectancy at age 65, England		Men: 0.4 (0.9)<br>Women: -0.3 (-0.6)
	Change in SSI in healthy life expectancy at birth, Wales		Men: 0.2 (0.9)<br>Women: -1.1 (-0.9)
	Change in SSI in healthy life expectancy at age 65, Wales		Men: -0.6 (0.8)<br>Women: -1.0 (0.2)

Spiers GF, *et al. BMJ Open* 2021; 11:e045567. doi: 10.1136/bmjopen-2020-045567

Table 5a. Summary of life expectancy trends data for studies from Europe (years)[a]

**Study**	**At age**	**Time points**	**T1**		**T2**		**T3**		**T4**		**T5**		**T6**		**T7**		**T8**		**Change between first and last time point**
			**M**	**F**	**M**	**F**	**M**	**F**	**M**	**F**	**M**	**F**	**M**	**F**	**M**	**F**	**M**	**F**	
Bronnum-Hansen 2017	65	T1: 2006-2007<br>T2: 2010-2011<br>T3: 2013-2014	High: 17.9<br>Med: 16.3<br>Low: 15.6	High: 20.4<br>Med: 19.4<br>Low: 18.5	Med: 16.8<br>Low: 16.2	High: 21.0<br>Med: 20.0<br>Low: 18.9	High: 19.3<br>Med: 17.5<br>Low: 16.9	High: 21.7<br>Med: 20.6<br>Low: 19.5											Difference in change in LE between high and low education over time:<br>Men: 0.1<br>Women: 0.3
Deeg 2018	65	T1: 1993<br>T2: 1996<br>T3: 1999<br>T4: 2002<br>T5: 2006<br>T6: 2009<br>T7: 2012<br>T8: 2016	14.7	19.2	15.1	19.4	15.5	19.5	16	19.7	17.1	20.5	17.8	21.1	18.2	21.2	18.7	21.4	Men: 4.0<br>Women: 2.2
Gheorghe 2016[b]	25	T1: 2001<br>T2: 2011	High: 53.83<br>Med: 50.70<br>Low: 47.85	High: 58.75<br>Med: 56.82<br>Low: 53.57	High: 56.71<br>Med: 53.92<br>Low: 50.28	High: 60.53<br>Med: 58.35<br>Low: 54.72													Men:<br>High: 2.88<br>Med: 3.22<br>Low: 2.43<br><br>Women:<br>High: 1.78<br>Med: 1.53<br>Low: 1.15
	65		High: 16.81<br>Med: 15.29<br>Low: 14.08	High: 21.00<br>Med: 20.08<br>Low: 18.26	High: 19.29<br>Med: 17.53<br>Low: 15.76	High: 22.64<br>Med: 21.49<br>Low: 19.38													Men:<br>High: 2.48<br>Med: 2.24<br>Low: 1.68<br><br>Women:<br>High: 1.64<br>Med: 1.41<br>Low: 1.12
Remund 2019	30	T1: 1990/94<br>T2: 1995/99<br>T3: 2000/04<br>T4: 2005/09<br>T5: 2010/14	**data only reported in graphs, unable to extract**																Men: 5.02<br>Women: 3.09

Study	At age	Time points	T1		T2		T3		T4		T5		T6		T7		T8		Change between first and last time point
			M	F	M	F	M	F	M	F	M	F	M	F	M	F	M	F	
Renard 2019[b]	25	T1: 2001<br>T2: 2011	High: 55.82<br>Med: 53.18<br>Low: 50.63	High: 60.90<br>Med: 59.14<br>Low: 57.14	High: 57.81<br>Med: 54.73<br>Low: 51.74	High: 61.90<br>Med: 60.09<br>Low: 57.32													Men:<br>Low: 1.11<br>Med: 1.55<br>High: 1.99<br><br>WOMEN:<br>Low: 0.18<br>Med: 0.95<br>High: 1.00
Reus-Pons 2017	50	T1: 2001<br>T2: 2011	28.05	32.47	30.87	34.31													Men: 2.82 [d]<br>Women: 1.84 [d]
Storeng 2018	30	T1: 1984/86<br>T2: 1995/97<br>T3: 2006/08	45.1 (44.6–45.5)	50.4 (49.9–50.8)	48.3 (47.8–48.7)	52.9 (52.5–53.4)	52.1 (51.5–52.6)	57.1 (56.6–57.6)											Men: 6.99 (5.27–8.72)<br>Women: 6.75 (5.16–8.34)
			Primary: 47.0 (46.5–47.5)<br>Secondary: 47.9 (47.4–48.5)<br>Tertiary: 51.2 (49.6–52.7)		Primary: 49.7 (49.1–50.2)<br>Secondary: 50.9 (50.4–51.4)<br>Tertiary: 52.0 (50.9–53.1)		Primary: 52.2 (51.0–53.4)<br>Secondary: 54.9 (54.3–55.4)<br>Tertiary: 55.2 (53.1–57.4)												Primary: 5.19 (3.52–6.85)<br>Secondary: 6.95 (5.87–8.02)<br>Tertiary: 3.23 (–0.04–6.51)
Sundberg 2016	77	SWEOLD<br>T1: 1992<br>T2: 2002<br>T3: 2004<br>T4: 2011	8.2	10.7	8.9	10.8	9.3	11.4	9.9	11.8									Men: 1.7 [d]<br>Women: 1.1 [d]
		SHARE:<br>T1: 2004<br>T2: 2011	9.3	11.4	9.9	11.8													Men: 0.6 [d]<br>Women: 0.4 [d]
Yokota 2016	15	T1: 2001<br>T2: 2004<br>T3: 2008	60.5	66.4	61.4	67	62.1	67.4											Men: 1.6 [d]<br>Women: 1.0[d]

[a]95% confidence intervals in brackets where reported; [b] Trends reported by high, medium and low education; [c]Trends not reported by sex; [d]Change estimate not reported, and thus calculated by us (T^2-T^1)

Table 5b. Summary of healthy life expectancy trends data for studies from Europe[a]

Study	Trend	At age	Time points	T1		T2		T3		T4		T5		T6		T7		T8		Change between first and last time point
				M	F	M	F	M	F	M	F	M	F	M	F	M	F	M	F	
Deeg 2018	% life in good physical health	65	T1: 1993<br>T2: 1996<br>T3: 1999<br>T4: 2002<br>T5: 2006<br>T6: 2009<br>T7: 2012<br>T8: 2016	46.6	28.7	37.6	26.0	33.6	24.5	32.4	22.7	32.7	21.2	30.5	20.2	22.8	14.9	25.0	18.9	Men: -21.6<br>Women: -9.8
	HLE: Physical (years)			6.9	5.5													4.7	4.0	Men: -2.2[c]<br>Women: -1.5[c]
Gheorghe 2016[b]	Quality Adjusted Life Expectancy	25	T1: 2001<br>T2: 2011	High: 44.08<br>Med: 40.82<br>Low: 36.71	High: 45.79<br>Med: 44.2<br>Low: 39.51	High:4 6.93<br>Med: 43.83<br>Low: 38.84	High: 48.43<br>Med: 45.77<br>Low: 41.31													MEN:<br>High 2.85<br>Med: 3.01<br>Low: 2.13<br><br>WOMEN:<br>High: 2.64<br>Med: 1.57<br>Low: 1.80
		65		High: 13.42<br>Med: 12.06<br>Low: 10.73	High: 15.66<br>Med: 15.12<br>Low: 13.09	High: 15.59<br>Med: 13.97<br>Low: 12.10	High: 17.40<br>Med: 16.33<br>Low: 14.23													MEN:<br>High: 2.17<br>Med: 1.91<br>Low: 1.37<br><br>WOMEN:<br>High: 1.74<br>Med: 1.21<br>Low: 1.14
Remund 2019	HLE (years)	30	T1: 1990/94<br>T2: 1995/99<br>T3: 2000/04<br>T4: 2005/09	**data only reported in graphs, unable to extract**																Men: 4.52<br>Women: 3.09

Spiers GF, *et al. BMJ Open* 2021; 11:e045567. doi: 10.1136/bmjopen-2020-045567

Study	Trend	At age	Time points	T1		T2		T3		T4		T5		T6		T7		T8		Change between first and last time point
				M	F	M	F	M	F	M	F	M	F	M	F	M	F	M	F	
	HLE (years), educational gap between primary and tertiary attainment		T5: 2010/14	7.6	3.3	**not reported**						8.8	5.0							Change in educational gap (between primary and tertiary): Men: 1.2[c] Women: 1.7[c]
Reus-Pons 2017	HLE (years)	50	T1: 2001<br>T2: 2011	18.62 (18.40 - 18.84)	19.43 (19.19 - 19.68)	20.83 (20.55 - 21.10)	20.68 (20.37 - 20.99)													Men: 2.21<br>Women: 1.25
Storeng 2018[d]	HLE (years)	30	T1: 1984/86<br>T2: 1995/97<br>T3: 2006/08	31.6 (31.2–31.9)	32.6 (32.3–33.0)	34.1 (33.7–34.5)	33.9 (33.6–34.3)	38.5 (38.0–39.0)	38.0 (37.5–38.5)											Men: 6.90 (6.08–7.73)<br>Women: 5.40 (4.56–6.25)
				Primary:29.5 (29.2–29.9)<br>Secondary:34.0 (33.6–34.5)<br>Tertiary: 40.1 (38.8–41.4)		Primary:29.8 (29.4–30.3)<br>Secondary: 35.7 (35.3–36.2)<br>Tertiary: 40.3 (39.3–41.3)		Primary: 31.5 (30.6–32.4)<br>Secondary:38.8 (38.3–39.4)<br>Tertiary: 42.8 (41.9–43.8)												Primary: 1.94 (0.68–3.20)<br>Secondary: 4.80 (3.85–5.75)<br>Tertiary: 2.76 (0.49–5.02)<br><br>Change in gap between primary and tertiary education: 0.7[c]

[a]95% confidence intervals in brackets where reported; [b]Trends reported by high, medium and low education; [c]Change estimate not reported, and thus calculated by us (T^2-T^1); [d]Trends reported by primary, secondary and tertiary education;

Table 5c. Summary of disability-free life expectancy trends data for studies from Europe (years)[a]

**Study**	**Trend**	**At age**	**Time points**	**T1**		**T2**		**T3**		**T4**		**Change between first and last time point**
				**Male**	**Female**	**Male**	**Female**	**Male**	**Female**	**Male**	**Female**	
Bronnum-Hansen 2017[b]	DFLE	65	T1: 2006-2007<br>T2: 2010-2011<br>T3: 2013-2014	High: 10.6 (9.2–12.1)<br>Med: 9.4 (8.3–10.5)<br>Low: 7.4 (5.9–8.9)	High: 12.5 (10.5–14.5)<br>Med: 9.5 (8.1–10.9)<br>Low: 8.8 (7.7–9.9)	High: 10.6 (9.2–12.0)<br>Med: 9.3 (8.2–10.4)<br>Low: 7.0 (5.3–8.8)	High: 13.2 (10.9–15.6)<br>Med: 10.4 (8.9–11.8)<br>Low: 7.9 (6.6–9.3)	High: 10.9 (9.9–12.0)<br>Med: 9.6 (8.8–10.5)<br>Low: 8.0 (6.7–9.3)	High: 12.9 (11.2–14.6)<br>Med: 10.2 (9.0–11.4)<br>Low: 9.5 (8.5–10.5)			Difference in change in DFLE between those with the lowest and highest education<br>Men: -0.3<br>Women: -0.3
Lagergren 2017	Added years of life without ADL limitations	65+	T1: 1980/85<br>T2: 1994/99<br>T3: 2006/11	**data not reported**								Men: 4.0<br>Women: 3.14
	Added years of life without mobility limitations			**data not reported**								Men: 4.21<br>Women: 3.69
Renard 2019	DFLE	25	T1: 2001<br>T2: 2011	Low: 36.34<br>Med: 41.40<br>High: 42.85	Low: 39.32<br>Med: 43.37<br>High: 48.62	Low:37.0 2<br>Med: 42.05<br>High: 47.49	Low:35.5 4<br>Med:42.9 8<br>High: 48.98					Difference in change in DFLE between levels of education (ref high education) change over time<br>Men:<br>Low: 3.96<br>Med: 3.99

**Study**	**Trend**	**At age**	**Time points**	**T1**		**T2**		**T3**		**T4**		**Change between first and last time point**
				**Male**	**Female**	**Male**	**Female**	**Male**	**Female**	**Male**	**Female**	
												Women: Low: 4.14 Med: 0.76
Storeng 2018[e]	DFLE	30	T1: 1984/86 T2: 1995/97 T3: 2006/08	25.9 (25.6–26.2)	28.8 (28.6–29.1)	28.5 (28.1–28.8)	29.4 (29.0–29.7)	28.6 (28.2–29.0)	29.2 (28.8–29.6)			Men: 2.71 (2.01–3.42) Women: 0.33 (–0.40–1.06)
				Primary: 25.0 (24.7–25.3) Secondary: 28.8 (28.5–29.2) Tertiary: 33.6 (32.5–34.7)		Primary: 25.3 (24.9–25.7) Secondary: 30.1 (29.7–30.5) Tertiary: 33.0 (32.3–33.8)		Primary: 22.5 (21.7–23.2) Secondary: 29.1 (28.6–29.5) Tertiary: 33.2 (32.4–34.1)				Primary: –2.53 (–3.61–1.45) Secondary: 0.23 (–0.57–1.03) Tertiary: –0.32 (–2.23–1.60)
Sundberg 2016	DFLE	77	SWEOLD T1: 1992 T2: 2002 T3: 2004 T4: 2011	6.7 (6.3–7.1)	6.9 (6.4–7.4)	6.9 (6.4–7.3	7.0 (6.5–7.5)	7.4 (7.0–7.9)	7.9 (7.4–8.4)	7.8 (7.2–8.3)	8.5 (8.0–9.0	Men: 1.1 Women: 1.6
			SHARE: T1: 2004 T2: 2011	7.5 (7.0–8.0	7.9 (7.3–8.6	7.6 (7.0–8.1)	9.2 (8.5–9.8)					Men: 0.1 Women: 1.3
Yokota 2019	DFLE	15	T1: 2001 T2: 2004 T3: 2008	54.1	56.0	54.9	56.2	54.8	55.3			Men: 0.7 Women: -0.7

[a]95% confidence intervals in brackets where reported; [b]Stratified by high, medium and low education; [c]Not stratified by sex; [d]Change estimate not reported, and thus calculated by us (T^2-T^1) [e]Second row stratified by primary, secondary and tertiary education for men and women combined

Table 5d. Summary of disability prevalence trends data for studies from Europe

Study	Trend	Time points	Change between first and last time point[a]
Lagergren 2017	Proportion reporting dependency in activities of daily living, 65-69	T1:1980/85<br>T2: 1994/95<br>T3: 2006/11	Men:-1.5<br>Women:-3.7
	Proportion reporting dependency in activities of daily living, 70-74		Men:-5.6<br>Women:-3.5
	Proportion reporting dependency in activities of daily living, 75-79		Men:-10.7<br>Women:-7.1
	Proportion reporting dependency in activities of daily living, 80-84		Men: -11.5<br>Women: -14.0
	Proportion reporting dependency in activities of daily living, 85+		Men:-20.3<br>Women: -10.5
	Proportion reporting mobility limitations, 65-69		Men:-6.5<br>Women: -8.3
	Proportion reporting mobility limitations, 70-74		Men: -10.9<br>Women: -8.5
	Proportion reporting mobility limitations, 75-79		Men: -15.7<br>Women: -14.8
	Proportion reporting mobility limitations, 80-84		Men: -15.1<br>Women: -19.4
	Proportion reporting mobility limitations, 85+		Men: -16.1<br>Women: -17.5

[a]Change estimate not reported, and thus calculated by us (T^2-T^1)

Spiers GF, *et al. BMJ Open* 2021; 11:e045567. doi: 10.1136/bmjopen-2020-045567

Table 6a. Summary of life expectancy trends data for studies from Japan and Korea (years)[a]

Study	At age	Time points	T1		T2		T3		T4		T5		T6		T7		T8		Change between first and last time point
			M	F	M	F	M	F	M	F	M	F	M	F	M	F	M	F	
Jo 2019	0	T1: 2005<br>T2: 2007<br>T3: 2008<br>T4: 2009<br>T5: 2010<br>T6: 2011<br>T7: 2012	75.13	81.87	76.13	82.71	76.54	83.27	76.99	83.74	77.19	84.04	77.64	84.42	77.94	84.61	78.51	85.02	Men: 3.38[b] Women: 3.15[b]
Lee 2016	0	T1: 2005<br>T2: 2008<br>T3: 2011	75.1	81.8	76.5	83.2	77.6	84.4											Men: 2.5[b] Women: 2.6[b]
	65		15.8	19.8	16.6	21.0	17.4	21.8											Men: 1.6[b] Women: 2.0[b]
	85		5.1	6.2	5.2	6.5	5.4	6.9											Men: 0.3[b] Women: 0.7[b]
Sugawara 2016	0	T1: 2000<br>T2: 2010	77.6	84.7	79.5	86.4													Men: 1.9 Women:1.7
Tokudome 2016	0	T1: 1990<br>T2: 1995<br>T3: 2000<br>T4: 2005<br>T5: 2010<br>T6: 2013	76.04 (75.98–76.10	81.96 (81.86–82.05	76.45 (76.14–76.57	82.84 (82.62–82.94	77.55 (77.53–77.58	84.32 (84.29–84.35	78.66 (78.60–78.71	85.48 (85.41–85.54	79.34 (79.31–79.36	85.09 (86.06–86.11	80.05 (79.26–80.84	86.39 (85.74–87.12					Men: 4.01[b] Women: 4.43[b]

[a]95% confidence intervals in brackets where reported; [b]Change estimate not reported, and thus calculated by us (T^2-T^1)

Table 6b. Summary of healthy life expectancy trends data for studies from Japan and Korea (years)[a]

**Study**	**At age**	**Time points**	**T1**		**T2**		**T3**		**T4**		**T5**		**T6**		**T7**		**T8**		**Change between first and last time point**
			**M**	**F**	**M**	**F**	**M**	**F**	**M**	**F**	**M**	**F**	**M**	**F**	**M**	**F**	**M**	**F**	
Jo 2019	0	T1: 2005<br>T2: 2007<br>T3: 2008<br>T4: 2009<br>T5: 2010<br>T6: 2011<br>T7: 2012	70.8	74.7	71.86	73.92	72.62	75.18	73.4	76.65	73.87	77.21	74.33	76.53	74.71	78.3	74.83	78.14	Men: 4.03<br>Women: 3.44
Lee 2016	0	T1: 2005<br>T2: 2008<br>T3: 2011	64.4	67.7	64.0	67.3	65.8	68.9											Men: 1.4[b]<br>Women: 1.2[b]
	65		9.5	11.5	10.4	12.8	11.7	14.1											Men: 2.2[b]<br>Women: 2.6[b]
	85		2.6	3.8	3.3	4.7	4.0	5.2											Men: 1.4[b]<br>Women: 1.4[b]
Tokudome 2016	0	T1: 1990<br>T2: 1995<br>T3: 2000<br>T4: 2005<br>T5: 2010<br>T6: 2013	68.09 (65.83–70.11)	72.24 (69.38–74.77)	68.44 (66.07–70.58)	72.92 (70.05–75.49)	69.08 (66.67–71.24)	73.95 (70.94–76.55)	69.89 (67.31–72.12)	74.77 (71.66–77.46)	70.78 (68.20–73.06)	75.41 (72.34–78.23)	71.11 (68.50–73.57))	75.56 (72.46–78.42)					Men: 3.02[b]<br>Women: 3.32[b]

[a]95% confidence intervals in brackets where reported; [b]Change estimate not reported, and thus calculated by us (T^2-T^1)

Table 6c. Summary of disability-free life expectancy trends data for studies from Japan[a]

Study	Trend	At age	Time points	T1		T2		Change between first and last time point
				Male	Female	Male	Female	
Sugawara 2016	DFLE (years)	0	T1: 2000<br>T2: 2010	68.0	71.7	69.0	72.1	Men: 1.0<br>Women: 0.4
	% of life disability-free			87.6	84.6	86.8	83.4	Men: -0.8<br>Women: -1.2

[a]95% confidence intervals in brackets where reported;

Table 7a. Summary of life expectancy trends data high income countries combined from the GBD studies (years)[a]

Study author and date	At age	Time points	T1		T2		Change between first and last time point
			Male	Female	Male	Female	
GBD 2016	0	T1: 2005<br>T2: 2015	76·35 (76·33–76·37)	82·09 (82·07–82·11)	78·10 (78·05–78·15)	83·42 (83·38–83·46)	Men: 1.75[b]<br>Women: 1.33[b]
GBD 2017	0	T1: 1990<br>T2: 2016	72·64 (72·54–72·75)	79·34 (79·24–79·43)	78·27 (78·04–78·49)	83·48 (83·28–83·67)	Men: 5.63[b]<br>Women: 4.14[b]
	65		14·96 (14·90–15·01)	18·77 (18·71–18·84)	18·47 (18·32–18·60)	21·80 (21·66–21·94)	Men: 3.51[b]<br>Women: 3.03[b]
GBD 2018	0	T1: 1990<br>T2: 2017	72·8 (72·7–72·8)	79·4 (79·4–79·4)	78·4 (78·2–78·6)	83·6 (83·4–83·7)	Men: 5.6[b]<br>Women: 4.2[b]

[a]95% confidence intervals in brackets where reported; [b]Change estimate not reported in publication, and calculated by us (T^2-T^1);

Spiers GF, *et al. BMJ Open* 2021; 11:e045567. doi: 10.1136/bmjopen-2020-045567

Table 7b. Summary of healthy life expectancy trends data for high income countries combined from the GBD studies[a]

Study	Trend	At age	Time points	T1		T2		Change between first and last time point
				Male	Female	Male	Female	
GBD 2016	HLE (years)	0	T1: 2005<br>T2: 2015	67.48 (64.90–69.75)	71.13 (67.95–73.87)	68.91 (66.27–71.25)	72.21 (68.97–75.02)	Men: 1.43[b]<br>Women: 1.08[b]
GBD 2017	HLE (years)	0	T1: 1990<br>T2: 2016	64.15 (61.70–66.33)	68.40 (65.23–71.19)	68.58 (65.82–71.06)	71.61 (68.12–74.66)	Men: 4.43[b]<br>Women: 3.21[b]
		65		11.0 (10.45–12.27)	14.28 (13.06–15.39)	14.00 (12.79–15.11)	16.55 (15.09–17.85)	Men: 2.60[b]<br>Women: 2.27[b]
GBD 2018	HLE (years)	0	T1: 1990<br>T2: 2017	64.0 (61.4–66.1)	68.0 (64.7–70.8)	68.2 (65.3–70.7)	71.0 (67.5–74.0)	Men: 3.0[b]<br>Women: 4.2[b]

[a]95% confidence intervals in brackets where reported; [b]Change estimate not reported in publication, and calculated by us (T^2-T^1);

Search strategy as applied to Medline

Database(s): **Ovid MEDLINE(R) and In-Process & Other Non-Indexed Citations** 1946 to September 13, 2019

#	Searches	Results
1	Life Expectancy/	16893
2	Longevity/	20149
3	Needs assessment/	28915
4	((expectancy or Dependen* or disabilit*) adj2 years).ti,ab.	968
5	(expectanc* adj5 disabilit*).ti,ab.	406
6	(Care need* adj2 years).ti,ab.	13
7	(healthy life years or "years of healthy life").ti,ab,kw.	260
8	or/1-7	65417
9	exp Age Factors/ or Race Factors/ or Sex Factors/	631787
10	Time Factors/	1159988
11	exp Forecasting/	82762
12	Life Tables/	6387
13	td.fs.	367819
14	or/9-13	2116951
15	8 and 14	14752
16	Life Expectancy/td [Trends]	2146

17	Needs Assessment/td [Trends]	249
18	((life expectancy or expected years or care need*) adj2 (trend* or direction* or factor* or forecast*)).ti,ab.	334
19	or/16-18	2649
20	15 or 19	14962
21	limit 20 to (humans and yr="2016 -Current")	1661
22	letter/	1034636
23	Editorial/	498100
24	News/	196781
25	exp Historical Article/	390531
26	Anecdotes as Topic/	4730
27	Comment/	799622
28	(letter or comment$).ti.	138712
29	or/22-28	2365158
30	21 not 29	1502

Spiers GF, *et al. BMJ Open* 2021; 11:e045567. doi: 10.1136/bmjopen-2020-045567
