# Supplementary material for: Trends in health expectancies: a systematic review of international evidence
Source: BMJ Open. 2021 May 25;11(5):e045567. doi: 10.1136/bmjopen-2020-045567 (PMC8154999; doi:10.1136/bmjopen-2020-045567)
Supplement: Supplementary data [file bmjopen-2020-045567supp002.pdf]

Supplementary materials Figure 1. Expansion and compression of poor health/disability by country and time period

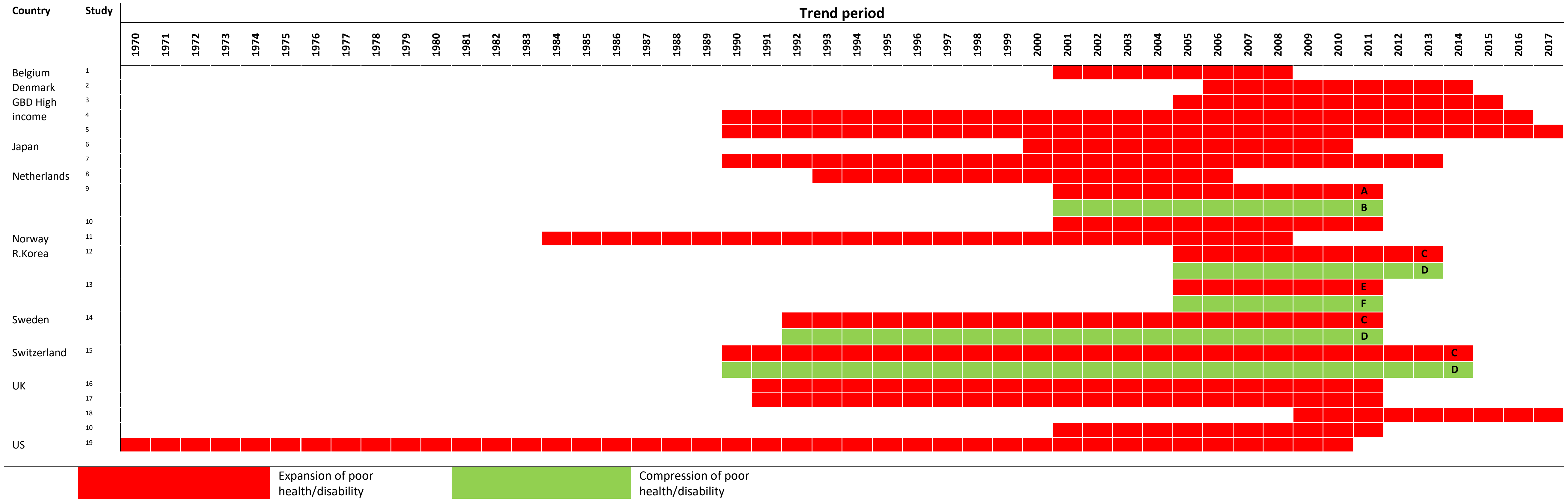

<u>Where multiple studies in countries or conclusions differ between sub-groups:</u> [a]Men, 25 and 65, all levels of education; [b]Women (age 25, all levels of education; age 65, high and low education); [c]Men; [d]Women; [e]At birth; [f]Age 65 and 86
