# Supplementary material for: Trends in health expectancies: a systematic review of international evidence
Source: BMJ Open. 2021 May 25;11(5):e045567. doi: 10.1136/bmjopen-2020-045567 (PMC8154999; doi:10.1136/bmjopen-2020-045567)
Supplement: Author's manuscript [file bmjopen-2020-045567.draft_revisions.pdf]

BMJ Open is committed to open peer review. As part of this commitment we make the peer review history of every article we publish publicly available.

When an article is published we post the peer reviewers' comments and the authors' responses online. We also post the versions of the paper that were used during peer review. These are the versions that the peer review comments apply to.

The versions of the paper that follow are the versions that were submitted during the peer review process. They are not the versions of record or the final published versions. They should not be cited or distributed as the published version of this manuscript.

BMJ Open is an open access journal and the full, final, typeset and author-corrected version of record of the manuscript is available on our site with no access controls, subscription charges or pay-per-view fees (http://bmjopen.bmj.com).

If you have any questions on BMJ Open's open peer review process please email info.bmjopen@bmj.com

BMJ Open

Trends in healthy and disability-free life expectancy: a systematic review of international evidence

Journal:	*BMJ Open*
Manuscript ID	bmjopen-2020-045567
Article Type:	Original research
Date Submitted by the Author:	05-Oct-2020
Complete List of Authors:	Spiers, Gemma; Newcastle University, Population & Health Sciences Institute<br>Kunonga, Tafadzwa; Newcastle University, Population & Health Sciences Institute<br>Beyer, Fiona ; Newcastle University, Population & Health Sciences Institute<br>Craig, Dawn; Newcastle University, Population & Health Sciences Institute<br>Hanratty, Barbara; Newcastle University, Population & Health Sciences Institute<br>Jagger, Carol; Newcastle University, Population & Health Sciences Institute
Keywords:	Health policy < HEALTH SERVICES ADMINISTRATION & MANAGEMENT, Organisation of health services < HEALTH SERVICES ADMINISTRATION & MANAGEMENT, GERIATRIC MEDICINE

SCHOLARONE™
Manuscripts

I, the Submitting Author has the right to grant and does grant on behalf of all authors of the Work (as defined in the below author licence), an exclusive licence and/or a non-exclusive licence for contributions from authors who are: i) UK Crown employees; ii) where BMJ has agreed a CC-BY licence shall apply, and/or iii) in accordance with the terms applicable for US Federal Government officers or employees acting as part of their official duties; on a worldwide, perpetual, irrevocable, royalty-free basis to BMJ Publishing Group Ltd ("BMJ") its licensees and where the relevant Journal is co-owned by BMJ to the co-owners of the Journal, to publish the Work in this journal and any other BMJ products and to exploit all rights, as set out in our licence.

The Submitting Author accepts and understands that any supply made under these terms is made by BMJ to the Submitting Author unless you are acting as an employee on behalf of your employer or a postgraduate student of an affiliated institution which is paying any applicable article publishing charge ("APC") for Open Access articles. Where the Submitting Author wishes to make the Work available on an Open Access basis (and intends to pay the relevant APC), the terms of reuse of such Open Access shall be governed by a Creative Commons licence – details of these licences and which Creative Commons licence will apply to this Work are set out in our licence referred to above.

Other than as permitted in any relevant BMJ Author's Self Archiving Policies, I confirm this Work has not been accepted for publication elsewhere, is not being considered for publication elsewhere and does not duplicate material already published. I confirm all authors consent to publication of this Work and authorise the granting of this licence.

For peer review only

Trends in healthy and disability-free life expectancy: a systematic review of international evidence

Dr Gemma Spiers,[a]* gemma-frances.spiers@newcastle.ac.uk

Ms Tafadzwa Patience Kunonga[a]

Ms Fiona Beyer[b]

Professor Dawn Craig[b]

Professor Barbara Hanratty[a]

Professor Carol Jagger[a]

[a] Population and Health Sciences Institute, Newcastle University, Campus for Ageing & Vitality, Newcastle upon Tyne, NE4 5PL

[b] Population and Health Sciences Institute, Newcastle University, Baddiley-Clark, Newcastle upon Tyne, NE2 4AX

*Corresponding author

Word count: 2915 excluding abstract, references, tables and figures

Number of references: 43

Number of tables and figures: 4

Funding acknowledgement

National Institute for Health Research (NIHR) Policy Research Programme conducted through the NIHR Older People and Frailty Policy Research Unit, PR-PRU-1217-21502. The views expressed are those of the authors and not necessarily those of the NIHR or the Department of Health and Social Care.

Author contributions

Author contributions: CJ planned the study, developed the protocol, and co-wrote the paper; GS developed the protocol, contributed to all stages of the review, and co-wrote the paper; PK developed the protocol, contributed to all stages of the review, and co-wrote the paper, FB developed the protocol, developed and undertook the searches, and co-wrote the paper; DC developed the protocol, oversaw all stages of the review, and co-wrote the paper; BH developed the protocol and co-wrote the paper.

For peer review only - http://bmjopen.bmj.com/site/about/guidelines.xhtml

Trends in healthy and disability-free life expectancy: a systematic review of international evidence

BACKGROUND

A clear understanding of whether increases in longevity are spent in good health is necessary to support ageing, health and care-related policy. We conducted a systematic review to update and summarise evidence on trends in healthy and disability-free life expectancy, in OECD high-income countries.

METHODS

Four electronic databases, and the UK Office for National Statistics website, were searched for English language studies published from 2016 that reported trends in healthy, active, and/or disability-free life expectancy in an OECD high-income country. Records were screened independently by two researchers. Study quality was assessed using published criteria designed to identify sources of bias in studies reporting trends, and evidence summarised by narrative synthesis.

FINDINGS

Thirty publications from twelve countries were included, covering periods from six to 40 years, between 1970 and 2017. In most countries, gains in healthy and disability-free life expectancy do not match the growth in total life expectancy. Exceptions were demonstrated for women in Sweden, where there were greater gains in disability-free years than life expectancy. Gains in healthy and disability-free life expectancy were greater for men than women in most countries except the US (age 85), Japan (birth), Korea (age 65) and Sweden (age 77).

CONCLUSION

An expansion of disability in later life is evident in a number of high-income countries, with implications for the sustainability of health and care systems. The recent COVID-19 pandemic is likely to further impact health expectancies in the longer term.

Keywords: Trends, Life Expectancy, Disability, Health, Ageing

For peer review only

Summary Box

Strengths and limitations of this study

- To our knowledge, this is the first systematic review using reproducible methods to synthesise evidence about healthy life expectancy and report quantitative comparisons of life and health expectancies to differentiate compression or expansion of morbidity.
- We used analyses published from 2016 to focus on the most contemporary evidence in trends.
- The quality of evidence was judged using criteria designed to assess threats to the validity of trends over time. However, due to the absence of methodological detail reported, it was not possible to give a clear judgement of study quality and bias for around half of the studies included in the review.

For peer review only

For peer review only - http://bmjopen.bmj.com/site/about/guidelines.xhtml

BACKGROUND

Populations are ageing worldwide. Globally, the proportion of those aged 65 and over has increased by 9 per cent in the last two decades, and is expected to grow by a further 16% by 2050.[1] This demographic shift will require societies to adapt, and governments are facing the challenge of providing accessible, high quality and sustainable long-term care for growing numbers of older people.[2-4] The growth in life expectancy is a positive, but with this comes a responsibility to ensure people have the support they need as they age, and to facilitate ageing in place.[5]

In 2019, the World Health Organisation renewed its commitment to support countries to achieve longer and healthier lives with the Decade of Healthy Ageing 2020-2030 strategy.[6] A critical part of achieving longevity is understanding whether longer lives are typified by more years spent in good health (compression of disability) or poor health (expansion of disability). This has important implications for the provision of health and care services to respond to the needs of people as they age. It is therefore crucial to keep abreast of trends, specifically how the growth in life expectancy is matched by a growth in years spent in good health. Metrics to assess this include *healthy life expectancy* and *disability-free life expectancy*. Both provide an estimate of life expectancy spent in good health, but differ slightly with respect to their measurement. Healthy life expectancy tends to rely on single item questions of self-reported health, and is thus subject to fluctuations as expectations of health change over time.[7] Disability-free life expectancy is often calculated from multiple items about activity limitations and/or dependencies.[8] and therefore does not bear the same limitations as that of healthy life expectancy.

Previous reviews have summarised trends in total, healthy and disability-free life expectancy, the most recently in 2016.[7, 9] Typically, such evidence shows that whilst people are living longer, gains in life expectancy are not consistently matched by a growth in the number of years lived in good health and free of disability. Nevertheless, this is an evolving evidence base requiring ongoing scrutiny. Longitudinal datasets, which often form the bedrock of the analysis of these trends, are continually acquiring new data, whilst in the UK, the Office of National Statistics (ONS) updates and publishes trends yearly. Regardless, the pressing policy relevance of these issues globally warrants an updated overview of trends in healthy and disability-free life expectancy. Understanding these issues in a global

For peer review only

For peer review only - http://bmjopen.bmj.com/site/about/guidelines.xhtml

context is equally important in order to identify, and learn from, those countries whose populations are living healthier for longer. To obtain an up to date understanding of population trends in life expectancy and healthy ageing, we undertook a systematic review to a) synthesise evidence about trends in healthy and disability-free life expectancy in high-income countries, and b) assess whether such trends are keeping pace with total life expectancy.

METHODS

The methods used for this systematic review are reported according to the PRISMA checklist and guidance.[10]

Search strategy

A search strategy was developed using the concepts [*life expectancy*] AND [*trends* OR *impacting factors*]. The search was designed in MEDLINE using thesaurus headings and title, abstract and keyword field terms, and these elements translated to other databases. Electronic searches were carried out in MEDLINE (OVID), Embase (OVID), Scopus and Health Management Information Consortium (OVID), in October 2019 (see the supplementary materials for the strategy applied to MEDLINE). As this systematic review updated previous reviews,[7, 9] searches were limited to studies published from 2016. The ONS website was also hand searched (November 2019) for reports published from 2016.

Review criteria

Review criteria are summarised in table 1. Studies were included if they examined trends (i.e. more than one time point) in: healthy life expectancy; active life expectancy; or disability-free life expectancy; in an Organisation for Economic Co-operation and Development (OECD) high-income country.[11] OECD high-income countries were selected for this review for comparability. Studies reporting trends in life expectancy *only* were ineligible. Where studies also reported prevalence of disability, dependency or self-rated health alongside the above outcomes, these were reported for context. For ONS reports, the most up-to-date analyses were included, with reports excluded if they were superseded by a more recently published analysis of the same data.

For peer review only - http://bmjopen.bmj.com/site/about/guidelines.xhtml

TABLE 1 HERE

Study selection

Titles and abstracts of all records were screened for relevance. The full texts of publications selected as relevant were retrieved and assessed for inclusion against the review criteria. Both stages of screening were undertaken independently by two researchers, with disagreements resolved through consensus.

Quality appraisal

Study quality and bias were assessed using an adapted version of previously published criteria for studies reporting trends.[12] This approach assesses 'threats to the validity of comparisons over time' (p. 3140). Criteria were rated as *good*, *fair* or *poor*, according to the parameters summarised in table 2. Where study publications did not report the required information to assess quality, other associated publications (e.g. methodological and technical reports for the datasets used) were consulted. If the required information was not available from either of these sources, the criterion was assessed as *unclear*.

Using the assessments (good, fair, poor or unclear) for each criterion, studies were given a summary rating of quality. As studies were often based on summaries of cross-sectional data, they did not typically report information to assess the criterion *proportion lost to follow up.* We also found that studies did not typically report information to assess the criteria *proportion of proxy interviews* and *proportion of missing data.* The summary rating therefore considered only the first four criteria, using the following judgements:

- GOOD: studies with three out of four (one to include the quality of the outcome measure) criteria rated *good* and no criteria rated *poor* or *unclear*.
- FAIR: studies with two or more criteria rated *fair*, with no criteria rated *poor* or *unclear*.
- POOR: studies with any criteria rated *poor*.
- UNCLEAR: studies with any criteria rated *unclear*.

Where studies used multiple outcomes and were given different judgements for each, this was noted in the summary rating.

For peer review only - http://bmjopen.bmj.com/site/about/guidelines.xhtml

TABLE 2 HERE

Data extraction and synthesis

Study details (author, publication date, country, study design) and trend estimates (for each time point measured, and the change between the first and last time point) were extracted onto an Excel spreadsheet by one researcher. Fifty percent of studies were checked for accuracy by a second researcher. Where publications did not report the change between the first and last time point, we calculated this by subtracting the first time point estimate from the last time point estimate.

A narrative synthesis was used to summarise evidence on trends by outcome (healthy life expectancy, disability-free life expectancy, disability or dependency prevalence), supported by data summary tables. Estimates of changes in each healthy life expectancy and disability-free life expectancy were compared to changes in life expectancy. This comparison provides evidence of whether there had been an expansion (healthy or disability-free life expectancy gains are smaller than life expectancy gains) or compression (healthy or disability-free life expectancy gains are equivalent to or greater than life expectancy gains) of disability and ill health.

FINDINGS

Thirty studies met the review criteria (figure 1, and supplementary materials table 1). Most (eight) studies reported trends in the UK,[7, 8, 13-18] including England,[7, 8, 14, 15] England & Wales,[13, 16, 18] and each of the four devolved countries and the UK as a whole.[17, 19-21] Two of the UK studies were ONS reports.[16, 17] The remaining studies reported trends in Belgium,[18, 22, 23] Canada,[24] Denmark,[25] France,[26] Japan,[27, 28] the Netherlands,[29, 30] Norway,[31] Republic of Korea,[32, 33] Sweden,[34, 35] Switzerland,[36] and the US.[37-40] Three Global Burden of Disease studies were included, which reported trends across multiple countries, and high income countries combined.[19-21] For these three studies, we used data for all high income countries combined.

The assessment of study quality is detailed in supplementary materials table 2. Four studies were rated good,[8, 14, 15, 23] seven were rated fair,[13, 16, 17, 22, 25, 37, 38] one was rated good *and* fair (as it used two outcome measures that each received a different quality rating),[7] and three were rated poor.[31, 34, 36] Fifteen studies were rated unclear due to

For peer review only - http://bmjopen.bmj.com/site/about/guidelines.xhtml

a lack of information required to assess quality.[18-21, 24, 26-30, 32, 33, 35, 39, 40] As the majority of studies received a summary rating of unclear, the synthesis below does not prioritise evidence with a higher quality rating. Instead, the summary judgement is provided in supplementary materials table 2 for the reader's reference.

FIGURE 1 HERE

Across most studies, trends demonstrated a growth in healthy and disability-free life expectancy, with gains typically greater for men than women (supplementary materials tables 3a – 7b). Exceptions where gains were greater for women include the US (age 85),[38] Japan (birth),[28] Korea (age 65),[33] and Sweden (age 77).[35] One analysis of UK data over the more recent period of 2009/12 to 2015/17 also indicated that healthy life expectancy declined for women by 0.2 years.[16] Disability-free life expectancy at age 20 - 64 years also declined by 0.6 years between 1970 and 2010 for women in the US in one study.[38]

Is there evidence of an expansion or compression of disability?

Table 3 summarises the change in each life expectancy, healthy life expectancy and disability-free life expectancy across trend periods for each study where comparisons were possible. The comparison does not include studies where: the metric of change was not comparable between life expectancy and health expectancy;[22, 24] trends were reported as a slope of index inequality only;[17] only forecasts were reported;[13-15, 37] and life expectancy is reported only in a graph.[34, 39, 40]

For peer review only - http://bmjopen.bmj.com/site/about/guidelines.xhtml

TABLE 3 HERE

There is evidence from observed (i.e. not forecasted) trends in the UK that gains in healthy and disability-free life expectancy were smaller than gains in life expectancy. This is consistent with evidence of a reduction in the proportion of life spent without disability in the UK. This pattern was also observed in: all high income countries combined in the Global Burden of Disease studies,[19-21] and France,[26] Norway,[31] Belgium,[23] Japan,[27, 28] and the US[38] for all, and in Switzerland [36] and Sweden [35] for men but not women. This points to evidence of an expansion of disability in a number of high-income countries, although not always consistently between men and women.

Gains in disability-free life expectancy were greater than gains in life expectancy for women in Sweden, a finding that was evident in two different datasets.[35] This indicates a compression of disability for women in Sweden. In another study, gains in life expectancy and healthy life expectancy were similar for women in Switzerland,[36] which might also suggest a compression of disability. However, this finding should be interpreted with caution as the quality assessment highlighted a potential threat to the validity of the trend due to changes in the phrasing of, and response items for, the outcome measure between data waves.

Evidence from Korea and the Netherlands was less certain. Contrasting findings between studies gave an unclear picture about whether these countries had experienced a compression or expansion of disability. For example, in one study from Korea, gains in quality adjusted life expectancy at birth were greater than gains in life expectancy.[32] Another study, using the same data but a different measure (healthy life expectancy), reported that gains at birth still lagged behind those for life expectancy, although gains in healthy life expectancy at age 65 and 85 were slightly higher than gains in life expectancy over the study period.[33] Similarly, the two studies from the Netherlands also offered contrasting findings, [18, 30] again limiting any inference about whether there has been a compression or expansion of disability.

DISCUSSION

This systematic review was undertaken to update our current understanding of trends in healthy and disability-free life expectancy in OECD high-income countries. The principal

For peer review only - http://bmjopen.bmj.com/site/about/guidelines.xhtml

finding is that gains in healthy and disability-free life expectancy have not kept pace with the growth in life expectancy in a number of high income countries. One clear exception was Sweden, where gains in women's disability-free life expectancy were greater than gains in life expectancy, a finding evidenced from two different datasets within Sundberg and colleagues' study. This finding contrasts with an earlier review, which found evidence of a compression of disability for men, but not women, in Sweden:[9]this has now been reversed with an expansion of disability for men.[35] The equivalence of total and healthy life expectancy for women in Switzerland also indicates a compression of disability in Switzerland, although as noted earlier, this finding should be interpreted with caution. Nevertheless, based on the countries with data, our findings suggest that countries still need to make significant progress to achieve the World Health Organisation's Decade of Healthy Ageing goal of healthier, longer lives for all.

The observed expansion of disability and poor health has important implications for older people's health and care. Across many high-income countries, care services that support older people's day to day independence and quality of life are not universal but subject to payment barriers.[3, 4] Living longer with greater disability and in poor health signals a need for greater policy focus on ensuring older people have timely access to care. This is a particularly timely message for policy makers in the UK, as the All Party Parliamentary Group for Longevity set out recommendations to achieve the UK Government's goal of extending healthy life by 2035 and reducing inequalities.[41] Furthermore, the finding that women are living with longer periods of disability may warrant a particular focus on how services can support women in later life. This is especially important given that women are more likely than men to experience financial insecurity in later life,[42] and thus may face greater barriers to paid-for care services.

Finally, given the high death rates in some countries as a result of the COVID-19 pandemic, and the likely long-term health consequences of the virus that are becoming apparent, further scrutiny of future trends in health expectancies is needed.

Strengths and limitations

This systematic review provides a robust picture of trends in healthy and disability-free life expectancy in high-income countries. Although previous reviews have been published,[43]

For peer review only - http://bmjopen.bmj.com/site/about/guidelines.xhtml

a strength of our study is that we used reproducible systematic search techniques and reported quantitative comparisons of life and health expectancies to differentiate compression or expansion of morbidity. We chose to focus on evidence published from 2016, in order to avoid duplication of previous work and focus on contemporary evidence. This criterion has enabled a synthesis that prioritises the most recent evidence on trends in healthy and disability-free life expectancy. In addition to searching for studies, we searched the Office for National Statistics website, the UK government body responsible for analysing data about the UK population. We did not search the equivalent national statistical bodies for every other high income country as the volume of translation this would necessitate was unfeasible. However, this is not a major shortcoming: most of the non-UK studies used nationally representative samples (*see quality assessment in supplementary materials*), and none indicated unusual or contradictory trends that required verification from other data sources.

A key limitation of our synthesis is that whilst we were able to make some broad comparisons between studies, a more detailed comparison of trends was not possible. This was due to the high degree of heterogeneity between studies in: the age at which the health expectancy is estimated, trend periods (years and time frame), the measures used, and the way trends were stratified. This not only prevented a more meaningful comparison, but also limited the extent to which we were able explain differences in these trends between studies and countries.

Finally, the quality of this evidence was judged using criteria designed to assess threats to the validity of trends and the adequacy of the outcome measure.[12] For more than half the studies, it was not possible to arrive at a clear judgement of this due to the absence of required methodological detail. This is an important limitation, but one that is easily addressed if future studies of health expectancy trends report the methodological detail required. Where a judgement on quality was possible, studies were generally of fair or good quality, signalling no major concerns to the validity of the evidence synthesised. The rating of poor for a minority of studies largely reflected a change in the outcome measure over time,[31, 34, 36] which may reduce comparability between observed time points. To some extent, such changes are expected as panel and longitudinal studies evolve at each new point of data collection. Such reduced comparability may undermine the validity of the

For peer review only - http://bmjopen.bmj.com/site/about/guidelines.xhtml

trends reported in these studies, but this should be balanced against other aspects of the quality assessment (see supplementary materials). Overall, for studies where a clear judgment on quality was possible, we did not identify any studies that were particularly concerning in terms of quality and validity.

CONCLUSIONS

In a number of high-income countries, gains in healthy and disability-free life expectancy over time have not kept pace with the growth in life expectancy. That is, people are living longer but disability and poor health are occupying an increasing proportion of later life. A compression of disability for women in Sweden signals some progress in achieving healthier longer lives. These findings have implications for health and care related policy, and in particular ensuring people have timely access to care in later life. Further scrutiny of health expectancies in the wake of the COVID-19 pandemic is necessary given the high death rates in some countries and the long-term health consequences of the virus that are becoming apparent.

For peer review only

For peer review only - http://bmjopen.bmj.com/site/about/guidelines.xhtml

REFERENCES

1 Department of Social and Economic Affairs. World Population Ageing 2019 Highlights. *United Nations* 2019.

2 Organisation for Economic and Co-Operative Development. A Good Life in Old Age? Monitoring and Improving Quality in Long-Term Care. 2013.

3 Organisation for Economic Co-operative Development. Help Wanted? Providing and Paying for Long-Term Care. Organisation for Economic and Co-operative Development 2011.

4 Organisation for Economic Co-operative Development. Public long-term care financing arrangements in OECD countries. In: Oecd, ed. *Help Wanted?*: OECD 2011.

5 Pani-Harreman KE, Bours GJJW, Zander I*, et al.* Definitions, key themes and aspects of 'ageing in place': a scoping review. *Ageing and Society* 2020:1-34.

6 World Health Organisation. Decade of Healthy Ageing 2020-2030. 2019.

7 Jagger C, Matthews FE, Wohland P*, et al.* A comparison of health expectancies over two decades in England: results of the Cognitive Function and Ageing Study I and II. *Lancet* 2016;**387**:779-86.

8 Kingston A, Wohland P, Wittenberg R*, et al.* Is late-life dependency increasing or not? A comparison of the Cognitive Function and Ageing Studies (CFAS). *Lancet* 2017;**390**:1676-84.

9 Jagger C. Trends in life expectancy and healthy life expectancy. Newcastle University Institute for Ageing and Institute of Health & Society, Government Office for Science 2015.

10 Moher D, Liberati A, Tetzlaff J*, et al.* Preferred Reporting Items for Systematic Reviews and Meta-Analyses: The PRISMA Statement. *PLoS Medicine* 2009;**6**:e1000097.

11 Group WB. Fact Sheet: OECD High-Income. *Doing Business 2019*: World Bank 2019.

12 Freedman VA, Martin LG, Schoeni RF. Recent trends in disability and functioning among older adults in the United States: a systematic review. *Jama* 2002;**288**:3137-46.

13 Guzman-Castillo M, Ahmadi-Abhari S, Bandosz P*, et al.* Forecasted trends in disability and life expectancy in England and Wales up to 2025: a modelling study. *The Lancet Public Health* 2017;**2**:e307-e13.

14 Kingston A, Comas-Herrera A, Jagger C*, et al.* Forecasting the care needs of the older population in England over the next 20 years: estimates from the Population Ageing and Care Simulation (PACSim) modelling study. *Lancet Public Health* 2018;**3**:e447-e55.

15 Kingston A, Robinson L, Booth H*, et al.* Projections of multi-morbidity in the older population in England to 2035: Estimates from the Population Ageing and Care Simulation (PACSim) model. *Age and Ageing* 2018;**47**:374-80.

16 Office for National Statistics. Health state life expectancies, UK: 2015 to 2017. Office for National Statistics 2018.

17 Office for National Statistics. Health state life expectancies by national deprivation deciles, England and Wales: 2015 to 2017. Office for National Statistics 2019.

18 Reus-Pons M, Kibele EUB, Janssen F. Differences in healthy life expectancy between older migrants and non-migrants in three European countries over time. *International Journal of Public Health* 2017;**62**:531-40.

19 Global Burden of Disease study, Collaborators H. Global, regional, and national disability-adjusted life-years (DALYs) for 359 diseases and injuries and healthy life expectancy (HALE) for 195 countries and territories, 1990-2017: a systematic analysis for the Global Burden of Disease Study 2017. *Lancet* 2018;**392**:1859-922.

20 Hay SI, Abajobir AA, Abate KH*, et al.* Global, regional, and national disability-adjusted life-years (DALYs) for 333 diseases and injuries and healthy life expectancy (HALE) for 195 countries and territories, 1990-2016: A systematic analysis for the Global Burden of Disease Study 2016. *The Lancet* 2017;**390**:1260-344.

For peer review only - http://bmjopen.bmj.com/site/about/guidelines.xhtml

21 Kassebaum NJ, Arora M, Barber RM*, et al.* Global, regional, and national disability-adjusted life-years (DALYs) for 315 diseases and injuries and healthy life expectancy (HALE), 1990–2015: a systematic analysis for the Global Burden of Disease Study 2015. *The Lancet* 2016;**388**:1603-58.
22 Renard F, Devleesschauwer B, Van Oyen H*, et al.* Evolution of educational inequalities in life and health expectancies at 25 years in Belgium between 2001 and 2011: a census-based study. *Arch Public Health* 2019;**77**:6.
23 Yokota RTC, Nusselder WJ, Robine JM*, et al.* Contribution of chronic conditions to gender disparities in health expectancies in Belgium, 2001, 2004 and 2008. *Eur J Public Health* 2019;**29**:82-7.
24 Steensma C, Loukine L, Choi BC. Evaluating compression or expansion of morbidity in Canada: trends in life expectancy and health-adjusted life expectancy from 1994 to 2010. *Health Promot Chronic Dis Prev Can* 2017;**37**:68-76.
25 Bronnum-Hansen H, Eriksen ML, Andersen-Ranberg K*, et al.* Persistent social inequality in life expectancy and disability-free life expectancy: Outlook for a differential pension age in Denmark? *Scand J Public Health* 2017;**45**:459-62.
26 Grasset L, Peres K, Joly P*, et al.* Secular trends of mortality and dementia-free life expectancy over a 10-year period in France. *Eur J Epidemiol* 2019;**34**:115-23.
27 Sugawara YM, Saito Y. Changes in disability-free life expectancy (DFLE) at birth between 2000 and 2010 across Japanese prefectures. *International Journal of Public Health* 2016;**61**:739-49.
28 Tokudome S, Hashimoto S, Igata A. Life expectancy and healthy life expectancy of Japan: the fastest graying society in the world. *BMC research notes* 2016;**9**:482.
29 Deeg DJH, Comijs HC, Hoogendijk EO*, et al.* 23-Year Trends in Life Expectancy in Good and Poor Physical and Cognitive Health at Age 65 Years in the Netherlands, 1993-2016. *Am J Public Health* 2018;**108**:1652-8.
30 Gheorghe M, Wubulihasimu P, Peters F*, et al.* Health inequalities in the Netherlands: trends in quality-adjusted life expectancy (QALE) by educational level. *Eur J Public Health* 2016;**26**:794-9.
31 Storeng SH, Krokstad S, Westin S*, et al.* Decennial trends and inequalities in healthy life expectancy: The HUNT Study, Norway. *Scand J Public Health* 2018;**46**:124-31.
32 Jo MW, Seo W, Lim SY*, et al.* The Trends in Health Life Expectancy in Korea according to Age, Gender, Education Level, and Subregion: Using Quality-Adjusted Life Expectancy Method. *J Korean Med Sci* 2019;**34**:e88.
33 Lee JY, Ock M, Kim SH*, et al.* Health-Adjusted Life Expectancy (HALE) in Korea: 2005-2011. *J Korean Med Sci* 2016;**31 Suppl 2**:S139-S45.
34 Lagergren M, Johnell K, Schon P*, et al.* Towards a postponement of activities of daily living dependence and mobility limitations: Trends in healthy life years in old age in Sweden. *Scand J Public Health* 2017;**45**:520-7.
35 Sundberg L, Agahi N, Fritzell J*, et al.* Trends in health expectancies among the oldest old in Sweden, 1992-2011. *Eur J Public Health* 2016;**26**:1069-74.
36 Remund A, Cullati S, Sieber S*, et al.* Longer and healthier lives for all? Successes and failures of a universal consumer-driven healthcare system, Switzerland, 1990–2014. *International Journal of Public Health* 2019.
37 Cao B. Future healthy life expectancy among older adults in the US: a forecast based on cohort smoking and obesity history. *Popul Health Metr* 2016;**14**:23.
38 Crimmins EM, Zhang Y, Saito Y. Trends Over 4 Decades in Disability-Free Life Expectancy in the United States. *Am J Public Health* 2016;**106**:1287-93.
39 Freedman VA, Spillman BC. Active life expectancy in the older US population, 1982-2011 : differences between blacks and whites persisted. 2016.
40 Freedman VA, Wolf DA, Spillman BC. Disability-Free Life Expectancy Over 30 Years: A Growing Female Disadvantage in the US Population. *Am J Public Health* 2016;**106**:1079-85.
41 All Party Parliamentary Group for Longevity. The Health of the Nation: A Strategy for Healthier Longer Lives. All Party Parliamentary Group for Longevity, 2020.
42 Scharf T, Shaw C. Inequalities in later life. Centre for Ageing Better 2017.

For peer review only - http://bmjopen.bmj.com/site/about/guidelines.xhtml

43 J-M R, C J, Crimmins E.M ea. Trends in health expectancies. In: Jagger C CE, Saito Y, et al, ed. *International Handbook of Health Expectancies*. London: Springer 2020.

For peer review only

For peer review only - http://bmjopen.bmj.com/site/about/guidelines.xhtml

Table 1. Review criteria

**Population**	Studies must examine, trends from birth, 65 years and 85 years. Studies reporting trends from other ages were also reviewed where evidence was available. Studies must examine these trends in whole populations. Studies reporting trends in population sub-groups only (i.e. only those with heart failure) were ineligible.
**Exposure**	As this review reports evidence on life expectancy, active life expectancy, healthy life expectancy or disability-free life expectancy trends, an exposure variable was not required.
**Comparator**	Not applicable.
**Outcome(s)**	Active life expectancy, healthy life expectancy, disability-free life expectancy. Studies reporting *only* life expectancy trends were ineligible. Studies must examine changes in these outcomes over time (i.e. include more than one time point). Studies that report projections/forecasts of these outcomes were also eligible.
**Study design**	Studies must use an observational design and be carried out in an OECD high-income country. The review focused on evidence from the UK with comparison to evidence from other OECD high-income countries where possible. Studies published from 2016 were eligible. ONS reports were excluded if they were not the latest release, or reported trends for a period contained within a more recent ONS publication using the same data.

For peer review only

For peer review only - http://bmjopen.bmj.com/site/about/guidelines.xhtml

Table 2. Quality assessment criteria

Criteria	Parameters
**Study design**	Good: Independent repeat cross section<br>Fair: Panel design with aged in cohorts<br>Poor: Other
**Population coverage**	Good: National including institutionalised[a]<br>Fair: National but excluding those in institutions<br>*No criteria for poor*
**Comparability of interview methods between time points**	Good: Identical<br>Fair: Change in mode<br>Poor: Change in disability, functioning or health outcomes
**Quality of outcome measure**	Good: Detailed multiple item measure<br>Fair: Single item global measure<br>*No criteria for poor*
**Loss to follow up**	Good: NA or <5%<br>Fair: 5-10%<br>Poor: >10%<br>Note: This only applies to longitudinal study designs (i.e. not independent repeat cross sections)
**Proportion of proxy interviews**	Good: <5%<br>Fair: 10-20%<br>Poor: >20%
**Proportion of missing data**	Good: <5%<br>Fair: 5-10%<br>Poor: >10%

[a]Taken here to mean those resident in care homes, although 'institutionalised', where described in studies, was not always defined in terms of whether this related *only* to those resident in care homes

For peer review only

For peer review only - http://bmjopen.bmj.com/site/about/guidelines.xhtml

Table 3. Change in life expectancy, healthy life expectance and disability-free life expectancy across all studies where reported[a]

Study	Country	Age	Trend period	Change in LE	Change in HLE	Change in DFLE
All high-income countries combined from the Global Burden of Disease studies						
GBD study 2016	All high income	0	2005, 2015	Men: 1.75<br>Women: 1.33	Men: 1.43<br>Women: 1.08	-
GBD study 2017	All high income	0	1990, 2016	Men: 5.63<br>Women: 4.14	Men: 4.43<br>Women:3.21	-
		65		Men: 3.51<br>Women: 3.03	Men:2.27<br>Women: 2.6	-
GBD study 2018	All high income	0	1990, 2017	Men: 5.6<br>Women: 4.2	Men: 4.2<br>Women: 3.0	-
Studies with samples from Europe						
Jagger 2016	England	65	1991, 2011	Men: 4·5<br>Women: 3·6	Men: 3·8 (3·5 to 4·1)<br>Women: 3·1 (2·7 to 3·4)	Men: 2.6 (2.3, 2.9)<br>Women: 0.5 (0.2, 0.9)
Kingston 2017	England	65	1991, 2011	Men: 4.7<br>Women: 4.1		Men: 1.7 (1.2, 2.1)<br>Women: 0.2 (-0.4, 0.7)
Reus-Pons 2017	England & Wales	50	2001, 2011	Men: 2.8<br>Women: 2.2	Men: 0.25<br>Women: -0.15	-
ONS 2018	UK	0	2009/11 – 2015/17	Men: 0.8<br>Women: 0.4	Men: 0.4<br>Women: -0.2	-
Bronnum-Hansen 2017	Denmark	65	2006/07, 2010/11,2013/14	Difference in change in LE between high and low education:<br>Men: 0.1<br>Women: 0.3	-	Difference in change in DFLE between high and low education<br>Men: -0.3<br>Women: -0.3
Deeg 2018	Netherlands	65	1993, 2016	Men: 4.0<br>Women: 2.2	Physical<br>Men: -2.2<br>Women: -1.5<br>Cognitive<br>Men: 4.7<br>Women: 4.6	-
Gheorghe 2016	Netherlands	25	2001, 2011	Men:<br>High: 2.88<br>Med: 3.22<br>Low: 2.43<br><br>Women:<br>High: 1.78<br>Med: 1.53<br>Low: 1.15	Men:<br>High 2.85<br>Med: 3.01<br>Low: 2.13<br><br>Women:<br>High: 2.64<br>Med: 1.57<br>Low: 1.80	-
		65		Men:<br>High: 2.48	Men:<br>High: 2.17	-

For peer review only - http://bmjopen.bmj.com/site/about/guidelines.xhtml

				Med: 2.24<br>Low: 1.68<br><br>Women:<br>High: 1.64<br>Med: 1.41<br>Low: 1.12	Med: 1.91<br>Low: 1.37<br><br>Women:<br>High: 1.74<br>Med: 1.21<br>Low: 1.14	
Reus-Pons 2017	Netherlands	50	2001, 2011	Men: 2.82<br>Women: 1.84	Men: 2.21<br>Women: 1.25	-
Grasset 2019	France	70	1988/1989, 1999/2000	3.0	-	2.7
		75		2.4	-	2.2
		80		1.8	-	1.5
		85		1.3	-	1.1
		90		0.8	-	0.7
Remund 2019	Switzerland	30	1990, 2014	Men: 5.02<br>Women: 3.09	Men: 4.52<br>Women: 3.09	-
Storeng 2018	Norway	50	1984/86, 1995/97, 2006/08	Men: 6.99 (5.27–8.72)<br>Women: 6.75 (5.16–8.34)	Men: 6.90 (6.08–7.73)<br>Women: 5.40 (4.56–6.25)	Men: 2.71 (2.01–3.42)<br>Women: 0.33 (–0.40–1.06)
Sundburg 2016	Sweden (SWEOLD)	77	1992, 2002, 2004, 2011	Men: 1.7<br>Women: 1.1	-	Men: 1.1<br>Women: 1.6
	Sweden (SHARE)			Men: 0.6<br>Women: 0.4	-	Men: 0.1<br>Women: 1.3
Yokota 2019	Belgium	15	2001, 2004, 2008	Men: 1.6<br>Women: 1.0	-	Men: 0.7<br>Women: -0.7
Studies with samples from Asia						
Jo 2019	R. Korea	0	2005, 2013	Men: 3.38<br>Women: 3.15	Men: 4.03<br>Women: 3.44	-
Lee 2016	R. Korea	0	2005, 2008, 2011	Men: 2.5<br>Women: 2.6	Men: 1.4[b]<br>Women: 1.2[b]	-
		65		Men: 1.6<br>Women: 2.0	Men: 2.2[b]<br>Women: 2.6[b]	-
		85		Men: 0.3<br>Women: 0.7	Men: 1.4[b]<br>Women: 1.4[b]	-
Sugawara 2016	Japan	0	2000, 2010	Men: 1.9<br>Women:1.7	-	Men: 1.0<br>Women: 0.4
Tokudome 2016	Japan	0	1990, 2013	Men: 4.01<br>Women: 4.43	Men: 3.02<br>Women: 3.32	-
Studies with sample from North America						
Crimmins 2016	US	0	1970, 1980, 1990, 2000, 2010	Men: 9.2<br>Women: 6.4	-	Men: 4.5<br>Women: 2.7
		20-64		Men: 1.8<br>Women: 0.9	-	Men: 0.9<br>Women: -0.6
		65		Men: 4.7<br>Women: 3.5	-	Men: 2.7<br>Women: 2.4
		85		Men: 1.1<br>Women: 1.3	-	Men: 0.5<br>Women: 0.8

[a]Table and comparison does not include studies where: the metric of change was not comparable for each health expectancy (Steensma et al. 2017); trends were reported as slope of index inequality only (ONS 2019); only forecasts are reported (Kingston et al., 2018,a,b, Guzman-Castillo et al., 2017, Cao et al., 2016); total life expectancy is reported only as a graph (Lagergren et al., 2017, Freedman 2016a,b); trends are reported as difference in change in DFLE between levels of education and not comparable to the LE trend (Renard 2015).

For peer review only

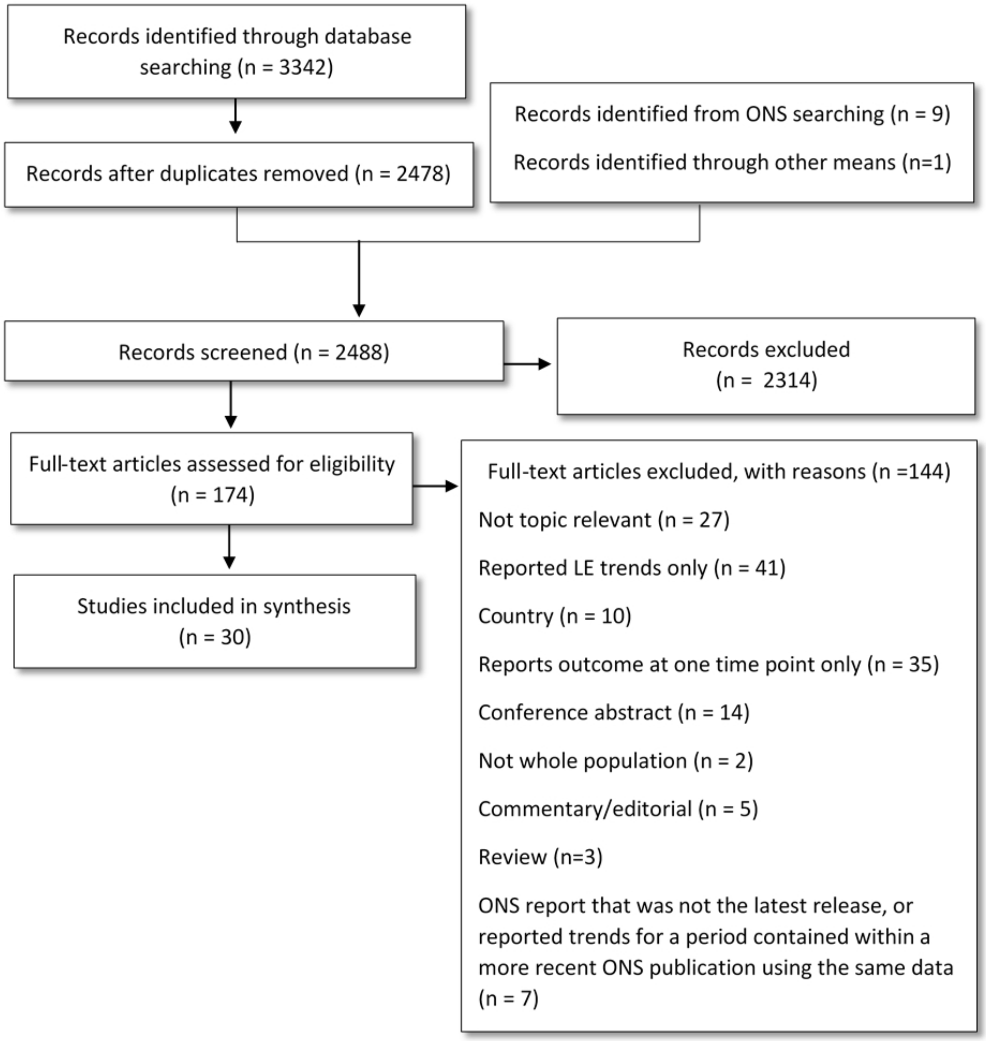

Figure 1. PRISMA Flowchart

Supplementary Materials

Table 1. Summary of included studies

Study	Country	Data source	Trend years	Trends reported	Disability/health measured from a global (single) item or multiple items?[a]	Stratification	At age	Reports evidence about factors associated with trends?	Quality rating
High income countries combined									
Global Burden of Disease study 2016[b]	High income countries combined	Global Burden of Disease Study	2005, 2015	LE, HLE	Multiple	Sex	0	No[c]	UNCLEAR
Global Burden of Disease study 2017[b]	High income countries combined	Global Burden of Disease Study	1990, 2016	LE, HLE	Multiple	Sex	0, 65	No[c]	UNCLEAR
Global Burden of Disease study 2018[b]	High income countries combined	Global Burden of Disease Study	1990, 2017	LE, HLE	Multiple	Sex	0	No[c]	UNCLEAR
Studies with samples from OECD high-income countries in Europe									
Jagger 2016	England	CFAS I and II	1991, 2011	LE, HLE, DFLE, LE without cognitive impairment, % of LE without disability, % of LE without cognitive impairment, % of life spent healthy, disability prevalence	HLE: global CI free LE: multiple DFLE: multiple	Sex	65	No	GOOD (disability outcome) FAIR (health outcome)
Kingston 2017	England	CFAS I and II	1991, 2011, and 2015, 2025, 2035 (projection)	LE, DFLE, % of LE without disability, disability prevalence	Multiple	Sex	65	No	GOOD

For peer review only

Kingston 2018a	England	CFAS II, ELSA, Understanding Society	2015, 2025, 2035 (projection)	LE, DFLE, % of LE without disability, disability prevalence	Multiple	Sex, age	65-74, 75-84, 85	No	GOOD
Kingston 2018b	England	CFAS II, ELSA	2015, 2025, 2035 (projection)	LE, LE with 2+ diseases, disability prevalence	Multiple	Sex, age	65-74, 75-84, 85	No	GOOD
Guzman-Castillo 2017	England, Wales	ELSA	2015, 2025 (projection)	LE, DFLE, disability prevalence	Multiple	None	65	No	FAIR
Reus-Pons 2017	England & Wales	Census, national death registries, Permanent Survey on the Living Situation (POLS), Health Survey	2001, 2011	LE, HLE	Global	Sex, migrant/non-migrant	50	No	UNCLEAR
ONS 2019	England & Wales	UK Census, Annual Population Survey, Indices of Multiple Deprivation 2015, Welsh Index of Multiple Deprivation 2014	2012/14 – 2015/17	LE, HLE	Global	Sex, deprivation decile[d]	0, 65	No	FAIR
ONS 2018	UK, England, Wales, Northern Ireland, Scotland	UK Census, Annual Population Survey	2009/11 – 2015/17	LE, HLE	Global	Sex	0	No	FAIR
Bronnum-Hansen 2017a	Denmark	Danish Surveys of Health, Ageing and Retirement in Europe	2006/07, 2010/11,2013/14	LE, DFLE,	Global	Sex, education	65	No	FAIR
Deeg 2018	Netherlands	Longitudinal Aging Study Amsterdam	1993, 2016	LE, HLE, % of life spent healthy	Multiple	Sex	65	No	UNCLEAR
Gheorghe 2016	Netherlands	Dutch Labour force Survey and National Mortality Registry	2001, 2011	LE, HLE (as quality adjusted life expectancy),	Multiple	Sex, education	25, 65	No	UNCLEAR

For peer review only

Grasset 2019	France	PAQUID and Three City cohort studies	1988/1989, 1999/2000	LE, DFLE (as dementia-free life expectancy),	Multiple	Sex	65	No	UNCLEAR
Lagergren 2017	Sweden	National mortality statistics, Nationwide Swedish Surveys of Living Conditions	1980/1985, 1994/95, 2006/2011	LE (graph only), DFLE, disability prevalence	Multiple	Sex	65	No	POOR
Remund 2019	Switzerland	Swiss National Cohort, Swiss Health Interview Survey	1990, 2014	LE, HLE,	Global	Sex, education	30	No	POOR
Renard 2019	Belgium	Census, National Registry	2001, 2011	LE, DFLE,	Global	Sex, education	25	No	FAIR
Reus-Pons 2017	Netherlands	Census, national death registries, Permanent Survey on the Living Situation (POLS) and Health Survey	2001, 2011	LE, HLE,	Global	Sex	50	No	UNCLEAR
Storeng 2018	Norway	HUNT Study, Norwegian Education Database	1984/86, 1995/97, 2006/08	LE, HLE, DFLE,	HLE & DFLE: Global	Sex, education	30	No	POOR
Sundburg 2016	Sweden	National mortality statistics, Swedish Panel Study of Living Conditions of the Oldest Old (SWEOLD), Survey of Health, Ageing and Retirement in Europe (SHARE)	1992, 2002, 2004, 2011	LE, DFLE,	Multiple	Sex	77	No	UNCLEAR
Yokota 2019	Belgium	Health Interview Survey, National Statistics	2001, 2004, 2008	LE, DFLE,	Multiple	Sex	15	No	GOOD
Studies with samples from OECD high-income countries in Asia									
Jo 2019	R. Korea	Korean Statistical Information Service,	2005, 2013	LE, HLE,	Multiple	Sex	0	No	UNCLEAR

For peer review only

		Korea National Health and Nutrition Examination Survey							
Lee 2016	R. Korea	Korean Statistical Information Service, Korea National Health and Nutrition Examination Survey	2005, 2008, 2011	LE, HLE,	Multiple	Sex	0-100 at five year intervals; ages 0, 65 and 85 reported here	No	UNCLEAR
Sugawara 2016	Japan	Census, Comprehensive Survey of Living Conditions of the People on Health and Welfare	2000, 2010	LE, DFLE, % life without disability	Multiple	None	0	No	UNCLEAR
Tokudome 2016	Japan	Global Burden of Disease Study 2013	1990, 2013	LE, HLE,	Multiple	Sex	0	No	UNCLEAR
Studies with samples from OECD high-income countries in North America									
Cao 2016	US	Human Mortality Database, US National Health Interview Survey (NHIS)	1982, 2010, 2040 (projection)	LE, DFLE,	Global	Sex	55-85	Yes	FAIR
Crimmins 2016	US	Census, US National Vital Statistics, National Health Interview	1970, 1980, 1990, 2000, 2010	LE, DFLE, % life without disability, disability prevalence	Global	Sex	0, 20-64, 65, 85	No	FAIR
Freedman 2016a	US	National Long-Term Care Survey, National Health and Aging Trends Study	1982, 2004, 2011	LE (graph only), DFLE, prevalence	Multiple	Sex	65, 85	No	UNCLEAR
Freedman 2016b	US	National Long-Term Care Survey, National Health and Aging Trends Study	1982, 2004, 2011	LE (graph only), Active Life Expectancy	Multiple	Sex, Race	65, 85	No	UNCLEAR

For peer review only

Steensma 2017	Canada	Statistics Canada, National Population Health Survey, Canadian Community Health Survey	1994, 2010	LE, HLE	Multiple	Sex	0, 20, 65	No	UNCLEAR

[a]Global item measures include self-rated health, the Global Limitation Activity Indicator, or other single item measures about having a limitation; [b]Disability adjusted life years reported as global estimates (i.e. not country specific) and were thus not included here; [c]Disease contributions were not reported for individual countries; [d]Healthy life expectancy trends are not reported by deprivation deciles, but as a slope index of inequality between the most and least deprived deciles

For peer review only

Table 2. Quality assessment

Study	Study design	Population coverage	Comparability of interview methods	Outcome measure	Loss to follow up %	Proxy %	Missing data %	Other publications consulted	SUMMARY JUDGMENT
Bronnum-Hansen 2017	FAIR<br>Danish Surveys of Health, Ageing and Retirement in Europe	FAIR<br>National samples but inclusion of those in institutions varies according to different countries within the study.	FAIR<br>Change in items across waves, but disability question did not change across waves 1-7.	Disability: FAIR<br>(Single item question - Global Activity Limitation Indicator)	UNCLEAR<br>Not reported in publication and unable to find loss to follow up data on SHARE methods documentation	UNCLEAR<br>Not reported	UNCLEAR<br>Not reported.	SHARE methods report: http://www.share-project.org/fileadmin/pdf_documentation/Methodology/Methodology_2005.pdf	FAIR
Cao 2016	GOOD<br>US National Health Interview Survey	FAIR	FAIR<br>Not reported, but in Freedman et al. (2002), the NHIS (which is the survey used in Cao et al.), they note the sampling frame was redesigned in 1995. A change in disability items is reported, but this study used a different set of items to ensure disability is comparable across waves.	Disability: FAIR<br>Although this measure draws upon multiple ADLs, the question is whether the participant has any limitation in any of the ADLs, and so is a single item measure.	GOOD: NA	UNCLEAR<br>Not reported.	UNCLEAR<br>Not reported.	Freedman et al. (2002)	FAIR
Crimmins 2016	GOOD<br>US National Health Interview Survey	FAIR	FAIR<br>Not reported, but in Freedman et al. (2002), the NHIS (which is the survey used in Crimmins et al), they note the sampling frame was	Disability: FAIR<br>Although question draws upon multiple ADLs, the question is whether the participant has any limitation in any of the ADLs, and so	GOOD: NA	UNCLEAR<br>Not reported.	UNCLEAR<br>Not reported.	Freedman et al. (2002)	FAIR

			redesigned in 1995. A change in disability items is reported, but study uses different set of items to ensure disability is comparable across waves.	is a single item measure.					
Deeg 2018	FAIR Longitudinal Ageing Study Amsterdam	GOOD	UNCLEAR The methods paper for survey used in this study states that measures have changed, including, among others, cognitive tests, Parkinson' diagnosis, malnutrition, and use of help. However, it is not clear if any of these (such as the cognitive tests) were used as part of the outcome measures in this study.	Disability: GOOD Cognitive: GOOD	UNCLEAR Study reports only average non-mortality attrition: 4.0% for participants in good cognitive health and 7.1% for those in poor cognitive health. No detail on attrition on whole sample (including attrition from death) from wave to wave.	GOOD From publication appendix: "Across the study period, the average use of proxies was 2% for respondents in good physical health, 3% for those in fair physical health, and 8% for those in poor physical health."	UNCLEAR Not reported.	https://www.ncbi.nlm.nih.gov/pmc/articles/PMC5010587/	UNCLEAR However, both outcomes (disability and cognition) used multi-item measures.
Freedman 2016a	GOOD National Long Term Care Survey and the National Health and Aging Trends Study	GOOD	UNCLEAR Not reported.	Disability: GOOD	UNCLEAR Not reported.	UNCLEAR Not reported.	UNCLEAR Not reported.		UNCLEAR However, the outcome was a multi-item measure.
Freedman 2016b	GOOD National Long Term Care Survey and the National Health and Aging Trends Study	GOOD	FAIR	Disability: GOOD	UNCLEAR Not reported.	UNCLEAR Not reported.	UNCLEAR Not reported.		UNCLEAR However, the outcome was a multi-item measure.

GBD 2016 DALYs and HALE Collaborators	GOOD UK estimates based on UK census population data	UNCLEAR Not reported.	UNCLEAR Not reported.	UNCLEAR Not reported.	UNCLEAR Not reported.	UNCLEAR Not reported.	UNCLEAR Not reported.		UNCLEAR
GBD 2017 DALYs and HALE Collaborators	GOOD UK estimates based on UK census population data	UNCLEAR Not reported.	UNCLEAR Not reported.	UNCLEAR Not reported.	UNCLEAR Not reported.	UNCLEAR Not reported.	UNCLEAR Not reported.		UNCLEAR
GBD 2018 DALYs and HALE Collaborators	GOOD UK estimates based on UK census population data	UNCLEAR Not reported.	UNCLEAR Not reported.	UNCLEAR Not reported.	UNCLEAR Not reported.	UNCLEAR Not reported.	UNCLEAR Not reported.		UNCLEAR
Gheorghe 2016	FAIR Dutch Labour force Survey	FAIR Does not include those in an institution.	UNCLEAR Not reported.	Health: GOOD	UNCLEAR Not reported.	UNCLEAR Not reported.	UNCLEAR Missing data % not reported and used complete case analysis.		UNCLEAR However, the outcome was a multi-item measure.
Grasset 2019	FAIR PAQUID and Three City Cohorts	FAIR Initial cohort were those living at home, follow ups included those moving into a care home.	UNCLEAR Not reported.	Dementia Free LE: GOOD	POOR Between baseline and final follow up: 65% (PAQUID) and 44% (3C data). (Calculated by us based on follow up data in supplementary materials).	UNCLEAR Not reported.	UNCLEAR Not reported.	See supplementary material for loss to follow up	UNCLEAR However, the outcome was a multi-item measure.
Guzman-Castillo 2017	FAIR English Longitudinal Study of Ageing	FAIR	NA as forecasting from baseline data	Disability: GOOD	UNCLEAR Not reported.	UNCLEAR Not reported.	UNCLEAR Not reported.		FAIR However, the outcome was a multi-item measure.
Jagger 2016	GOOD CFAS I and II	GOOD Not national but includes multiple	GOOD	SRH: FAIR Disability: GOOD	GOOD: NA	FAIR 20%	GOOD SRH: 2·9% (1991) and 4·2% (2011)	https://www.sciencedirect.com/science	GOOD: for disability and cognition

For peer review only - http://bmjopen.bmj.com/site/about/guidelines.xhtml

		sites and includes those in care homes.		Cognitive health: GOOD			Cognitive: 1·8% (1991) and 3·7% (2011)<br>Disability: 1·1% (1991) and 4·2% (2011)	/article/pii/S0140673613615706	outcome, but FAIR for self-rated health outcome.
Jo 2019	GOOD<br>Korea National Health and Nutrition Examination Survey	FAIR	UNCLEAR<br>Methods paper states that the surveys changed but does not detail what these changes include, such as whether these changes include the disability questions and measures	QALE: GOOD	GOOD: NA	UNCLEAR<br>Not reported.	UNCLEAR<br>Not reported.	https://www.ncbi.nlm.nih.gov/pmc/articles/PMC3937975/	UNCLEAR<br>However, the outcome was a multi-item measure.
Kingston 2017	GOOD<br>CFAS I and II	GOOD<br>Not national but includes multiple sites and includes those in care homes.	GOOD	Disability: GOOD	GOOD: NA	FAIR<br>20%	GOOD<br>Not reported but assumed to be same as Jagger et al. as uses same data.	https://www.sciencedirect.com/science/article/pii/S0140673613615706	GOOD
Kingston 2018a	GOOD<br>English Longitudinal Study of Aging Wave 5 only, CFAS II, Understanding Society	GOOD	NA as forecasting from baseline data	Disability: GOOD	GOOD: NA	UNCLEAR/FAIR depending on dataset<br><br>CFAS: Not reported, but assumed to be as reported elsewhere for CFAS (FAIR). No information on % proxy interviews for ELSA (UNCLEAR).	UNCLEAR<br>Not reported, but missing values imputed.		GOOD
Kingston 2018b	GOOD<br>English Longitudinal Study of Aging Wave 5 only, CFAS II, Understanding Society	GOOD	NA as forecasting from baseline data	Disability: GOOD<br>Multi-morbidity: GOOD	GOOD: NA	UNCLEAR/FAIR depending on dataset<br><br>CFAS: Not reported, but assumed to be	UNCLEAR<br>Not reported, but missing values imputed.		GOOD

For peer review only - http://bmjopen.bmj.com/site/about/guidelines.xhtml

						as reported elsewhere for CFAS (FAIR). No information on % proxy interviews for ELSA (UNCLEAR).			
Lagergren 2017	GOOD<br>Swedish Survey of Living Conditions	GOOD	POOR<br>"In the years 1996–2000, a filter was introduced into the questionnaire to the effect that only people who were dependent for all instrumental ADL (IADL) were asked the questions about ADL." (p.552)<br><br>"From 1980 to 2001 there was an upper age limit of 84 years, with the exception of 1988/89." (p.56)	Disability: GOOD	GOOD: NA	FAIR<br>From methods paper on dataset: "The proportion of proxy interviews varies from 2–3% among those aged 65–79 years to 14–16% among individuals aged 80 years and older." (p.56)	UNCLEAR<br>Not reported.	https://journals.sagepub.com/doi/full/10.1177/1403494815605195	POOR<br>However, the outcome was a multi-item measure.
Lee 2016	GOOD<br>Korea National Nutrition and Health Survey	UNCLEAR	UNCLEAR<br>Not reported, but uses the same data as Jo et al. 2019, which report changes in survey, although such changes are not detailed.	GOOD<br>Health adjusted life expectancy was calculated using disability weights attached to self-reported morbidity for 45 diseases. This was converted into a score of disability measured on a scale from 0 (full health) to 1 (being dead). This would suggest it	GOOD: NA	UNCLEAR<br>Not reported.	UNCLEAR<br>Not reported		UNCLEAR

For peer review only - http://bmjopen.bmj.com/site/about/guidelines.xhtml

				is a multiple item measure.					
ONS 2018	GOOD<br>UK Census and Annual Population Survey	FAIR<br>Excludes those in communal establishments	GOOD<br>No change in health question/measure, but items used to measure disability changed from the 2013 APS survey (p.4). However, the ONS analysis does not report trends in DFLE (HLE and LE only).	Health: FAIR	GOOD: NA	UNCLEAR<br>Not reported.	UNCLEAR<br>Not reported.	https://www.ons.gov.uk/peoplepopulationandcommunity/healthandsocialcare/healthandlifeexpectancies/methodologies/healthstatelifeexpectanciesukqmi#_blank	FAIR
ONS 2019	GOOD<br>UK Census and Annual Population Survey	FAIR<br>Excludes those in communal establishments	GOOD<br>No change in health question/measure, but items used to measure disability changed from the 2013 APS survey (p.4). However, the ONS analysis does not report trends in DFLE (HLE and LE only).	Health: FAIR	GOOD: NA	UNCLEAR<br>Not reported.	UNCLEAR<br>Not reported.		FAIR
Remund 2019	GOOD<br>Swiss Health Interview Survey	UNCLEAR<br>National, but unclear whether it includes those in institutions.	POOR<br>The phrasing of the SRH question and response items were not identical in each survey wave.	SRH: FAIR	GOOD: NA	UNCLEAR<br>Not reported.	UNCLEAR<br>Not reported.		POOR
Renard 2019	GOOD<br>Belgium Health Interview Survey	GOOD	GOOD<br>Methods paper states no major methodological changes have been implemented between waves,	Disability: FAIR (Single item question - Global Activity Limitation Indicator)	GOOD: NA	UNCLEAR<br>Not reported.	UNCLEAR<br>Not reported.	https://archpublichealth.biomedcentral.com/articles/10.1186/0778-7367-71-24	FAIR

For peer review only

			except oversampling from 1997.						
Reus-Pons 2017	GOOD<br>Permanent Survey on the Living Situation (POLS) and Health Survey (Netherlands) and Census (England & Wales)	UNCLEAR<br>Not reported.	UNCLEAR<br>Not reported.	SRH: FAIR	GOOD: NA	UNCLEAR<br>Not reported.	POOR/UNCLEAR<br>For Belgium sample: data on self-rated health were missing for 'around' 5% of the non-migrant population and 'around' 10% of the migrant population. Exact % not reported, thus could be >10%.<br>Not reported for England and Wales sample.		UNCLEAR
Steensma 2017	GOOD<br>National Population Health Survey and the Canadian Community Health Survey	FAIR	UNCLEAR<br>Different surveys used between 1994-1999 and 2000-2010, although it is not clear if this reflected any change in questions and methods used.	Health: GOOD (multiple item health utilities index)	GOOD: NA	UNCLEAR<br>Not reported.	UNCLEAR<br>Not reported.		UNCLEAR<br>However, outcome was a multi-item measure
Storeng 2018	GOOD<br>HUNT Study	POOR<br>Data are sampled from one county only (i.e. not national, with 18 other counties). Not clear if it includes those in institution or not.	POOR<br>Self-rated health was measured the same at all three HUNT studies. The question for longstanding limiting illness differed between HUNT1 and HUNT 2 and 3 (that is, HUNT 2 and 3 asked about limiting illness	Health: FAIR<br>Disability: FAIR	GOOD: NA	UNCLEAR<br>Not reported.	GOOD<br>Missing health data < 5%	https://academic.oup.com/ije/article/42/4/968/655743#11331793	POOR

For peer review only

			lasting for at least one year, whereas HUNT 1 did not).						
Sugawara 2016	GOOD Comprehensive Survey of Living Conditions of the People on Health and Welfare	FAIR	UNCLEAR Not reported.	UNCLEAR/POOR Disability was measured using the following two survey questions: "Are you currently institutionalized in hospitals, clinics, or long-term care facilities?" and "Do you have any limitations in carrying out normal activities due to health problems?" Those who answered "yes" to **either** question are considered to have disability. However, the authors also state that those institutionalised are excluded, so the first question would, in theory, result in a No. There is no clarification in the paper on this inconsistency. It is possible, therefore, that only the second question (single item) was measuring disability. Without further detail, it is	GOOD: NA	UNCLEAR Not reported.	UNCLEAR Not reported.		UNCLEAR

For peer review only

				not possible to clarify this.					
Sundburg 2016	FAIR<br>Swedish Panel Study of Living Conditions of the Oldest Old and the Survey of Health, Ageing and Retirement in Europe	GOOD	UNCLEAR<br>Not reported.	Disability: GOOD	UNCLEAR<br>Not reported.	UNCLEAR/POOR depending on dataset<br><br>For SWEOLD: in another paper describing this dataset, it is reported that 13.3 were proxy interviews, and another 7.3% of participants needed proxy help answering questions (POOR).<br><br>For SHARE, there is no information on this (UNCLEAR).	UNCLEAR<br>Not reported.	https://link.springer.com/article/10.1007%2Fs10433-013-0275-7 and https://academic.oup.com/ije/article/43/3/731/2949546#57616062	UNCLEAR
Tokudome 2016	GOOD<br>Global Burden of Disease 2013 study estimates for Japan, which are taken from national registries and population statistics	UNCLEAR<br>Not reported.	UNCLEAR<br>Not reported.	UNCLEAR<br>Not reported.	UNCLEAR<br>Not reported.	UNCLEAR<br>Not reported.	UNCLEAR<br>Not reported.		UNCLEAR
Yokota 2019	GOOD<br>Belgium Health Interview Survey	GOOD	GOOD<br>Methods paper states no major methodological changes have been implemented between waves, except oversampling from 1997.	Disability: GOOD	GOOD: NA	FAIR<br>Time 1: 4.3%, time 2: 10.2%, time 3: 13%	UNCLEAR<br>Missing data % not reported, and used complete data only (for disability and chronic conditions)		GOOD

For peer review only

Table 3a. Summary of life expectancy trends data for studies from the US and Canada (years)[a]

Study	At age	Time points	T1		T2		T3		T4		T5		T6		T7		Change between first and last time point
			M	F	M	F	M	F	M	F	M	F	M	F	M	F	
Cao 2016	55-85	T1: 1982<br>T2: 1990<br>T3: 2000<br>T4: 2010<br>T5: 2020<br>T6: 2030<br>T7: 2040	19.96	23.40	20.70	23.64	21.75	23.92	22.82	24.69	23.83 (23.5 - 24.05)	25.11 (24.9 - 25.24)	24.63 (24.2 - 24.98)	25.49 (25.2 - 25.69)	25.24 (24.7 - 25.67)	25.74 (25.4 - 26.00)	Men: 5.28[b]<br>Women: 2.34[b]
Crimmins 2016	0	T1: 1970<br>T2: 1980<br>T3: 1990<br>T4: 2000<br>T5: 2010	67.0	74.6	70.1	77.6	71.8	78.8	74.1	79.5	76.2	81.0					Men: 9.2<br>Women: 6.4
	20-64		40.8	42.8	41.6	43.3	41.8	43.5	42.4	43.6	42.6	43.7					Men: 1.8<br>Women: 0.9
	65		13.0	16.8	14.2	18.4	15.1	19.0	16.1	19.1	17.7	20.3					Men: 4.7<br>Women: 3.5
	85		4.7	5.6	5.1	6.4	5.3	6.7	5.5	6.6	5.8	6.9					Men: 1.1<br>Women: 1.3
Steensma 2017	0	T1: 1994/95<br>T2: 96/97<br>T3: 98/99<br>T4: 00/01<br>T5: 03<br>T6: 05<br>T7: 09/10	75.2	81.3	75.7	81.5	76.2	81.8	77.2	82.5	77.7	82.8	78.3	83.2	79.6	84.1	Men: 4.4[b]<br>Women: 2.8[b]
	20		55.7	61.7	56.2	61.8	56.7	62.1	57.7	62.8	58.1	63.0	58.7	63.4	59.9	64.4	Men: 4.2[b]<br>Women: 2.7[b]
	65		15.8	19.9	15.9	19.9	16.2	20.2	17.0	20.7	17.4	20.9	19.9	21.3	18.9	22.1	Men: 3.1[b]<br>Women: 2.2[b]

[a]95% confidence intervals in brackets where reported; [b]Change estimate not reported, and thus calculated by us (T^2-T^1)

For peer review only

Table 3b. Summary of healthy life expectancy trends data for studies from the US and Canada (years)[a]

Study	At age	Time points	T1		T2		T3		T4		T5		T6		T7		Average annual change (%)
			M	F	M	F	M	F	M	F	M	F	M	F	M	F	
Steensma 2017	0	T1: 1994/95 T2: 96/97 T3: 98/99 T4: 00/01 T5: 03 T6: 05 T7: 09/10	66.4 (66.0 - 66.8)	70.0 (69.5 - 70.4)	69.0 (68.7 - 69.2)	72.9 (72.5 - 73.2)	68.9 (68.6 - 69.3)	72.1 (71.7 - 72.5)	68.5 (68.3 - 68.7)	71.5 (71.3 - 71.7)	69.5 (69.1 - 69.8)	71.7 (71.3 - 72.1)	69.3 (69.0 - 69.7)	72.3 (71.9 - 72.6)	70.7 (70.5 - 70.9)	73.3 (73.1 - 73.5)	Men: 0.3 Women: 0.2
	20		48.0 (47.6 - 48.3)	51.4 (51.0 - 51.9)	50.1 (49.8 - 50.3)	54.0 (53.7 - 54.3)	50.1 (49.7 - 50.4)	53.1 (52.7 - 53.5)	49.8 (49.6 - 50.0)	52.7 (52.5 - 52.9)	50.9 (50.5 - 51.2)	53.1 (52.7 - 53.5)	50.8 (50.4 - 51.2)	55.3 (53.1 - 53.9)	50.2 (51.8 - 52.2)	54.5 (54.3 - 54.8)	Men: 0.4 Women: 0.2
	65		12.3 (12.0 - 12.6)	14.8 (14.4 - 15.1)	12.8 (12.6 - 13.1)	15.6 (15.3 - 15.9)	12.8 (12.5 - 13.1)	15.3 (15.0 - 15.7)	13.1 (13.0 - 13.3)	15.4 (15.2 - 15.6)	14.0 (13.7 - 14.3)	15.7 (15.4 - 16.1)	14.2 (13.9 - 14.5)	16.0 (15.6 - 16.3)	14.9 (14.7 - 15.1)	16.8 (16.7 - 17.0)	Men: 1.2 Women: 0.7

[a]95% confidence intervals in brackets where reported;

For peer review only

Table 3c. Summary of disability-free life expectancy trends data for studies from the US and Canada[a]

Study	Trend	At age	Time points	T1		T2		T3		T4		T5		Change between first and last time point
				Male	Female	Male	Female	Male	Female	Male	Female	Male	Female	
Crimmins (2016)	DFLE (years)	0	T1: 1970<br>T2: 1980<br>T3: 1990<br>T4: 2000<br>T5: 2010	56.5 (56.4 - 56.6)	62.7 (62.6 - 62.8)	56.5 (56.4 - 56.6)	62.8 (62.6 - 63.0)	58.8 (58.6 - 58.9)	63.9 (63.8 - 64.0)	60.0 (59.9 - 60.2)	64.6 (64.4 - 64.7)	61.0 (60.9 - 61.2)	65.4 (65.3 - 65.6)	Men: 4.5<br>Women: 2.7
		20-64		34.9 (34.8 - 35.0)	37.4 (37.3 - 37.5)	34.7 (34.6 - 34.8)	36.7 (36.6 - 36.8)	35.3 (35.2 - 35.4)	37.0 (36.9 - 37.1)	35.6 (35.5 - 35.7)	36.9 (36.8 - 37.0)	35.8 (35.7 - 35.9)	36.8 (36.7 - 37.0)	Men: 0.9<br>Women: -0.6
		65		6.6 (6.6 - 6.8)	9.1 (9.0 - 9.2)	6.8 (6.7 - 6.9)	9.3 (9.2 - 9.4)	7.4 (7.3 - 7.5)	9.9 (9.8 - 10.0)	8.2 (8.0 - 8.3)	10.5 (10.3 - 10.6)	9.3 (9.1 - 9.4)	11.5 (11.4 - 11.6)	Men: 2.7<br>Women: 2.4
		85		1.4 (1.3 - 1.6)	1.4 (1.3 - 1.6)	1.5 (1.3 - 1.7)	1.7 (1.5 - 1.8)	1.6 (1.5 - 1.8)	1.7 (1.6 - 1.8)	1.8 (1.6 - 1.9)	1.9 (1.8 - 2.0)	1.9 (1.8 - 2.1)	2.2 (2.1 - 2.4)	Men: 0.5<br>Women: 0.8
	% of life spent disability-free	0		84.2	84.0	81.6	80.9	81.8	81.1	81.0	81.3	80.1	80.7	Men: -4.1<br>Women: -3.3
		20-64		85.5	87.4	83.4	84.8	84.4	85.0	84.0	84.6	84.0	84.2	Men: -1.5<br>Women: -3.2
		65		51.2	54.2	47.8	50.5	49.1	52	50.9	55	52.5	56.7	Men: 1.3<br>Women: 2.5
		85		30.4	25.2	29	26	30.8	25.5	32.7	28.8	32.8	31.9	Men: 2.4<br>Women: 6.7
	LE for those in an institution	0		0.6 (0.6 - 0.6)	1.1 (1.0 - 1.2)	0.6 (0.6 - 0.7)	1.4 (1.3 - 1.5)	0.7 (0.6 - 0.7)	1.5 (1.4 - 1.5)	0.5 (0.5 - 0.6)	1.1 (1.0 - 1.2)	0.4 (0.4 - 0.5)	0.8 (0.8 - 0.9)	Men: -0.2<br>Women: -0.3
		20-64		0.3 (0.2 - 0.3)	0.2 (0.2 - 0.2)	0.2 (0.2 - 0.2)	0.2 (0.1 - 0.2)	0.2 (0.2 - 0.2)	0.1 (0.1 - 0.1)	0.1 (0.1 - 0.1)	0.1 (0.1 - 0.1)	0.1 (0.1 - 0.1)	0.1 (0.0 - 0.1)	Men: -0.2<br>Women: -0.1

For peer review only

		65		0.5 (0.5 - 0.5)	1.1 (1.0 - 1.2)	0.6 (0.5 - 0.6)	1.5 (1.4 - 1.6)	0.7 (0.6 - 0.7)	1.6 (1.5 - 1.6)	0.5 (0.5 - 0.6)	1.2 (1.1 - 1.3)	0.4 (0.4 - 0.5)	0.9 (0.8 - 0.9)	Men: -0.1<br>Women: -0.2
		85		0.6 (0.5 - 0.7)	1.2 (1.1 - 1.3)	0.8 (0.7 - 1.0)	1.7 (1.5 - 1.8)	0.9 (0.8 - 1.0)	1.9 (1.8 - 2.0)	0.7 (0.6 - 0.8)	1.4 (1.3, 1.5 -	0.4 (0.4 - 0.5)	0.9 (0.8 - 1.0)	Men: -0.2<br>Women: -0.3
Cao 2016	DFLE (years)	55-85	T1: 1982<br>T2: 1990<br>T3: 2020<br>T4: 2030<br>T5: 2040	12.98	14.9	16.92	17.53	17.86 (17.70 - 18.03)	18.00 (17.89 - 18.13)	18.99 (18.68 - 19.36	18.67 (18.41 - 18.97)	20.10 (19.60 - 20.64)	19.30 (18.88 - 19.77)	Men: 7.12[b]<br>Women: 4.4[b]
Freedman (2016)a	DFLE (years)	65	T1: 1982<br>T2: 2004<br>T3: 2011	**data only reported in graphs, unable to extract**										Men: 4.5<br>Women: 1.4
		85		2.5	2.5	4.4	2.6							Men: 1.9<br>Women: 0.1[b]
Freedman (2016)b	Active Life Expectancy (years)	65	T1: 1982<br>T2: 2011	White: 12.2<br>Black: 9.8		White: 15.9<br>Black: 12.0								White: 2.8<br>Black: 2.2
		85		White: 2.5<br>Black: 2.25		White: 3.0<br>Black: 2.0								White: 0.5<br>Black: -0.25

[a]95% confidence intervals in brackets where reported; [b]Change estimate not reported, and thus calculated by us (T^2-T^1)

For peer review only

Table 3d. Summary of disability prevalence trends data for studies from the US and Canada[a]

Study	Trend	Time points	Change between first and last time point
Crimmins (2016)	Proportion (%) of individuals with disability in the community (age 65-84)	T1: 1970<br>T2: 1980<br>T3: 1990<br>T4: 2000<br>T5: 2010	Men: -2.5<br>Women: -2.2
	Proportion (%) of individuals with disability in the community (age 85+)		Men: -0.5<br>Women: -5.8
Freedman (2016)	Proportion of those with a severe limitation (limited in 3+ personal care activities or living in a nursing home)	T1: 1982<br>T2: 2004<br>T3: 2011	Men: -3.4[b]<br>Women: -3.0[b]

[a]Estimates for each time point were not extracted here due to volume. Reader is referred to publications and associated supplementary materials; [b]Change estimate not reported, and thus calculated by us (T^2-T^1)

For peer review only

Table 4a. Summary of life expectancy trends data for UK studies and studies with UK samples (years)[a]

Study author and date	At age	Time points	T1		T2		T3		Change between first and last time point
			Male	Female	Male	Female	Male	Female	
Reus-Pons 2017	50	T1: 2001<br>T2: 2011	28.54<br><br>Immigrant: 27.52<br>Non-immigrant: 28.66	32.32<br><br>Immigrant: 31.82<br>Non-immigrant: 32.39	31.29<br><br>Immigrant: 31.01<br>Non-immigrant: 31.32	34.5<br><br>Immigrant: 34.84<br>Non-immigrant: 34.47			Men: 2.8[b]<br>Women: 2.2[b]
Jagger 2016	65	T1: 1991<br>T2: 2011	13·0	16·7	17·5	20·3			Men: 4·5<br>Women: 3·6
Kingston 2018a	65	T1: 2015<br>T2: 2025<br>T3: 2035	18·7 (18·3 to 19·0)	21·1 (20·8 to 21·1)	20·7 (20·5 to 21·0)	22·7 (22·5 to 23·3)	22·2 (21·7 to 22·4)	24·1 (23·9 to 24·4)	Men: 3·5 (3·1 to 4·1)<br>Women: 3·0 (3·0 to 3·6)
Kingston 2017	65	T1: 1991<br>T2: 2011	12.9	16.5	17.6	20.6			Men: 4.7<br>Women: 4.1
ONS 2018	0	T1: 2009/12<br>T2: 2015/17	**Not reported**						UK<br>Men: 0.8<br>Women: 0.4<br>England<br>Men: 0.8<br>Women: 0.4<br>Northern Ireland<br>Men: 1.0<br>Women:0.5<br>Scotland<br>Men: 0.8<br>Women: 0.5

For peer review only

				Wales<br>Men: 0.5<br>Women: 0.2

[a]95% confidence intervals in brackets where reported; [b]Change estimate not reported in publication, and calculated by us (T^2-T^1);

For peer review only

Table 4b. Summary of healthy life expectancy trends data for UK studies and studies with UK samples[a]

Study	Trend	At age	Time points	T1		T2		Change between first and last time point
				Male	Female	Male	Female	
Reus-Pons 2017	HLE (years)	50	T1: 2001 T2: 2011	18.47 (18.46-18.48)	19.82 (19.81-19.83)	18.71 (18.70-18.72)	19.67 (19.66-, 19.68)	Men: 0.25 Women: -0.15
Jagger 2016	HLE (years)	65	T1: 1991 T2: 2011	8·8 (8·6-9·1)	11·2 (11·0 - 11·5)	12·6 (12·4-12·9)	14·3 (14·0 - 14·6)	Men: 3·8 (3·5 to 4·1) Women: 3·1 (2·7 to 3·4)
	Proportion (%) of life spent healthy			68·2% (66·5 - 69·9)	67·3% (65·9 - 68·7)	72·4% (70·9 - 73·9)	70·3% (68·8 - 71·7)	Men: 4·2% (2·0 to 6·5) Women: 3·0% (1·0 to 4·9)
Kingston 2018b	Life expectancy with 2+ diseases (years)	65	T1: 2015 T2: 2035	**Not reported**				Men: 5.5 Women: 5.0
ONS 2018	HLE (years) - UK	0	T1: 2009/11 T2: 2015/17	62.7	63.8	63.1	63.6	Men: 0.4 [b] Women: -0.2 [b]

[a]95% confidence intervals in brackets where reported; [b]Change estimate not reported in publication, and calculated by us (T^2-T^1);

For peer review only

Table 4c. Summary of disability-free life expectancy trends data for UK studies and studies with UK samples[a]

Study	Trend	At age	Time points	T1		T2		T3		Change between first and last time point
				Male	Female	Male	Female	Male	Female	
Jagger 2016	DFLE (years)	65	T1: 1991 T2: 2011	10.3 (10.2-10.5)	11.0 (10.8-11.2)	12.9 (12.7-13.2)	11.5 (11.3-11.8)			Men: 2.6 (2.3, 2.9) Women: 0.5 (0.2, 0.9)
	% of life disability-free			79.7 (78.3-81.0)	66.1 (64.9-67.4)	74.4 (73.0-75.8)	56.8 (55.5-58.2)			Men: -5.3 (-7.2, -3.4) Women: -9.3 (-11.1, -7.5)
	Cognitive impairment free LE (years)		T1: 1991 T2: 2011	9·4 (9·2 - 9·6)	10·1 (9·8 - 10·4)	13·6 (13·4 - 13·9)	14·5 (14·1 - 14·8)			Men: 4·2 (4·2 to 4·3) Women: 4·4 (4·3 to 4·5)
	% life expectancy free of cognitive impairment			72·4 (70·6 - 74·3)	60·5 (58·6 - 62·3)	78·2 (76·6 - 79·8)	71·2 (69·5 - 72·9)			Men: 5·8 (3·3 to 8·2) Women: 10·7 (8·2 to 13·2)
Kingston 2017	DFLE (years)	65	T1: 1991 T2: 2011	9.5 (9.3-9.7)	9.5 (9.2-9.8)	11.2 (10.8-11.5)	9.7 (9.3-10.2)			Men: 1.7 (1.2, 2.1) Women: 0.2 (-0.4, 0.7)
	% of life disability-free			73.6 (71.8-75.4)	58.0 (56.2-59.9)	63.5 (61.4-65.6)	47.3 (45.0-49.5)			Men: -10.1 (-12.9, -7.3) Women: -10.7 (-13.6, -7.8)
Kingston 2018a	DFLE (years)	65	T1: 2015 T2: 2025 T3: 2035	11.1 (10.9-11.3)	10.7 (10.5-10.7)	14.5 (14.4-14.6)	11.4 (11.3-11.5)	15.2 (15.1-15.2)	11.6 (11.6-11.8)	Men: 4.2 (3.9, 4.2) Women: 0.9 (0.9, 1.2)
	% of life disability-free			59.3 (59.3-60.2)	50.6 (50.1-50.7)	70.2 (69.7-70.4)	49.9 (49.4-50.3)	68.7 (67.9-69.5)	48.0 (48.0-48.6)	Men: 9.4 (8.0, 9.7) Women: -2.6%
Guzman-Castillo 2017	DFLE (years)	65	T1: 2015 T2: 2025	14.9 (14.7-15.1)	15.8 (15.7-15.9)	15.4 (15.3-15.5)	16.5 (15.4-17.6)	16.4 (15.1-17.1)	16.4 (15.5-17.3)	Men: 1.6 (0.5, 2.7) Women: 0.6 (-0.7, 1.9)

[a]95% confidence intervals in brackets where reported;

Table 4d. Summary of disability prevalence trends data for UK studies and studies with UK samples[a,b]

Study	Trend	Time points	Change between first and last time point
Jagger 2016	Odds of any disability at time 2 compared to time 1	T1: 1991	1.22 (1.14-1.30)
	Odds of moderate-severe disability at time 2 compared to time 1	T2: 2011	0.76 (0.70-0.82)
	Odds of fair or poor self-rated health at time 2 compared to time 1		0.83 (0.78–0.88)
	Odds of any cognitive impairment at time 2 compared to time 1		0.53 (0.49–0.56)
	Odds of severe cognitive impairment at time 2 compared to time 1		0.49 (0.43-0.56)
Kingston 2017	% increase in projected number of people with high dependency	T1: 2015	61.5
	% increase in projected number of people with medium dependency	T2: 2025	66.6
	% increase in projected number of care home places needed for those with high dependency	T3: 2035	84.5
	% increase in projected number of care home places needed for those with medium dependency		89.3
Kingston 2018a	Relative change in projected proportion (%) of sample with high dependency (65-74 years)	T1: 2015	−15.0 (−20.0 to −13.0)
	Relative change in projected proportion (%) of sample with high dependency (75 - 84 years)	T2: 2025	42.0 (36.6- 42.7)
	Relative change in projected proportion (%) of sample with high dependency (85+ years)	T3: 2035	91.8 (87.3- 94.1)
	Relative change in projected proportion (%) of sample with high dependency (65+)		36.0 (32.6- 36.0)
Kingston 2018b	Percentage change in projected proportion of those with 2 or more diseases between 2015 and 2035 (65-74)	T1: 2015	51.5
	Percentage change in projected proportion of those with 2 or more diseases between 2015 and 2035 (75-84)	T2: 2025	85.4
	Percentage change in projected proportion of those with 2 or more diseases between 2015 and 2035 (85+)	T3: 2035	181.6
	Percentage change in projected proportion of those with 4 or more diseases between 2015 and 2035 (65-74)		20.9
	Percentage change in projected proportion of those with 4 or more diseases between 2015 and 2035 (75-84)		130.3
	Percentage change in projected proportion of those with 4 or more diseases between 2015 and 2035 (85+)		470.2
Guzman-Castillo 2017	Relative change in projected number of disability cases 2015-2025, 65+	T1: 2015	25.0 (21.3–28.2)
	Relative change in projected number of disability cases 2015-2025, 65-84	T2: 2025	18.9 (16.6–20.9)
	Relative change in projected number of disability cases 2015-2025, 85		43.2 (34.2–52.1)

[a]Estimates for each time point were not extracted here due to volume. Reader is referred to publications and associated supplementary materials; [b]95% confidence intervals in brackets where reported;

For peer review only

Table 4e. Summary of inequalities in life expectancy trends, reported as the slope index of inequality between those living in the most and least deprived decile of area deprivation

Study	Trend	Time points	Change between first and last time point (range)
ONS 2019	Change in SSI in life expectancy at birth, England	T1: 2012/14<br>T2: 2015/17	Men: 0.3 (0.2)<br>Women: 0.5 (0.5)
	Change in SSI in life expectancy at age 65, England		Men: 0.3 (0.2)<br>Women: 0.5 (0.5)
	Change in SSI in life expectancy at birth, Wales		Men: 0.5 (0.3)<br>Women: 0.6 (0.7)
	Change in SSI in life expectancy at age 65, Wales		Men: 0.3 (0.2)<br>Women: 0.1 (0.3)

For peer review only

Table 4f. Summary of inequalities in healthy life expectancy trends, reported as the slope index of inequality between those living in the most and least deprived decile of area deprivation

Study	Trend	Time points	Change between first and last time point (range)
ONS 2019	Change in SSI in healthy life expectancy at birth, England	T1: 2012/14<br>T2: 2015/17	Men: 0.2 (0.2)<br>Women: -0.9 (-0.8)
	Change in SSI in healthy life expectancy at age 65, England		Men: 0.4 (0.9)<br>Women: -0.3 (-0.6)
	Change in SSI in healthy life expectancy at birth, Wales		Men: 0.2 (0.9)<br>Women: -1.1 (-0.9)
	Change in SSI in healthy life expectancy at age 65, Wales		Men: -0.6 (0.8)<br>Women: -1.0 (0.2)

For peer review only

For peer review only

Table 5a. Summary of life expectancy trends data for studies from Europe (years)[a]

Study	At age	Time points	T1		T2		T3		T4		T5		T6		T7		T8		Change between first and last time point
			M	F	M	F	M	F	M	F	M	F	M	F	M	F	M	F	
Bronnum-Hansen 2017	65	T1: 2006-2007<br>T2: 2010-2011<br>T3: 2013-2014	High: 17.9<br>Med: 16.3<br>Low: 15.6	High: 20.4<br>Med: 19.4<br>Low: 18.5	Med: 16.8<br>Low: 16.2	High: 21.0<br>Med: 20.0<br>Low: 18.9	High: 19.3<br>Med: 17.5<br>Low: 16.9	High: 21.7<br>Med: 20.6<br>Low: 19.5											Difference in change in LE between high and low education over time:<br>Men: 0.1<br>Women: 0.3
Deeg 2018	65	T1: 1993<br>T2: 1996<br>T3: 1999<br>T4: 2002<br>T5: 2006<br>T6: 2009<br>T7: 2012<br>T8: 2016	14.7	19.2	15.1	19.4	15.5	19.5	16	19.7	17.1	20.5	17.8	21.1	18.2	21.2	18.7	21.4	Men: 4.0<br>Women: 2.2
Gheorghe 2016[b]	25	T1: 2001<br>T2: 2011	High: 53.83<br>Med: 50.70<br>Low: 47.85	High: 58.75<br>Med: 56.82<br>Low: 53.57	High: 56.71<br>Med: 53.92<br>Low: 50.28	High: 60.53<br>Med: 58.35<br>Low: 54.72													Men:<br>High: 2.88<br>Med: 3.22<br>Low: 2.43<br>Women:<br>High: 1.78<br>Med: 1.53<br>Low: 1.15
	65		High: 16.81<br>Med: 15.29<br>Low: 14.08	High: 21.00<br>Med: 20.08<br>Low: 18.26	High: 19.29<br>Med: 17.53<br>Low: 15.76	High: 22.64<br>Med: 21.49<br>Low: 19.38													Men:<br>High: 2.48<br>Med: 2.24<br>Low: 1.68<br>Women:<br>High: 1.64<br>Med: 1.41<br>Low: 1.12
Grasset 2019[c]	70	T1: 1990<br>T2: 2000	15.6 (14.8–16.2)		18.6 (17.9–19.2)														3.0[d]
	75		12.3 (11.7–12.8)		14.7 (14.0–15.2)														2.4[d]
	80		9.4 (8.9–9.9)		11.2 (10.6–11.6)														1.8[d]
	85		7.1 (6.5–7.6)		8.4 (7.8–9.0)														1.3[d]
	90		5.2 (4.6–5.8)		6.0 (5.5–6.4)														0.8[d]
Remund 2019	30	T1: 1990/94<br>T2: 1995/99	**data only reported in graphs, unable to extract**																Men: 5.02<br>Women: 3.09

For peer review only

		T3: 2000/04<br>T4: 2005/09<br>T5: 2010/14																	
Renard 2019[b]	25	T1: 2001<br>T2: 2011	High: 55.82<br>Med: 53.18<br>Low: 50.63	High: 60.90<br>Med: 59.14<br>Low: 57.14	High: 57.81<br>Med: 54.73<br>Low: 51.74	High: 61.90<br>Med: 60.09<br>Low: 57.32													Men:<br>Low: 1.11<br>Med: 1.55<br>High: 1.99<br><br>WOMEN:<br>Low: 0.18<br>Med: 0.95<br>High: 1.00
Reus-Pons 2017	50	T1: 2001<br>T2: 2011	28.05	32.47	30.87	34.31													Men: 2.82 [d]<br>Women: 1.84 [d]
Storeng 2018	30	T1: 1984/86<br>T2: 1995/97<br>T3: 2006/08	45.1 (44.6–45.5)	50.4 (49.9–50.8)	48.3 (47.8–48.7)	52.9 (52.5–53.4)	52.1 (51.5–52.6)	57.1 (56.6–57.6)											Men: 6.99 (5.27–8.72)<br>Women: 6.75 (5.16–8.34)
			Primary: 47.0 (46.5–47.5)<br>Secondary: 47.9 (47.4–48.5)<br>Tertiary: 51.2 (49.6–52.7)		Primary: 49.7 (49.1–50.2)<br>Secondary: 50.9 (50.4–51.4)<br>Tertiary: 52.0 (50.9–53.1)		Primary: 52.2 (51.0–53.4)<br>Secondary: 54.9 (54.3–55.4)<br>Tertiary: 55.2 (53.1–57.4)												Primary: 5.19 (3.52–6.85)<br>Secondary: 6.95 (5.87–8.02)<br>Tertiary: 3.23 (–0.04–6.51)
Sundburg 2016	77	SWEOLD<br>T1: 1992<br>T2: 2002<br>T3: 2004<br>T4: 2011	8.2	10.7	8.9	10.8	9.3	11.4	9.9	11.8									Men: 1.7 [d]<br>Women: 1.1 [d]
		SHARE:<br>T1: 2004<br>T2: 2011	9.3	11.4	9.9	11.8													Men: 0.6 [d]<br>Women: 0.4 [d]
Yokota 2016	15	T1: 2001<br>T2: 2004<br>T3: 2008	60.5	66.4	61.4	67	62.1	67.4											Men: 1.6 [d]<br>Women: 1.0[d]

[a]95% confidence intervals in brackets where reported; [b] Trends reported by high, medium and low education; [c]Trends not reported by sex; [d]Change estimate not reported, and thus calculated by us (T^2-T^1)

For peer review only

Table 5b. Summary of healthy life expectancy trends data for studies from Europe[a]

Study	Trend	At age	Time points	T1		T2		T3		T4		T5		T6		T7		T8		Change between first and last time point
				M	F	M	F	M	F	M	F	M	F	M	F	M	F	M	F	
Deeg 2018	% life in good physical health	65	T1: 1993<br>T2: 1996<br>T3: 1999<br>T4: 2002<br>T5: 2006<br>T6: 2009<br>T7: 2012<br>T8: 2016	46.6	28.7	37.6	26.0	33.6	24.5	32.4	22.7	32.7	21.2	30.5	20.2	22.8	14.9	25.0	18.9	Men: -21.6<br>Women: -9.8
	% life in good cognitive health			74.8	69.9	78.0	73.7	81.5	76.1	83.8	78.2	81.5	78.9	82.8	82.4	85.5	84.4	83.7	84.4	Men: 8.9<br>Women: 15.5
	HLE: Physical (years)			6.9	5.5													4.7	4.0	Men: -2.2[c]<br>Women: -1.5[c]
	HLE: Cognitive (years)			11.0	13.4													15.7	18.0	Men: 4.7[c]<br>Women: 4.6[c]
Gheorghe 2016[b]	Quality Adjusted Life Expectancy	25	T1: 2001<br>T2: 2011	High: 44.08<br>Med: 40.82<br>Low: 36.71	High: 45.79<br>Med: 44.2<br>Low: 39.51	High:46.93<br>Med: 43.83<br>Low: 38.84	High: 48.43<br>Med: 45.77<br>Low: 41.31													MEN:<br>High 2.85<br>Med: 3.01<br>Low: 2.13<br><br>WOMEN:<br>High: 2.64<br>Med: 1.57<br>Low: 1.80
		65		High: 13.42<br>Med: 12.06<br>Low: 10.73	High: 15.66<br>Med: 15.12<br>Low: 13.09	High: 15.59<br>Med: 13.97<br>Low: 12.10	High: 17.40<br>Med: 16.33<br>Low: 14.23													MEN:<br>High: 2.17<br>Med: 1.91<br>Low: 1.37<br><br>WOMEN:<br>High: 1.74<br>Med: 1.21<br>Low: 1.14
Remund 2019	HLE (years)	30	T1: 1990/94<br>T2: 1995/99<br>T3: 2000/04<br>T4: 2005/09	**data only reported in graphs, unable to extract**																Men: 4.52<br>Women: 3.09

For peer review only

	HLE (years), educational gap between primary and tertiary attainment		T5: 2010/14	7.6	3.3	**not reported**						8.8	5.0							Change in educational gap (between primary and tertiary): Men: 1.2[c] Women: 1.7[c]
Reus-Pons 2017	HLE (years)	50	T1: 2001 T2: 2011	18.62 (18.40 - 18.84)	19.43 (19.19 - 19.68)	20.83 (20.55 - 21.10)	20.68 (20.37 - 20.99)													Men: 2.21 Women: 1.25
Storeng 2018[d]	HLE (years)	30	T1: 1984/86 T2: 1995/97 T3: 2006/08	31.6 (31.2–31.9)	32.6 (32.3–33.0)	34.1 (33.7–34.5)	33.9 (33.6–34.3)	38.5 (38.0–39.0)	38.0 (37.5–38.5)											Men: 6.90 (6.08–7.73) Women: 5.40 (4.56–6.25)
				Primary:29.5 (29.2–29.9) Secondary:34.0 (33.6–34.5) Tertiary: 40.1 (38.8–41.4)		Primary:29.8 (29.4–30.3) Secondary: 35.7 (35.3–36.2) Tertiary: 40.3 (39.3–41.3)		Primary: 31.5 (30.6–32.4) Secondary:38.8 (38.3–39.4) Tertiary: 42.8 (41.9–43.8)												Primary: 1.94 (0.68–3.20) Secondary: 4.80 (3.85–5.75) Tertiary: 2.76 (0.49–5.02) Change in gap between primary and tertiary education: 0.7[c]

[a]95% confidence intervals in brackets where reported; [b]Trends reported by high, medium and low education; [c]Change estimate not reported, and thus calculated by us (T^2-T^1); [d]Trends reported by primary, secondary and tertiary education;

For peer review only

Table 5c. Summary of disability-free life expectancy trends data for studies from Europe (years)[a]

Study	Trend	At age	Time points	T1		T2		T3		T4		Change between first and last time point
				Male	Female	Male	Female	Male	Female	Male	Female	
Bronnum-Hansen 2017[b]	DFLE	65	T1: 2006-2007<br>T2: 2010-2011<br>T3: 2013-2014	High: 10.6 (9.2–12.1)<br>Med: 9.4 (8.3–10.5)<br>Low: 7.4 (5.9–8.9)	High: 12.5 (10.5–14.5)<br>Med: 9.5 (8.1–10.9)<br>Low: 8.8 (7.7–9.9)	High: 10.6 (9.2–12.0)<br>Med: 9.3 (8.2–10.4)<br>Low: 7.0 (5.3–8.8)	High: 13.2 (10.9–15.6)<br>Med: 10.4 (8.9–11.8)<br>Low: 7.9 (6.6–9.3)	High: 10.9 (9.9–12.0)<br>Med: 9.6 (8.8–10.5)<br>Low: 8.0 (6.7–9.3)	High: 12.9 (11.2–14.6)<br>Med: 10.2 (9.0–11.4)<br>Low: 9.5 (8.5–10.5)			Difference in change in DFLE between those with the lowest and highest education<br>Men: -0.3<br>Women: -0.3
Grasset 2019[c]	Dementia Free LE	70	T1: 1990<br>T2: 2000	13.6 (12.9–14.0)		16.3 (15.6–16.7)						2.7[d]
		75		10.2 (9.7–10.6)		12.4 (11.8–12.7)						2.2 [d]
		80		7.4 (6.9–7.9)		8.9 (8.5–9.3)						1.5 [d]
		85		5.3 (4.8–5.8)		6.4 (6.0–6.9)						1.1 [d]
		90		3.8 (3.4–4.3)		4.5 (4.1–5.0)						0.7 [d]
Lagergren 2017	Added years of life without ADL limitations	65+	T1: 1980/85<br>T2: 1994/99<br>T3: 2006/11	**data not reported**								Men: 4.0<br>Women: 3.14
	Added years of life without mobility limitations			**data not reported**								Men: 4.21<br>Women: 3.69
Renard 2019	DFLE	25	T1: 2001<br>T2: 2011	Low: 36.34<br>Med: 41.40<br>High: 42.85	Low: 39.32<br>Med: 43.37<br>High: 48.62	Low:37.02<br>Med: 42.05<br>High: 47.49	Low:35.54<br>Med:42.98<br>High: 48.98					Difference in change in DFLE between levels of education (ref high education)

For peer review only

												change over time<br>Men:<br>Low: 3.96<br>Med: 3.99<br><br>Women:<br>Low: 4.14<br>Med: 0.76
Storeng 2018[e]	DFLE	30	T1: 1984/86<br>T2: 1995/97<br>T3: 2006/08	25.9 (25.6–26.2)	28.8 (28.6–29.1)	28.5 (28.1–28.8)	29.4 (29.0–29.7)	28.6 (28.2–29.0)	29.2 (28.8–29.6)			Men: 2.71 (2.01–3.42)<br>Women: 0.33 (–0.40–1.06)
				Primary: 25.0 (24.7–25.3)<br>Secondary: 28.8 (28.5–29.2)<br>Tertiary: 33.6 (32.5–34.7)		Primary: 25.3 (24.9–25.7)<br>Secondary: 30.1 (29.7–30.5)<br>Tertiary: 33.0 (32.3–33.8)		Primary: 22.5 (21.7–23.2)<br>Secondary: 29.1 (28.6–29.5)<br>Tertiary: 33.2 (32.4–34.1)				Primary: –2.53 (–3.61–1.45)<br>Secondary: 0.23 (–0.57–1.03)<br>Tertiary: –0.32 (–2.23–1.60)
Sundburg 2016	DFLE	77	SWEOLD<br>T1: 1992<br>T2: 2002<br>T3: 2004<br>T4: 2011	6.7 (6.3–7.1)	6.9 (6.4–7.4)	6.9 (6.4–7.3	7.0 (6.5–7.5)	7.4 (7.0–7.9)	7.9 (7.4–8.4)	7.8 (7.2–8.3)	8.5 (8.0–9.0	Men: 1.1<br>Women: 1.6
			SHARE:<br>T1: 2004<br>T2: 2011	7.5 (7.0–8.0	7.9 (7.3–8.6	7.6 (7.0–8.1)	9.2 (8.5–9.8)					Men: 0.1<br>Women: 1.3
Yokota 2019	DFLE	15	T1: 2001<br>T2: 2004<br>T3: 2008	54.1	56.0	54.9	56.2	54.8	55.3			Men: 0.7<br>Women: -0.7

[a]95% confidence intervals in brackets where reported; [b]Stratified by high, medium and low education; [c]Not stratified by sex; [d]Change estimate not reported, and thus calculated by us (T^2-T^1) [e]Second row stratified by primary, secondary and tertiary education for men and women combined

For peer review only

Table 5d. Summary of disability prevalence trends data for studies from Europe

Study	Trend	Time points	Change between first and last time point[a]
Lagergren 2017	Proportion reporting dependency in activities of daily living, 65-69	T1:1980/85<br>T2: 1994/95<br>T3: 2006/11	Men:-1.5<br>Women:-3.7
	Proportion reporting dependency in activities of daily living, 70-74		Men:-5.6<br>Women:-3.5
	Proportion reporting dependency in activities of daily living, 75-79		Men:-10.7<br>Women:-7.1
	Proportion reporting dependency in activities of daily living, 80-84		Men: -11.5<br>Women: -14.0
	Proportion reporting dependency in activities of daily living, 85+		Men:-20.3<br>Women: -10.5
	Proportion reporting mobility limitations, 65-69		Men:-6.5<br>Women: -8.3
	Proportion reporting mobility limitations, 70-74		Men: -10.9<br>Women: -8.5
	Proportion reporting mobility limitations, 75-79		Men: -15.7<br>Women: -14.8
	Proportion reporting mobility limitations, 80-84		Men: -15.1<br>Women: -19.4
	Proportion reporting mobility limitations, 85+		Men: -16.1<br>Women: -17.5

[a]Change estimate not reported, and thus calculated by us (T^2-T^1)

For peer review only

Table 6a. Summary of life expectancy trends data for studies from Japan and Korea (years)[a]

Study	At age	Time points	T1		T2		T3		T4		T5		T6		T7		T8		Change between first and last time point
			M	F	M	F	M	F	M	F	M	F	M	F	M	F	M	F	
Jo 2019	0	T1: 2005<br>T2: 2007<br>T3: 2008<br>T4: 2009<br>T5: 2010<br>T6: 2011<br>T7: 2012	75.13	81.87	76.13	82.71	76.54	83.27	76.99	83.74	77.19	84.04	77.64	84.42	77.94	84.61	78.51	85.02	Men: 3.38[b] Women: 3.15[b]
Lee 2016	0	T1: 2005<br>T2: 2008<br>T3: 2011	75.1	81.8	76.5	83.2	77.6	84.4											Men: 2.5[b] Women: 2.6[b]
	65		15.8	19.8	16.6	21.0	17.4	21.8											Men: 1.6[b] Women: 2.0[b]
	85		5.1	6.2	5.2	6.5	5.4	6.9											Men: 0.3[b] Women: 0.7[b]
Sugawara 2016	0	T1: 2000<br>T2: 2010	77.6	84.7	79.5	86.4													Men: 1.9 Women:1.7
Tokudome 2016	0	T1: 1990<br>T2: 1995<br>T3: 2000<br>T4: 2005<br>T5: 2010<br>T6: 2013	76.04 (75.98–76.10	81.96 (81.86–82.05	76.45 (76.14–76.57	82.84 (82.62–82.94	77.55 (77.53–77.58	84.32 (84.29–84.35	78.66 (78.60–78.71	85.48 (85.41–85.54	79.34 (79.31–79.36	85.09 (86.06–86.11	80.05 (79.26–80.84	86.39 (85.74–87.12					Men: 4.01[b] Women: 4.43[b]

[a]95% confidence intervals in brackets where reported; [b]Change estimate not reported, and thus calculated by us (T^2-T^1)

For peer review only

Table 6b. Summary of healthy life expectancy trends data for studies from Japan and Korea (years)[a]

Study	At age	Time points	T1		T2		T3		T4		T5		T6		T7		T8		Change between first and last time point
			M	F	M	F	M	F	M	F	M	F	M	F	M	F	M	F	
Jo 2019	0	T1: 2005<br>T2: 2007<br>T3: 2008<br>T4: 2009<br>T5: 2010<br>T6: 2011<br>T7: 2012	70.8	74.7	71.86	73.92	72.62	75.18	73.4	76.65	73.87	77.21	74.33	76.53	74.71	78.3	74.83	78.14	Men: 4.03<br>Women: 3.44
Lee 2016	0	T1: 2005<br>T2: 2008<br>T3: 2011	64.4	67.7	64.0	67.3	65.8	68.9											Men: 1.4[b]<br>Women: 1.2[b]
	65		9.5	11.5	10.4	12.8	11.7	14.1											Men: 2.2[b]<br>Women: 2.6[b]
	85		2.6	3.8	3.3	4.7	4.0	5.2											Men: 1.4[b]<br>Women: 1.4[b]
Tokudome 2016	0	T1: 1990<br>T2: 1995<br>T3: 2000<br>T4: 2005<br>T5: 2010<br>T6: 2013	68.09 (65.83–70.11)	72.24 (69.38–74.77)	68.44 (66.07–70.58)	72.92 (70.05–75.49)	69.08 (66.67–71.24)	73.95 (70.94–76.55)	69.89 (67.31–72.12)	74.77 (71.66–77.46)	70.78 (68.20–73.06)	75.41 (72.34–78.23)	71.11 (68.50–73.57))	75.56 (72.46–78.42)					Men: 3.02[b]<br>Women: 3.32[b]

[a]95% confidence intervals in brackets where reported; [b]Change estimate not reported, and thus calculated by us (T^2-T^1)

Table 6c. Summary of disability-free life expectancy trends data for studies from Japan[a]

Study	Trend	At age	Time points	T1		T2		Change between first and last time point
				Male	Female	Male	Female	
Suguwara 2016	DFLE (years)	0	T1: 2000<br>T2: 2010	68.0	71.7	69.0	72.1	Men: 1.0<br>Women: 0.4
	% of life disability-free			87.6	84.6	86.8	83.4	Men: -0.8<br>Women: -1.2

[a]95% confidence intervals in brackets where reported;

For peer review only

Table 7a. Summary of life expectancy trends data high income countries combined from the GBD studies (years)[a]

Study author and date	At age	Time points	T1		T2		Change between first and last time point
			Male	Female	Male	Female	
GBD 2016	0	T1: 2005<br>T2: 2015	76·35 (76·33–76·37)	82·09 (82·07–82·11)	78·10 (78·05–78·15)	83·42 (83·38–83·46)	Men: 1.75[b]<br>Women: 1.33[b]
GBD 2017	0	T1: 1990<br>T2: 2016	72·64 (72·54–72·75)	79·34 (79·24–79·43)	78·27 (78·04–78·49)	83·48 (83·28–83·67)	Men: 5.63[b]<br>Women: 4.14[b]
	65		14·96 (14·90–15·01)	18·77 (18·71–18·84)	18·47 (18·32–18·60)	21·80 (21·66–21·94)	Men: 3.51[b]<br>Women: 3.03[b]
GBD 2018	0	T1: 1990<br>T2: 2017	72·8 (72·7–72·8)	79·4 (79·4–79·4)	78·4 (78·2–78·6)	83·6 (83·4–83·7)	Men: 5.6[b]<br>Women: 4.2[b]

[a]95% confidence intervals in brackets where reported; [b]Change estimate not reported in publication, and calculated by us (T^2-T^1);

For peer review only

Table 7b. Summary of healthy life expectancy trends data for high income countries combined from the GBD studies[a]

Study	Trend	At age	Time points	T1		T2		Change between first and last time point
				Male	Female	Male	Female	
GBD 2016	HLE (years)	0	T1: 2005<br>T2: 2015	67.48 (64.90–69.75)	71.13 (67.95–73.87)	68.91 (66.27–71.25)	72.21 (68.97–75.02)	Men: 1.43[b]<br>Women: 1.08[b]
GBD 2017	HLE (years)	0	T1: 1990<br>T2: 2016	64.15 (61.70–66.33)	68.40 (65.23–71.19)	68.58 (65.82–71.06)	71.61 (68.12–74.66)	Men: 4.43[b]<br>Women: 3.21[b]
		65		11.0 (10.45–12.27)	14.28 (13.06–15.39)	14.00 (12.79–15.11)	16.55 (15.09–17.85)	Men: 2.60[b]<br>Women: 2.27[b]
GBD 2018	HLE (years)	0	T1: 1990<br>T2: 2017	64.0 (61.4–66.1)	68.0 (64.7–70.8)	68.2 (65.3–70.7)	71.0 (67.5–74.0)	Men: 3.0[b]<br>Women: 4.2[b]

[a]95% confidence intervals in brackets where reported; [b]Change estimate not reported in publication, and calculated by us (T^2-T^1);

For peer review only

Search strategy as applied to Medline

Database(s): **Ovid MEDLINE(R) and In-Process & Other Non-Indexed Citations** 1946 to September 13, 2019

#	Searches	Results
1	Life Expectancy/	16893
2	Longevity/	20149
3	Needs assessment/	28915
4	((expectancy or Dependen* or disabilit*) adj2 years).ti,ab.	968
5	(expectanc* adj5 disabilit*).ti,ab.	406
6	(Care need* adj2 years).ti,ab.	13
7	(healthy life years or "years of healthy life").ti,ab,kw.	260
8	or/1-7	65417
9	exp Age Factors/ or Race Factors/ or Sex Factors/	631787
10	Time Factors/	1159988
11	exp Forecasting/	82762
12	Life Tables/	6387
13	td.fs.	367819
14	or/9-13	2116951
15	8 and 14	14752
16	Life Expectancy/td [Trends]	2146
17	Needs Assessment/td [Trends]	249

For peer review only

18	((life expectancy or expected years or care need*) adj2 (trend* or direction* or factor* or forecast*)).ti,ab.	334
19	or/16-18	2649
20	15 or 19	14962
21	limit 20 to (humans and yr="2016 -Current")	1661
22	letter/	1034636
23	Editorial/	498100
24	News/	196781
25	exp Historical Article/	390531
26	Anecdotes as Topic/	4730
27	Comment/	799622
28	(letter or comment$).ti.	138712
29	or/22-28	2365158
30	21 not 29	1502

For peer review only

PRISMA 2009 Checklist

Section/topic	#	Checklist item	Reported on page #
**TITLE**			
Title	1	Identify the report as a systematic review, meta-analysis, or both.	1
**ABSTRACT**			
Structured summary	2	Provide a structured summary including, as applicable: background; objectives; data sources; study eligibility criteria, participants, and interventions; study appraisal and synthesis methods; results; limitations; conclusions and implications of key findings; systematic review registration number.	2
**INTRODUCTION**			
Rationale	3	Describe the rationale for the review in the context of what is already known.	3-4
Objectives	4	Provide an explicit statement of questions being addressed with reference to participants, interventions, comparisons, outcomes, and study design (PICOS).	4
**METHODS**			
Protocol and registration	5	Indicate if a review protocol exists, if and where it can be accessed (e.g., Web address), and, if available, provide registration information including registration number.	NA
Eligibility criteria	6	Specify study characteristics (e.g., PICOS, length of follow-up) and report characteristics (e.g., years considered, language, publication status) used as criteria for eligibility, giving rationale.	4-5
Information sources	7	Describe all information sources (e.g., databases with dates of coverage, contact with study authors to identify additional studies) in the search and date last searched.	4
Search	8	Present full electronic search strategy for at least one database, including any limits used, such that it could be repeated.	4
Study selection	9	State the process for selecting studies (i.e., screening, eligibility, included in systematic review, and, if applicable, included in the meta-analysis).	5-6
Data collection process	10	Describe method of data extraction from reports (e.g., piloted forms, independently, in duplicate) and any processes for obtaining and confirming data from investigators.	7
Data items	11	List and define all variables for which data were sought (e.g., PICOS, funding sources) and any assumptions and simplifications made.	4-5
Risk of bias in individual studies	12	Describe methods used for assessing risk of bias of individual studies (including specification of whether this was done at the study or outcome level), and how this information is to be used in any data synthesis.	6-7
Summary measures	13	State the principal summary measures (e.g., risk ratio, difference in means).	7-8
Synthesis of results	14	Describe the methods of handling data and combining results of studies, if done, including measures of consistency (e.g., I^2) for each meta-analysis.	7-8

For peer review only - http://bmjopen.bmj.com/site/about/guidelines.xhtml

PRISMA 2009 Checklist

Section/topic	#	Checklist item	Reported on page #
Risk of bias across studies	15	Specify any assessment of risk of bias that may affect the cumulative evidence (e.g., publication bias, selective reporting within studies).	NA
Additional analyses	16	Describe methods of additional analyses (e.g., sensitivity or subgroup analyses, meta-regression), if done, indicating which were pre-specified.	NA
**RESULTS**			
Study selection	17	Give numbers of studies screened, assessed for eligibility, and included in the review, with reasons for exclusions at each stage, ideally with a flow diagram.	8, and figure 1
Study characteristics	18	For each study, present characteristics for which data were extracted (e.g., study size, PICOS, follow-up period) and provide the citations.	Supplement materials table 1
Risk of bias within studies	19	Present data on risk of bias of each study and, if available, any outcome level assessment (see item 12).	Supplementary materials table 2
Results of individual studies	20	For all outcomes considered (benefits or harms), present, for each study: (a) simple summary data for each intervention group (b) effect estimates and confidence intervals, ideally with a forest plot.	Table 3, Supplementary materials tables 3-7
Synthesis of results	21	Present results of each meta-analysis done, including confidence intervals and measures of consistency.	NA – no meta analysis
Risk of bias across studies	22	Present results of any assessment of risk of bias across studies (see Item 15).	Supplementary materials table 2
Additional analysis	23	Give results of additional analyses, if done (e.g., sensitivity or subgroup analyses, meta-regression [see Item 16]).	NA
**DISCUSSION**			
Summary of evidence	24	Summarize the main findings including the strength of evidence for each main outcome; consider their relevance to key groups (e.g., healthcare providers, users, and policy makers).	12-15
Limitations	25	Discuss limitations at study and outcome level (e.g., risk of bias), and at review-level (e.g., incomplete retrieval of identified research, reporting bias).	13-14
Conclusions	26	Provide a general interpretation of the results in the context of other evidence, and implications for future	12-15

For peer review only

PRISMA 2009 Checklist

		research.	
**FUNDING**			
Funding	27	Describe sources of funding for the systematic review and other support (e.g., supply of data); role of funders for the systematic review.	Title page

From: Moher D, Liberati A, Tetzlaff J, Altman DG, The PRISMA Group (2009). Preferred Reporting Items for Systematic Reviews and Meta-Analyses: The PRISMA Statement. PLoS Med 6(7): e1000097. doi:10.1371/journal.pmed1000097

For more information, visit: **www.prisma-statement.org**.

For peer review only

BMJ Open

Trends in health expectancies: a systematic review of international evidence

Journal:	*BMJ Open*
Manuscript ID	bmjopen-2020-045567.R1
Article Type:	Original research
Date Submitted by the Author:	09-Mar-2021
Complete List of Authors:	Spiers, Gemma; Newcastle University, Population & Health Sciences Institute<br>Kunonga, Tafadzwa; Newcastle University, Population & Health Sciences Institute<br>Beyer, Fiona ; Newcastle University, Population & Health Sciences Institute<br>Craig, Dawn; Newcastle University, Population & Health Sciences Institute<br>Hanratty, Barbara; Newcastle University, Population & Health Sciences Institute<br>Jagger, Carol; Newcastle University, Population & Health Sciences Institute
**Primary Subject Heading**:	Health policy
Secondary Subject Heading:	Epidemiology
Keywords:	Health policy < HEALTH SERVICES ADMINISTRATION & MANAGEMENT, Organisation of health services < HEALTH SERVICES ADMINISTRATION & MANAGEMENT, GERIATRIC MEDICINE

SCHOLARONE™
Manuscripts

I, the Submitting Author has the right to grant and does grant on behalf of all authors of the Work (as defined in the below author licence), an exclusive licence and/or a non-exclusive licence for contributions from authors who are: i) UK Crown employees; ii) where BMJ has agreed a CC-BY licence shall apply, and/or iii) in accordance with the terms applicable for US Federal Government officers or employees acting as part of their official duties; on a worldwide, perpetual, irrevocable, royalty-free basis to BMJ Publishing Group Ltd ("BMJ") its licensees and where the relevant Journal is co-owned by BMJ to the co-owners of the Journal, to publish the Work in this journal and any other BMJ products and to exploit all rights, as set out in our licence.

The Submitting Author accepts and understands that any supply made under these terms is made by BMJ to the Submitting Author unless you are acting as an employee on behalf of your employer or a postgraduate student of an affiliated institution which is paying any applicable article publishing charge ("APC") for Open Access articles. Where the Submitting Author wishes to make the Work available on an Open Access basis (and intends to pay the relevant APC), the terms of reuse of such Open Access shall be governed by a Creative Commons licence – details of these licences and which Creative Commons licence will apply to this Work are set out in our licence referred to above.

Other than as permitted in any relevant BMJ Author's Self Archiving Policies, I confirm this Work has not been accepted for publication elsewhere, is not being considered for publication elsewhere and does not duplicate material already published. I confirm all authors consent to publication of this Work and authorise the granting of this licence.

For peer review only

Trends in health expectancies: a systematic review of international evidence

Dr Gemma Spiers,[a]* gemma-frances.spiers@newcastle.ac.uk

Ms Tafadzwa Patience Kunonga[a]

Ms Fiona Beyer[b]

Professor Dawn Craig[b]

Professor Barbara Hanratty[a]

Professor Carol Jagger[a]

[a] Population and Health Sciences Institute, Newcastle University, Campus for Ageing & Vitality, Newcastle upon Tyne, NE4 5PL

[b] Population and Health Sciences Institute, Newcastle University, Baddiley-Clark, Newcastle upon Tyne, NE2 4AX

*Corresponding author

Word count: 3301 excluding abstract, references, tables and figures

Number of references: 43

Number of tables and figures: 4

For peer review only

Trends in health expectancies: a systematic review of international evidence

OBJECTIVES

A clear understanding of whether increases in longevity are spent in good health is necessary to support ageing, health and care-related policy.

DESIGN

We conducted a systematic review to update and summarise evidence on trends in health expectancies, in OECD high-income countries.

DATA SOURCES

Four electronic databases (MEDLINE, 1946 to September 19th 2019; Embase 1980 to 2019 Week 38; Scopus 1966 to 22nd September 2019, Health Management Information Consortium, 1979 to September 2019), and the UK Office for National Statistics website (November 2019).

ELIGIBILITY CRITERIA

English language studies published from 2016 that reported trends in healthy, active, and/or disability-free life expectancy in an OECD high-income country.

DATA EXTRACTION AND SYNTHESIS

Records were screened independently by two researchers. Study quality was assessed using published criteria designed to identify sources of bias in studies reporting trends, and evidence summarised by narrative synthesis.

FINDINGS

Twenty-eight publications from eleven countries were included, covering periods from six to 40 years, between 1970 and 2017. In most countries, gains in healthy and disability-free life expectancy do not match the growth in total life expectancy. Exceptions were demonstrated for women in Sweden, where there were greater gains in disability-free years than life expectancy. Gains in healthy and disability-free life expectancy were greater for men than women in most countries except the US (age 85), Japan (birth), Korea (age 65) and Sweden (age 77).

CONCLUSION

An expansion of disability in later life is evident in a number of high-income countries, with implications for the sustainability of health and care systems. The recent COVID-19 pandemic may adversely impact health expectancies in the longer term.

Keywords: Trends, Life Expectancy, Disability, Health, Ageing

For peer review only - http://bmjopen.bmj.com/site/about/guidelines.xhtml

Summary Box

Strengths and limitations of this study

- To our knowledge, this is the first systematic review using reproducible methods to synthesise evidence about health expectancies and report quantitative comparisons of life and health expectancies to differentiate compression or expansion of morbidity.
- We used analyses published from 2016 to focus on the most contemporary evidence in trends.
- The quality of evidence was judged using criteria designed to assess threats to the validity of trends over time. However, due to the absence of methodological detail reported, it was not possible to give a clear judgement of study quality and bias for around half of the studies included in the review.

For peer review only

For peer review only - http://bmjopen.bmj.com/site/about/guidelines.xhtml

BACKGROUND

Populations are ageing worldwide. Globally, the proportion of those aged 65 and over has increased by 9 per cent in the last two decades, and is expected to grow by a further 16% by 2050.[1] This demographic shift will require societies to adapt. If longer lives are spent in poor health, governments face the challenge of providing accessible, high quality and sustainable long-term care.[2-4] The growth in life expectancy is a positive, but with this comes a responsibility to ensure people have the support they need as they age, and to facilitate ageing in place.[5]

In 2019, the World Health Organisation renewed its commitment to support countries to achieve longer and healthier lives with the Decade of Healthy Ageing 2020-2030 strategy.[6] A critical part of achieving longevity is understanding whether longer lives are typified by more years spent in good health (compression of disability) or poor health (expansion of disability). This has important implications for the provision of health and care services to respond to the needs of people as they age. It is therefore crucial to keep abreast of trends, specifically how the growth in life expectancy is matched by a growth in years spent in good health. Metrics to assess this most commonly include *healthy life expectancy* and *disability-free life expectancy*. Both provide an estimate of life expectancy spent in good health, but differ slightly with respect to their measurement. Healthy life expectancy tends to rely on single item questions of self-reported health, and is thus subject to fluctuations as expectations of health change over time.[7] Disability-free life expectancy is often calculated from multiple items about activity limitations and/or dependencies,[8] and therefore does not bear the same limitations as that of healthy life expectancy.

Previous reviews have summarised trends in total, healthy and disability-free life expectancy, the most recently in 2016.[8, 9] Typically, such evidence shows that whilst people are living longer, gains in life expectancy are not consistently matched by a growth in the number of years lived in good health and free of disability. Nevertheless, this is an evolving evidence base requiring ongoing scrutiny. Longitudinal datasets, which often form the bedrock of the analysis of these trends, are continually acquiring new data, whilst in the UK, the Office of National Statistics (ONS) updates and publishes trends yearly. Regardless, the pressing policy relevance of these issues globally warrants an updated overview of trends in health expectancies. Understanding these issues in a global context is equally

For peer review only - http://bmjopen.bmj.com/site/about/guidelines.xhtml

important in order to identify, and learn from, those countries whose populations are living healthier for longer. To obtain an up to date understanding of population trends in life expectancy and healthy ageing, we undertook a systematic review to a) synthesise evidence about trends in health expectancies in high-income countries, and b) assess whether such trends are keeping pace with total life expectancy.

METHODS

The methods used for this systematic review are reported according to the PRISMA checklist and guidance.[10]

Search strategy

A search strategy was developed using the concepts [*life expectancy*] AND [*trends* OR *impacting factors*]. The search was designed in MEDLINE using thesaurus headings and title, abstract and keyword field terms, and these elements translated to other databases. Electronic searches were carried out in MEDLINE (OVID) (1946 to September 19th 2019), Embase (OVID) 1980 to 2019 Week 38, Scopus (1966 to 22nd September 2019) and Health Management Information Consortium (OVID) (1979 to September 2019), in October 2019 (see the supplementary materials for the strategy applied to MEDLINE). As this systematic review updated previous reviews,[8, 9] searches were limited to studies published from 2016. The ONS website was also hand searched (November 2019) for reports published from 2016.

Review criteria

Review criteria are summarised in table 1. Studies were included if they examined trends (i.e. more than one time point) in health expectancies. Eligible health expectancies were general healthy life expectancy, disability free life expectancy, active life expectancy, health-related quality adjusted life expectancy, and health adjusted life expectancy. We did not include healthy cognitive life expectancy, dementia free life expectancy, or life expectancy with diseases. Studies must be conducted in an Organisation for Economic Co-operation and Development (OECD) high-income country.[11] OECD high-income countries were selected for this review for comparability. Studies reporting trends in life expectancy *only* were ineligible. Where studies also reported prevalence of disability, dependency or self-rated health alongside the above outcomes, these were reported for context. For ONS reports,

For peer review only - http://bmjopen.bmj.com/site/about/guidelines.xhtml

For peer review only

the most up-to-date analyses were included, with reports excluded if they were superseded by a more recently published analysis of the same data.

TABLE 1 HERE

Study selection

Titles and abstracts of all records were screened for relevance. The full texts of publications selected as relevant were retrieved and assessed for inclusion against the review criteria. Both stages of screening were undertaken independently by two researchers, with disagreements resolved through consensus.

Quality appraisal

Study quality and bias were assessed using an adapted version of previously published criteria for studies reporting trends.[12] This approach assesses ‘threats to the validity of comparisons over time’ (p. 3140). Following expert advice, we also judged two further criteria: whether the trend analysis used more than two time points, and the % of non-response in repeated cross-section surveys. Criteria were rated as *good*, *fair* or *poor*, according to the parameters summarised in table 2. Where study publications did not report the required information to assess quality, other associated publications (e.g. methodological and technical reports for the datasets used) were consulted, and we attempted to contact all authors and/or administrators of the datasets used. If the required information was not available from these sources, the criterion was assessed as *unclear*.

Using the assessments (good, fair, poor or unclear) for each criterion, studies were given a summary rating of quality. As studies were often based on summaries of cross-sectional data, they did not typically report information to assess the criterion *proportion lost to follow up.* We also struggled to find information to assess the criteria *proportion of proxy interviews* and *proportion of missing data.* We therefore based our summary judgement on four criteria: the comparability of methods over time, quality of the outcome measure, response rate or loss to follow up, and whether more than two time points were used. Studies with all four criteria rated good received a summary judgment of good. Studies with one or more criteria rated poor received a summary judgment of poor. The remainder were rated fair.

For peer review only - http://bmjopen.bmj.com/site/about/guidelines.xhtml

Where studies used multiple outcomes and were given different judgements for each, this was noted in the summary rating.

TABLE 2 HERE

Data extraction and synthesis

Study details (author, publication date, country, study design) and trend estimates (for each time point measured, and the change between the first and last time point) were extracted onto an Excel spreadsheet by one researcher. Fifty percent of studies were checked for accuracy by a second researcher. Where publications did not report the change between the first and last time point, we calculated this by subtracting the first time point estimate from the last time point estimate.

A narrative synthesis was used to summarise evidence on trends by outcome (e.g. healthy life expectancy, disability-free life expectancy, disability or dependency prevalence), supported by data summary tables. Estimates of changes in each health expectancy were compared to changes in life expectancy. This comparison provides evidence of whether there had been an expansion (health expectancy gains are smaller than life expectancy gains) or compression (health expectancy gains are equivalent to or greater than life expectancy gains) of disability and ill health.

Patient and public involvement

This review was requested by our funder within a timescale that did not allow for meaningful public and patient involvement.

FINDINGS

Twenty-eight studies met the review criteria (figure 1, and supplementary materials table 1). Seven studies reported trends in the UK,[7, 8, 13-17] including England,[7, 8, 14] England & Wales,[13, 15, 17] and each of the four devolved countries and the UK as a whole.[16] Two of the UK studies were ONS reports.[15, 16] The remaining studies reported trends in Belgium,[17-19] Canada,[20] Denmark,[21] Japan,[22, 23] the Netherlands,[24, 25] Norway,[26] Republic of Korea,[27, 28] Sweden,[29, 30] Switzerland,[31] and the US.[32-35] Three Global Burden of Disease studies were included, which reported trends across

For peer review only - http://bmjopen.bmj.com/site/about/guidelines.xhtml

For peer review only

multiple countries, and high income countries combined.[36-38] For these three studies, we used data for all high income countries combined.

The assessment of study quality is detailed in supplementary materials table 2. Three studies were rated good,[20, 34, 35] eleven were rated fair,[8, 13, 14, 18, 19, 21, 22, 24, 30, 32, 33], one was rated good *and* fair (as it used two outcome measures that each received a different quality rating),[7] and three were rated poor.[26, 29, 31] Ten studies were rated unclear due to a lack of information required to assess quality on the four key criteria.[15-17, 23, 25, 27, 28, 36-38] As majority large proportion of studies received a summary rating of unclear, the synthesis below does not prioritise evidence with a higher quality rating. Instead, the summary judgement is provided in supplementary materials table 2 for the reader's reference.

FIGURE 1 HERE

Across most studies, trends demonstrated a growth in health expectancies, with gains typically greater for men than women (supplementary materials tables 3a-d, 4a-f, 5a-d, 6a-c, 7a-b). Exceptions where changes were greater for women include the US (age 85),[33] Japan (birth),[23] Korea (age 65),[28] and Sweden (age 77).[30] One analysis of UK data over the more recent period of 2009/12 to 2015/17 also indicated that healthy life expectancy declined for women by 0.2 years.[15] Disability-free life expectancy at age 20 - 64 years also declined by 0.6 years between 1970 and 2010 for women in the US in one study.[33]

Is there evidence of an expansion or compression of poor health and disability?

Table 3 summarises the change in each life expectancy and health expectancy across trend periods for each study where comparisons were possible. The comparison does not include studies where: the metric of change was not comparable between life expectancy and health expectancy;[18, 20] trends were reported as a slope of index inequality only;[16] only forecasts were reported;[13, 14, 32] and life expectancy is reported only in a graph.[29, 34, 35]

For peer review only - http://bmjopen.bmj.com/site/about/guidelines.xhtml

TABLE 3 HERE

There is evidence from observed (i.e. not forecasted) trends in the UK that changes in health expectancies were smaller than those in life expectancy. This is consistent with evidence of a reduction in the proportion of life spent without disability in the UK. This pattern was also observed in: all high income countries combined in the Global Burden of Disease studies,[36-38] Norway,[26] Belgium,[19] Japan,[22, 23] and the US[33] for all, and in Switzerland [31] and Sweden [30] for men but not women. This points to evidence of an expansion of disability in a number of high-income countries, although not always consistently between men and women.

Gains in disability-free life expectancy were greater than gains in life expectancy for women in Sweden, a finding that was evident in two different datasets.[30] This indicates a compression of disability for women in Sweden. In another study, gains in life expectancy and healthy life expectancy were similar for women in Switzerland,[31] which might also suggest a compression of disability. However, this finding should be interpreted with some caution: the quality assessment highlighted a potential threat to the validity of the trend due to changes in the phrasing of, and response items for, the outcome measure between data waves.

Evidence from Korea and the Netherlands was less certain. Contrasting findings between studies gave an unclear picture about whether these countries had experienced a compression or expansion of poor health. For example, in one study from Korea, changes in quality adjusted life expectancy at birth were greater than changes in life expectancy.[27] Another study, using the same data but a different measure (healthy life expectancy), reported that gains at birth still lagged behind those for life expectancy, although gains in healthy life expectancy at age 65 and 85 were slightly higher than gains in life expectancy over the study period.[28] This contrast may reflect the difference in measures; Jo and colleagues suggest that quality adjusted life expectancy may be overestimated using the EQ-5D-3L, which forms the basis of this measure.[27]

Three studies from the Netherlands also offered inconsistent findings: this may reflect differences in the trend periods, age at which expectancies were estimated and the measures used.[17, 24, 25] For example, Reus-Pons and colleagues demonstrated gains in

For peer review only - http://bmjopen.bmj.com/site/about/guidelines.xhtml

healthy life expectancy were smaller than gains in total life expectancy at age 50, over a ten year period (2001-2011). Deeg and colleagues examined physical healthy life expectancy over a 23 year period (1993-2016): a decline was observed, whilst total life expectancy increased. Gheorghe and colleagues estimated quality adjusted life expectancy at age 25 and 65, and stratified by educational attainment. These trends indicated an expansion of poor health at age 25 and 65 for men at all levels of educational attainment, but a compression of poor health for women at age 25 (all levels of education) and 65 (high and low education).

DISCUSSION

This systematic review was undertaken to update our current understanding of trends in health expectancies in OECD high-income countries. The principal finding is that changes in health expectancies have not kept pace with the growth in life expectancy in a number of high income countries. One clear exception was Sweden, where gains in women's disability-free life expectancy were greater than gains in life expectancy over a period of almost 20 years. This was a finding evidenced from two different datasets within Sundberg and colleagues' study (SWEOLD: 1992-2011; SHARE: 2004-2011). This finding contrasts with an earlier review, which found evidence of a compression of disability for men, but not women, in Sweden.[9] This has now been reversed with an expansion of disability for men.[30] Sundberg and colleagues attribute the compression of disability for women to improved health for women but not men, whilst men were living longer due to falling deaths from cardiovascular disease.

The equivalence of total and healthy life expectancy for women in Switzerland also indicates a compression of poor health in Switzerland, although as noted earlier, this finding should be interpreted with some caution. Nevertheless, based on the countries with data, our findings suggest that countries still need to make significant progress to achieve the World Health Organisation's Decade of Healthy Ageing goal of healthier, longer lives for all.

The observed expansion of disability and poor health has important implications for older people's health and care. Across many high-income countries, care services that support older people's day to day independence and quality of life are not universal but subject to payment barriers.[3, 4] Living longer with greater disability and in poor health signals a

For peer review only - http://bmjopen.bmj.com/site/about/guidelines.xhtml

need for greater policy focus on ensuring older people have timely access to care. This is a particularly timely message for policy makers in the UK, as the All Party Parliamentary Group for Longevity set out recommendations to achieve the UK Government's goal of extending healthy life by 2035 and reducing inequalities.[39] Furthermore, the finding that women are living with longer periods of disability may warrant a particular focus on how services can support women in later life. This is especially important given that women are more likely than men to experience financial insecurity in later life,[40] and thus may face greater barriers to paid-for care services.

Finally, given the high death rates in some countries as a result of the COVID-19 pandemic, and the likely long-term health consequences of the virus that are becoming apparent, further scrutiny of future trends in health expectancies is needed. A recent analysis of UK mortality data in 2020, for example, indicates that COVID has reduced average life expectancy by around a year.[41]

Strengths and limitations

This systematic review provides a robust picture of trends in health expectancies in high-income countries. Although previous reviews have been published,[42] a strength of our study is that we used reproducible systematic search techniques and reported quantitative comparisons to differentiate the compression or expansion of morbidity.

We chose to focus on evidence published from 2016, in order to avoid duplication of previous work and focus on contemporary evidence. A limitation of this approach is that we have omitted potentially useful studies published prior to 2016. However, this criterion has enabled a synthesis that prioritises the most recent evidence on trends in health expectancies. In addition to searching for published studies, we searched the Office for National Statistics website, the UK government body responsible for analysing data about the UK population. We did not search the equivalent national statistical bodies for every other high income country as the volume of translation this would necessitate was unfeasible. However, this is not a major shortcoming: most of the non-UK studies used nationally representative samples, and none indicated unusual or contradictory trends that required verification from other data sources.

For peer review only

A key limitation of our synthesis is that whilst we were able to make some broad comparisons between studies, a more detailed comparison of trends was not possible. This was due to the high degree of heterogeneity between studies in: the age at which the health expectancy is estimated, trend periods (years and time frame), the measures used, and the way trends were stratified. This not only prevented a more meaningful comparison, but also limited the extent to which we were able explain differences in these trends between studies and countries. A further consideration here is the comparability of the overlapping and non-overlapping trend periods within and between countries in the studies included in this review (supplementary materials figure 1). Different countries face national challenges at different times, whilst global challenges can differentially affect countries across the same time periods. Therefore, even similar trends across the same time periods may be due to different reasons.

Finally, the quality of this evidence was judged using criteria designed to assess threats to the validity of trends and the adequacy of the outcome measure.[12] For some studies, it was not possible to arrive at a clear judgement due to the absence of required methodological detail regarding survey response rates. This is an important limitation, but one that is easily addressed if future studies of health expectancy trends report the methodological detail required. Where a judgement on quality was possible, studies were generally of fair or good quality, signalling no major concerns to the validity of the evidence synthesised. The rating of poor for a minority of studies largely reflected a change in the outcome measure over time,[26, 29, 31] which may reduce comparability between observed time points. To some extent, such changes are expected as panel and longitudinal studies evolve at each new point of data collection. Such reduced comparability may undermine the validity of the trends reported in these studies. However, this should be balanced against the judgements for the other quality assessment criteria, where methods were rated favourably and indicated minimal bias (see supplementary materials). Overall, for studies where a clear judgment on quality was possible, we did not identify any studies that were particularly concerning in terms of quality and validity.

CONCLUSIONS

In a number of high-income countries, changes in health expectancies over time have not kept pace with the growth in life expectancy. That is, people are living longer but disability

For peer review only - http://bmjopen.bmj.com/site/about/guidelines.xhtml

For peer review only

and poor health are occupying an increasing proportion of later life. A compression of disability for women in Sweden signals some progress in achieving healthier longer lives. These findings have implications for health and care related policy, and in particular ensuring people have timely access to care in later life. Further scrutiny of health expectancies in the wake of the COVID-19 pandemic is necessary given the high death rates in some countries and the long-term health consequences of the virus that are becoming apparent.

For peer review only

For peer review only - http://bmjopen.bmj.com/site/about/guidelines.xhtml

Funding acknowledgement

National Institute for Health Research (NIHR) Policy Research Programme conducted through the NIHR Older People and Frailty Policy Research Unit, PR-PRU-1217-21502. The views expressed are those of the authors and not necessarily those of the NIHR or the Department of Health and Social Care.

Author contributions

Author contributions: CJ planned the study, developed the protocol, and co-wrote the paper; GS developed the protocol, contributed to all stages of the review, and co-wrote the paper; PK developed the protocol, contributed to all stages of the review, and co-wrote the paper, FB developed the protocol, developed and undertook the searches, and co-wrote the paper; DC developed the protocol, oversaw all stages of the review, and co-wrote the paper; BH developed the protocol and co-wrote the paper.

Data availability statement

All data relevant to the study are included in the article or uploaded as supplementary information.

Competing interests: None declared.

For peer review only - http://bmjopen.bmj.com/site/about/guidelines.xhtml

REFERENCES

1 Department of Social and Economic Affairs. World Population Ageing 2019 Highlights. *United Nations* 2019.
2 Organisation for Economic and Co-Operative Development. A Good Life in Old Age? Monitoring and Improving Quality in Long-Term Care. 2013.
3 Organisation for Economic Co-operative Development. Help Wanted? Providing and Paying for Long-Term Care. Organisation for Economic and Co-operative Development 2011.
4 Organisation for Economic Co-operative Development. Public long-term care financing arrangements in OECD countries. In: Oecd, ed. *Help Wanted?*: OECD 2011.
5 Pani-Harreman KE, Bours GJJW, Zander I*, et al.* Definitions, key themes and aspects of 'ageing in place': a scoping review. *Ageing and Society* 2020:1-34.
6 World Health Organisation. Decade of Healthy Ageing 2020-2030. 2019.
7 Jagger C, Matthews FE, Wohland P*, et al.* A comparison of health expectancies over two decades in England: results of the Cognitive Function and Ageing Study I and II. *Lancet* 2016;**387**:779-86.
8 Kingston A, Wohland P, Wittenberg R*, et al.* Is late-life dependency increasing or not? A comparison of the Cognitive Function and Ageing Studies (CFAS). *Lancet* 2017;**390**:1676-84.
9 Jagger C. Trends in life expectancy and healthy life expectancy. Newcastle University Institute for Ageing and Institute of Health & Society, Government Office for Science 2015.
10 Moher D, Liberati A, Tetzlaff J*, et al.* Preferred Reporting Items for Systematic Reviews and Meta-Analyses: The PRISMA Statement. *PLoS Medicine* 2009;**6**:e1000097.
11 Group WB. Fact Sheet: OECD High-Income. *Doing Business 2019*: World Bank 2019.
12 Freedman VA, Martin LG, Schoeni RF. Recent trends in disability and functioning among older adults in the United States: a systematic review. *Jama* 2002;**288**:3137-46.
13 Guzman-Castillo M, Ahmadi-Abhari S, Bandosz P*, et al.* Forecasted trends in disability and life expectancy in England and Wales up to 2025: a modelling study. *The Lancet Public Health* 2017;**2**:e307-e13.
14 Kingston A, Comas-Herrera A, Jagger C*, et al.* Forecasting the care needs of the older population in England over the next 20 years: estimates from the Population Ageing and Care Simulation (PACSim) modelling study. *Lancet Public Health* 2018;**3**:e447-e55.
15 Office for National Statistics. Health state life expectancies, UK: 2015 to 2017. Office for National Statistics 2018.
16 Office for National Statistics. Health state life expectancies by national deprivation deciles, England and Wales: 2015 to 2017. Office for National Statistics 2019.
17 Reus-Pons M, Kibele EUB, Janssen F. Differences in healthy life expectancy between older migrants and non-migrants in three European countries over time. *International Journal of Public Health* 2017;**62**:531-40.
18 Renard F, Devleesschauwer B, Van Oyen H*, et al.* Evolution of educational inequalities in life and health expectancies at 25 years in Belgium between 2001 and 2011: a census-based study. *Arch Public Health* 2019;**77**:6.
19 Yokota RTC, Nusselder WJ, Robine JM*, et al.* Contribution of chronic conditions to gender disparities in health expectancies in Belgium, 2001, 2004 and 2008. *Eur J Public Health* 2019;**29**:82-7.
20 Steensma C, Loukine L, Choi BC. Evaluating compression or expansion of morbidity in Canada: trends in life expectancy and health-adjusted life expectancy from 1994 to 2010. *Health Promot Chronic Dis Prev Can* 2017;**37**:68-76.
21 Bronnum-Hansen H, Eriksen ML, Andersen-Ranberg K*, et al.* Persistent social inequality in life expectancy and disability-free life expectancy: Outlook for a differential pension age in Denmark? *Scand J Public Health* 2017;**45**:459-62.
22 Sugawara YM, Saito Y. Changes in disability-free life expectancy (DFLE) at birth between 2000 and 2010 across Japanese prefectures. *International Journal of Public Health* 2016;**61**:739-49.

For peer review only - http://bmjopen.bmj.com/site/about/guidelines.xhtml

23 Tokudome S, Hashimoto S, Igata A. Life expectancy and healthy life expectancy of Japan: the fastest graying society in the world. *BMC research notes* 2016;**9**:482.
24 Deeg DJH, Comijs HC, Hoogendijk EO*, et al.* 23-Year Trends in Life Expectancy in Good and Poor Physical and Cognitive Health at Age 65 Years in the Netherlands, 1993-2016. *Am J Public Health* 2018;**108**:1652-8.
25 Gheorghe M, Wubulihasimu P, Peters F*, et al.* Health inequalities in the Netherlands: trends in quality-adjusted life expectancy (QALE) by educational level. *Eur J Public Health* 2016;**26**:794-9.
26 Storeng SH, Krokstad S, Westin S*, et al.* Decennial trends and inequalities in healthy life expectancy: The HUNT Study, Norway. *Scand J Public Health* 2018;**46**:124-31.
27 Jo MW, Seo W, Lim SY*, et al.* The Trends in Health Life Expectancy in Korea according to Age, Gender, Education Level, and Subregion: Using Quality-Adjusted Life Expectancy Method. *J Korean Med Sci* 2019;**34**:e88.
28 Lee JY, Ock M, Kim SH*, et al.* Health-Adjusted Life Expectancy (HALE) in Korea: 2005-2011. *J Korean Med Sci* 2016;**31 Suppl 2**:S139-S45.
29 Lagergren M, Johnell K, Schon P*, et al.* Towards a postponement of activities of daily living dependence and mobility limitations: Trends in healthy life years in old age in Sweden. *Scand J Public Health* 2017;**45**:520-7.
30 Sundberg L, Agahi N, Fritzell J*, et al.* Trends in health expectancies among the oldest old in Sweden, 1992-2011. *Eur J Public Health* 2016;**26**:1069-74.
31 Remund A, Cullati S, Sieber S*, et al.* Longer and healthier lives for all? Successes and failures of a universal consumer-driven healthcare system, Switzerland, 1990–2014. *International Journal of Public Health* 2019.
32 Cao B. Future healthy life expectancy among older adults in the US: a forecast based on cohort smoking and obesity history. *Popul Health Metr* 2016;**14**:23.
33 Crimmins EM, Zhang Y, Saito Y. Trends Over 4 Decades in Disability-Free Life Expectancy in the United States. *Am J Public Health* 2016;**106**:1287-93.
34 Freedman VA, Spillman BC. Active life expectancy in the older US population, 1982-2011 : differences between blacks and whites persisted. 2016.
35 Freedman VA, Wolf DA, Spillman BC. Disability-Free Life Expectancy Over 30 Years: A Growing Female Disadvantage in the US Population. *Am J Public Health* 2016;**106**:1079-85.
36 Global Burden of Disease study, Collaborators H. Global, regional, and national disability-adjusted life-years (DALYs) for 359 diseases and injuries and healthy life expectancy (HALE) for 195 countries and territories, 1990-2017: a systematic analysis for the Global Burden of Disease Study 2017. *Lancet* 2018;**392**:1859-922.
37 Hay SI, Abajobir AA, Abate KH*, et al.* Global, regional, and national disability-adjusted life-years (DALYs) for 333 diseases and injuries and healthy life expectancy (HALE) for 195 countries and territories, 1990-2016: A systematic analysis for the Global Burden of Disease Study 2016. *The Lancet* 2017;**390**:1260-344.
38 Kassebaum NJ, Arora M, Barber RM*, et al.* Global, regional, and national disability-adjusted life-years (DALYs) for 315 diseases and injuries and healthy life expectancy (HALE), 1990–2015: a systematic analysis for the Global Burden of Disease Study 2015. *The Lancet* 2016;**388**:1603-58.
39 All Party Parliamentary Group for Longevity. The Health of the Nation: A Strategy for Healthier Longer Lives. All Party Parliamentary Group for Longevity, 2020.
40 Scharf T, Shaw C. Inequalities in later life. Centre for Ageing Better 2017.
41 Aburto JM, Kashyap R, Schöley J*, et al.* Estimating the burden of the COVID-19 pandemic on mortality, life expectancy and lifespan inequality in England and Wales: a population-level analysis. *Journal of Epidemiology and Community Health* 2021:jech-2020-215505.
42 J-M R, C J, Crimmins E.M ea. Trends in health expectancies. In: Jagger C CE, Saito Y, et al, ed. *International Handbook of Health Expectancies*. London: Springer 2020.

For peer review only - http://bmjopen.bmj.com/site/about/guidelines.xhtml

For peer review only

For peer review only - http://bmjopen.bmj.com/site/about/guidelines.xhtml

Table 1. Review criteria

**Population**	Studies must examine, trends from birth, 65 years and 85 years. Studies reporting trends from other ages were also reviewed where evidence was available. Studies must examine these trends in whole populations. Studies reporting trends in population sub-groups only (i.e. only those with heart failure) were ineligible.
**Exposure**	As this review reports evidence on life expectancy, health expectancy trends, an exposure variable was not required.
**Comparator**	Not applicable.
**Outcome(s)**	Active life expectancy, healthy life expectancy, disability-free life expectancy, health-related quality adjusted life expectancy, health-adjusted life expectancy. Studies reporting *only* life expectancy trends were ineligible. Studies must examine changes in these outcomes over time (i.e. include more than one time point). Studies that report projections/forecasts of these outcomes were also eligible.
**Study design**	Studies must use an observational design and be carried out in an OECD high-income country. The review focused on evidence from the UK with comparison to evidence from other OECD high-income countries where possible. Studies published from 2016 were eligible. ONS reports were excluded if they were not the latest release, or reported trends for a period contained within a more recent ONS publication using the same data.

For peer review only

For peer review only - http://bmjopen.bmj.com/site/about/guidelines.xhtml

Table 2. Quality assessment criteria

Criteria	Parameters
**Comparability of interview methods between time points**	Good: Identical<br>Fair: Change in mode<br>Poor: Change in disability, functioning or health outcomes
**Quality of outcome measure**	Good: Detailed multiple item measure<br>Fair: Single item global measure<br>*No criteria for poor*
**Uses more than two time points to assess trend[b]**	Good: Uses more than two time points<br>Fair: Uses only two time points<br>*No criteria for poor*
**% Response in repeated cross-sectional studies[a]**	Good: >70% response rate and <10% drop in subsequent surveys<br>Fair: <70% or >10% drop in subsequent surveys<br>*No criteria for poor*
**Loss to follow up**	Good: NA or <5%<br>Fair: 5-10%<br>Poor: >10%<br>Note: This only applies to longitudinal study designs (i.e. not independent repeat cross sections)
**Proportion of proxy interviews**	Good: <10%<br>Fair: 10-20%<br>Poor: >20%
**Proportion of missing data**	Good: <5%<br>Fair: 5-10%<br>Poor: >10%

[a] Added to the quality assessment following expert advice

For peer review only - http://bmjopen.bmj.com/site/about/guidelines.xhtml

Table 3. Change in life expectancy, healthy life expectance and disability-free life expectancy across all studies where reported[a]

Study	Country	Age	Trend period	Change in LE	Change in HLE	Change in DFLE
All high-income countries combined from the Global Burden of Disease studies						
GBD study 2016	All high income	0	2005, 2015	Men: 1.75 Women: 1.33	Men: 1.43 Women: 1.08	-
GBD study 2017	All high income	0	1990, 2016	Men: 5.63 Women: 4.14	Men: 4.43 Women:3.21	-
		65		Men: 3.51 Women: 3.03	Men:2.27 Women: 2.6	-
GBD study 2018	All high income	0	1990, 2017	Men: 5.6 Women: 4.2	Men: 4.2 Women: 3.0	-
Studies with samples from Europe						
Jagger 2016	England	65	1991, 2011	Men: 4·5 Women: 3·6	Men: 3·8 (3·5 to 4·1) Women: 3·1 (2·7 to 3·4)	Men: 2.6 (2.3, 2.9) Women: 0.5 (0.2, 0.9)
Kingston 2017	England	65	1991, 2011	Men: 4.7 Women: 4.1		Men: 1.7 (1.2, 2.1) Women: 0.2 (-0.4, 0.7)
Reus-Pons 2017	England & Wales	50	2001, 2011	Men: 2.8 Women: 2.2	Men: 0.25 Women: -0.15	-
ONS 2018	UK	0	2009/11 – 2015/17	Men: 0.8 Women: 0.4	Men: 0.4 Women: -0.2	-
Bronnum-Hansen 2017	Denmark	65	2006/07, 2010/11,2013/14	Difference in change in LE between high and low education: Men: 0.1 Women: 0.3	-	Difference in change in DFLE between high and low education Men: -0.3 Women: -0.3
Deeg 2018	Netherlands	65	1993, 1996, 1999, 2002, 2006, 2009, 2012, 2016	Men: 4.0 Women: 2.2	Physical Men: -2.2 Women: -1.5	-
Gheorghe 2016	Netherlands	25	2001, 2011	Men: High: 2.88 Med: 3.22 Low: 2.43 Women: High: 1.78 Med: 1.53 Low: 1.15	Men: High 2.85 Med: 3.01 Low: 2.13 Women: High: 2.64 Med: 1.57 Low: 1.80	-
		65		Men: High: 2.48	Men: High: 2.17	-

For peer review only - http://bmjopen.bmj.com/site/about/guidelines.xhtml

				Med: 2.24<br>Low: 1.68<br><br>Women:<br>High: 1.64<br>Med: 1.41<br>Low: 1.12	Med: 1.91<br>Low: 1.37<br><br>Women:<br>High: 1.74<br>Med: 1.21<br>Low: 1.14	
Reus-Pons 2017	Netherlands	50	2001, 2011	Men: 2.82<br>Women: 1.84	Men: 2.21<br>Women: 1.25	-
Remund 2019	Switzerland	30	1990/94, 1995/99, 2004/04, 2010/14	Men: 5.02<br>Women: 3.09	Men: 4.52<br>Women: 3.09	-
Storeng 2018	Norway	50	1984/86, 1995/97, 2006/08	Men: 6.99 (5.27–8.72)<br>Women: 6.75 (5.16–8.34)	Men: 6.90 (6.08–7.73)<br>Women: 5.40 (4.56–6.25)	Men: 2.71 (2.01–3.42)<br>Women: 0.33 (–0.40–1.06)
Sundberg 2016	Sweden (SWEOLD)	77	1992, 2002, 2004, 2011	Men: 1.7<br>Women: 1.1	-	Men: 1.1<br>Women: 1.6
	Sweden (SHARE)		2004, 2011	Men: 0.6<br>Women: 0.4	-	Men: 0.1<br>Women: 1.3
Yokota 2019	Belgium	15	2001, 2004, 2008	Men: 1.6<br>Women: 1.0	-	Men: 0.7<br>Women: -0.7
Studies with samples from Asia						
Jo 2019	R. Korea	0	2005, 2007, 2008, 2009, 2010, 2011, 2012, 2013	Men: 3.38<br>Women: 3.15	Men: 4.03<br>Women: 3.44	-
Lee 2016	R. Korea	0	2005, 2008, 2011	Men: 2.5<br>Women: 2.6	Men: 1.4[b]<br>Women: 1.2[b]	-
		65		Men: 1.6<br>Women: 2.0	Men: 2.2[b]<br>Women: 2.6[b]	-
		85		Men: 0.3<br>Women: 0.7	Men: 1.4[b]<br>Women: 1.4[b]	-
Sugawara 2016	Japan	0	2000, 2010	Men: 1.9<br>Women:1.7	-	Men: 1.0<br>Women: 0.4
Tokudome 2016	Japan	0	1990, 1995, 2000, 2005, 2010, 2013	Men: 4.01<br>Women: 4.43	Men: 3.02<br>Women: 3.32	-
Studies with sample from North America						
Crimmins 2016	US	0	1970, 1980, 1990, 2000, 2010	Men: 9.2<br>Women: 6.4	-	Men: 4.5<br>Women: 2.7
		20-64		Men: 1.8<br>Women: 0.9	-	Men: 0.9<br>Women: -0.6
		65		Men: 4.7<br>Women: 3.5	-	Men: 2.7<br>Women: 2.4
		85		Men: 1.1<br>Women: 1.3	-	Men: 0.5<br>Women: 0.8

[a]Table and comparison does not include studies where: the metric of change was not comparable for each health expectancy (Steensma et al. 2017); trends were reported as slope of index inequality only (ONS 2019); only forecasts are reported (Kingston et al., 2018, Guzman-Castillo et al., 2017, Cao et al., 2016); total life expectancy is reported only as a graph (Lagergren et al., 2017, Freedman 2016a,b); trends are reported as difference in change in DFLE between levels of education and not comparable to the LE trend (Renard 2015).

For peer review only - http://bmjopen.bmj.com/site/about/guidelines.xhtml

Figure legends:

Figure 1. PRISMA Flowchart

Supplementary Materials Figure 1. Expansion and compression of poor health/disability by country and time period

For peer review only

For peer review only - http://bmjopen.bmj.com/site/about/guidelines.xhtml

Figure 1. PRISMA diagram

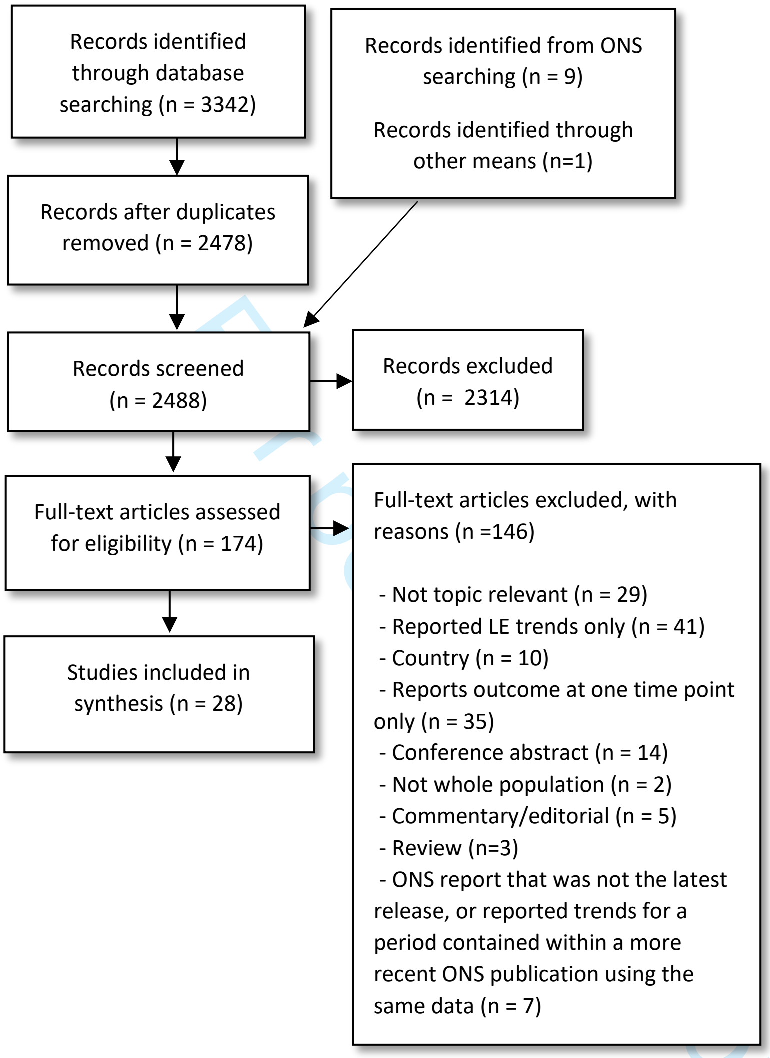

Supplementary Materials

Table 1. Summary of included studies

Study	Country	Data source	Trend years	Trends reported	Disability/health measured from a global (single) item or multiple items?[a]	Stratification	At age	Method used to calculate life expectancy
High income countries combined								
Global Burden of Disease study 2016 [b]	High income countries combined	Global Burden of Disease Study	2005, 2015	LE, HLE	Multiple	Sex	0	Sullivan
Global Burden of Disease study 2017 [b]	High income countries combined	Global Burden of Disease Study	1990, 2016	LE, HLE	Multiple	Sex	0, 65	Sullivan
Global Burden of Disease study 2018 [b]	High income countries combined	Global Burden of Disease Study	1990, 2017	LE, HLE	Multiple	Sex	0	Sullivan
Studies with samples from OECD high-income countries in Europe								
Jagger 2016	England	CFAS I and II	1991, 2011	LE, HLE, DFLE, t % of LE without disability, % of life spent healthy, disability prevalence	HLE: global DFLE: multiple	Sex	65	Sullivan
Kingston 2017	England	CFAS I and II	1991, 2011, and 2015, 2025, 2035 (projection)	LE, DFLE, % of LE without disability, disability prevalence	Multiple	Sex	65	Sullivan

For peer review only - http://bmjopen.bmj.com/site/about/guidelines.xhtml

Study	Country	Data source	Trend years	Trends reported	Disability/health measured from a global (single) item or multiple items?[a]	Stratification	At age	Method used to calculate life expectancy
Kingston 2018	England	CFAS II, ELSA, Understanding Society	2015, 2025, 2035 (projection)	LE, DFLE, % of LE without disability, disability prevalence	Multiple	Sex, age	65-74, 75-84, 85	Sullivan
Guzman-Castillo 2017	England, Wales	ELSA	2015, 2025 (projection)	LE, DFLE, disability prevalence	Multiple	None	65	Sullivan
Reus-Pons 2017	England & Wales	Census, national death registries, Permanent Survey on the Living Situation (POLS), Health Survey	2001, 2011	LE, HLE	Global	Sex, migrant/non-migrant	50	Sullivan
ONS 2019	England & Wales	UK Census, Annual Population Survey, Indices of Multiple Deprivation 2015, Welsh Index of Multiple Deprivation 2014	2012/14 – 2015/17	LE, HLE	Global	Sex, deprivation decile[c]	0, 65	Sullivan
ONS 2018	UK, England, Wales, Northern Ireland, Scotland	UK Census, Annual Population Survey	2009/11 – 2015/17	LE, HLE	Global	Sex	0	Sullivan
Bronnum-Hansen 2017a	Denmark	Danish Surveys of Health, Ageing and Retirement in Europe	2006/07, 2010/11,2013/14	LE, DFLE,	Global	Sex, education	65	Sullivan
Deeg 2018	Netherlands	Longitudinal Aging Study Amsterdam	1993, 1996, 1999, 2002, 2006, 2009, 2012, 2016	LE, HLE, % of life spent healthy	Multiple	Sex	65	Sullivan
Gheorghe 2016	Netherlands	Dutch Labour force Survey and National Mortality Registry	2001, 2011	LE, Quality Adjusted Life Expectancy),	Multiple	Sex, education	25, 65	Sullivan

For peer review only - http://bmjopen.bmj.com/site/about/guidelines.xhtml

Study	Country	Data source	Trend years	Trends reported	Disability/health measured from a global (single) item or multiple items?[a]	Stratification	At age	Method used to calculate life expectancy
Lagergren 2017	Sweden	National mortality statistics, Nationwide Swedish Surveys of Living Conditions	1980/1985, 1994/95, 2006/2011	LE (graph only), DFLE, disability prevalence	Multiple	Sex	65	Sullivan
Remund 2019	Switzerland	Swiss National Cohort, Swiss Health Interview Survey	1990/94, 1995/99, 2004/04, 2010/14	LE, HLE,	Global	Sex, education	30	Sullivan
Renard 2019	Belgium	Census, National Registry	2001, 2011	LE, DFLE,	Global	Sex, education	25	Sullivan
Reus-Pons 2017	Netherlands	Census, national death registries, Permanent Survey on the Living Situation (POLS) and Health Survey	2001, 2011	LE, HLE,	Global	Sex	50	Sullivan
Storeng 2018	Norway	HUNT Study, Norwegian Education Database	1984/86, 1995/97, 2006/08	LE, HLE, DFLE,	HLE & DFLE: Global	Sex, education	30	Sullivan
Sundberg 2016	Sweden	National mortality statistics, Swedish Panel Study of Living Conditions of the Oldest Old (SWEOLD), Survey of Health, Ageing and Retirement in Europe (SHARE)	1992, 2002, 2004, 2011	LE, DFLE,	Multiple	Sex	77	Sullivan
Yokota 2019	Belgium	Health Interview Survey, National Statistics	2001, 2004, 2008	LE, DFLE,	Multiple	Sex	15	Sullivan
Studies with samples from OECD high-income countries in Asia								
Jo 2019	R. Korea	Korean Statistical Information Service, Korea National Health and Nutrition Examination Survey	2005, 2007, 2008, 2009, 2010, 2011, 2012, 2013	LE, HLE,	Multiple	Sex	0	Sullivan
Lee 2016	R. Korea	Korean Statistical Information Service,	2005, 2008, 2011	LE, HLE,	Multiple	Sex	0-100 at five year	Sullivan

For peer review only - http://bmjopen.bmj.com/site/about/guidelines.xhtml

Study	Country	Data source	Trend years	Trends reported	Disability/health measured from a global (single) item or multiple items?[a]	Stratification	At age	Method used to calculate life expectancy
		Korea National Health and Nutrition Examination Survey					intervals; ages 0, 65 and 85 reported here	
Sugawara 2016	Japan	Census, Comprehensive Survey of Living Conditions of the People on Health and Welfare	2000, 2010	LE, DFLE, % life without disability	Multiple	None	0	Sullivan
Tokudome 2016	Japan	Global Burden of Disease Study 2013	1990, 1995, 2000, 2005, 2010, 2013	LE, HLE,	Multiple	Sex	0	Sullivan (based on GBD estimates)
Studies with samples from OECD high-income countries in North America								
Cao 2016	US	Human Mortality Database, US National Health Interview Survey (NHIS)	1982, 2010, 2040 (projection)	LE, DFLE,	Global	Sex	55-85	Multi-state life table
Crimmins 2016	US	Census, US National Vital Statistics, National Health Interview	1970, 1980, 1990, 2000, 2010	LE, DFLE, % life without disability, disability prevalence	Global	Sex	0, 20-64, 65, 85	Sullivan
Freedman 2016a	US	National Long-Term Care Survey, National Health and Aging Trends Study	1982, 2004, 2011	LE (graph only), DFLE, prevalence	Multiple	Sex	65, 85	Sullivan
Freedman 2016b	US	National Long-Term Care Survey, National Health and Aging Trends Study	1982, 2004, 2011	LE (graph only), Active Life Expectancy	Multiple	Sex, Race	65, 85	Sullivan
Steensma 2017	Canada	Statistics Canada, National Population Health Survey, Canadian Community Health Survey	1994, , 1994/95, 1996/97, 1998/99, 2000/01 2003, 2005, 2009/10	LE, HLE	Multiple	Sex	0, 20, 65	Sullivan

For peer review only - http://bmjopen.bmj.com/site/about/guidelines.xhtml

[a]Global item measures include self-rated health, the Global Limitation Activity Indicator, or other single item measures about having a limitation; [b]Disability adjusted life years reported as global estimates (i.e. not country specific) and were thus not included here; [c]Healthy life expectancy trends are not reported by deprivation deciles, but as a slope index of inequality between the most and least deprived deciles

For peer review only

For peer review only - http://bmjopen.bmj.com/site/about/guidelines.xhtml

Table 2. Quality assessment

Study	Comparability of interview methods	Outcome measure	Does the trend consider more than 2 time points?	% Response rate in cross-sectional surveys or loss to follow up in longitudinal studies	Proxy %	Missing data %	Other publications consulted	SUMMARY JUDGMENT
Bronnum-Hansen 2017	FAIR<br>Change in items across waves, but disability question did not change across waves 1-7.	Disability: FAIR (Single item question - Global Activity Limitation Indicator)	GOOD: Yes	FAIR<br><br>Response rates:<br><br>2006/07: 69.0%<br>2011/12: 61.5%<br>2013/14: 61.0%),	UNCLEAR	UNCLEAR	SHARE methods report: http://www.share-project.org/fileadmin/pdf_documentation/Methodology/Methodology_2005.pdf	FAIR
Cao 2016	FAIR<br>Not reported, but in Freedman et al. (2002), the NHIS (which is the survey used in Cao et al.), they note the sampling frame was redesigned in 1995. A change in disability items is reported, but this study used a different set of items to ensure disability is comparable across waves.	Disability: FAIR<br>Although this measure draws upon multiple ADLs, the question is whether the participant has any limitation in any of the ADLs, and so is a single item measure.	GOOD: Yes (third time point is projection)	GOOD<br>Annual response rate approximately 80%	UNCLEAR	UNCLEAR	Freedman et al. (2002)<br><br>https://www.cms.gov/About-CMS/Agency-Information/OMH/resource-center/hcps-and-researchers/data-tools/sgm-clearinghouse/nhis	FAIR
Crimmins 2016	FAIR<br>Not reported, but in Freedman et al. (2002), the NHIS (which is the survey used in Crimmins et al), they note the sampling frame was redesigned in 1995. A change in disability items is	Disability: FAIR<br>Although question draws upon multiple ADLs, the question is whether the participant has any limitation in any of the ADLs,	GOOD: Yes	GOOD<br>Annual response rate approximately 80%	UNCLEAR	UNCLEAR	Freedman et al. (2002)	FAIR

For peer review only - http://bmjopen.bmj.com/site/about/guidelines.xhtml

Study	Comparability of interview methods	Outcome measure	Does the trend consider more than 2 time points?	% Response rate in cross-sectional surveys or loss to follow up in longitudinal studies	Proxy %	Missing data %	Other publications consulted	SUMMARY JUDGMENT
	reported, but study uses different set of items to ensure disability is comparable across waves.	and so is a single item measure.						
Deeg 2018	GOOD	Disability: GOOD	GOOD: Yes	FAIR<br><br>Response rates:<br><br>1992/93: 81.7%<br>1995/96: 66.9%<br>1998/99: 54.6%<br>2001/02: 44.4% (cohort 1 only, replenished in 2002/2003)<br>2005/06: 33.0% (cohort 1) and : 90.6% (cohort 2)<br>2008/09: 25.9% (cohort 1) and 83.1% (cohort 2)<br>2011/12: 20.1% (cohort 1) and 75.7% (cohort 2)<br>2015/16: 13.1% (cohort 1) and 67.0% (cohort 2) and 83.4% (cohort 3)	GOOD<br>From publication appendix: "Across the study period, the average use of proxies was 2% for respondents in good physical health, 3% for those in fair physical health, and 8% for those in poor physical health."	GOOD<br>Across waves:<br><br>Men: 3.3%<br>Women: 4.4%	https://www.ncbi.nlm.nih.gov/pmc/articles/PMC5010587/<br><br>https://link.springer.com/article/10.1007/s10654-019-00541-2	FAIR
Freedman 2016a	GOOD	Disability: GOOD	GOOD: Yes	GOOD<br><br>Response rates:<br><br>1982: 87.3%<br>2004: 80.6%<br>2011: (NHATS round 1) 70.9%	POOR<br>1982: 20.2%<br>2011: 7.2%	GOOD<br>1982: 0%<br>2011: 2.1%	https://aspe.hhs.gov/basic-report/introduction-national-long-term-care-survey<br><br>https://www.nhats.org/sites/default/files/2021-01/NHATS_User_Guide_R9_Final_Release_0.pdf<br><br>https://www.ncbi.nlm.nih.gov/pmc/articles/PMC6934030/	GOOD

For peer review only - http://bmjopen.bmj.com/site/about/guidelines.xhtml

Study	Comparability of interview methods	Outcome measure	Does the trend consider more than 2 time points?	% Response rate in cross-sectional surveys or loss to follow up in longitudinal studies	Proxy %	Missing data %	Other publications consulted	SUMMARY JUDGMENT
Freedman 2016b	GOOD	Disability: GOOD	GOOD: Yes	GOOD<br><br>Response rates:<br><br>1982: 87.3%<br>2004: 80.6%<br>2011: (NHATS round 1) 70.9%	POOR<br>1982: 20.2%<br>2011: 7.2%	GOOD<br>1982: 0%<br>2011: 2.1%	https://aspe.hhs.gov/basic-report/introduction-national-long-term-care-survey<br><br>https://www.nhats.org/sites/default/files/2021-01/NHATS_User_Guide_R9_Final_Release_0.pdf<br><br>https://www.ncbi.nlm.nih.gov/pmc/articles/PMC6934030/	GOOD
GBD 2016 DALYs and HALE Collaborators	UNCLEAR<br>Not reported.	Disability: GOOD	FAIR: No	UNCLEAR	UNCLEAR	UNCLEAR		UNCLEAR
GBD 2017 DALYs and HALE Collaborators	UNCLEAR<br>Not reported.	Disability: GOOD	FAIR: No	UNCLEAR	UNCLEAR	UNCLEAR		UNCLEAR
GBD 2018 DALYs and HALE Collaborators	UNCLEAR<br>Not reported.	Disability: GOOD	FAIR: No	UNCLEAR	UNCLEAR	UNCLEAR		UNCLEAR
Gheorghe 2016	FAIR<br>Mode of collection differed but health questions remained the same.	Health: GOOD	FAIR: No	UNCLEAR	UNCLEAR	UNCLEAR		UNCLEAR
Guzman-Castillo 2017	NA as forecasting from baseline data	Disability: GOOD	FAIR: No	NA (forecasts)	GOOD<br>Full interview by proxy 4%	UNCLEAR	Rogers et al. (2016) DOI 10.1007/978-981-287-080-3_52-1	FAIR

For peer review only - http://bmjopen.bmj.com/site/about/guidelines.xhtml

Study	Comparability of interview methods	Outcome measure	Does the trend consider more than 2 time points?	% Response rate in cross-sectional surveys or loss to follow up in longitudinal studies	Proxy %	Missing data %	Other publications consulted	SUMMARY JUDGMENT
					Partial interview or institute proxy <1%		https://www.ifs.org.uk/uploads/elsa/docs_w7/ELSA%20Wave%207%20report.pdf	
Jagger 2016	GOOD	SRH: FAIR<br>Disability: GOOD	FAIR: No	FAIR<br><br>Response rates:<br><br>CFAS I: 80%<br>CFAS II: 56%	GOOD<br><br>CFAS I: 0.1%<br>CFAS II: 0.1%	GOOD<br>SRH: 2·9% (1991) and 4·2% (2011)<br>Disability: 1·1% (1991) and 4·2% (2011)	https://www.sciencedirect.com/science/article/pii/S0140673613615706<br><br>https://ars.els-cdn.com/content/image/1-s2.0-S0140673613615706-mmc1.pdf	FAIR/GOOD depending on outcome
Jo 2019	GOOD<br>Although there were changes to the survey, only waves 3-5 were used where the health question used was the same.	QALE: GOOD	GOOD: Yes	UNCLEAR<br>Not reported	GOOD<br>3-4%	GOOD<br>3-4%	https://www.ncbi.nlm.nih.gov/pmc/articles/PMC3937975/	UNCLEAR
Kingston 2017	GOOD	Disability: GOOD	GOOD: Yes (fourth and fifth time points are projections)	FAIR<br><br>Response rates:<br><br>CFAS I: 80%<br>CFAS II: 56%	GOOD<br><br>CFAS I: https://ars.els-cdn.com/content/image/1-s2.0-S0140673613615706-mmc1.pdf<br>CFAS II: 0.1%	GOOD<br>Not reported but assumed to be same as Jagger et al. as uses same data.	https://www.sciencedirect.com/science/article/pii/S0140673613615706<br><br>https://ars.els-cdn.com/content/image/1-s2.0-S0140673613615706-mmc1.pdf	FAIR
Kingston 2018	NA as forecasting from baseline data	Disability: GOOD	GOOD: Yes (third time point is projection)	FAIR<br><br>Response rates:<br><br>CFAS II: 56%<br>ELSA Wave 5: 80%<br>Understanding society wave 1: 57% (General population)	UNCLEAR/GOOD depending on dataset<br><br>CFAS II: 0.1% (GOOD)<br><br>No information on % proxy	UNCLEAR<br>Not reported, but missing values imputed.	https://www.understandingsociety.ac.uk/sites/default/files/downloads/documentation/mainstage/technical-reports/Wave_1_Technical_Report.pdf	FAIR

Study	Comparability of interview methods	Outcome measure	Does the trend consider more than 2 time points?	% Response rate in cross-sectional surveys or loss to follow up in longitudinal studies	Proxy %	Missing data %	Other publications consulted	SUMMARY JUDGMENT
				and 40% (Ethnic Minority Boost)	interviews for ELSA (UNCLEAR).			
Lagergren 2017	POOR<br>"In the years 1996–2000, a filter was introduced into the questionnaire to the effect that only people who were dependent for all instrumental ADL (IADL) were asked the questions about ADL." (p.552)<br><br>"From 1980 to 2001 there was an upper age limit of 84 years, with the exception of 1988/89." (p.56)	Disability: GOOD	GOOD: Yes	UNCLEAR<br>Response rate reported to be 70-75% with slight decrease up to 2006, but 'lower' after 2006 (actual rates not reported).	FAIR<br>From methods paper on dataset: "The proportion of proxy interviews varies from 2–3% among those aged 65–79 years to 14–16% among individuals aged 80 years and older." (p.56)	UNCLEAR	https://journals.sagepub.com/doi/full/10.1177/1403494815605195	POOR
Lee 2016	GOOD<br>Same criteria used across the waves used to identify diseases.	GOOD<br>Health adjusted life expectancy was calculated using disability weights attached to self-reported morbidity for 45 diseases. This was converted into a score of disability measured on a scale from 0 (full health) to 1 (being dead). This would suggest it	GOOD: Yes	UNCLEAR<br>Not reported, but 2011 KNHANES wave known to be 76.1% for health interview and examination (Jo et al., 2019)	GOOD<br>3-4%<br>Not reported, but assumed to be the same as for Lee et al., 2016, which uses the same data.	GOOD<br>3-4%<br>Not reported, but assumed to be the same as for Lee et al., 2016, which uses the same data.		UNCLEAR

For peer review only - http://bmjopen.bmj.com/site/about/guidelines.xhtml

Study	Comparability of interview methods	Outcome measure	Does the trend consider more than 2 time points?	% Response rate in cross-sectional surveys or loss to follow up in longitudinal studies	Proxy %	Missing data %	Other publications consulted	SUMMARY JUDGMENT
		is a multiple item measure.						
ONS 2018	GOOD<br>No change in health question/measure, but items used to measure disability changed from the 2013 APS survey (p.4). However, the ONS analysis does not report trends in DFLE (HLE and LE only).	Health: FAIR	FAIR: No	UNCLEAR	UNCLEAR	UNCLEAR<br>Methods report states that missing data are deleted from the survey, but there is no detail on what proportion of data is missing.	https://www.ons.gov.uk/peoplepopulationandcommunity/healthandsocialcare/healthandlifeexpectancies/methodologies/healthstatelifeexpectanciesukqmi#_blank	UNCLEAR
ONS 2019	GOOD<br>No change in health question/measure, but items used to measure disability changed from the 2013 APS survey (p.4). However, the ONS analysis does not report trends in DFLE (HLE and LE only).	Health: FAIR	FAIR: No	UNCLEAR	UNCLEAR	UNCLEAR<br>Methods report states that missing data are deleted from the survey, but there is no detail on what proportion of data is missing.	https://www.ons.gov.uk/peoplepopulationandcommunity/healthandsocialcare/healthandlifeexpectancies/methodologies/healthstatelifeexpectanciesukqmi#_blank	UNCLEAR
Remund 2019	POOR<br>The phrasing of the SRH question and response items were not identical in each survey wave. However, additional analysis supported the use of	SRH: FAIR	GOOD: Yes	GOOD<br><br>Response rates:<br><br>1992: 70.8%<br>1997: 68.8%<br>2002: 63.9%<br>2007: 66.3%	GOOD<br>(% cases, % weights)<br>1992: 0.67%, 0.86%<br>1997: 0.74%, 0.79%	GOOD<br>(% cases, % weights)<br>1992: 0.0%, 0.0%<br>1997: 0.03%, 0.04%	Supplementary materials	POOR

Study	Comparability of interview methods	Outcome measure	Does the trend consider more than 2 time points?	% Response rate in cross-sectional surveys or loss to follow up in longitudinal studies	Proxy %	Missing data %	Other publications consulted	SUMMARY JUDGMENT
	dichotomisation to maximise the comparability of the questions between waves.			2012: 53.1	2002: 1.05%, 0.86%<br>2007: 4.41%, 6.23%<br>2012: 3.32%, 4.26%	2002: 0.03%, 0.02%<br>2007: 0.05%, 0.05%<br>2012: 0.12%, 0.12%		
Renard 2019	GOOD<br>Methods paper states no major methodological changes have been implemented between waves, except oversampling from 1997.	Disability: FAIR (Single item question - Global Activity Limitation Indicator)	FAIR: No	FAIR<br><br>Response rates:<br><br>2001: 49%<br>2004: 49.9%<br>2008: 40.2%<br>2013: Not reported.	UNCLEAR	UNCLEAR	https://archpublichealth.biomedcentral.com/articles/10.1186/0778-7367-71-24	FAIR
Reus-Pons 2017	GOOD<br>For England & Wales data, paper indicates SRH question changed but an adjustment was made for comparability. For Netherlands data, footnote to table states no major changes were made to SRH question and choices.	SRH: FAIR	FAIR: No	UNCLEAR<br><br>Response rates:<br><br>POLS: UNCLEAR<br>Census 2011: 94% (person response rate)<br>Census 2001: 98%	UNCLEAR	UNCLEAR<br>For Belgium sample: data on self-rated health were missing for 'around' 5% of the non-migrant population and 'around' 10% of the migrant population. Exact % not reported, thus could be >10%.<br>Not reported for England	https://www.mzes.uni-mannheim.de/projekte/mikrodaten/wp_pdf/wp_30_NL-ro.pdf<br><br>https://www.ons.gov.uk/census/2001censusandearlier/dataandproducts/qualityofthecensusdata/responserates<br><br>https://www.ons.gov.uk/peoplepopulationandcommunity/populationandmigration/populationestimates/bulletins/2011censuspopulationandhouseholdestimatesforenglandandwales/2012-07-16	UNCLEAR

For peer review only - http://bmjopen.bmj.com/site/about/guidelines.xhtml

Study	Comparability of interview methods	Outcome measure	Does the trend consider more than 2 time points?	% Response rate in cross-sectional surveys or loss to follow up in longitudinal studies	Proxy %	Missing data %	Other publications consulted	SUMMARY JUDGMENT
						and Wales sample.		
Steensma 2017	GOOD<br>Different surveys used between 1994-1999 and 2000-2010, but same question for health used in both.	Health: GOOD (multiple item health utilities index)	GOOD: Yes	GOOD<br><br>Response rates:<br><br>NPHS:<br>1994/95: 88.7%,<br>1996/97: 82.6%<br>1998/99: 89.7%<br><br>CCHS:<br>2000/01: 84.7%<br>2009/10: 72.3%	POOR (NPHS), GOOD (CCHS)<br><br>NPHS: 27.5% (for health data)<br><br>CCHS: 6.3%	NPHS: GOOD 1994/1995: between 1% and 9% depending on variable.<br>CCHS: UNCLEAR	https://www23.statcan.gc.ca/imdb/p2SV.pl?Function=getSurvey&Id=3359<br>https://pubmed.ncbi.nlm.nih.gov/10607414/<br>https://pubmed.ncbi.nlm.nih.gov/11565112/	GOOD
Storeng 2018	POOR<br>Self-rated health was measured the same at all three HUNT studies. The question for longstanding limiting illness differed between HUNT1 and HUNT 2 and 3 (that is, HUNT 2 and 3 asked about limiting illness lasting for at least one year, whereas HUNT 1 did not).	Health: FAIR<br>Disability: FAIR	GOOD: Yes	FAIR<br><br>Response rates:<br><br>HUNT 1: 90%<br>HUNT 3: 54%	UNCLEAR	GOOD<br>Missing health data < 5%	https://academic.oup.com/ije/article/42/4/968/655743#11331793	POOR
Sugawara 2016	GOOD	FAIR<br>Disability was measured using the following two survey questions: “Are you currently institutionalized in hospitals,	FAIR: No	GOOD<br><br>Response rates:<br><br>2001: 87.4%<br>2010: 79.4%	UNCLEAR	UNCLEAR		FAIR

For peer review only - http://bmjopen.bmj.com/site/about/guidelines.xhtml

Study	Comparability of interview methods	Outcome measure	Does the trend consider more than 2 time points?	% Response rate in cross-sectional surveys or loss to follow up in longitudinal studies	Proxy %	Missing data %	Other publications consulted	SUMMARY JUDGMENT
		clinics, or long-term care facilities?" and "Do you have any limitations in carrying out normal activities due to health problems?" Those who answered "yes" to **either** question are considered to have disability.						
Sundberg 2016	FAIR<br>Mode of data collection changed but health question remain the same.	Disability: GOOD	GOOD: Yes	GOOD/FAIR depending on dataset<br><br>Response rates:<br><br>SWEOLD<br>1992: 95.4%<br>2002: 84.4%<br>2004: 46.9%<br>2011: 86.2%<br><br>SHARE (retention plus recovery):<br>2004: 70.6%<br>2011:108.4%	FAIR/POOR depending on dataset<br><br>SHARE<br>2004: 14.4%<br>2011: 9 %<br><br>SWEOLD<br>1992: 15%<br>2002: 19.8%<br>2004: 21.5%<br>2011: 20.9%	GOOD/UNCLEAR depending on dataset<br><br>SHARE<br>Not reported.<br><br>SWEOLD (severe disability, mild disability, mobility)<br>1992: 1.7%, 0.4%, 0%<br>2002: 1.0%, 1.1%, 1.3%<br>2004: 2.0%, 0.9%, 0.9%<br>2011: 2.4%, 1.3%, 1.4%	https://link.springer.com/article/10.1007%2Fs10433-013-0275-7 and https://academic.oup.com/ije/article/43/3/731/2949546#57616062<br><br>https://academic.oup.com/ije/article/43/3/731/2949546<br><br>https://academic.oup.com/ije/article/42/4/992/657275<br><br>Borsch-Supan, A & Scherpenzeel , A. The Survey of Health, Ageing and Retirement in Europe. Munich Center for the Economics of Aging,	FAIR

For peer review only - http://bmjopen.bmj.com/site/about/guidelines.xhtml

Study	Comparability of interview methods	Outcome measure	Does the trend consider more than 2 time points?	% Response rate in cross-sectional surveys or loss to follow up in longitudinal studies	Proxy %	Missing data %	Other publications consulted	SUMMARY JUDGMENT
Tokudome 2016	GOOD	Disability: GOOD	GOOD: Yes	UNCLEAR<br>(Based on HALE values from GBD 2013)	UNCLEAR	UNCLEAR<br>But reported to be rare by author.	http://www.healthdata.org/sites/default/files/files/Projects/GBD/GBD_Protocol.pdf	UNCLEAR
Yokota 2019	GOOD<br>Methods paper states no major methodological changes have been implemented between waves, except oversampling from 1997.	Disability: GOOD	GOOD: Yes	FAIR<br><br>2001: 49%<br>2004: 49.9%<br>2008: 40.2%	FAIR<br>Time 1: 4.3%,<br>time 2: 10.2%,<br>time 3: 13%	UNCLEAR	https://archpublichealth.biomedcentral.com/articles/10.1186/0778-7367-71-24	FAIR

For peer review only - http://bmjopen.bmj.com/site/about/guidelines.xhtml

Table 3a. Summary of life expectancy trends data for studies from the US and Canada (years)[a]

Study	At age	Time points	T1		T2		T3		T4		T5		T6		T7		Change between first and last time point
			M	F	M	F	M	F	M	F	M	F	M	F	M	F	
Cao 2016	55-85	T1: 1982<br>T2: 1990<br>T3: 2000<br>T4: 2010<br>T5: 2020<br>T6: 2030<br>T7: 2040	19.96	23.40	20.70	23.64	21.75	23.92	22.82	24.69	23.83 (23.5 - 24.05)	25.11 (24.9 - 25.24)	24.63 (24.2 - 24.98)	25.49 (25.2 - 25.69)	25.24 (24.7 - 25.67)	25.74 (25.4 - 26.00)	Men: 5.28[b]<br>Women: 2.34[b]
Crimmins 2016	0	T1: 1970<br>T2: 1980<br>T3: 1990<br>T4: 2000<br>T5: 2010	67.0	74.6	70.1	77.6	71.8	78.8	74.1	79.5	76.2	81.0					Men: 9.2<br>Women: 6.4
	20-64		40.8	42.8	41.6	43.3	41.8	43.5	42.4	43.6	42.6	43.7					Men: 1.8<br>Women: 0.9
	65		13.0	16.8	14.2	18.4	15.1	19.0	16.1	19.1	17.7	20.3					Men: 4.7<br>Women: 3.5
	85		4.7	5.6	5.1	6.4	5.3	6.7	5.5	6.6	5.8	6.9					Men: 1.1<br>Women: 1.3
Steensma 2017	0	T1: 1994/95<br>T2: 96/97<br>T3: 98/99<br>T4: 00/01<br>T5: 03<br>T6: 05<br>T7: 09/10	75.2	81.3	75.7	81.5	76.2	81.8	77.2	82.5	77.7	82.8	78.3	83.2	79.6	84.1	Men: 4.4[b]<br>Women: 2.8[b]
	20		55.7	61.7	56.2	61.8	56.7	62.1	57.7	62.8	58.1	63.0	58.7	63.4	59.9	64.4	Men: 4.2[b]<br>Women: 2.7[b]
	65		15.8	19.9	15.9	19.9	16.2	20.2	17.0	20.7	17.4	20.9	19.9	21.3	18.9	22.1	Men: 3.1[b]<br>Women: 2.2[b]

[a]95% confidence intervals in brackets where reported; [b]Change estimate not reported, and thus calculated by us (T^2-T^1)

For peer review only

Table 3b. Summary of healthy life expectancy trends data for studies from the US and Canada (years)[a]

Study	At age	Time points	T1		T2		T3		T4		T5		T6		T7		Average annual change (%)
			M	F	M	F	M	F	M	F	M	F	M	F	M	F	
Steensma 2017	0	T1: 1994/95 T2: 96/97 T3: 98/99 T4: 00/01 T5: 03 T6: 05 T7: 09/10	66.4 (66.0 - 66.8)	70.0 (69.5 - 70.4)	69.0 (68.7 - 69.2)	72.9 (72.5 - 73.2)	68.9 (68.6 - 69.3)	72.1 (71.7 - 72.5)	68.5 (68.3 - 68.7)	71.5 (71.3 - 71.7)	69.5 (69.1 - 69.8)	71.7 (71.3 - 72.1)	69.3 (69.0 - 69.7)	72.3 (71.9 - 72.6)	70.7 (70.5 - 70.9)	73.3 (73.1 - 73.5)	Men: 0.3 Women: 0.2
	20		48.0 (47.6 - 48.3)	51.4 (51.0 - 51.9)	50.1 (49.8 - 50.3)	54.0 (53.7 - 54.3)	50.1 (49.7 - 50.4)	53.1 (52.7 - 53.5)	49.8 (49.6 - 50.0)	52.7 (52.5 - 52.9)	50.9 (50.5 - 51.2)	53.1 (52.7 - 53.5)	50.8 (50.4 - 51.2)	55.3 (53.1 - 53.9)	50.2 (51.8 - 52.2)	54.5 (54.3 - 54.8)	Men: 0.4 Women: 0.2
	65		12.3 (12.0 - 12.6)	14.8 (14.4 - 15.1)	12.8 (12.6 - 13.1)	15.6 (15.3 - 15.9)	12.8 (12.5 - 13.1)	15.3 (15.0 - 15.7)	13.1 (13.0 - 13.3)	15.4 (15.2 - 15.6)	14.0 (13.7 - 14.3)	15.7 (15.4 - 16.1)	14.2 (13.9 - 14.5)	16.0 (15.6 - 16.3)	14.9 (14.7 - 15.1)	16.8 (16.7 - 17.0)	Men: 1.2 Women: 0.7

[a]95% confidence intervals in brackets where reported;

For peer review only

For peer review only - http://bmjopen.bmj.com/site/about/guidelines.xhtml

Table 3c. Summary of disability-free life expectancy trends data for studies from the US and Canada[a]

Study	Trend	At age	Time points	T1 Male	T1 Female	T2 Male	T2 Female	T3 Male	T3 Female	T4 Male	T4 Female	T5 Male	T5 Female	Change between first and last time point
Crimmins (2016)	DFLE (years)	0	T1: 1970 T2: 1980 T3: 1990 T4: 2000 T5: 2010	56.5 (56.4 - 56.6)	62.7 (62.6 - 62.8)	56.5 (56.4 - 56.6)	62.8 (62.6 - 63.0)	58.8 (58.6 - 58.9)	63.9 (63.8 - 64.0)	60.0 (59.9 - 60.2)	64.6 (64.4 - 64.7)	61.0 (60.9 - 61.2)	65.4 (65.3 - 65.6)	Men: 4.5 Women: 2.7
		20-64		34.9 (34.8 - 35.0)	37.4 (37.3 - 37.5)	34.7 (34.6 - 34.8)	36.7 (36.6 - 36.8)	35.3 (35.2 - 35.4)	37.0 (36.9 - 37.1)	35.6 (35.5 - 35.7)	36.9 (36.8 - 37.0)	35.8 (35.7 - 35.9)	36.8 (36.7 - 37.0)	Men: 0.9 Women: -0.6
		65		6.6 (6.6 - 6.8)	9.1 (9.0 - 9.2)	6.8 (6.7 - 6.9)	9.3 (9.2 - 9.4)	7.4 (7.3 - 7.5)	9.9 (9.8 - 10.0)	8.2 (8.0 - 8.3)	10.5 (10.3 - 10.6)	9.3 (9.1 - 9.4)	11.5 (11.4 - 11.6)	Men: 2.7 Women: 2.4
		85		1.4 (1.3 - 1.6)	1.4 (1.3 - 1.6)	1.5 (1.3 - 1.7)	1.7 (1.5 - 1.8)	1.6 (1.5 - 1.8)	1.7 (1.6 - 1.8)	1.8 (1.6 - 1.9)	1.9 (1.8 - 2.0)	1.9 (1.8 - 2.1)	2.2 (2.1 - 2.4)	Men: 0.5 Women: 0.8
	% of life spent disability-free	0		84.2	84.0	81.6	80.9	81.8	81.1	81.0	81.3	80.1	80.7	Men: -4.1 Women: -3.3
		20-64		85.5	87.4	83.4	84.8	84.4	85.0	84.0	84.6	84.0	84.2	Men: -1.5 Women: -3.2
		65		51.2	54.2	47.8	50.5	49.1	52	50.9	55	52.5	56.7	Men: 1.3 Women: 2.5
		85		30.4	25.2	29	26	30.8	25.5	32.7	28.8	32.8	31.9	Men: 2.4 Women: 6.7
	LE for those in an institution	0		0.6 (0.6 - 0.6)	1.1 (1.0 - 1.2)	0.6 (0.6 - 0.7)	1.4 (1.3 - 1.5)	0.7 (0.6 - 0.7)	1.5 (1.4 - 1.5)	0.5 (0.5 - 0.6)	1.1 (1.0 - 1.2)	0.4 (0.4 - 0.5)	0.8 (0.8 - 0.9)	Men: -0.2 Women: -0.3
		20-64		0.3 (0.2 - 0.3)	0.2 (0.2 - 0.2)	0.2 (0.2 - 0.2)	0.2 (0.1 - 0.2)	0.2 (0.2 - 0.2)	0.1 (0.1 - 0.1)	0.1 (0.1 - 0.1)	0.1 (0.1 - 0.1)	0.1 (0.1 - 0.1)	0.1 (0.0 - 0.1)	Men: -0.2 Women: -0.1

For peer review only - http://bmjopen.bmj.com/site/about/guidelines.xhtml

Study	Trend	At age	Time points	T1		T2		T3		T4		T5		Change between first and last time point
				Male	Female	Male	Female	Male	Female	Male	Female	Male	Female	
		65		0.5 (0.5 - 0.5)	1.1 (1.0 - 1.2)	0.6 (0.5 - 0.6)	1.5 (1.4 - 1.6)	0.7 (0.6 - 0.7)	1.6 (1.5 - 1.6)	0.5 (0.5 - 0.6)	1.2 (1.1 - 1.3)	0.4 (0.4 - 0.5)	0.9 (0.8 - 0.9)	Men: -0.1<br>Women: -0.2
		85		0.6 (0.5 - 0.7)	1.2 (1.1 - 1.3)	0.8 (0.7 - 1.0)	1.7 (1.5 - 1.8)	0.9 (0.8 - 1.0)	1.9 (1.8 - 2.0)	0.7 (0.6 - 0.8)	1.4 (1.3, 1.5 -	0.4 (0.4 - 0.5)	0.9 (0.8 - 1.0)	Men: -0.2<br>Women: -0.3
Cao 2016	DFLE (years)	55-85	T1: 1982<br>T2: 1990<br>T3: 2020<br>T4: 2030<br>T5: 2040	12.98	14.9	16.92	17.53	17.86 (17.70 - 18.03)	18.00 (17.89 - 18.13)	18.99 (18.68 - 19.36)	18.67 (18.41 - 18.97)	20.10 (19.60 - 20.64)	19.30 (18.88 - 19.77)	Men: 7.12[b]<br>Women: 4.4[b]
Freedman (2016)a	DFLE (years)	65	T1: 1982<br>T2: 2004<br>T3: 2011	**data only reported in graphs, unable to extract**										Men: 4.5<br>Women: 1.4
		85		2.5	2.5	4.4	2.6							Men: 1.9<br>Women: 0.1[b]
Freedman (2016)b	Active Life Expectancy (years)	65	T1: 1982<br>T2: 2011	White: 12.2<br>Black: 9.8		White: 15.9<br>Black: 12.0								White: 2.8<br>Black: 2.2
		85		White: 2.5<br>Black: 2.25		White: 3.0<br>Black: 2.0								White: 0.5<br>Black: -0.25

[a]95% confidence intervals in brackets where reported; [b]Change estimate not reported, and thus calculated by us (T^2-T^1)

For peer review only

Table 3d. Summary of disability prevalence trends data for studies from the US and Canada[a]

Study	Trend	Time points	Change between first and last time point
Crimmins (2016)	Proportion (%) of individuals with disability in the community (age 65-84)	T1: 1970<br>T2: 1980	Men: -2.5<br>Women: -2.2
	Proportion (%) of individuals with disability in the community (age 85+)	T3: 1990<br>T4: 2000<br>T5: 2010	Men: -0.5<br>Women: -5.8
Freedman (2016)	Proportion of those with a severe limitation (limited in 3+ personal care activities or living in a nursing home)	T1: 1982<br>T2: 2004<br>T3: 2011	Men: -3.4[b]<br>Women: -3.0[b]

[a]Estimates for each time point were not extracted here due to volume. Reader is referred to publications and associated supplementary materials; [b]Change estimate not reported, and thus calculated by us (T^2-T^1)

For peer review only - http://bmjopen.bmj.com/site/about/guidelines.xhtml

Table 4a. Summary of life expectancy trends data for UK studies and studies with UK samples (years)[a]

Study author and date	At age	Time points	T1		T2		T3		Change between first and last time point
			Male	Female	Male	Female	Male	Female	
Reus-Pons 2017	50	T1: 2001<br>T2: 2011	28.54<br><br>Immigrant: 27.52<br>Non-immigrant: 28.66	32.32<br><br>Immigrant: 31.82<br>Non-immigrant: 32.39	31.29<br><br>Immigrant: 31.01<br>Non-immigrant: 31.32	34.5<br><br>Immigrant: 34.84<br>Non-immigrant: 34.47			Men: 2.8[b]<br>Women: 2.2[b]
Jagger 2016	65	T1: 1991<br>T2: 2011	13·0	16·7	17·5	20·3			Men: 4·5<br>Women: 3·6
Kingston 2018	65	T1: 2015<br>T2: 2025<br>T3: 2035	18·7 (18·3 to 19·0)	21·1 (20·8 to 21·1)	20·7 (20·5 to 21·0)	22·7 (22·5 to 23·3)	22·2 (21·7 to 22·4)	24·1 (23·9 to 24·4)	Men: 3·5 (3·1 to 4·1)<br>Women: 3·0 (3·0 to 3·6)
Kingston 2017	65	T1: 1991<br>T2: 2011	12.9	16.5	17.6	20.6			Men: 4.7<br>Women: 4.1
ONS 2018	0	T1: 2009/12<br>T2: 2015/17	**Not reported**						UK<br>Men: 0.8<br>Women: 0.4<br>England<br>Men: 0.8<br>Women: 0.4<br>Northern Ireland<br>Men: 1.0<br>Women:0.5<br>Scotland<br>Men: 0.8<br>Women: 0.5

For peer review only - http://bmjopen.bmj.com/site/about/guidelines.xhtml

Study author and date	At age	Time points	T1		T2		T3		Change between first and last time point
			Male	Female	Male	Female	Male	Female	
									Wales<br>Men: 0.5<br>Women: 0.2

[a]95% confidence intervals in brackets where reported; [b]Change estimate not reported in publication, and calculated by us (T^2-T^1);

For peer review only

Table 4b. Summary of healthy life expectancy trends data for UK studies and studies with UK samples[a]

Study	Trend	At age	Time points	T1		T2		Change between first and last time point
				Male	Female	Male	Female	
Reus-Pons 2017	HLE (years)	50	T1: 2001 T2: 2011	18.47 (18.46-18.48)	19.82 (19.81-19.83)	18.71 (18.70-18.72)	19.67 (19.66-, 19.68)	Men: 0.25 Women: -0.15
Jagger 2016	HLE (years)	65	T1: 1991 T2: 2011	8·8 (8·6-9·1)	11·2 (11·0 - 11·5)	12·6 (12·4-12·9)	14·3 (14·0 - 14·6)	Men: 3·8 (3·5 to 4·1) Women: 3·1 (2·7 to 3·4)
	Proportion (%) of life spent healthy			68·2% (66·5 - 69·9)	67·3% (65·9 - 68·7)	72·4% (70·9 - 73·9)	70·3% (68·8 - 71·7)	Men: 4·2% (2·0 to 6·5) Women: 3·0% (1·0 to 4·9)
ONS 2018	HLE (years) - UK	0	T1: 2009/11 T2: 2015/17	62.7	63.8	63.1	63.6	Men: 0.4 [b] Women: -0.2 [b]

[a]95% confidence intervals in brackets where reported; [b]Change estimate not reported in publication, and calculated by us (T^2-T^1);

For peer review only - http://bmjopen.bmj.com/site/about/guidelines.xhtml

Table 4c. Summary of disability-free life expectancy trends data for UK studies and studies with UK samples[a]

Study	Trend	At age	Time points	T1		T2		T3		Change between first and last time point
				Male	Female	Male	Female	Male	Female	
Jagger 2016	DFLE (years)	65	T1: 1991<br>T2: 2011	10.3 (10.2-10.5)	11.0 (10.8-11.2)	12.9 (12.7-13.2)	11.5 (11.3-11.8)			Men: 2.6 (2.3, 2.9)<br>Women: 0.5 (0.2, 0.9)
	% of life disability-free			79.7 (78.3-81.0)	66.1 (64.9-67.4)	74.4 (73.0-75.8)	56.8 (55.5-58.2)			Men: -5.3 (-7.2, -3.4)<br>Women: -9.3 (-11.1, -7.5)
Kingston 2017	DFLE (years)	65	T1: 1991<br>T2: 2011	9.5 (9.3-9.7)	9.5 (9.2-9.8)	11.2 (10.8-11.5)	9.7 (9.3-10.2)			Men: 1.7 (1.2, 2.1)<br>Women: 0.2 (-0.4, 0.7)
	% of life disability-free			73.6 (71.8-75.4)	58.0 (56.2-59.9)	63.5 (61.4-65.6)	47.3 (45.0-49.5)			Men: -10.1 (-12.9, -7.3)<br>Women: -10.7 (-13.6, -7.8)
Kingston 2018	DFLE (years)	65	T1: 2015<br>T2: 2025<br>T3: 2035	11.1 (10.9-11.3)	10.7 (10.5-10.7)	14.5 (14.4-14.6)	11.4 (11.3-11.5)	15.2 (15.1-15.2)	11.6 (11.6-11.8)	Men: 4.2 (3.9, 4.2)<br>Women: 0.9 (0.9, 1.2)
	% of life disability-free			59.3 (59.3-60.2)	50.6 (50.1-50.7)	70.2 (69.7-70.4)	49.9 (49.4-50.3)	68.7 (67.9-69.5)	48.0 (48.0-48.6)	Men: 9.4 (8.0, 9.7)<br>Women: -2.6%
Guzman-Castillo 2017	DFLE (years)	65	T1: 2015<br>T2: 2025	14.9 (14.7-15.1)	15.8 (15.7-15.9)	16.5 (15.4-17.6)	16.4 (15.1-17.7)			Men: 1.6 (0.5, 2.7)<br>Women: 0.6 (-0.7, 1.9)

[a]95% confidence intervals in brackets where reported;

For peer review only - http://bmjopen.bmj.com/site/about/guidelines.xhtml

Table 4d. Summary of disability prevalence trends data for UK studies and studies with UK samples[a,b]

Study	Trend	Time points	Change between first and last time point
Jagger 2016	Odds of any disability at time 2 compared to time 1	T1: 1991<br>T2: 2011	1.22 (1.14-1.30)
	Odds of moderate-severe disability at time 2 compared to time 1		0.76 (0.70-0.82)
	Odds of fair or poor self-rated health at time 2 compared to time 1		0.83 (0.78–0.88)
Kingston 2017	% increase in projected number of people with high dependency	T1: 2015<br>T2: 2025<br>T3: 2035	61.5
	% increase in projected number of people with medium dependency		66.6
	% increase in projected number of care home places needed for those with high dependency		84.5
	% increase in projected number of care home places needed for those with medium dependency		89.3
Kingston 2018	Relative change in projected proportion (%) of sample with high dependency (65-74 years)	T1: 2015<br>T2: 2025<br>T3: 2035	−15.0 (−20.0 to −13.0)
	Relative change in projected proportion (%) of sample with high dependency (75 - 84 years)		42.0 (36.6- 42.7)
	Relative change in projected proportion (%) of sample with high dependency (85+ years)		91.8 (87.3- 94.1)
	Relative change in projected proportion (%) of sample with high dependency (65+)		36.0 (32.6- 36.0)
Guzman-Castillo 2017	Relative change in projected number of disability cases 2015-2025, 65+	T1: 2015<br>T2: 2025	25.0 (21.3–28.2)
	Relative change in projected number of disability cases 2015-2025, 65-84		18.9 (16.6–20.9)
	Relative change in projected number of disability cases 2015-2025, 85		43.2 (34.2–52.1)

[a]Estimates for each time point were not extracted here due to volume. Reader is referred to publications and associated supplementary materials; [b]95% confidence intervals in brackets where reported;

For peer review only

For peer review only - http://bmjopen.bmj.com/site/about/guidelines.xhtml

Table 4e. Summary of inequalities in life expectancy trends, reported as the slope index of inequality between those living in the most and least deprived decile of area deprivation

Study	Trend	Time points	Change between first and last time point (range)
ONS 2019	Change in SSI in life expectancy at birth, England	T1: 2012/14<br>T2: 2015/17	Men: 0.3 (0.2)<br>Women: 0.5 (0.5)
	Change in SSI in life expectancy at age 65, England		Men: 0.3 (0.2)<br>Women: 0.5 (0.5)
	Change in SSI in life expectancy at birth, Wales		Men: 0.5 (0.3)<br>Women: 0.6 (0.7)
	Change in SSI in life expectancy at age 65, Wales		Men: 0.3 (0.2)<br>Women: 0.1 (0.3)

For peer review only - http://bmjopen.bmj.com/site/about/guidelines.xhtml

Table 4f. Summary of inequalities in healthy life expectancy trends, reported as the slope index of inequality between those living in the most and least deprived decile of area deprivation

Study	Trend	Time points	Change between first and last time point (range)
ONS 2019	Change in SSI in healthy life expectancy at birth, England	T1: 2012/14<br>T2: 2015/17	Men: 0.2 (0.2)<br>Women: -0.9 (-0.8)
	Change in SSI in healthy life expectancy at age 65, England		Men: 0.4 (0.9)<br>Women: -0.3 (-0.6)
	Change in SSI in healthy life expectancy at birth, Wales		Men: 0.2 (0.9)<br>Women: -1.1 (-0.9)
	Change in SSI in healthy life expectancy at age 65, Wales		Men: -0.6 (0.8)<br>Women: -1.0 (0.2)

For peer review only

For peer review only

For peer review only - http://bmjopen.bmj.com/site/about/guidelines.xhtml

Table 5a. Summary of life expectancy trends data for studies from Europe (years)[a]

Study	At age	Time points	T1		T2		T3		T4		T5		T6		T7		T8		Change between first and last time point
			M	F	M	F	M	F	M	F	M	F	M	F	M	F	M	F	
Bronnum-Hansen 2017	65	T1: 2006-2007<br>T2: 2010-2011<br>T3: 2013-2014	High: 17.9<br>Med: 16.3<br>Low: 15.6	High: 20.4<br>Med: 19.4<br>Low: 18.5	Med: 16.8<br>Low: 16.2	High: 21.0<br>Med: 20.0<br>Low: 18.9	High: 19.3<br>Med: 17.5<br>Low: 16.9	High: 21.7<br>Med: 20.6<br>Low: 19.5											Difference in change in LE between high and low education over time:<br>Men: 0.1<br>Women: 0.3
Deeg 2018	65	T1: 1993<br>T2: 1996<br>T3: 1999<br>T4: 2002<br>T5: 2006<br>T6: 2009<br>T7: 2012<br>T8: 2016	14.7	19.2	15.1	19.4	15.5	19.5	16	19.7	17.1	20.5	17.8	21.1	18.2	21.2	18.7	21.4	Men: 4.0<br>Women: 2.2
Gheorghe 2016[b]	25	T1: 2001<br>T2: 2011	High: 53.83<br>Med: 50.70<br>Low: 47.85	High: 58.75<br>Med: 56.82<br>Low: 53.57	High: 56.71<br>Med: 53.92<br>Low: 50.28	High: 60.53<br>Med: 58.35<br>Low: 54.72													Men:<br>High: 2.88<br>Med: 3.22<br>Low: 2.43<br>Women:<br>High: 1.78<br>Med: 1.53<br>Low: 1.15
	65		High: 16.81<br>Med: 15.29<br>Low: 14.08	High: 21.00<br>Med: 20.08<br>Low: 18.26	High: 19.29<br>Med: 17.53<br>Low: 15.76	High: 22.64<br>Med: 21.49<br>Low: 19.38													Men:<br>High: 2.48<br>Med: 2.24<br>Low: 1.68<br>Women:<br>High: 1.64<br>Med: 1.41<br>Low: 1.12
Remund 2019	30	T1: 1990/94<br>T2: 1995/99<br>T3: 2000/04<br>T4: 2005/09<br>T5: 2010/14	**data only reported in graphs, unable to extract**																Men: 5.02<br>Women: 3.09

For peer review only

For peer review only

Study	At age	Time points	T1		T2		T3		T4		T5		T6		T7		T8		Change between first and last time point
			M	F	M	F	M	F	M	F	M	F	M	F	M	F	M	F	
Renard 2019[b]	25	T1: 2001<br>T2: 2011	High: 55.82<br>Med: 53.18<br>Low: 50.63	High: 60.90<br>Med: 59.14<br>Low: 57.14	High: 57.81<br>Med: 54.73<br>Low: 51.74	High: 61.90<br>Med: 60.09<br>Low: 57.32													Men:<br>Low: 1.11<br>Med: 1.55<br>High: 1.99<br><br>WOMEN:<br>Low: 0.18<br>Med: 0.95<br>High: 1.00
Reus-Pons 2017	50	T1: 2001<br>T2: 2011	28.05	32.47	30.87	34.31													Men: 2.82 [d]<br>Women: 1.84 [d]
Storeng 2018	30	T1: 1984/86<br>T2: 1995/97<br>T3: 2006/08	45.1 (44.6–45.5)	50.4 (49.9–50.8)	48.3 (47.8–48.7)	52.9 (52.5–53.4)	52.1 (51.5–52.6)	57.1 (56.6–57.6)											Men: 6.99 (5.27–8.72)<br>Women: 6.75 (5.16–8.34)
			Primary: 47.0 (46.5–47.5)<br>Secondary: 47.9 (47.4–48.5)<br>Tertiary: 51.2 (49.6–52.7)		Primary: 49.7 (49.1–50.2)<br>Secondary: 50.9 (50.4–51.4)<br>Tertiary: 52.0 (50.9–53.1)		Primary: 52.2 (51.0–53.4)<br>Secondary: 54.9 (54.3–55.4)<br>Tertiary: 55.2 (53.1–57.4)												Primary: 5.19 (3.52–6.85)<br>Secondary: 6.95 (5.87–8.02)<br>Tertiary: 3.23 (–0.04–6.51)
Sundberg 2016	77	SWEOLD<br>T1: 1992<br>T2: 2002<br>T3: 2004<br>T4: 2011	8.2	10.7	8.9	10.8	9.3	11.4	9.9	11.8									Men: 1.7 [d]<br>Women: 1.1 [d]
		SHARE:<br>T1: 2004<br>T2: 2011	9.3	11.4	9.9	11.8													Men: 0.6 [d]<br>Women: 0.4 [d]
Yokota 2016	15	T1: 2001<br>T2: 2004<br>T3: 2008	60.5	66.4	61.4	67	62.1	67.4											Men: 1.6 [d]<br>Women: 1.0[d]

[a]95% confidence intervals in brackets where reported; [b] Trends reported by high, medium and low education; [c]Trends not reported by sex; [d]Change estimate not reported, and thus calculated by us (T^2-T^1)

Table 5b. Summary of healthy life expectancy trends data for studies from Europe[a]

Study	Trend	At age	Time points	T1		T2		T3		T4		T5		T6		T7		T8		Change between first and last time point
				M	F	M	F	M	F	M	F	M	F	M	F	M	F	M	F	
Deeg 2018	% life in good physical health	65	T1: 1993<br>T2: 1996<br>T3: 1999<br>T4: 2002<br>T5: 2006<br>T6: 2009<br>T7: 2012<br>T8: 2016	46.6	28.7	37.6	26.0	33.6	24.5	32.4	22.7	32.7	21.2	30.5	20.2	22.8	14.9	25.0	18.9	Men: -21.6<br>Women: -9.8
	HLE: Physical (years)			6.9	5.5													4.7	4.0	Men: -2.2[c]<br>Women: -1.5[c]
Gheorghe 2016[b]	Quality Adjusted Life Expectancy	25	T1: 2001<br>T2: 2011	High: 44.08<br>Med: 40.82<br>Low: 36.71	High: 45.79<br>Med: 44.2<br>Low: 39.51	High:46.93<br>Med: 43.83<br>Low: 38.84	High: 48.43<br>Med: 45.77<br>Low: 41.31													MEN:<br>High 2.85<br>Med: 3.01<br>Low: 2.13<br><br>WOMEN:<br>High: 2.64<br>Med: 1.57<br>Low: 1.80
		65		High: 13.42<br>Med: 12.06<br>Low: 10.73	High: 15.66<br>Med: 15.12<br>Low: 13.09	High: 15.59<br>Med: 13.97<br>Low: 12.10	High: 17.40<br>Med: 16.33<br>Low: 14.23													MEN:<br>High: 2.17<br>Med: 1.91<br>Low: 1.37<br><br>WOMEN:<br>High: 1.74<br>Med: 1.21<br>Low: 1.14
Remund 2019	HLE (years)	30	T1: 1990/94<br>T2: 1995/99<br>T3: 2000/04<br>T4: 2005/09	**data only reported in graphs, unable to extract**																Men: 4.52<br>Women: 3.09

For peer review only

Study	Trend	At age	Time points	T1		T2		T3		T4		T5		T6		T7		T8		Change between first and last time point
				M	F	M	F	M	F	M	F	M	F	M	F	M	F	M	F	
	HLE (years), educational gap between primary and tertiary attainment		T5: 2010/14	7.6	3.3	**not reported**						8.8	5.0							Change in educational gap (between primary and tertiary):<br>Men: 1.2[c]<br>Women: 1.7[c]
Reus-Pons 2017	HLE (years)	50	T1: 2001<br>T2: 2011	18.62 (18.40 - 18.84)	19.43 (19.19 - 19.68)	20.83 (20.55 - 21.10)	20.68 (20.37 - 20.99)													Men: 2.21<br>Women: 1.25
Storeng 2018[d]	HLE (years)	30	T1: 1984/86<br>T2: 1995/97<br>T3: 2006/08	31.6 (31.2–31.9)	32.6 (32.3–33.0)	34.1 (33.7–34.5)	33.9 (33.6–34.3)	38.5 (38.0–39.0)	38.0 (37.5–38.5)											Men: 6.90 (6.08–7.73)<br>Women: 5.40 (4.56–6.25)
				Primary:29.5 (29.2–29.9)<br>Secondary:34.0 (33.6–34.5)<br>Tertiary: 40.1 (38.8–41.4)		Primary:29.8 (29.4–30.3)<br>Secondary: 35.7 (35.3–36.2)<br>Tertiary: 40.3 (39.3–41.3)		Primary: 31.5 (30.6–32.4)<br>Secondary:38.8 (38.3–39.4)<br>Tertiary: 42.8 (41.9–43.8)												Primary: 1.94 (0.68–3.20)<br>Secondary: 4.80 (3.85–5.75)<br>Tertiary: 2.76 (0.49–5.02)<br><br>Change in gap between primary and tertiary education: 0.7[c]

[a]95% confidence intervals in brackets where reported; [b]Trends reported by high, medium and low education; [c]Change estimate not reported, and thus calculated by us (T^{2}-T^{1}); [d]Trends reported by primary, secondary and tertiary education;

For peer review only

For peer review only - http://bmjopen.bmj.com/site/about/guidelines.xhtml

Table 5c. Summary of disability-free life expectancy trends data for studies from Europe (years)[a]

Study	Trend	At age	Time points	T1		T2		T3		T4		Change between first and last time point
				Male	Female	Male	Female	Male	Female	Male	Female	
Bronnum-Hansen 2017[b]	DFLE	65	T1: 2006-2007<br>T2: 2010-2011<br>T3: 2013-2014	High: 10.6 (9.2–12.1)<br>Med: 9.4 (8.3–10.5)<br>Low: 7.4 (5.9–8.9)	High: 12.5 (10.5–14.5)<br>Med: 9.5 (8.1–10.9)<br>Low: 8.8 (7.7–9.9)	High: 10.6 (9.2–12.0)<br>Med: 9.3 (8.2–10.4)<br>Low: 7.0 (5.3–8.8)	High: 13.2 (10.9–15.6)<br>Med: 10.4 (8.9–11.8)<br>Low: 7.9 (6.6–9.3)	High: 10.9 (9.9–12.0)<br>Med: 9.6 (8.8–10.5)<br>Low: 8.0 (6.7–9.3)	High: 12.9 (11.2–14.6)<br>Med: 10.2 (9.0–11.4)<br>Low: 9.5 (8.5–10.5)			Difference in change in DFLE between those with the lowest and highest education<br>Men: -0.3<br>Women: -0.3
Lagergren 2017	Added years of life without ADL limitations	65+	T1: 1980/85<br>T2: 1994/99<br>T3: 2006/11	**data not reported**								Men: 4.0<br>Women: 3.14
	Added years of life without mobility limitations			**data not reported**								Men: 4.21<br>Women: 3.69
Renard 2019	DFLE	25	T1: 2001<br>T2: 2011	Low: 36.34<br>Med: 41.40<br>High: 42.85	Low: 39.32<br>Med: 43.37<br>High: 48.62	Low:37.02<br>Med: 42.05<br>High: 47.49	Low:35.54<br>Med:42.98<br>High: 48.98					Difference in change in DFLE between levels of education (ref high education) change over time<br>Men:<br>Low: 3.96<br>Med: 3.99

For peer review only - http://bmjopen.bmj.com/site/about/guidelines.xhtml

Study	Trend	At age	Time points	T1		T2		T3		T4		Change between first and last time point
				Male	Female	Male	Female	Male	Female	Male	Female	
												Women:<br>Low: 4.14<br>Med: 0.76
Storeng 2018[e]	DFLE	30	T1: 1984/86<br>T2: 1995/97<br>T3: 2006/08	25.9 (25.6–26.2)	28.8 (28.6–29.1)	28.5 (28.1–28.8)	29.4 (29.0–29.7)	28.6 (28.2–29.0)	29.2 (28.8–29.6)			Men: 2.71 (2.01–3.42)<br>Women: 0.33 (–0.40–1.06)
				Primary: 25.0 (24.7–25.3)<br>Secondary: 28.8 (28.5–29.2)<br>Tertiary: 33.6 (32.5–34.7)		Primary: 25.3 (24.9–25.7)<br>Secondary: 30.1 (29.7–30.5)<br>Tertiary: 33.0 (32.3–33.8)		Primary: 22.5 (21.7–23.2)<br>Secondary: 29.1 (28.6–29.5)<br>Tertiary: 33.2 (32.4–34.1)				Primary: –2.53 (–3.61–1.45)<br>Secondary: 0.23 (–0.57–1.03)<br>Tertiary: –0.32 (–2.23–1.60)
Sundberg 2016	DFLE	77	SWEOLD<br>T1: 1992<br>T2: 2002<br>T3: 2004<br>T4: 2011	6.7 (6.3–7.1)	6.9 (6.4–7.4)	6.9 (6.4–7.3	7.0 (6.5–7.5)	7.4 (7.0–7.9)	7.9 (7.4–8.4)	7.8 (7.2–8.3)	8.5 (8.0–9.0	Men: 1.1<br>Women: 1.6
			SHARE:<br>T1: 2004<br>T2: 2011	7.5 (7.0–8.0	7.9 (7.3–8.6	7.6 (7.0–8.1)	9.2 (8.5–9.8)					Men: 0.1<br>Women: 1.3
Yokota 2019	DFLE	15	T1: 2001<br>T2: 2004<br>T3: 2008	54.1	56.0	54.9	56.2	54.8	55.3			Men: 0.7<br>Women: -0.7

[a]95% confidence intervals in brackets where reported; [b]Stratified by high, medium and low education; [c]Not stratified by sex; [d]Change estimate not reported, and thus calculated by us (T^2-T^1) [e]Second row stratified by primary, secondary and tertiary education for men and women combined

For peer review only

Table 5d. Summary of disability prevalence trends data for studies from Europe

Study	Trend	Time points	Change between first and last time point[a]
Lagergren 2017	Proportion reporting dependency in activities of daily living, 65-69	T1:1980/85<br>T2: 1994/95<br>T3: 2006/11	Men:-1.5<br>Women:-3.7
	Proportion reporting dependency in activities of daily living, 70-74		Men:-5.6<br>Women:-3.5
	Proportion reporting dependency in activities of daily living, 75-79		Men:-10.7<br>Women:-7.1
	Proportion reporting dependency in activities of daily living, 80-84		Men: -11.5<br>Women: -14.0
	Proportion reporting dependency in activities of daily living, 85+		Men:-20.3<br>Women: -10.5
	Proportion reporting mobility limitations, 65-69		Men:-6.5<br>Women: -8.3
	Proportion reporting mobility limitations, 70-74		Men: -10.9<br>Women: -8.5
	Proportion reporting mobility limitations, 75-79		Men: -15.7<br>Women: -14.8
	Proportion reporting mobility limitations, 80-84		Men: -15.1<br>Women: -19.4
	Proportion reporting mobility limitations, 85+		Men: -16.1<br>Women: -17.5

[a]Change estimate not reported, and thus calculated by us (T^2-T^1)

For peer review only

For peer review only - http://bmjopen.bmj.com/site/about/guidelines.xhtml

Table 6a. Summary of life expectancy trends data for studies from Japan and Korea (years)[a]

Study	At age	Time points	T1		T2		T3		T4		T5		T6		T7		T8		Change between first and last time point
			M	F	M	F	M	F	M	F	M	F	M	F	M	F	M	F	
Jo 2019	0	T1: 2005<br>T2: 2007<br>T3: 2008<br>T4: 2009<br>T5: 2010<br>T6: 2011<br>T7: 2012	75.13	81.87	76.13	82.71	76.54	83.27	76.99	83.74	77.19	84.04	77.64	84.42	77.94	84.61	78.51	85.02	Men: 3.38[b]<br>Women: 3.15[b]
Lee 2016	0	T1: 2005<br>T2: 2008<br>T3: 2011	75.1	81.8	76.5	83.2	77.6	84.4											Men: 2.5[b]<br>Women: 2.6[b]
	65		15.8	19.8	16.6	21.0	17.4	21.8											Men: 1.6[b]<br>Women: 2.0[b]
	85		5.1	6.2	5.2	6.5	5.4	6.9											Men: 0.3[b]<br>Women: 0.7[b]
Sugawara 2016	0	T1: 2000<br>T2: 2010	77.6	84.7	79.5	86.4													Men: 1.9<br>Women:1.7
Tokudome 2016	0	T1: 1990<br>T2: 1995<br>T3: 2000<br>T4: 2005<br>T5: 2010<br>T6: 2013	76.04 (75.98–76.10)	81.96 (81.86–82.05)	76.45 (76.14–76.57)	82.84 (82.62–82.94)	77.55 (77.53–77.58)	84.32 (84.29–84.35)	78.66 (78.60–78.71)	85.48 (85.41–85.54)	79.34 (79.31–79.36)	85.09 (86.06–86.11)	80.05 (79.26–80.84)	86.39 (85.74–87.12)					Men: 4.01[b]<br>Women: 4.43[b]

[a]95% confidence intervals in brackets where reported; [b]Change estimate not reported, and thus calculated by us (T^2-T^1)

For peer review only

For peer review only - http://bmjopen.bmj.com/site/about/guidelines.xhtml

Table 6b. Summary of healthy life expectancy trends data for studies from Japan and Korea (years)[a]

Study	At age	Time points	T1		T2		T3		T4		T5		T6		T7		T8		Change between first and last time point
			M	F	M	F	M	F	M	F	M	F	M	F	M	F	M	F	
Jo 2019	0	T1: 2005<br>T2: 2007<br>T3: 2008<br>T4: 2009<br>T5: 2010<br>T6: 2011<br>T7: 2012	70.8	74.7	71.86	73.92	72.62	75.18	73.4	76.65	73.87	77.21	74.33	76.53	74.71	78.3	74.83	78.14	Men: 4.03<br>Women: 3.44
Lee 2016	0	T1: 2005<br>T2: 2008<br>T3: 2011	64.4	67.7	64.0	67.3	65.8	68.9											Men: 1.4[b]<br>Women: 1.2[b]
	65		9.5	11.5	10.4	12.8	11.7	14.1											Men: 2.2[b]<br>Women: 2.6[b]
	85		2.6	3.8	3.3	4.7	4.0	5.2											Men: 1.4[b]<br>Women: 1.4[b]
Tokudome 2016	0	T1: 1990<br>T2: 1995<br>T3: 2000<br>T4: 2005<br>T5: 2010<br>T6: 2013	68.09 (65.83–70.11)	72.24 (69.38–74.77)	68.44 (66.07–70.58)	72.92 (70.05–75.49)	69.08 (66.67–71.24)	73.95 (70.94–76.55)	69.89 (67.31–72.12)	74.77 (71.66–77.46)	70.78 (68.20–73.06)	75.41 (72.34–78.23)	71.11 (68.50–73.57))	75.56 (72.46–78.42)					Men: 3.02[b]<br>Women: 3.32[b]

[a]95% confidence intervals in brackets where reported; [b]Change estimate not reported, and thus calculated by us (T^2-T^1)

For peer review only - http://bmjopen.bmj.com/site/about/guidelines.xhtml

Table 6c. Summary of disability-free life expectancy trends data for studies from Japan[a]

Study	Trend	At age	Time points	T1		T2		Change between first and last time point
				Male	Female	Male	Female	
Sugawara 2016	DFLE (years)	0	T1: 2000<br>T2: 2010	68.0	71.7	69.0	72.1	Men: 1.0<br>Women: 0.4
	% of life disability-free			87.6	84.6	86.8	83.4	Men: -0.8<br>Women: -1.2

[a]95% confidence intervals in brackets where reported;

For peer review only - http://bmjopen.bmj.com/site/about/guidelines.xhtml

Table 7a. Summary of life expectancy trends data high income countries combined from the GBD studies (years)[a]

Study author and date	At age	Time points	T1		T2		Change between first and last time point
			Male	Female	Male	Female	
GBD 2016	0	T1: 2005<br>T2: 2015	76·35 (76·33–76·37)	82·09 (82·07–82·11)	78·10 (78·05–78·15)	83·42 (83·38–83·46)	Men: 1.75[b]<br>Women: 1.33[b]
GBD 2017	0	T1: 1990<br>T2: 2016	72·64 (72·54–72·75)	79·34 (79·24–79·43)	78·27 (78·04–78·49)	83·48 (83·28–83·67)	Men: 5.63[b]<br>Women: 4.14[b]
	65		14·96 (14·90–15·01)	18·77 (18·71–18·84)	18·47 (18·32–18·60)	21·80 (21·66–21·94)	Men: 3.51[b]<br>Women: 3.03[b]
GBD 2018	0	T1: 1990<br>T2: 2017	72·8 (72·7–72·8)	79·4 (79·4–79·4)	78·4 (78·2–78·6)	83·6 (83·4–83·7)	Men: 5.6[b]<br>Women: 4.2[b]

[a]95% confidence intervals in brackets where reported; [b]Change estimate not reported in publication, and calculated by us (T^2-T^1);

For peer review only

Table 7b. Summary of healthy life expectancy trends data for high income countries combined from the GBD studies[a]

Study	Trend	At age	Time points	T1		T2		Change between first and last time point
				Male	Female	Male	Female	
GBD 2016	HLE (years)	0	T1: 2005<br>T2: 2015	67.48 (64.90–69.75)	71.13 (67.95–73.87)	68.91 (66.27–71.25)	72.21 (68.97–75.02)	Men: 1.43[b]<br>Women: 1.08[b]
GBD 2017	HLE (years)	0	T1: 1990<br>T2: 2016	64.15 (61.70–66.33)	68.40 (65.23–71.19)	68.58 (65.82–71.06)	71.61 (68.12–74.66)	Men: 4.43[b]<br>Women: 3.21[b]
		65		11.0 (10.45–12.27)	14.28 (13.06–15.39)	14.00 (12.79–15.11)	16.55 (15.09–17.85)	Men: 2.60[b]<br>Women: 2.27[b]
GBD 2018	HLE (years)	0	T1: 1990<br>T2: 2017	64.0 (61.4–66.1)	68.0 (64.7–70.8)	68.2 (65.3–70.7)	71.0 (67.5–74.0)	Men: 3.0[b]<br>Women: 4.2[b]

[a]95% confidence intervals in brackets where reported; [b]Change estimate not reported in publication, and calculated by us (T^2-T^1);

For peer review only - http://bmjopen.bmj.com/site/about/guidelines.xhtml

Search strategy as applied to Medline

Database(s): **Ovid MEDLINE(R) and In-Process & Other Non-Indexed Citations** 1946 to September 13, 2019

#	Searches	Results
1	Life Expectancy/	16893
2	Longevity/	20149
3	Needs assessment/	28915
4	((expectancy or Dependen* or disabilit*) adj2 years).ti,ab.	968
5	(expectanc* adj5 disabilit*).ti,ab.	406
6	(Care need* adj2 years).ti,ab.	13
7	(healthy life years or "years of healthy life").ti,ab,kw.	260
8	or/1-7	65417
9	exp Age Factors/ or Race Factors/ or Sex Factors/	631787
10	Time Factors/	1159988
11	exp Forecasting/	82762
12	Life Tables/	6387
13	td.fs.	367819
14	or/9-13	2116951
15	8 and 14	14752
16	Life Expectancy/td [Trends]	2146

For peer review only - http://bmjopen.bmj.com/site/about/guidelines.xhtml

17	Needs Assessment/td [Trends]	249
18	((life expectancy or expected years or care need*) adj2 (trend* or direction* or factor* or forecast*)).ti,ab.	334
19	or/16-18	2649
20	15 or 19	14962
21	limit 20 to (humans and yr="2016 -Current")	1661
22	letter/	1034636
23	Editorial/	498100
24	News/	196781
25	exp Historical Article/	390531
26	Anecdotes as Topic/	4730
27	Comment/	799622
28	(letter or comment$).ti.	138712
29	or/22-28	2365158
30	21 not 29	1502

For peer review only - http://bmjopen.bmj.com/site/about/guidelines.xhtml

Supplementary materials Figure 1. Expansion and compression of poor health/disability by country and time period

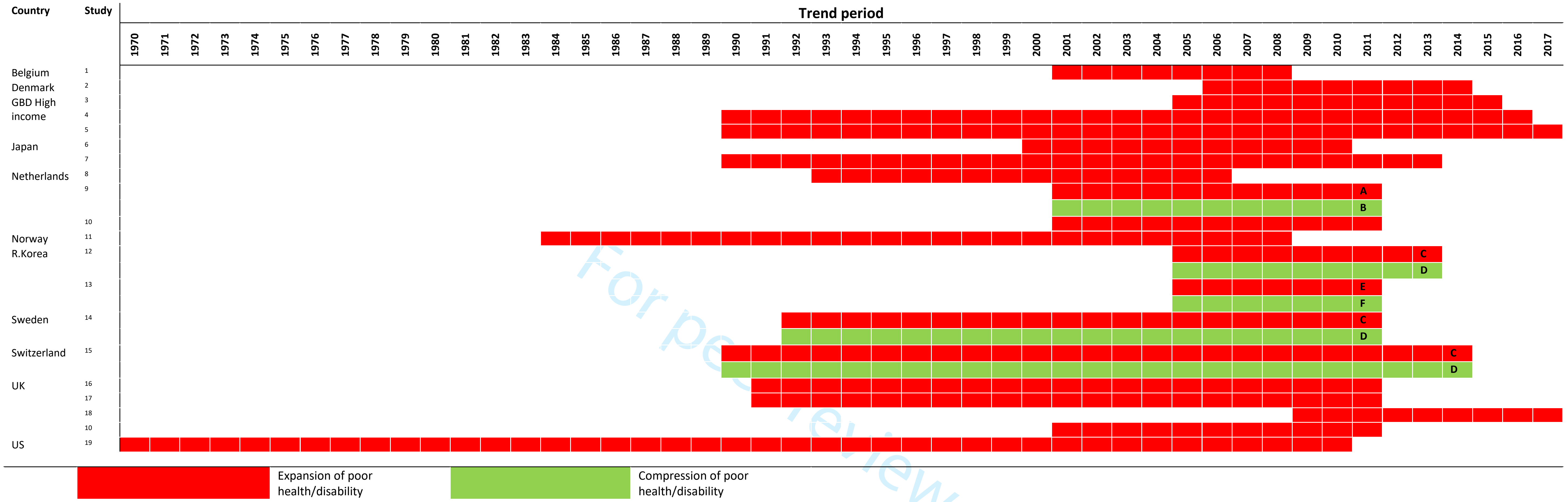

__Where multiple studies in countries or conclusions differ between sub-groups:__ [a]Men, 25 and 65, all levels of education; [b]Women (age 25, all levels of education; age 65, high and low education); [c]Men; [d]Women; [e]At birth; [f]Age 65 and 86

For peer review only

PRISMA 2009 Checklist

Section/topic	#	Checklist item	Reported on page #
**TITLE**			
Title	1	Identify the report as a systematic review, meta-analysis, or both.	1
**ABSTRACT**			
Structured summary	2	Provide a structured summary including, as applicable: background; objectives; data sources; study eligibility criteria, participants, and interventions; study appraisal and synthesis methods; results; limitations; conclusions and implications of key findings; systematic review registration number.	2
**INTRODUCTION**			
Rationale	3	Describe the rationale for the review in the context of what is already known.	3-4
Objectives	4	Provide an explicit statement of questions being addressed with reference to participants, interventions, comparisons, outcomes, and study design (PICOS).	4
**METHODS**			
Protocol and registration	5	Indicate if a review protocol exists, if and where it can be accessed (e.g., Web address), and, if available, provide registration information including registration number.	NA
Eligibility criteria	6	Specify study characteristics (e.g., PICOS, length of follow-up) and report characteristics (e.g., years considered, language, publication status) used as criteria for eligibility, giving rationale.	4-5
Information sources	7	Describe all information sources (e.g., databases with dates of coverage, contact with study authors to identify additional studies) in the search and date last searched.	4
Search	8	Present full electronic search strategy for at least one database, including any limits used, such that it could be repeated.	4
Study selection	9	State the process for selecting studies (i.e., screening, eligibility, included in systematic review, and, if applicable, included in the meta-analysis).	5-6
Data collection process	10	Describe method of data extraction from reports (e.g., piloted forms, independently, in duplicate) and any processes for obtaining and confirming data from investigators.	7
Data items	11	List and define all variables for which data were sought (e.g., PICOS, funding sources) and any assumptions and simplifications made.	4-5
Risk of bias in individual studies	12	Describe methods used for assessing risk of bias of individual studies (including specification of whether this was done at the study or outcome level), and how this information is to be used in any data synthesis.	6-7
Summary measures	13	State the principal summary measures (e.g., risk ratio, difference in means).	7-8
Synthesis of results	14	Describe the methods of handling data and combining results of studies, if done, including measures of consistency (e.g., I^2) for each meta-analysis.	7-8

For peer review only - http://bmjopen.bmj.com/site/about/guidelines.xhtml

PRISMA 2009 Checklist

Section/topic	#	Checklist item	Reported on page #
Risk of bias across studies	15	Specify any assessment of risk of bias that may affect the cumulative evidence (e.g., publication bias, selective reporting within studies).	NA
Additional analyses	16	Describe methods of additional analyses (e.g., sensitivity or subgroup analyses, meta-regression), if done, indicating which were pre-specified.	NA
**RESULTS**			
Study selection	17	Give numbers of studies screened, assessed for eligibility, and included in the review, with reasons for exclusions at each stage, ideally with a flow diagram.	8, and figure 1
Study characteristics	18	For each study, present characteristics for which data were extracted (e.g., study size, PICOS, follow-up period) and provide the citations.	Supplement materials table 1
Risk of bias within studies	19	Present data on risk of bias of each study and, if available, any outcome level assessment (see item 12).	Supplementary materials table 2
Results of individual studies	20	For all outcomes considered (benefits or harms), present, for each study: (a) simple summary data for each intervention group (b) effect estimates and confidence intervals, ideally with a forest plot.	Table 3, Supplementary materials tables 3-7
Synthesis of results	21	Present results of each meta-analysis done, including confidence intervals and measures of consistency.	NA – no meta analysis
Risk of bias across studies	22	Present results of any assessment of risk of bias across studies (see Item 15).	Supplementary materials table 2
Additional analysis	23	Give results of additional analyses, if done (e.g., sensitivity or subgroup analyses, meta-regression [see Item 16]).	NA
**DISCUSSION**			
Summary of evidence	24	Summarize the main findings including the strength of evidence for each main outcome; consider their relevance to key groups (e.g., healthcare providers, users, and policy makers).	12-15
Limitations	25	Discuss limitations at study and outcome level (e.g., risk of bias), and at review-level (e.g., incomplete retrieval of identified research, reporting bias).	13-14
Conclusions	26	Provide a general interpretation of the results in the context of other evidence, and implications for future	12-15

For peer review only - http://bmjopen.bmj.com/site/about/guidelines.xhtml

PRISMA 2009 Checklist

		research.	
**FUNDING**			
Funding	27	Describe sources of funding for the systematic review and other support (e.g., supply of data); role of funders for the systematic review.	Title page

From: Moher D, Liberati A, Tetzlaff J, Altman DG, The PRISMA Group (2009). Preferred Reporting Items for Systematic Reviews and Meta-Analyses: The PRISMA Statement. PLoS Med 6(7): e1000097. doi:10.1371/journal.pmed1000097

For more information, visit: **www.prisma-statement.org**.

For peer review only

BMJ Open

Trends in health expectancies: a systematic review of international evidence

Journal:	*BMJ Open*
Manuscript ID	bmjopen-2020-045567.R2
Article Type:	Original research
Date Submitted by the Author:	31-Mar-2021
Complete List of Authors:	Spiers, Gemma; Newcastle University, Population & Health Sciences Institute<br>Kunonga, Tafadzwa; Newcastle University, Population & Health Sciences Institute<br>Beyer, Fiona ; Newcastle University, Population & Health Sciences Institute<br>Craig, Dawn; Newcastle University, Population & Health Sciences Institute<br>Hanratty, Barbara; Newcastle University, Population & Health Sciences Institute<br>Jagger, Carol; Newcastle University, Population & Health Sciences Institute
**Primary Subject Heading**:	Health policy
Secondary Subject Heading:	Epidemiology
Keywords:	Health policy < HEALTH SERVICES ADMINISTRATION & MANAGEMENT, Organisation of health services < HEALTH SERVICES ADMINISTRATION & MANAGEMENT, GERIATRIC MEDICINE

SCHOLARONE™
Manuscripts

I, the Submitting Author has the right to grant and does grant on behalf of all authors of the Work (as defined in the below author licence), an exclusive licence and/or a non-exclusive licence for contributions from authors who are: i) UK Crown employees; ii) where BMJ has agreed a CC-BY licence shall apply, and/or iii) in accordance with the terms applicable for US Federal Government officers or employees acting as part of their official duties; on a worldwide, perpetual, irrevocable, royalty-free basis to BMJ Publishing Group Ltd ("BMJ") its licensees and where the relevant Journal is co-owned by BMJ to the co-owners of the Journal, to publish the Work in this journal and any other BMJ products and to exploit all rights, as set out in our licence.

The Submitting Author accepts and understands that any supply made under these terms is made by BMJ to the Submitting Author unless you are acting as an employee on behalf of your employer or a postgraduate student of an affiliated institution which is paying any applicable article publishing charge ("APC") for Open Access articles. Where the Submitting Author wishes to make the Work available on an Open Access basis (and intends to pay the relevant APC), the terms of reuse of such Open Access shall be governed by a Creative Commons licence – details of these licences and which Creative Commons licence will apply to this Work are set out in our licence referred to above.

Other than as permitted in any relevant BMJ Author's Self Archiving Policies, I confirm this Work has not been accepted for publication elsewhere, is not being considered for publication elsewhere and does not duplicate material already published. I confirm all authors consent to publication of this Work and authorise the granting of this licence.

For peer review only

Trends in health expectancies: a systematic review of international evidence

Dr Gemma Spiers,[a]* gemma-frances.spiers@newcastle.ac.uk

Ms Tafadzwa Patience Kunonga[a]

Ms Fiona Beyer[b]

Professor Dawn Craig[b]

Professor Barbara Hanratty[a]

Professor Carol Jagger[a]

[a] Population and Health Sciences Institute, Newcastle University, Campus for Ageing & Vitality, Newcastle upon Tyne, NE4 5PL

[b] Population and Health Sciences Institute, Newcastle University, Baddiley-Clark, Newcastle upon Tyne, NE2 4AX

*Corresponding author

Word count: 3301 excluding abstract, references, tables and figures

Number of references: 43

Number of tables and figures: 4

For peer review only

Trends in health expectancies: a systematic review of international evidence

OBJECTIVES

A clear understanding of whether increases in longevity are spent in good health is necessary to support ageing, health and care-related policy.

DESIGN

We conducted a systematic review to update and summarise evidence on trends in health expectancies, in OECD high-income countries.

DATA SOURCES

Four electronic databases (MEDLINE, 1946 to September 19th 2019; Embase 1980 to 2019 Week 38; Scopus 1966 to 22nd September 2019, Health Management Information Consortium, 1979 to September 2019), and the UK Office for National Statistics website (November 2019).

ELIGIBILITY CRITERIA

English language studies published from 2016 that reported trends in healthy, active, and/or disability-free life expectancy in an OECD high-income country.

DATA EXTRACTION AND SYNTHESIS

Records were screened independently by two researchers. Study quality was assessed using published criteria designed to identify sources of bias in studies reporting trends, and evidence summarised by narrative synthesis.

FINDINGS

Twenty-eight publications from eleven countries were included, covering periods from six to 40 years, between 1970 and 2017. In most countries, gains in healthy and disability-free life expectancy do not match the growth in total life expectancy. Exceptions were demonstrated for women in Sweden, where there were greater gains in disability-free years than life expectancy. Gains in healthy and disability-free life expectancy were greater for men than women in most countries except the US (age 85), Japan (birth), Korea (age 65) and Sweden (age 77).

CONCLUSION
An expansion of disability in later life is evident in a number of high-income countries, with implications for the sustainability of health and care systems. The recent COVID-19 pandemic may also impact health expectancies in the longer term.

Keywords: Trends, Life Expectancy, Disability, Health, Ageing

For peer review only

Summary Box

Strengths and limitations of this study

- To our knowledge, this is the first systematic review using reproducible methods to synthesise evidence about health expectancies and report quantitative comparisons of life and health expectancies to differentiate compression or expansion of morbidity.
- We used analyses published from 2016 to focus on the most contemporary evidence in trends.
- The quality of evidence was judged using criteria designed to assess threats to the validity of trends over time. However, due to the absence of methodological detail reported, it was not possible to give a clear judgement of study quality and bias for 10 of 28 studies included in the review.

For peer review only

For peer review only - http://bmjopen.bmj.com/site/about/guidelines.xhtml

BACKGROUND

Populations are ageing worldwide. Globally, the proportion of those aged 65 and over has increased by 9 per cent in the last two decades, and is expected to grow by a further 16% by 2050.[1] This demographic shift will require societies to adapt. If longer lives are spent in poor health, governments face the challenge of providing accessible, high quality and sustainable long-term care.[2-4] The growth in life expectancy is a positive, but with this comes a responsibility to ensure people have the support they need as they age, and to facilitate ageing in place.[5]

In 2019, the World Health Organisation renewed its commitment to support countries to achieve longer and healthier lives with the Decade of Healthy Ageing 2020-2030 strategy.[6] A critical part of achieving longevity is understanding whether longer lives are typified by more years spent in good health (compression of disability) or poor health (expansion of disability). This has important implications for the provision of health and care services to respond to the needs of people as they age. It is therefore crucial to keep abreast of trends, specifically how the growth in life expectancy is matched by a growth in years spent in good health. Metrics to assess this most commonly include *healthy life expectancy* and *disability-free life expectancy*. Both provide an estimate of life expectancy spent in good health, but differ slightly with respect to their measurement. Healthy life expectancy tends to rely on single item questions of self-reported health, and is thus subject to fluctuations as expectations of health change over time.[7] Disability-free life expectancy is often calculated from multiple items about activity limitations and/or dependencies,[8] and therefore does not bear the same limitations as that of healthy life expectancy.

Previous reviews have summarised trends in total, healthy and disability-free life expectancy, the most recently in 2016.[8, 9] Typically, such evidence shows that whilst people are living longer, gains in life expectancy are not consistently matched by a growth in the number of years lived in good health and free of disability. Nevertheless, this is an evolving evidence base requiring ongoing scrutiny. Longitudinal datasets, which often form the bedrock of the analysis of these trends, are continually acquiring new data, whilst in the UK, the Office of National Statistics (ONS) updates and publishes trends yearly. Regardless, the pressing policy relevance of these issues globally warrants an updated overview of trends in health expectancies. Understanding these issues in a global context is equally

For peer review only - http://bmjopen.bmj.com/site/about/guidelines.xhtml

important in order to identify, and learn from, those countries whose populations are living healthier for longer. To obtain an up to date understanding of population trends in life expectancy and healthy ageing, we undertook a systematic review to a) synthesise evidence about trends in health expectancies in high-income countries, and b) assess whether such trends are keeping pace with total life expectancy.

METHODS

The methods used for this systematic review are reported according to the PRISMA checklist and guidance.[10]

Search strategy

A search strategy was developed using the concepts [*life expectancy*] AND [*trends* OR *impacting factors*]. The search was designed in MEDLINE using thesaurus headings and title, abstract and keyword field terms, and these elements translated to other databases. Electronic searches were carried out in MEDLINE (OVID) (1946 to September 19th 2019), Embase (OVID) 1980 to 2019 Week 38, Scopus (1966 to 22nd September 2019) and Health Management Information Consortium (OVID) (1979 to September 2019), in October 2019 (see the supplementary materials for the strategy applied to MEDLINE). As this systematic review updated previous reviews,[8, 9] searches were limited to studies published from 2016. The ONS website was also hand searched (November 2019) for reports published from 2016.

Review criteria

Review criteria are summarised in table 1. Studies were included if they examined trends (i.e. more than one time point) in health expectancies. Eligible health expectancies were general healthy life expectancy, disability free life expectancy, active life expectancy, health-related quality adjusted life expectancy, and health adjusted life expectancy. We did not include healthy cognitive life expectancy, dementia free life expectancy, or life expectancy with diseases. Studies must be conducted in an Organisation for Economic Co-operation and Development (OECD) high-income country.[11] OECD high-income countries were selected for this review for comparability. Studies reporting trends in life expectancy *only* were ineligible. Where studies also reported prevalence of disability, dependency or self-rated health alongside the above outcomes, these were reported for context. For ONS reports,

For peer review only - http://bmjopen.bmj.com/site/about/guidelines.xhtml

For peer review only

the most up-to-date analyses were included, with reports excluded if they were superseded by a more recently published analysis of the same data.

TABLE 1 HERE

Study selection

Titles and abstracts of all records were screened for relevance. The full texts of publications selected as relevant were retrieved and assessed for inclusion against the review criteria. Both stages of screening were undertaken independently by two researchers, with disagreements resolved through consensus.

Quality appraisal

Study quality and bias were assessed using an adapted version of previously published criteria for studies reporting trends.[12] This approach assesses 'threats to the validity of comparisons over time' (p. 3140). Following expert advice, we also judged two further criteria: whether the trend analysis used more than two time points, and the % of non-response in repeated cross-section surveys. Criteria were rated as *good*, *fair* or *poor*, according to the parameters summarised in table 2. Where study publications did not report the required information to assess quality, other associated publications (e.g. methodological and technical reports for the datasets used) were consulted, and we attempted to contact all authors and/or administrators of the datasets used. If the required information was not available from these sources, the criterion was assessed as *unclear*.

Using the assessments (good, fair, poor or unclear) for each criterion, studies were given a summary rating of quality. As studies were often based on summaries of cross-sectional data, they did not typically report information to assess the criterion *proportion lost to follow up.* We also struggled to find information to assess the criteria *proportion of proxy interviews* and *proportion of missing data.* We therefore based our summary judgement on four criteria: the comparability of methods over time, quality of the outcome measure, response rate or loss to follow up, and whether more than two time points were used. Studies with all four criteria rated good received a summary judgment of good. Studies with one or more criteria rated poor received a summary judgment of poor. The remainder were rated fair.

For peer review only - http://bmjopen.bmj.com/site/about/guidelines.xhtml

Where studies used multiple outcomes and were given different judgements for each, this was noted in the summary rating.

TABLE 2 HERE

Data extraction and synthesis

Study details (author, publication date, country, study design) and trend estimates (for each time point measured, and the change between the first and last time point) were extracted onto an Excel spreadsheet by one researcher. Fifty percent of studies were checked for accuracy by a second researcher. Where publications did not report the change between the first and last time point, we calculated this by subtracting the first time point estimate from the last time point estimate.

A narrative synthesis was used to summarise evidence on trends by outcome (e.g. healthy life expectancy, disability-free life expectancy, disability or dependency prevalence), supported by data summary tables. Estimates of changes in each health expectancy were compared to changes in life expectancy. This comparison provides evidence of whether there had been an expansion (health expectancy gains are smaller than life expectancy gains) or compression (health expectancy gains are equivalent to or greater than life expectancy gains) of disability and ill health.

Patient and public involvement

This review was requested by our funder within a timescale that did not allow for meaningful public and patient involvement.

FINDINGS

Twenty-eight studies met the review criteria (figure 1, and supplementary materials table 1). Seven studies reported trends in the UK,[7, 8, 13-17] including England,[7, 8, 14] England & Wales,[13, 15, 17] and each of the four devolved countries and the UK as a whole.[16] Two of the UK studies were ONS reports.[15, 16] The remaining studies reported trends in Belgium,[17-19] Canada,[20] Denmark,[21] Japan,[22, 23] the Netherlands,[24, 25] Norway,[26] Republic of Korea,[27, 28] Sweden,[29, 30] Switzerland,[31] and the US.[32-35] Three Global Burden of Disease studies were included, which reported trends across

For peer review only - http://bmjopen.bmj.com/site/about/guidelines.xhtml

multiple countries, and high income countries combined.[36-38] For these three studies, we used data for all high income countries combined.

The assessment of study quality is detailed in supplementary materials table 2. Three studies were rated good,[20, 34, 35] eleven were rated fair,[8, 13, 14, 18, 19, 21, 22, 24, 30, 32, 33], one was rated good *and* fair (as it used two outcome measures that each received a different quality rating),[7] and three were rated poor.[26, 29, 31] Ten studies were rated unclear due to a lack of information required to assess quality on the four key criteria.[15-17, 23, 25, 27, 28, 36-38] As majority large proportion of studies received a summary rating of unclear, the synthesis below does not prioritise evidence with a higher quality rating. Instead, the summary judgement is provided in supplementary materials table 2 for the reader's reference.

FIGURE 1 HERE

Across most studies, trends demonstrated a growth in health expectancies, with gains typically greater for men than women (supplementary materials tables 3a-d, 4a-f, 5a-d, 6a-c, 7a-b). Exceptions where changes were greater for women include the US (age 85),[33] Japan (birth),[23] Korea (age 65),[28] and Sweden (age 77).[30] One analysis of UK data over the more recent period of 2009/12 to 2015/17 also indicated that healthy life expectancy declined for women by 0.2 years.[15] Disability-free life expectancy at age 20 - 64 years also declined by 0.6 years between 1970 and 2010 for women in the US in one study.[33]

Is there evidence of an expansion or compression of poor health and disability?

Table 3 summarises the change in each life expectancy and health expectancy across trend periods for each study where comparisons were possible. The comparison does not include studies where: the metric of change was not comparable between life expectancy and health expectancy;[18, 20] trends were reported as a slope of index inequality only;[16] only forecasts were reported;[13, 14, 32] and life expectancy is reported only in a graph.[29, 34, 35]

For peer review only - http://bmjopen.bmj.com/site/about/guidelines.xhtml

TABLE 3 HERE

There is evidence from observed (i.e. not forecasted) trends in the UK that changes in health expectancies were smaller than those in life expectancy. This is consistent with evidence of a reduction in the proportion of life spent without disability in the UK. This pattern was also observed in: all high income countries combined in the Global Burden of Disease studies,[36-38] Norway,[26] Belgium,[19] Japan,[22, 23] and the US[33] for all, and in Switzerland [31] and Sweden [30] for men but not women. This points to evidence of an expansion of disability in a number of high-income countries, although not always consistently between men and women.

Gains in disability-free life expectancy were greater than gains in life expectancy for women in Sweden, a finding that was evident in two different datasets.[30] This indicates a compression of disability for women in Sweden. In another study, gains in life expectancy and healthy life expectancy were similar for women in Switzerland,[31] which might also suggest a compression of disability. However, this finding should be interpreted with some caution: the quality assessment highlighted a potential threat to the validity of the trend due to changes in the phrasing of, and response items for, the outcome measure between data waves.

Evidence from Korea and the Netherlands was less certain. Contrasting findings between studies gave an unclear picture about whether these countries had experienced a compression or expansion of poor health. For example, in one study from Korea, changes in quality adjusted life expectancy at birth were greater than changes in life expectancy.[27] Another study, using the same data but a different measure (healthy life expectancy), reported that gains at birth still lagged behind those for life expectancy, although gains in healthy life expectancy at age 65 and 85 were slightly higher than gains in life expectancy over the study period.[28] This contrast may reflect the difference in measures; Jo and colleagues suggest that quality adjusted life expectancy may be overestimated using the EQ-5D-3L, which forms the basis of this measure.[27]

Three studies from the Netherlands also offered inconsistent findings: this may reflect differences in the trend periods, age at which expectancies were estimated and the measures used.[17, 24, 25] For example, Reus-Pons and colleagues demonstrated gains in

For peer review only - http://bmjopen.bmj.com/site/about/guidelines.xhtml

healthy life expectancy were smaller than gains in total life expectancy at age 50, over a ten year period (2001-2011). Deeg and colleagues examined physical healthy life expectancy over a 23 year period (1993-2016): a decline was observed, whilst total life expectancy increased. Gheorghe and colleagues estimated quality adjusted life expectancy at age 25 and 65, and stratified by educational attainment. These trends indicated an expansion of poor health at age 25 and 65 for men at all levels of educational attainment, but a compression of poor health for women at age 25 (all levels of education) and 65 (high and low education).

DISCUSSION

This systematic review was undertaken to update our current understanding of trends in health expectancies in OECD high-income countries. The principal finding is that changes in health expectancies have not kept pace with the growth in life expectancy in a number of high income countries. One clear exception was Sweden, where gains in women's disability-free life expectancy were greater than gains in life expectancy over a period of almost 20 years. This was a finding evidenced from two different datasets within Sundberg and colleagues' study (SWEOLD: 1992-2011; SHARE: 2004-2011). This finding contrasts with an earlier review, which found evidence of a compression of disability for men, but not women, in Sweden.[9] This has now been reversed with an expansion of disability for men.[30] Sundberg and colleagues attribute the compression of disability for women to improved health for women but not men, whilst men were living longer due to falling deaths from cardiovascular disease.

The equivalence of total and healthy life expectancy for women in Switzerland also indicates a compression of poor health in Switzerland, although as noted earlier, this finding should be interpreted with some caution. Nevertheless, based on the countries with data, our findings suggest that countries still need to make significant progress to achieve the World Health Organisation's Decade of Healthy Ageing goal of healthier, longer lives for all.

The observed expansion of disability and poor health has important implications for older people's health and care. Across many high-income countries, care services that support older people's day to day independence and quality of life are not universal but subject to payment barriers.[3, 4] Living longer with greater disability and in poor health signals a

For peer review only - http://bmjopen.bmj.com/site/about/guidelines.xhtml

need for greater policy focus on ensuring older people have timely access to care. This is a particularly timely message for policy makers in the UK, as the All Party Parliamentary Group for Longevity set out recommendations to achieve the UK Government's goal of extending healthy life by 2035 and reducing inequalities.[39] Furthermore, the finding that women are living with longer periods of disability may warrant a particular focus on how services can support women in later life. This is especially important given that women are more likely than men to experience financial insecurity in later life,[40] and thus may face greater barriers to paid-for care services.

Finally, given the high death rates in some countries as a result of the COVID-19 pandemic, and the likely long-term health consequences of the virus that are becoming apparent, further scrutiny of future trends in health expectancies is needed. A recent analysis of UK mortality data in 2020, for example, indicates that COVID has reduced average life expectancy by around a year.[41] The ways in which this may impact health expectancies has yet to be determined.

Strengths and limitations

This systematic review provides a robust picture of trends in health expectancies in high-income countries. Although previous reviews have been published,[42] a strength of our study is that we used reproducible systematic search techniques and reported quantitative comparisons to differentiate the compression or expansion of morbidity.

We chose to focus on evidence published from 2016, in order to avoid duplication of previous work and focus on contemporary evidence. A limitation of this approach is that we have omitted potentially useful studies published prior to 2016. However, this criterion has enabled a synthesis that prioritises the most recent evidence on trends in health expectancies. In addition to searching for published studies, we searched the Office for National Statistics website, the UK government body responsible for analysing data about the UK population. We did not search the equivalent national statistical bodies for every other high income country as the volume of translation this would necessitate was unfeasible. However, this is not a major shortcoming: most of the non-UK studies used nationally representative samples, and none indicated unusual or contradictory trends that required verification from other data sources.

For peer review only - http://bmjopen.bmj.com/site/about/guidelines.xhtml

A key limitation of our synthesis is that whilst we were able to make some broad comparisons between studies, a more detailed comparison of trends was not possible. This was due to the high degree of heterogeneity between studies in: the age at which the health expectancy is estimated, trend periods (years and time frame), the measures used, and the way trends were stratified. This not only prevented a more meaningful comparison, but also limited the extent to which we were able explain differences in these trends between studies and countries. A further consideration here is the comparability of the overlapping and non-overlapping trend periods within and between countries in the studies included in this review (supplementary materials figure 1). Different countries face national challenges at different times, whilst global challenges can differentially affect countries across the same time periods. Therefore, even similar trends across the same time periods may be due to different reasons.

Finally, the quality of this evidence was judged using criteria designed to assess threats to the validity of trends and the adequacy of the outcome measure.[12] For some studies, it was not possible to arrive at a clear judgement due to the absence of required methodological detail regarding survey response rates. This is an important limitation, but one that is easily addressed if future studies of health expectancy trends report the methodological detail required. Where a judgement on quality was possible, studies were generally of fair or good quality, signalling no major concerns to the validity of the evidence synthesised. The rating of poor for a minority of studies largely reflected a change in the outcome measure over time,[26, 29, 31] which may reduce comparability between observed time points. To some extent, such changes are expected as panel and longitudinal studies evolve at each new point of data collection. Such reduced comparability may undermine the validity of the trends reported in these studies. However, this should be balanced against the judgements for the other quality assessment criteria, where methods were rated favourably and indicated minimal bias (see supplementary materials). Overall, for studies where a clear judgment on quality was possible, we did not identify any studies that were particularly concerning in terms of quality and validity.

CONCLUSIONS

In a number of high-income countries, changes in health expectancies over time have not kept pace with the growth in life expectancy. That is, people are living longer but disability

For peer review only - http://bmjopen.bmj.com/site/about/guidelines.xhtml

and poor health are occupying an increasing proportion of later life. A compression of disability for women in Sweden signals some progress in achieving healthier longer lives. These findings have implications for health and care related policy, and in particular ensuring people have timely access to care in later life. Further scrutiny of health expectancies in the wake of the COVID-19 pandemic is necessary given the high death rates in some countries and the long-term health consequences of the virus that are becoming apparent.

For peer review only

Funding

National Institute for Health Research (NIHR) Policy Research Programme conducted through the NIHR Older People and Frailty Policy Research Unit, PR-PRU-1217-21502. The views expressed are those of the authors and not necessarily those of the NIHR or the Department of Health and Social Care.

Ethics Statement

Ethics approval was not required for this project, as this was a systematic review and no human participants were involved.

Author contributions

Author contributions: CJ planned the study, developed the protocol, and co-wrote the paper; GS developed the protocol, contributed to all stages of the review, and co-wrote the paper; PK developed the protocol, contributed to all stages of the review, and co-wrote the paper, FB developed the protocol, developed and undertook the searches, and co-wrote the paper; DC developed the protocol, oversaw all stages of the review, and co-wrote the paper; BH developed the protocol and co-wrote the paper.

Data availability statement

All data relevant to the study are included in the article or uploaded as supplementary information.

Competing interests: None declared.

For peer review only

REFERENCES

1 Department of Social and Economic Affairs. World Population Ageing 2019 Highlights. *United Nations* 2019.
2 Organisation for Economic and Co-Operative Development. A Good Life in Old Age? Monitoring and Improving Quality in Long-Term Care. 2013.
3 Organisation for Economic Co-operative Development. Help Wanted? Providing and Paying for Long-Term Care. Organisation for Economic and Co-operative Development 2011.
4 Organisation for Economic Co-operative Development. Public long-term care financing arrangements in OECD countries. In: Oecd, ed. *Help Wanted?*: OECD 2011.
5 Pani-Harreman KE, Bours GJJW, Zander I*, et al.* Definitions, key themes and aspects of 'ageing in place': a scoping review. *Ageing and Society* 2020:1-34.
6 World Health Organisation. Decade of Healthy Ageing 2020-2030. 2019.
7 Jagger C, Matthews FE, Wohland P*, et al.* A comparison of health expectancies over two decades in England: results of the Cognitive Function and Ageing Study I and II. *Lancet* 2016;**387**:779-86.
8 Kingston A, Wohland P, Wittenberg R*, et al.* Is late-life dependency increasing or not? A comparison of the Cognitive Function and Ageing Studies (CFAS). *Lancet* 2017;**390**:1676-84.
9 Jagger C. Trends in life expectancy and healthy life expectancy. Newcastle University Institute for Ageing and Institute of Health & Society, Government Office for Science 2015.
10 Moher D, Liberati A, Tetzlaff J*, et al.* Preferred Reporting Items for Systematic Reviews and Meta-Analyses: The PRISMA Statement. *PLoS Medicine* 2009;**6**:e1000097.
11 Group WB. Fact Sheet: OECD High-Income. *Doing Business 2019*: World Bank 2019.
12 Freedman VA, Martin LG, Schoeni RF. Recent trends in disability and functioning among older adults in the United States: a systematic review. *Jama* 2002;**288**:3137-46.
13 Guzman-Castillo M, Ahmadi-Abhari S, Bandosz P*, et al.* Forecasted trends in disability and life expectancy in England and Wales up to 2025: a modelling study. *The Lancet Public Health* 2017;**2**:e307-e13.
14 Kingston A, Comas-Herrera A, Jagger C*, et al.* Forecasting the care needs of the older population in England over the next 20 years: estimates from the Population Ageing and Care Simulation (PACSim) modelling study. *Lancet Public Health* 2018;**3**:e447-e55.
15 Office for National Statistics. Health state life expectancies, UK: 2015 to 2017. Office for National Statistics 2018.
16 Office for National Statistics. Health state life expectancies by national deprivation deciles, England and Wales: 2015 to 2017. Office for National Statistics 2019.
17 Reus-Pons M, Kibele EUB, Janssen F. Differences in healthy life expectancy between older migrants and non-migrants in three European countries over time. *International Journal of Public Health* 2017;**62**:531-40.
18 Renard F, Devleesschauwer B, Van Oyen H*, et al.* Evolution of educational inequalities in life and health expectancies at 25 years in Belgium between 2001 and 2011: a census-based study. *Arch Public Health* 2019;**77**:6.
19 Yokota RTC, Nusselder WJ, Robine JM*, et al.* Contribution of chronic conditions to gender disparities in health expectancies in Belgium, 2001, 2004 and 2008. *Eur J Public Health* 2019;**29**:82-7.
20 Steensma C, Loukine L, Choi BC. Evaluating compression or expansion of morbidity in Canada: trends in life expectancy and health-adjusted life expectancy from 1994 to 2010. *Health Promot Chronic Dis Prev Can* 2017;**37**:68-76.
21 Bronnum-Hansen H, Eriksen ML, Andersen-Ranberg K*, et al.* Persistent social inequality in life expectancy and disability-free life expectancy: Outlook for a differential pension age in Denmark? *Scand J Public Health* 2017;**45**:459-62.
22 Sugawara YM, Saito Y. Changes in disability-free life expectancy (DFLE) at birth between 2000 and 2010 across Japanese prefectures. *International Journal of Public Health* 2016;**61**:739-49.

For peer review only - http://bmjopen.bmj.com/site/about/guidelines.xhtml

23 Tokudome S, Hashimoto S, Igata A. Life expectancy and healthy life expectancy of Japan: the fastest graying society in the world. *BMC research notes* 2016;**9**:482.
24 Deeg DJH, Comijs HC, Hoogendijk EO*, et al.* 23-Year Trends in Life Expectancy in Good and Poor Physical and Cognitive Health at Age 65 Years in the Netherlands, 1993-2016. *Am J Public Health* 2018;**108**:1652-8.
25 Gheorghe M, Wubulihasimu P, Peters F*, et al.* Health inequalities in the Netherlands: trends in quality-adjusted life expectancy (QALE) by educational level. *Eur J Public Health* 2016;**26**:794-9.
26 Storeng SH, Krokstad S, Westin S*, et al.* Decennial trends and inequalities in healthy life expectancy: The HUNT Study, Norway. *Scand J Public Health* 2018;**46**:124-31.
27 Jo MW, Seo W, Lim SY*, et al.* The Trends in Health Life Expectancy in Korea according to Age, Gender, Education Level, and Subregion: Using Quality-Adjusted Life Expectancy Method. *J Korean Med Sci* 2019;**34**:e88.
28 Lee JY, Ock M, Kim SH*, et al.* Health-Adjusted Life Expectancy (HALE) in Korea: 2005-2011. *J Korean Med Sci* 2016;**31 Suppl 2**:S139-S45.
29 Lagergren M, Johnell K, Schon P*, et al.* Towards a postponement of activities of daily living dependence and mobility limitations: Trends in healthy life years in old age in Sweden. *Scand J Public Health* 2017;**45**:520-7.
30 Sundberg L, Agahi N, Fritzell J*, et al.* Trends in health expectancies among the oldest old in Sweden, 1992-2011. *Eur J Public Health* 2016;**26**:1069-74.
31 Remund A, Cullati S, Sieber S*, et al.* Longer and healthier lives for all? Successes and failures of a universal consumer-driven healthcare system, Switzerland, 1990–2014. *International Journal of Public Health* 2019.
32 Cao B. Future healthy life expectancy among older adults in the US: a forecast based on cohort smoking and obesity history. *Popul Health Metr* 2016;**14**:23.
33 Crimmins EM, Zhang Y, Saito Y. Trends Over 4 Decades in Disability-Free Life Expectancy in the United States. *Am J Public Health* 2016;**106**:1287-93.
34 Freedman VA, Spillman BC. Active life expectancy in the older US population, 1982-2011 : differences between blacks and whites persisted. 2016.
35 Freedman VA, Wolf DA, Spillman BC. Disability-Free Life Expectancy Over 30 Years: A Growing Female Disadvantage in the US Population. *Am J Public Health* 2016;**106**:1079-85.
36 Global Burden of Disease study, Collaborators H. Global, regional, and national disability-adjusted life-years (DALYs) for 359 diseases and injuries and healthy life expectancy (HALE) for 195 countries and territories, 1990-2017: a systematic analysis for the Global Burden of Disease Study 2017. *Lancet* 2018;**392**:1859-922.
37 Hay SI, Abajobir AA, Abate KH*, et al.* Global, regional, and national disability-adjusted life-years (DALYs) for 333 diseases and injuries and healthy life expectancy (HALE) for 195 countries and territories, 1990-2016: A systematic analysis for the Global Burden of Disease Study 2016. *The Lancet* 2017;**390**:1260-344.
38 Kassebaum NJ, Arora M, Barber RM*, et al.* Global, regional, and national disability-adjusted life-years (DALYs) for 315 diseases and injuries and healthy life expectancy (HALE), 1990–2015: a systematic analysis for the Global Burden of Disease Study 2015. *The Lancet* 2016;**388**:1603-58.
39 All Party Parliamentary Group for Longevity. The Health of the Nation: A Strategy for Healthier Longer Lives. All Party Parliamentary Group for Longevity, 2020.
40 Scharf T, Shaw C. Inequalities in later life. Centre for Ageing Better 2017.
41 Aburto JM, Kashyap R, Schöley J*, et al.* Estimating the burden of the COVID-19 pandemic on mortality, life expectancy and lifespan inequality in England and Wales: a population-level analysis. *Journal of Epidemiology and Community Health* 2021:jech-2020-215505.
42 J-M R, C J, Crimmins E.M ea. Trends in health expectancies. In: Jagger C CE, Saito Y, et al, ed. *International Handbook of Health Expectancies*. London: Springer 2020.

For peer review only

For peer review only - http://bmjopen.bmj.com/site/about/guidelines.xhtml

For peer review only

For peer review only - http://bmjopen.bmj.com/site/about/guidelines.xhtml

Table 1. Review criteria

**Population**	Studies must examine, trends from birth, 65 years and 85 years. Studies reporting trends from other ages were also reviewed where evidence was available. Studies must examine these trends in whole populations. Studies reporting trends in population sub-groups only (i.e. only those with heart failure) were ineligible.
**Exposure**	As this review reports evidence on life expectancy, health expectancy trends, an exposure variable was not required.
**Comparator**	Not applicable.
**Outcome(s)**	Active life expectancy, healthy life expectancy, disability-free life expectancy, health-related quality adjusted life expectancy, health-adjusted life expectancy. Studies reporting *only* life expectancy trends were ineligible. Studies must examine changes in these outcomes over time (i.e. include more than one time point). Studies that report projections/forecasts of these outcomes were also eligible.
**Study design**	Studies must use an observational design and be carried out in an OECD high-income country. The review focused on evidence from the UK with comparison to evidence from other OECD high-income countries where possible. Studies published from 2016 were eligible. ONS reports were excluded if they were not the latest release, or reported trends for a period contained within a more recent ONS publication using the same data.

For peer review only

For peer review only - http://bmjopen.bmj.com/site/about/guidelines.xhtml

Table 2. Quality assessment criteria

Criteria	Parameters
**Comparability of interview methods between time points**	Good: Identical<br>Fair: Change in mode<br>Poor: Change in disability, functioning or health outcomes
**Quality of outcome measure**	Good: Detailed multiple item measure<br>Fair: Single item global measure<br>*No criteria for poor*
**Uses more than two time points to assess trend[b]**	Good: Uses more than two time points<br>Fair: Uses only two time points<br>*No criteria for poor*
**% Response in repeated cross-sectional studies[a]**	Good: >70% response rate and <10% drop in subsequent surveys<br>Fair: <70% or >10% drop in subsequent surveys<br>*No criteria for poor*
**Loss to follow up**	Good: NA or <5%<br>Fair: 5-10%<br>Poor: >10%<br>Note: This only applies to longitudinal study designs (i.e. not independent repeat cross sections)
**Proportion of proxy interviews**	Good: <10%<br>Fair: 10-20%<br>Poor: >20%
**Proportion of missing data**	Good: <5%<br>Fair: 5-10%<br>Poor: >10%

[a] Added to the quality assessment following expert advice

For peer review only

For peer review only - http://bmjopen.bmj.com/site/about/guidelines.xhtml

Table 3. Change in life expectancy, healthy life expectance and disability-free life expectancy across all studies where reported[a]

Study	Country	Age	Trend period	Change in LE	Change in HLE	Change in DFLE
All high-income countries combined from the Global Burden of Disease studies						
GBD study 2016	All high income	0	2005, 2015	Men: 1.75<br>Women: 1.33	Men: 1.43<br>Women: 1.08	-
GBD study 2017	All high income	0	1990, 2016	Men: 5.63<br>Women: 4.14	Men: 4.43<br>Women:3.21	-
		65		Men: 3.51<br>Women: 3.03	Men:2.27<br>Women: 2.6	-
GBD study 2018	All high income	0	1990, 2017	Men: 5.6<br>Women: 4.2	Men: 4.2<br>Women: 3.0	-
Studies with samples from Europe						
Jagger 2016	England	65	1991, 2011	Men: 4·5<br>Women: 3·6	Men: 3·8 (3·5 to 4·1)<br>Women: 3·1 (2·7 to 3·4)	Men: 2.6 (2.3, 2.9)<br>Women: 0.5 (0.2, 0.9)
Kingston 2017	England	65	1991, 2011	Men: 4.7<br>Women: 4.1		Men: 1.7 (1.2, 2.1)<br>Women: 0.2 (-0.4, 0.7)
Reus-Pons 2017	England & Wales	50	2001, 2011	Men: 2.8<br>Women: 2.2	Men: 0.25<br>Women: -0.15	-
ONS 2018	UK	0	2009/11 – 2015/17	Men: 0.8<br>Women: 0.4	Men: 0.4<br>Women: -0.2	-
Bronnum-Hansen 2017	Denmark	65	2006/07, 2010/11,2013/14	Difference in change in LE between high and low education:<br>Men: 0.1<br>Women: 0.3	-	Difference in change in DFLE between high and low education<br>Men: -0.3<br>Women: -0.3
Deeg 2018	Netherlands	65	1993, 1996, 1999, 2002, 2006, 2009, 2012, 2016	Men: 4.0<br>Women: 2.2	Physical<br>Men: -2.2<br>Women: -1.5	-
Gheorghe 2016	Netherlands	25	2001, 2011	Men:<br>High: 2.88<br>Med: 3.22<br>Low: 2.43<br><br>Women:<br>High: 1.78<br>Med: 1.53<br>Low: 1.15	Men:<br>High 2.85<br>Med: 3.01<br>Low: 2.13<br><br>Women:<br>High: 2.64<br>Med: 1.57<br>Low: 1.80	-
		65		Men:<br>High: 2.48	Men:<br>High: 2.17	-

For peer review only - http://bmjopen.bmj.com/site/about/guidelines.xhtml

				Med: 2.24<br>Low: 1.68<br><br>Women:<br>High: 1.64<br>Med: 1.41<br>Low: 1.12	Med: 1.91<br>Low: 1.37<br><br>Women:<br>High: 1.74<br>Med: 1.21<br>Low: 1.14	
Reus-Pons 2017	Netherlands	50	2001, 2011	Men: 2.82<br>Women: 1.84	Men: 2.21<br>Women: 1.25	-
Remund 2019	Switzerland	30	1990/94, 1995/99, 2004/04, 2010/14	Men: 5.02<br>Women: 3.09	Men: 4.52<br>Women: 3.09	-
Storeng 2018	Norway	50	1984/86, 1995/97, 2006/08	Men: 6.99 (5.27–8.72)<br>Women: 6.75 (5.16–8.34)	Men: 6.90 (6.08–7.73)<br>Women: 5.40 (4.56–6.25)	Men: 2.71 (2.01–3.42)<br>Women: 0.33 (–0.40–1.06)
Sundberg 2016	Sweden (SWEOLD)	77	1992, 2002, 2004, 2011	Men: 1.7<br>Women: 1.1	-	Men: 1.1<br>Women: 1.6
	Sweden (SHARE)		2004, 2011	Men: 0.6<br>Women: 0.4	-	Men: 0.1<br>Women: 1.3
Yokota 2019	Belgium	15	2001, 2004, 2008	Men: 1.6<br>Women: 1.0	-	Men: 0.7<br>Women: -0.7
Studies with samples from Asia						
Jo 2019	R. Korea	0	2005, 2007, 2008, 2009, 2010, 2011, 2012, 2013	Men: 3.38<br>Women: 3.15	Men: 4.03<br>Women: 3.44	-
Lee 2016	R. Korea	0	2005, 2008, 2011	Men: 2.5<br>Women: 2.6	Men: 1.4[b]<br>Women: 1.2[b]	-
		65		Men: 1.6<br>Women: 2.0	Men: 2.2[b]<br>Women: 2.6[b]	-
		85		Men: 0.3<br>Women: 0.7	Men: 1.4[b]<br>Women: 1.4[b]	-
Sugawara 2016	Japan	0	2000, 2010	Men: 1.9<br>Women:1.7	-	Men: 1.0<br>Women: 0.4
Tokudome 2016	Japan	0	1990, 1995, 2000, 2005, 2010, 2013	Men: 4.01<br>Women: 4.43	Men: 3.02<br>Women: 3.32	-
Studies with sample from North America						
Crimmins 2016	US	0	1970, 1980, 1990, 2000, 2010	Men: 9.2<br>Women: 6.4	-	Men: 4.5<br>Women: 2.7
		20-64		Men: 1.8<br>Women: 0.9	-	Men: 0.9<br>Women: -0.6
		65		Men: 4.7<br>Women: 3.5	-	Men: 2.7<br>Women: 2.4
		85		Men: 1.1<br>Women: 1.3	-	Men: 0.5<br>Women: 0.8

[a]Table and comparison does not include studies where: the metric of change was not comparable for each health expectancy (Steensma et al. 2017); trends were reported as slope of index inequality only (ONS 2019); only forecasts are reported (Kingston et al., 2018, Guzman-Castillo et al., 2017, Cao et al., 2016); total life expectancy is reported only as a graph (Lagergren et al., 2017, Freedman 2016a,b); trends are reported as difference in change in DFLE between levels of education and not comparable to the LE trend (Renard 2015).

For peer review only

Figure legends:

Figure 1. PRISMA Flowchart

Supplementary Materials Figure 1. Expansion and compression of poor health/disability by country and time period

For peer review only

For peer review only - http://bmjopen.bmj.com/site/about/guidelines.xhtml

Figure 1. PRISMA diagram

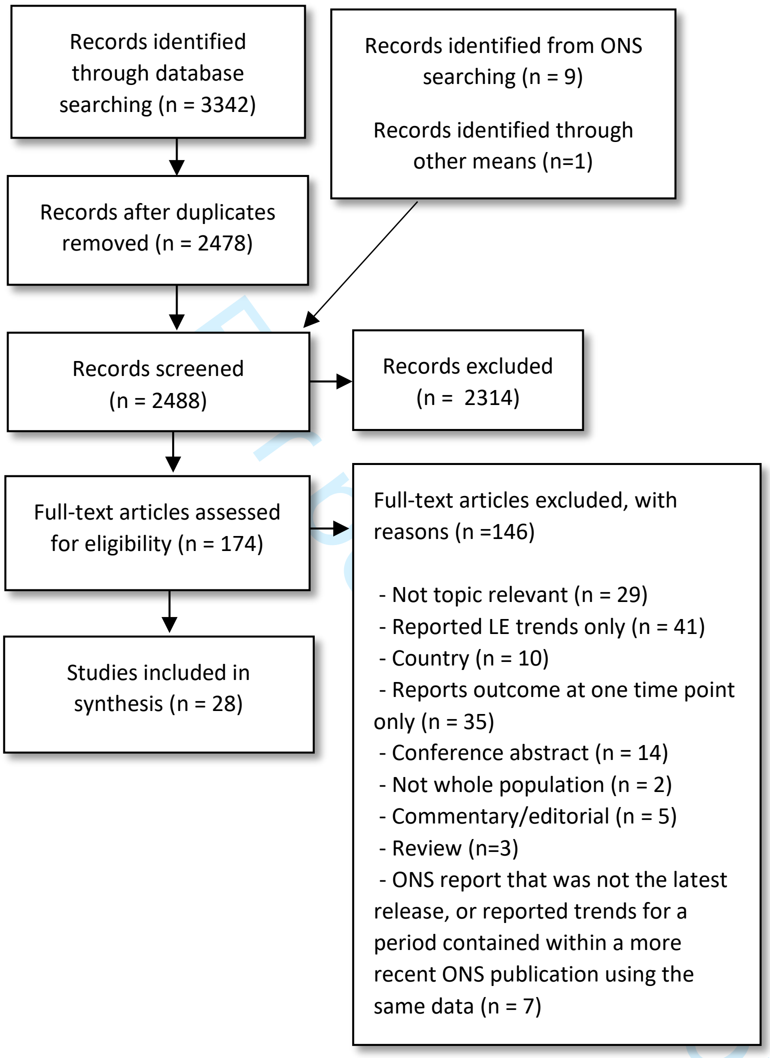

For peer review only

Supplementary Materials

Table 1. Summary of included studies

Study	Country	Data source	Trend years	Trends reported	Disability/health measured from a global (single) item or multiple items?[a]	Stratification	At age	Method used to calculate life expectancy
High income countries combined								
Global Burden of Disease study 2016 [b]	High income countries combined	Global Burden of Disease Study	2005, 2015	LE, HLE	Multiple	Sex	0	Sullivan
Global Burden of Disease study 2017 [b]	High income countries combined	Global Burden of Disease Study	1990, 2016	LE, HLE	Multiple	Sex	0, 65	Sullivan
Global Burden of Disease study 2018 [b]	High income countries combined	Global Burden of Disease Study	1990, 2017	LE, HLE	Multiple	Sex	0	Sullivan
Studies with samples from OECD high-income countries in Europe								
Jagger 2016	England	CFAS I and II	1991, 2011	LE, HLE, DFLE, t % of LE without disability, % of life spent healthy, disability prevalence	HLE: global DFLE: multiple	Sex	65	Sullivan
Kingston 2017	England	CFAS I and II	1991, 2011, and 2015, 2025, 2035 (projection)	LE, DFLE, % of LE without disability, disability prevalence	Multiple	Sex	65	Sullivan

For peer review only - http://bmjopen.bmj.com/site/about/guidelines.xhtml

Study	Country	Data source	Trend years	Trends reported	Disability/health measured from a global (single) item or multiple items?[a]	Stratification	At age	Method used to calculate life expectancy
Kingston 2018	England	CFAS II, ELSA, Understanding Society	2015, 2025, 2035 (projection)	LE, DFLE, % of LE without disability, disability prevalence	Multiple	Sex, age	65-74, 75-84, 85	Sullivan
Guzman-Castillo 2017	England, Wales	ELSA	2015, 2025 (projection)	LE, DFLE, disability prevalence	Multiple	None	65	Sullivan
Reus-Pons 2017	England & Wales	Census, national death registries, Permanent Survey on the Living Situation (POLS), Health Survey	2001, 2011	LE, HLE	Global	Sex, migrant/non-migrant	50	Sullivan
ONS 2019	England & Wales	UK Census, Annual Population Survey, Indices of Multiple Deprivation 2015, Welsh Index of Multiple Deprivation 2014	2012/14 – 2015/17	LE, HLE	Global	Sex, deprivation decile[c]	0, 65	Sullivan
ONS 2018	UK, England, Wales, Northern Ireland, Scotland	UK Census, Annual Population Survey	2009/11 – 2015/17	LE, HLE	Global	Sex	0	Sullivan
Bronnum-Hansen 2017a	Denmark	Danish Surveys of Health, Ageing and Retirement in Europe	2006/07, 2010/11,2013/14	LE, DFLE,	Global	Sex, education	65	Sullivan
Deeg 2018	Netherlands	Longitudinal Aging Study Amsterdam	1993, 1996, 1999, 2002, 2006, 2009, 2012, 2016	LE, HLE, % of life spent healthy	Multiple	Sex	65	Sullivan
Gheorghe 2016	Netherlands	Dutch Labour force Survey and National Mortality Registry	2001, 2011	LE, Quality Adjusted Life Expectancy),	Multiple	Sex, education	25, 65	Sullivan

For peer review only - http://bmjopen.bmj.com/site/about/guidelines.xhtml

Study	Country	Data source	Trend years	Trends reported	Disability/health measured from a global (single) item or multiple items?[a]	Stratification	At age	Method used to calculate life expectancy
Lagergren 2017	Sweden	National mortality statistics, Nationwide Swedish Surveys of Living Conditions	1980/1985, 1994/95, 2006/2011	LE (graph only), DFLE, disability prevalence	Multiple	Sex	65	Sullivan
Remund 2019	Switzerland	Swiss National Cohort, Swiss Health Interview Survey	1990/94, 1995/99, 2004/04, 2010/14	LE, HLE,	Global	Sex, education	30	Sullivan
Renard 2019	Belgium	Census, National Registry	2001, 2011	LE, DFLE,	Global	Sex, education	25	Sullivan
Reus-Pons 2017	Netherlands	Census, national death registries, Permanent Survey on the Living Situation (POLS) and Health Survey	2001, 2011	LE, HLE,	Global	Sex	50	Sullivan
Storeng 2018	Norway	HUNT Study, Norwegian Education Database	1984/86, 1995/97, 2006/08	LE, HLE, DFLE,	HLE & DFLE: Global	Sex, education	30	Sullivan
Sundberg 2016	Sweden	National mortality statistics, Swedish Panel Study of Living Conditions of the Oldest Old (SWEOLD), Survey of Health, Ageing and Retirement in Europe (SHARE)	1992, 2002, 2004, 2011	LE, DFLE,	Multiple	Sex	77	Sullivan
Yokota 2019	Belgium	Health Interview Survey, National Statistics	2001, 2004, 2008	LE, DFLE,	Multiple	Sex	15	Sullivan
Studies with samples from OECD high-income countries in Asia								
Jo 2019	R. Korea	Korean Statistical Information Service, Korea National Health and Nutrition Examination Survey	2005, 2007, 2008, 2009, 2010, 2011, 2012, 2013	LE, HLE,	Multiple	Sex	0	Sullivan
Lee 2016	R. Korea	Korean Statistical Information Service,	2005, 2008, 2011	LE, HLE,	Multiple	Sex	0-100 at five year	Sullivan

For peer review only

Study	Country	Data source	Trend years	Trends reported	Disability/health measured from a global (single) item or multiple items?[a]	Stratification	At age	Method used to calculate life expectancy
		Korea National Health and Nutrition Examination Survey					intervals; ages 0, 65 and 85 reported here	
Sugawara 2016	Japan	Census, Comprehensive Survey of Living Conditions of the People on Health and Welfare	2000, 2010	LE, DFLE, % life without disability	Multiple	None	0	Sullivan
Tokudome 2016	Japan	Global Burden of Disease Study 2013	1990, 1995, 2000, 2005, 2010, 2013	LE, HLE,	Multiple	Sex	0	Sullivan (based on GBD estimates)
Studies with samples from OECD high-income countries in North America								
Cao 2016	US	Human Mortality Database, US National Health Interview Survey (NHIS)	1982, 2010, 2040 (projection)	LE, DFLE,	Global	Sex	55-85	Multi-state life table
Crimmins 2016	US	Census, US National Vital Statistics, National Health Interview	1970, 1980, 1990, 2000, 2010	LE, DFLE, % life without disability, disability prevalence	Global	Sex	0, 20-64, 65, 85	Sullivan
Freedman 2016a	US	National Long-Term Care Survey, National Health and Aging Trends Study	1982, 2004, 2011	LE (graph only), DFLE, prevalence	Multiple	Sex	65, 85	Sullivan
Freedman 2016b	US	National Long-Term Care Survey, National Health and Aging Trends Study	1982, 2004, 2011	LE (graph only), Active Life Expectancy	Multiple	Sex, Race	65, 85	Sullivan
Steensma 2017	Canada	Statistics Canada, National Population Health Survey, Canadian Community Health Survey	1994, , 1994/95, 1996/97, 1998/99, 2000/01 2003, 2005, 2009/10	LE, HLE	Multiple	Sex	0, 20, 65	Sullivan

For peer review only - http://bmjopen.bmj.com/site/about/guidelines.xhtml

[a]Global item measures include self-rated health, the Global Limitation Activity Indicator, or other single item measures about having a limitation; [b]Disability adjusted life years reported as global estimates (i.e. not country specific) and were thus not included here; [c]Healthy life expectancy trends are not reported by deprivation deciles, but as a slope index of inequality between the most and least deprived deciles

For peer review only

Table 2. Quality assessment

Study	Comparability of interview methods	Outcome measure	Does the trend consider more than 2 time points?	% Response rate in cross-sectional surveys or loss to follow up in longitudinal studies	Proxy %	Missing data %	Other publications consulted	SUMMARY JUDGMENT
Bronnum-Hansen 2017	FAIR<br>Change in items across waves, but disability question did not change across waves 1-7.	Disability: FAIR (Single item question - Global Activity Limitation Indicator)	GOOD: Yes	FAIR<br><br>Response rates:<br><br>2006/07: 69.0%<br>2011/12: 61.5%<br>2013/14: 61.0%),	UNCLEAR	UNCLEAR	SHARE methods report: http://www.share-project.org/fileadmin/pdf_documentation/Methodology/Methodology_2005.pdf	FAIR
Cao 2016	FAIR<br>Not reported, but in Freedman et al. (2002), the NHIS (which is the survey used in Cao et al.), they note the sampling frame was redesigned in 1995. A change in disability items is reported, but this study used a different set of items to ensure disability is comparable across waves.	Disability: FAIR<br>Although this measure draws upon multiple ADLs, the question is whether the participant has any limitation in any of the ADLs, and so is a single item measure.	GOOD: Yes (third time point is projection)	GOOD<br>Annual response rate approximately 80%	UNCLEAR	UNCLEAR	Freedman et al. (2002)<br><br>https://www.cms.gov/About-CMS/Agency-Information/OMH/resource-center/hcps-and-researchers/data-tools/sgm-clearinghouse/nhis	FAIR
Crimmins 2016	FAIR<br>Not reported, but in Freedman et al. (2002), the NHIS (which is the survey used in Crimmins et al), they note the sampling frame was redesigned in 1995. A change in disability items is	Disability: FAIR<br>Although question draws upon multiple ADLs, the question is whether the participant has any limitation in any of the ADLs,	GOOD: Yes	GOOD<br>Annual response rate approximately 80%	UNCLEAR	UNCLEAR	Freedman et al. (2002)	FAIR

For peer review only - http://bmjopen.bmj.com/site/about/guidelines.xhtml

Study	Comparability of interview methods	Outcome measure	Does the trend consider more than 2 time points?	% Response rate in cross-sectional surveys or loss to follow up in longitudinal studies	Proxy %	Missing data %	Other publications consulted	SUMMARY JUDGMENT
	reported, but study uses different set of items to ensure disability is comparable across waves.	and so is a single item measure.						
Deeg 2018	GOOD	Disability: GOOD	GOOD: Yes	FAIR<br><br>Response rates:<br><br>1992/93: 81.7%<br>1995/96: 66.9%<br>1998/99: 54.6%<br>2001/02: 44.4% (cohort 1 only, replenished in 2002/2003)<br>2005/06: 33.0% (cohort 1) and : 90.6% (cohort 2)<br>2008/09: 25.9% (cohort 1) and 83.1% (cohort 2)<br>2011/12: 20.1% (cohort 1) and 75.7% (cohort 2)<br>2015/16: 13.1% (cohort 1) and 67.0% (cohort 2) and 83.4% (cohort 3)	GOOD<br>From publication appendix: "Across the study period, the average use of proxies was 2% for respondents in good physical health, 3% for those in fair physical health, and 8% for those in poor physical health."	GOOD<br>Across waves:<br><br>Men: 3.3%<br>Women: 4.4%	https://www.ncbi.nlm.nih.gov/pmc/articles/PMC5010587/<br><br>https://link.springer.com/article/10.1007/s10654-019-00541-2	FAIR
Freedman 2016a	GOOD	Disability: GOOD	GOOD: Yes	GOOD<br><br>Response rates:<br><br>1982: 87.3%<br>2004: 80.6%<br>2011: (NHATS round 1) 70.9%	POOR<br>1982: 20.2%<br>2011: 7.2%	GOOD<br>1982: 0%<br>2011: 2.1%	https://aspe.hhs.gov/basic-report/introduction-national-long-term-care-survey<br><br>https://www.nhats.org/sites/default/files/2021-01/NHATS_User_Guide_R9_Final_Release_0.pdf<br><br>https://www.ncbi.nlm.nih.gov/pmc/articles/PMC6934030/	GOOD

For peer review only - http://bmjopen.bmj.com/site/about/guidelines.xhtml

Study	Comparability of interview methods	Outcome measure	Does the trend consider more than 2 time points?	% Response rate in cross-sectional surveys or loss to follow up in longitudinal studies	Proxy %	Missing data %	Other publications consulted	SUMMARY JUDGMENT
Freedman 2016b	GOOD	Disability: GOOD	GOOD: Yes	GOOD<br><br>Response rates:<br><br>1982: 87.3%<br>2004: 80.6%<br>2011: (NHATS round 1) 70.9%	POOR<br>1982: 20.2%<br>2011: 7.2%	GOOD<br>1982: 0%<br>2011: 2.1%	https://aspe.hhs.gov/basic-report/introduction-national-long-term-care-survey<br><br>https://www.nhats.org/sites/default/files/2021-01/NHATS_User_Guide_R9_Final_Release_0.pdf<br><br>https://www.ncbi.nlm.nih.gov/pmc/articles/PMC6934030/	GOOD
GBD 2016 DALYs and HALE Collaborators	UNCLEAR<br>Not reported.	Disability: GOOD	FAIR: No	UNCLEAR	UNCLEAR	UNCLEAR		UNCLEAR
GBD 2017 DALYs and HALE Collaborators	UNCLEAR<br>Not reported.	Disability: GOOD	FAIR: No	UNCLEAR	UNCLEAR	UNCLEAR		UNCLEAR
GBD 2018 DALYs and HALE Collaborators	UNCLEAR<br>Not reported.	Disability: GOOD	FAIR: No	UNCLEAR	UNCLEAR	UNCLEAR		UNCLEAR
Gheorghe 2016	FAIR<br>Mode of collection differed but health questions remained the same.	Health: GOOD	FAIR: No	UNCLEAR	UNCLEAR	UNCLEAR		UNCLEAR
Guzman-Castillo 2017	NA as forecasting from baseline data	Disability: GOOD	FAIR: No	NA (forecasts)	GOOD<br>Full interview by proxy 4%	UNCLEAR	Rogers et al. (2016) DOI 10.1007/978-981-287-080-3_52-1	FAIR

For peer review only - http://bmjopen.bmj.com/site/about/guidelines.xhtml

Study	Comparability of interview methods	Outcome measure	Does the trend consider more than 2 time points?	% Response rate in cross-sectional surveys or loss to follow up in longitudinal studies	Proxy %	Missing data %	Other publications consulted	SUMMARY JUDGMENT
					Partial interview or institute proxy <1%		https://www.ifs.org.uk/uploads/elsa/docs_w7/ELSA%20Wave%207%20report.pdf	
Jagger 2016	GOOD	SRH: FAIR<br>Disability: GOOD	FAIR: No	FAIR<br><br>Response rates:<br><br>CFAS I: 80%<br>CFAS II: 56%	GOOD<br><br>CFAS I: 0.1%<br>CFAS II: 0.1%	GOOD<br>SRH: 2·9% (1991) and 4·2% (2011)<br>Disability: 1·1% (1991) and 4·2% (2011)	https://www.sciencedirect.com/science/article/pii/S0140673613615706<br><br>https://ars.els-cdn.com/content/image/1-s2.0-S0140673613615706-mmc1.pdf	FAIR/GOOD depending on outcome
Jo 2019	GOOD<br>Although there were changes to the survey, only waves 3-5 were used where the health question used was the same.	QALE: GOOD	GOOD: Yes	UNCLEAR<br>Not reported	GOOD<br>3-4%	GOOD<br>3-4%	https://www.ncbi.nlm.nih.gov/pmc/articles/PMC3937975/	UNCLEAR
Kingston 2017	GOOD	Disability: GOOD	GOOD: Yes (fourth and fifth time points are projections)	FAIR<br><br>Response rates:<br><br>CFAS I: 80%<br>CFAS II: 56%	GOOD<br><br>CFAS I: https://ars.els-cdn.com/content/image/1-s2.0-S0140673613615706-mmc1.pdf<br>CFAS II: 0.1%	GOOD<br>Not reported but assumed to be same as Jagger et al. as uses same data.	https://www.sciencedirect.com/science/article/pii/S0140673613615706<br><br>https://ars.els-cdn.com/content/image/1-s2.0-S0140673613615706-mmc1.pdf	FAIR
Kingston 2018	NA as forecasting from baseline data	Disability: GOOD	GOOD: Yes (third time point is projection)	FAIR<br><br>Response rates:<br><br>CFAS II: 56%<br>ELSA Wave 5: 80%<br>Understanding society wave 1: 57% (General population)	UNCLEAR/GOOD depending on dataset<br><br>CFAS II: 0.1% (GOOD)<br><br>No information on % proxy	UNCLEAR<br>Not reported, but missing values imputed.	https://www.understandingsociety.ac.uk/sites/default/files/downloads/documentation/mainstage/technical-reports/Wave_1_Technical_Report.pdf	FAIR

For peer review only - http://bmjopen.bmj.com/site/about/guidelines.xhtml

Study	Comparability of interview methods	Outcome measure	Does the trend consider more than 2 time points?	% Response rate in cross-sectional surveys or loss to follow up in longitudinal studies	Proxy %	Missing data %	Other publications consulted	SUMMARY JUDGMENT
				and 40% (Ethnic Minority Boost)	interviews for ELSA (UNCLEAR).			
Lagergren 2017	POOR<br>"In the years 1996–2000, a filter was introduced into the questionnaire to the effect that only people who were dependent for all instrumental ADL (IADL) were asked the questions about ADL." (p.552)<br><br>"From 1980 to 2001 there was an upper age limit of 84 years, with the exception of 1988/89." (p.56)	Disability: GOOD	GOOD: Yes	UNCLEAR<br>Response rate reported to be 70-75% with slight decrease up to 2006, but 'lower' after 2006 (actual rates not reported).	FAIR<br>From methods paper on dataset: "The proportion of proxy interviews varies from 2–3% among those aged 65–79 years to 14–16% among individuals aged 80 years and older." (p.56)	UNCLEAR	https://journals.sagepub.com/doi/full/10.1177/1403494815605195	POOR
Lee 2016	GOOD<br>Same criteria used across the waves used to identify diseases.	GOOD<br>Health adjusted life expectancy was calculated using disability weights attached to self-reported morbidity for 45 diseases. This was converted into a score of disability measured on a scale from 0 (full health) to 1 (being dead). This would suggest it	GOOD: Yes	UNCLEAR<br>Not reported, but 2011 KNHANES wave known to be 76.1% for health interview and examination (Jo et al., 2019)	GOOD<br>3-4%<br>Not reported, but assumed to be the same as for Lee et al., 2016, which uses the same data.	GOOD<br>3-4%<br>Not reported, but assumed to be the same as for Lee et al., 2016, which uses the same data.		UNCLEAR

For peer review only - http://bmjopen.bmj.com/site/about/guidelines.xhtml

Study	Comparability of interview methods	Outcome measure	Does the trend consider more than 2 time points?	% Response rate in cross-sectional surveys or loss to follow up in longitudinal studies	Proxy %	Missing data %	Other publications consulted	SUMMARY JUDGMENT
		is a multiple item measure.						
ONS 2018	GOOD<br>No change in health question/measure, but items used to measure disability changed from the 2013 APS survey (p.4). However, the ONS analysis does not report trends in DFLE (HLE and LE only).	Health: FAIR	FAIR: No	UNCLEAR	UNCLEAR	UNCLEAR<br>Methods report states that missing data are deleted from the survey, but there is no detail on what proportion of data is missing.	https://www.ons.gov.uk/peoplepopulationandcommunity/healthandsocialcare/healthandlifeexpectancies/methodologies/healthstatelifeexpectanciesukqmi#_blank	UNCLEAR
ONS 2019	GOOD<br>No change in health question/measure, but items used to measure disability changed from the 2013 APS survey (p.4). However, the ONS analysis does not report trends in DFLE (HLE and LE only).	Health: FAIR	FAIR: No	UNCLEAR	UNCLEAR	UNCLEAR<br>Methods report states that missing data are deleted from the survey, but there is no detail on what proportion of data is missing.	https://www.ons.gov.uk/peoplepopulationandcommunity/healthandsocialcare/healthandlifeexpectancies/methodologies/healthstatelifeexpectanciesukqmi#_blank	UNCLEAR
Remund 2019	POOR<br>The phrasing of the SRH question and response items were not identical in each survey wave. However, additional analysis supported the use of	SRH: FAIR	GOOD: Yes	GOOD<br><br>Response rates:<br><br>1992: 70.8%<br>1997: 68.8%<br>2002: 63.9%<br>2007: 66.3%	GOOD<br>(% cases, % weights)<br>1992: 0.67%, 0.86%<br>1997: 0.74%, 0.79%	GOOD<br>(% cases, % weights)<br>1992: 0.0%, 0.0%<br>1997: 0.03%, 0.04%	Supplementary materials	POOR

For peer review only - http://bmjopen.bmj.com/site/about/guidelines.xhtml

Study	Comparability of interview methods	Outcome measure	Does the trend consider more than 2 time points?	% Response rate in cross-sectional surveys or loss to follow up in longitudinal studies	Proxy %	Missing data %	Other publications consulted	SUMMARY JUDGMENT
	dichotomisation to maximise the comparability of the questions between waves.			2012: 53.1	2002: 1.05%, 0.86%<br>2007: 4.41%, 6.23%<br>2012: 3.32%, 4.26%	2002: 0.03%, 0.02%<br>2007: 0.05%, 0.05%<br>2012: 0.12%, 0.12%		
Renard 2019	GOOD<br>Methods paper states no major methodological changes have been implemented between waves, except oversampling from 1997.	Disability: FAIR (Single item question - Global Activity Limitation Indicator)	FAIR: No	FAIR<br><br>Response rates:<br><br>2001: 49%<br>2004: 49.9%<br>2008: 40.2%<br>2013: Not reported.	UNCLEAR	UNCLEAR	https://archpublichealth.biomedcentral.com/articles/10.1186/0778-7367-71-24	FAIR
Reus-Pons 2017	GOOD<br>For England & Wales data, paper indicates SRH question changed but an adjustment was made for comparability.<br>For Netherlands data, footnote to table states no major changes were made to SRH question and choices.	SRH: FAIR	FAIR: No	UNCLEAR<br><br>Response rates:<br><br>POLS: UNCLEAR<br>Census 2011: 94% (person response rate)<br>Census 2001: 98%	UNCLEAR	UNCLEAR<br>For Belgium sample: data on self-rated health were missing for 'around' 5% of the non-migrant population and 'around' 10% of the migrant population. Exact % not reported, thus could be >10%.<br>Not reported for England	https://www.mzes.uni-mannheim.de/projekte/mikrodaten/wp_pdf/wp_30_NL-ro.pdf<br><br>https://www.ons.gov.uk/census/2001censusandearlier/dataandproducts/qualityofthecensusdata/responserates<br><br>https://www.ons.gov.uk/peoplepopulationandcommunity/populationandmigration/populationestimates/bulletins/2011censuspopulationandhouseholdestimatesforenglandandwales/2012-07-16	UNCLEAR

For peer review only - http://bmjopen.bmj.com/site/about/guidelines.xhtml

Study	Comparability of interview methods	Outcome measure	Does the trend consider more than 2 time points?	% Response rate in cross-sectional surveys or loss to follow up in longitudinal studies	Proxy %	Missing data %	Other publications consulted	SUMMARY JUDGMENT
						and Wales sample.		
Steensma 2017	GOOD<br>Different surveys used between 1994-1999 and 2000-2010, but same question for health used in both.	Health: GOOD (multiple item health utilities index)	GOOD: Yes	GOOD<br><br>Response rates:<br><br>NPHS:<br>1994/95: 88.7%,<br>1996/97: 82.6%<br>1998/99: 89.7%<br><br>CCHS:<br>2000/01: 84.7%<br>2009/10: 72.3%	POOR (NPHS), GOOD (CCHS)<br><br>NPHS: 27.5% (for health data)<br><br>CCHS: 6.3%	NPHS: GOOD 1994/1995: between 1% and 9% depending on variable.<br>CCHS: UNCLEAR	https://www23.statcan.gc.ca/imdb/p2SV.pl?Function=getSurvey&Id=3359<br>https://pubmed.ncbi.nlm.nih.gov/10607414/<br>https://pubmed.ncbi.nlm.nih.gov/11565112/	GOOD
Storeng 2018	POOR<br>Self-rated health was measured the same at all three HUNT studies. The question for longstanding limiting illness differed between HUNT1 and HUNT 2 and 3 (that is, HUNT 2 and 3 asked about limiting illness lasting for at least one year, whereas HUNT 1 did not).	Health: FAIR<br>Disability: FAIR	GOOD: Yes	FAIR<br><br>Response rates:<br><br>HUNT 1: 90%<br>HUNT 3: 54%	UNCLEAR	GOOD<br>Missing health data < 5%	https://academic.oup.com/ije/article/42/4/968/655743#11331793	POOR
Sugawara 2016	GOOD	FAIR<br>Disability was measured using the following two survey questions: “Are you currently institutionalized in hospitals,	FAIR: No	GOOD<br><br>Response rates:<br><br>2001: 87.4%<br>2010: 79.4%	UNCLEAR	UNCLEAR		FAIR

For peer review only - http://bmjopen.bmj.com/site/about/guidelines.xhtml

Study	Comparability of interview methods	Outcome measure	Does the trend consider more than 2 time points?	% Response rate in cross-sectional surveys or loss to follow up in longitudinal studies	Proxy %	Missing data %	Other publications consulted	SUMMARY JUDGMENT
		clinics, or long-term care facilities?" and "Do you have any limitations in carrying out normal activities due to health problems?" Those who answered "yes" to **<u>either</u>** question are considered to have disability.						
Sundberg 2016	FAIR<br>Mode of data collection changed but health question remain the same.	Disability: GOOD	GOOD: Yes	GOOD/FAIR depending on dataset<br><br>Response rates:<br><br>SWEOLD<br>1992: 95.4%<br>2002: 84.4%<br>2004: 46.9%<br>2011: 86.2%<br><br>SHARE (retention plus recovery):<br>2004: 70.6%<br>2011:108.4%	FAIR/POOR depending on dataset<br><br>SHARE<br>2004: 14.4%<br>2011: 9 %<br><br>SWEOLD<br>1992: 15%<br>2002: 19.8%<br>2004: 21.5%<br>2011: 20.9%	GOOD/UNCLEAR depending on dataset<br><br>SHARE<br>Not reported.<br><br>SWEOLD (severe disability, mild disability, mobility)<br>1992: 1.7%, 0.4%, 0%<br>2002: 1.0%, 1.1%, 1.3%<br>2004: 2.0%, 0.9%, 0.9%<br>2011: 2.4%, 1.3%, 1.4%	https://link.springer.com/article/10.1007%2Fs10433-013-0275-7 and<br>https://academic.oup.com/ije/article/43/3/731/2949546#57616062<br><br>https://academic.oup.com/ije/article/43/3/731/2949546<br><br>https://academic.oup.com/ije/article/42/4/992/657275<br><br>Borsch-Supan, A & Scherpenzeel , A. The Survey of Health, Ageing and Retirement in Europe. Munich Center for the Economics of Aging,	FAIR

For peer review only - http://bmjopen.bmj.com/site/about/guidelines.xhtml

Study	Comparability of interview methods	Outcome measure	Does the trend consider more than 2 time points?	% Response rate in cross-sectional surveys or loss to follow up in longitudinal studies	Proxy %	Missing data %	Other publications consulted	SUMMARY JUDGMENT
Tokudome 2016	GOOD	Disability: GOOD	GOOD: Yes	UNCLEAR<br>(Based on HALE values from GBD 2013)	UNCLEAR	UNCLEAR<br>But reported to be rare by author.	http://www.healthdata.org/sites/default/files/files/Projects/GBD/GBD_Protocol.pdf	UNCLEAR
Yokota 2019	GOOD<br>Methods paper states no major methodological changes have been implemented between waves, except oversampling from 1997.	Disability: GOOD	GOOD: Yes	FAIR<br><br>2001: 49%<br>2004: 49.9%<br>2008: 40.2%	FAIR<br>Time 1: 4.3%,<br>time 2: 10.2%,<br>time 3: 13%	UNCLEAR	https://archpublichealth.biomedcentral.com/articles/10.1186/0778-7367-71-24	FAIR

For peer review only - http://bmjopen.bmj.com/site/about/guidelines.xhtml

For tables 3a to 7b, the change in expectancy from the first to the last time point represents the final estimated expectancy minus the first estimated expectancy.

Table 3a. Summary of life expectancy trends data for studies from the US and Canada (years)[a]

Study	At age	Time points	T1		T2		T3		T4		T5		T6		T7		Change between first and last time point
			M	F	M	F	M	F	M	F	M	F	M	F	M	F	
Cao 2016	55-85	T1: 1982<br>T2: 1990<br>T3: 2000<br>T4: 2010<br>T5: 2020<br>T6: 2030<br>T7: 2040	19.96	23.40	20.70	23.64	21.75	23.92	22.82	24.69	23.83 (23.5 - 24.05)	25.11 (24.9 - 25.24)	24.63 (24.2 - 24.98)	25.49 (25.2 - 25.69)	25.24 (24.7 - 25.67)	25.74 (25.4 - 26.00)	Men: 5.28[b]<br>Women: 2.34[b]
Crimmins 2016	0	T1: 1970<br>T2: 1980<br>T3: 1990<br>T4: 2000<br>T5: 2010	67.0	74.6	70.1	77.6	71.8	78.8	74.1	79.5	76.2	81.0					Men: 9.2<br>Women: 6.4
	20-64		40.8	42.8	41.6	43.3	41.8	43.5	42.4	43.6	42.6	43.7					Men: 1.8<br>Women: 0.9
	65		13.0	16.8	14.2	18.4	15.1	19.0	16.1	19.1	17.7	20.3					Men: 4.7<br>Women: 3.5
	85		4.7	5.6	5.1	6.4	5.3	6.7	5.5	6.6	5.8	6.9					Men: 1.1<br>Women: 1.3
Steensma 2017	0	T1: 1994/95<br>T2: 96/97<br>T3: 98/99<br>T4: 00/01<br>T5: 03<br>T6: 05<br>T7: 09/10	75.2	81.3	75.7	81.5	76.2	81.8	77.2	82.5	77.7	82.8	78.3	83.2	79.6	84.1	Men: 4.4[b]<br>Women: 2.8[b]
	20		55.7	61.7	56.2	61.8	56.7	62.1	57.7	62.8	58.1	63.0	58.7	63.4	59.9	64.4	Men: 4.2[b]<br>Women: 2.7[b]
	65		15.8	19.9	15.9	19.9	16.2	20.2	17.0	20.7	17.4	20.9	19.9	21.3	18.9	22.1	Men: 3.1[b]<br>Women: 2.2[b]

[a]95% confidence intervals in brackets where reported; [b]Change estimate not reported, and thus calculated by us (T^{2}-T^{1})

For peer review only

For peer review only - http://bmjopen.bmj.com/site/about/guidelines.xhtml

Table 3b. Summary of healthy life expectancy trends data for studies from the US and Canada (years)[a]

Study	At age	Time points	T1		T2		T3		T4		T5		T6		T7		Average annual change (%)
			M	F	M	F	M	F	M	F	M	F	M	F	M	F	
Steensma 2017	0	T1: 1994/95 T2: 96/97 T3: 98/99 T4: 00/01 T5: 03 T6: 05 T7: 09/10	66.4 (66.0 - 66.8)	70.0 (69.5 - 70.4)	69.0 (68.7 - 69.2)	72.9 (72.5 - 73.2)	68.9 (68.6 - 69.3)	72.1 (71.7 - 72.5)	68.5 (68.3 - 68.7)	71.5 (71.3 - 71.7)	69.5 (69.1 - 69.8)	71.7 (71.3 - 72.1)	69.3 (69.0 - 69.7)	72.3 (71.9 - 72.6)	70.7 (70.5 - 70.9)	73.3 (73.1 - 73.5)	Men: 0.3 Women: 0.2
	20		48.0 (47.6 - 48.3)	51.4 (51.0 - 51.9)	50.1 (49.8 - 50.3)	54.0 (53.7 - 54.3)	50.1 (49.7 - 50.4)	53.1 (52.7 - 53.5)	49.8 (49.6 - 50.0)	52.7 (52.5 - 52.9)	50.9 (50.5 - 51.2)	53.1 (52.7 - 53.5)	50.8 (50.4 - 51.2)	55.3 (53.1 - 53.9)	50.2 (51.8 - 52.2)	54.5 (54.3 - 54.8)	Men: 0.4 Women: 0.2
	65		12.3 (12.0 - 12.6)	14.8 (14.4 - 15.1)	12.8 (12.6 - 13.1)	15.6 (15.3 - 15.9)	12.8 (12.5 - 13.1)	15.3 (15.0 - 15.7)	13.1 (13.0 - 13.3)	15.4 (15.2 - 15.6)	14.0 (13.7 - 14.3)	15.7 (15.4 - 16.1)	14.2 (13.9 - 14.5)	16.0 (15.6 - 16.3)	14.9 (14.7 - 15.1)	16.8 (16.7 - 17.0)	Men: 1.2 Women: 0.7

[a]95% confidence intervals in brackets where reported;

For peer review only

Table 3c. Summary of disability-free life expectancy trends data for studies from the US and Canada[a]

Study	Trend	At age	Time points	T1		T2		T3		T4		T5		Change between first and last time point
				Male	Female	Male	Female	Male	Female	Male	Female	Male	Female	
Crimmins (2016)	DFLE (years)	0	T1: 1970<br>T2: 1980<br>T3: 1990<br>T4: 2000<br>T5: 2010	56.5 (56.4 - 56.6)	62.7 (62.6 - 62.8)	56.5 (56.4 - 56.6)	62.8 (62.6 - 63.0)	58.8 (58.6 - 58.9)	63.9 (63.8 - 64.0)	60.0 (59.9 - 60.2)	64.6 (64.4 - 64.7)	61.0 (60.9 - 61.2)	65.4 (65.3 - 65.6)	Men: 4.5<br>Women: 2.7
		20-64		34.9 (34.8 - 35.0)	37.4 (37.3 - 37.5)	34.7 (34.6 - 34.8)	36.7 (36.6 - 36.8)	35.3 (35.2 - 35.4)	37.0 (36.9 - 37.1)	35.6 (35.5 - 35.7)	36.9 (36.8 - 37.0)	35.8 (35.7 - 35.9)	36.8 (36.7 - 37.0)	Men: 0.9<br>Women: -0.6
		65		6.6 (6.6 - 6.8)	9.1 (9.0 - 9.2)	6.8 (6.7 - 6.9)	9.3 (9.2 - 9.4)	7.4 (7.3 - 7.5)	9.9 (9.8 - 10.0)	8.2 (8.0 - 8.3)	10.5 (10.3 - 10.6)	9.3 (9.1 - 9.4)	11.5 (11.4 - 11.6)	Men: 2.7<br>Women: 2.4
		85		1.4 (1.3 - 1.6)	1.4 (1.3 - 1.6)	1.5 (1.3 - 1.7)	1.7 (1.5 - 1.8)	1.6 (1.5 - 1.8)	1.7 (1.6 - 1.8)	1.8 (1.6 - 1.9)	1.9 (1.8 - 2.0)	1.9 (1.8 - 2.1)	2.2 (2.1 - 2.4)	Men: 0.5<br>Women: 0.8
	% of life spent disability-free	0		84.2	84.0	81.6	80.9	81.8	81.1	81.0	81.3	80.1	80.7	Men: -4.1<br>Women: -3.3
		20-64		85.5	87.4	83.4	84.8	84.4	85.0	84.0	84.6	84.0	84.2	Men: -1.5<br>Women: -3.2
		65		51.2	54.2	47.8	50.5	49.1	52	50.9	55	52.5	56.7	Men: 1.3<br>Women: 2.5
		85		30.4	25.2	29	26	30.8	25.5	32.7	28.8	32.8	31.9	Men: 2.4<br>Women: 6.7
	LE for those in an institution	0		0.6 (0.6 - 0.6)	1.1 (1.0 - 1.2)	0.6 (0.6 - 0.7)	1.4 (1.3 - 1.5)	0.7 (0.6 - 0.7)	1.5 (1.4 - 1.5)	0.5 (0.5 - 0.6)	1.1 (1.0 - 1.2)	0.4 (0.4 - 0.5)	0.8 (0.8 - 0.9)	Men: -0.2<br>Women: -0.3
		20-64		0.3 (0.2 - 0.3)	0.2 (0.2 - 0.2)	0.2 (0.2 - 0.2)	0.2 (0.1 - 0.2)	0.2 (0.2 - 0.2)	0.1 (0.1 - 0.1)	0.1 (0.1 - 0.1)	0.1 (0.1 - 0.1)	0.1 (0.1 - 0.1)	0.1 (0.0 - 0.1)	Men: -0.2<br>Women: -0.1

For peer review only

For peer review only - http://bmjopen.bmj.com/site/about/guidelines.xhtml

Study	Trend	At age	Time points	T1		T2		T3		T4		T5		Change between first and last time point
				Male	Female	Male	Female	Male	Female	Male	Female	Male	Female	
		65		0.5 (0.5 - 0.5)	1.1 (1.0 - 1.2)	0.6 (0.5 - 0.6)	1.5 (1.4 - 1.6)	0.7 (0.6 - 0.7)	1.6 (1.5 - 1.6)	0.5 (0.5 - 0.6)	1.2 (1.1 - 1.3)	0.4 (0.4 - 0.5)	0.9 (0.8 - 0.9)	Men: -0.1<br>Women: -0.2
		85		0.6 (0.5 - 0.7)	1.2 (1.1 - 1.3)	0.8 (0.7 - 1.0)	1.7 (1.5 - 1.8)	0.9 (0.8 - 1.0)	1.9 (1.8 - 2.0)	0.7 (0.6 - 0.8)	1.4 (1.3, 1.5 -	0.4 (0.4 - 0.5)	0.9 (0.8 - 1.0)	Men: -0.2<br>Women: -0.3
Cao 2016	DFLE (years)	55-85	T1: 1982<br>T2: 1990<br>T3: 2020<br>T4: 2030<br>T5: 2040	12.98	14.9	16.92	17.53	17.86 (17.70 - 18.03)	18.00 (17.89 - 18.13)	18.99 (18.68 - 19.36)	18.67 (18.41 - 18.97)	20.10 (19.60 - 20.64)	19.30 (18.88 - 19.77)	Men: 7.12[b]<br>Women: 4.4[b]
Freedman (2016)a	DFLE (years)	65	T1: 1982<br>T2: 2004<br>T3: 2011	**data only reported in graphs, unable to extract**										Men: 4.5<br>Women: 1.4
		85		2.5	2.5	4.4	2.6							Men: 1.9<br>Women: 0.1[b]
Freedman (2016)b	Active Life Expectancy (years)	65	T1: 1982<br>T2: 2011	White: 12.2<br>Black: 9.8		White: 15.9<br>Black: 12.0								White: 2.8<br>Black: 2.2
		85		White: 2.5<br>Black: 2.25		White: 3.0<br>Black: 2.0								White: 0.5<br>Black: -0.25

[a]95% confidence intervals in brackets where reported; [b]Change estimate not reported, and thus calculated by us (T^2-T^1)

For peer review only

Table 3d. Summary of disability prevalence trends data for studies from the US and Canada[a]

Study	Trend	Time points	Change between first and last time point
Crimmins (2016)	Proportion (%) of individuals with disability in the community (age 65-84)	T1: 1970<br>T2: 1980<br>T3: 1990<br>T4: 2000<br>T5: 2010	Men: -2.5<br>Women: -2.2
	Proportion (%) of individuals with disability in the community (age 85+)		Men: -0.5<br>Women: -5.8
Freedman (2016)	Proportion of those with a severe limitation (limited in 3+ personal care activities or living in a nursing home)	T1: 1982<br>T2: 2004<br>T3: 2011	Men: -3.4[b]<br>Women: -3.0[b]

[a]Estimates for each time point were not extracted here due to volume. Reader is referred to publications and associated supplementary materials; [b]Change estimate not reported, and thus calculated by us (T^2-T^1)

For peer review only

For peer review only - http://bmjopen.bmj.com/site/about/guidelines.xhtml

Table 4a. Summary of life expectancy trends data for UK studies and studies with UK samples (years)[a]

Study author and date	At age	Time points	T1		T2		T3		Change between first and last time point
			Male	Female	Male	Female	Male	Female	
Reus-Pons 2017	50	T1: 2001<br>T2: 2011	28.54<br><br>Immigrant: 27.52<br>Non-immigrant: 28.66	32.32<br><br>Immigrant: 31.82<br>Non-immigrant: 32.39	31.29<br><br>Immigrant: 31.01<br>Non-immigrant: 31.32	34.5<br><br>Immigrant: 34.84<br>Non-immigrant: 34.47			Men: 2.8[b]<br>Women: 2.2[b]
Jagger 2016	65	T1: 1991<br>T2: 2011	13·0	16·7	17·5	20·3			Men: 4·5<br>Women: 3·6
Kingston 2018	65	T1: 2015<br>T2: 2025<br>T3: 2035	18·7 (18·3 to 19·0)	21·1 (20·8 to 21·1)	20·7 (20·5 to 21·0)	22·7 (22·5 to 23·3)	22·2 (21·7 to 22·4)	24·1 (23·9 to 24·4)	Men: 3·5 (3·1 to 4·1)<br>Women: 3·0 (3·0 to 3·6)
Kingston 2017	65	T1: 1991<br>T2: 2011	12.9	16.5	17.6	20.6			Men: 4.7<br>Women: 4.1
ONS 2018	0	T1: 2009/12<br>T2: 2015/17	**Not reported**						UK<br>Men: 0.8<br>Women: 0.4<br>England<br>Men: 0.8<br>Women: 0.4<br>Northern Ireland<br>Men: 1.0<br>Women:0.5<br>Scotland<br>Men: 0.8<br>Women: 0.5

For peer review only

Study author and date	At age	Time points	T1		T2		T3		Change between first and last time point
			Male	Female	Male	Female	Male	Female	
									Wales<br>Men: 0.5<br>Women: 0.2

[a]95% confidence intervals in brackets where reported; [b]Change estimate not reported in publication, and calculated by us (T^2-T^1);

For peer review only

Table 4b. Summary of healthy life expectancy trends data for UK studies and studies with UK samples[a]

Study	Trend	At age	Time points	T1		T2		Change between first and last time point
				Male	Female	Male	Female	
Reus-Pons 2017	HLE (years)	50	T1: 2001 T2: 2011	18.47 (18.46-18.48)	19.82 (19.81-19.83)	18.71 (18.70-18.72)	19.67 (19.66-, 19.68)	Men: 0.25 Women: -0.15
Jagger 2016	HLE (years)	65	T1: 1991 T2: 2011	8·8 (8·6-9·1)	11·2 (11·0 - 11·5)	12·6 (12·4-12·9)	14·3 (14·0 - 14·6)	Men: 3·8 (3·5 to 4·1) Women: 3·1 (2·7 to 3·4)
	Proportion (%) of life spent healthy			68·2% (66·5 - 69·9)	67·3% (65·9 - 68·7)	72·4% (70·9 - 73·9)	70·3% (68·8 - 71·7)	Men: 4·2% (2·0 to 6·5) Women: 3·0% (1·0 to 4·9)
ONS 2018	HLE (years) - UK	0	T1: 2009/11 T2: 2015/17	62.7	63.8	63.1	63.6	Men: 0.4 [b] Women: -0.2 [b]

[a]95% confidence intervals in brackets where reported; [b]Change estimate not reported in publication, and calculated by us (T^2-T^1);

For peer review only - http://bmjopen.bmj.com/site/about/guidelines.xhtml

Table 4c. Summary of disability-free life expectancy trends data for UK studies and studies with UK samples[a]

Study	Trend	At age	Time points	T1		T2		T3		Change between first and last time point
				Male	Female	Male	Female	Male	Female	
Jagger 2016	DFLE (years)	65	T1: 1991 T2: 2011	10.3 (10.2-10.5)	11.0 (10.8-11.2)	12.9 (12.7-13.2)	11.5 (11.3-11.8)			Men: 2.6 (2.3, 2.9) Women: 0.5 (0.2, 0.9)
	% of life disability-free			79.7 (78.3-81.0)	66.1 (64.9-67.4)	74.4 (73.0-75.8)	56.8 (55.5-58.2)			Men: -5.3 (-7.2, -3.4) Women: -9.3 (-11.1, -7.5)
Kingston 2017	DFLE (years)	65	T1: 1991 T2: 2011	9.5 (9.3-9.7)	9.5 (9.2-9.8)	11.2 (10.8-11.5)	9.7 (9.3-10.2)			Men: 1.7 (1.2, 2.1) Women: 0.2 (-0.4, 0.7)
	% of life disability-free			73.6 (71.8-75.4)	58.0 (56.2-59.9)	63.5 (61.4-65.6)	47.3 (45.0-49.5)			Men: -10.1 (-12.9, -7.3) Women: -10.7 (-13.6, -7.8)
Kingston 2018	DFLE (years)	65	T1: 2015 T2: 2025 T3: 2035	11.1 (10.9-11.3)	10.7 (10.5-10.7)	14.5 (14.4-14.6)	11.4 (11.3-11.5)	15.2 (15.1-15.2)	11.6 (11.6-11.8)	Men: 4.2 (3.9, 4.2) Women: 0.9 (0.9, 1.2)
	% of life disability-free			59.3 (59.3-60.2)	50.6 (50.1-50.7)	70.2 (69.7-70.4)	49.9 (49.4-50.3)	68.7 (67.9-69.5)	48.0 (48.0-48.6)	Men: 9.4 (8.0, 9.7) Women: -2.6%
Guzman-Castillo 2017	DFLE (years)	65	T1: 2015 T2: 2025	14.9 (14.7-15.1)	15.8 (15.7-15.9)	16.5 (15.4-17.6)	16.4 (15.1-17.7)			Men: 1.6 (0.5, 2.7) Women: 0.6 (-0.7, 1.9)

[a]95% confidence intervals in brackets where reported;

For peer review only - http://bmjopen.bmj.com/site/about/guidelines.xhtml

Table 4d. Summary of disability prevalence trends data for UK studies and studies with UK samples[a,b]

Study	Trend	Time points	Change between first and last time point
Jagger 2016	Odds of any disability at time 2 compared to time 1	T1: 1991<br>T2: 2011	1.22 (1.14-1.30)
	Odds of moderate-severe disability at time 2 compared to time 1		0.76 (0.70-0.82)
	Odds of fair or poor self-rated health at time 2 compared to time 1		0.83 (0.78–0.88)
Kingston 2017	% increase in projected number of people with high dependency	T1: 2015<br>T2: 2025<br>T3: 2035	61.5
	% increase in projected number of people with medium dependency		66.6
	% increase in projected number of care home places needed for those with high dependency		84.5
	% increase in projected number of care home places needed for those with medium dependency		89.3
Kingston 2018	Relative change in projected proportion (%) of sample with high dependency (65-74 years)	T1: 2015<br>T2: 2025<br>T3: 2035	−15.0 (−20.0 to −13.0)
	Relative change in projected proportion (%) of sample with high dependency (75 - 84 years)		42.0 (36.6- 42.7)
	Relative change in projected proportion (%) of sample with high dependency (85+ years)		91.8 (87.3- 94.1)
	Relative change in projected proportion (%) of sample with high dependency (65+)		36.0 (32.6- 36.0)
Guzman-Castillo 2017	Relative change in projected number of disability cases 2015-2025, 65+	T1: 2015<br>T2: 2025	25.0 (21.3–28.2)
	Relative change in projected number of disability cases 2015-2025, 65-84		18.9 (16.6–20.9)
	Relative change in projected number of disability cases 2015-2025, 85		43.2 (34.2–52.1)

[a]Estimates for each time point were not extracted here due to volume. Reader is referred to publications and associated supplementary materials; [b]95% confidence intervals in brackets where reported;

For peer review only

For peer review only - http://bmjopen.bmj.com/site/about/guidelines.xhtml

Table 4e. Summary of inequalities in life expectancy trends, reported as the slope index of inequality between those living in the most and least deprived decile of area deprivation

Study	Trend	Time points	Change between first and last time point (range)
ONS 2019	Change in SSI in life expectancy at birth, England	T1: 2012/14<br>T2: 2015/17	Men: 0.3 (0.2)<br>Women: 0.5 (0.5)
	Change in SSI in life expectancy at age 65, England		Men: 0.3 (0.2)<br>Women: 0.5 (0.5)
	Change in SSI in life expectancy at birth, Wales		Men: 0.5 (0.3)<br>Women: 0.6 (0.7)
	Change in SSI in life expectancy at age 65, Wales		Men: 0.3 (0.2)<br>Women: 0.1 (0.3)

For peer review only - http://bmjopen.bmj.com/site/about/guidelines.xhtml

Table 4f. Summary of inequalities in healthy life expectancy trends, reported as the slope index of inequality between those living in the most and least deprived decile of area deprivation

Study	Trend	Time points	Change between first and last time point (range)
ONS 2019	Change in SSI in healthy life expectancy at birth, England	T1: 2012/14<br>T2: 2015/17	Men: 0.2 (0.2)<br>Women: -0.9 (-0.8)
	Change in SSI in healthy life expectancy at age 65, England		Men: 0.4 (0.9)<br>Women: -0.3 (-0.6)
	Change in SSI in healthy life expectancy at birth, Wales		Men: 0.2 (0.9)<br>Women: -1.1 (-0.9)
	Change in SSI in healthy life expectancy at age 65, Wales		Men: -0.6 (0.8)<br>Women: -1.0 (0.2)

For peer review only

For peer review only - http://bmjopen.bmj.com/site/about/guidelines.xhtml

For peer review only

For peer review only - http://bmjopen.bmj.com/site/about/guidelines.xhtml

Table 5a. Summary of life expectancy trends data for studies from Europe (years)[a]

Study	At age	Time points	T1		T2		T3		T4		T5		T6		T7		T8		Change between first and last time point
			M	F	M	F	M	F	M	F	M	F	M	F	M	F	M	F	
Bronnum-Hansen 2017	65	T1: 2006-2007<br>T2: 2010-2011<br>T3: 2013-2014	High: 17.9<br>Med: 16.3<br>Low: 15.6	High: 20.4<br>Med: 19.4<br>Low: 18.5	Med: 16.8<br>Low: 16.2	High: 21.0<br>Med: 20.0<br>Low: 18.9	High: 19.3<br>Med: 17.5<br>Low: 16.9	High: 21.7<br>Med: 20.6<br>Low: 19.5											Difference in change in LE between high and low education over time:<br>Men: 0.1<br>Women: 0.3
Deeg 2018	65	T1: 1993<br>T2: 1996<br>T3: 1999<br>T4: 2002<br>T5: 2006<br>T6: 2009<br>T7: 2012<br>T8: 2016	14.7	19.2	15.1	19.4	15.5	19.5	16	19.7	17.1	20.5	17.8	21.1	18.2	21.2	18.7	21.4	Men: 4.0<br>Women: 2.2
Gheorghe 2016[b]	25	T1: 2001<br>T2: 2011	High: 53.83<br>Med: 50.70<br>Low: 47.85	High: 58.75<br>Med: 56.82<br>Low: 53.57	High: 56.71<br>Med: 53.92<br>Low: 50.28	High: 60.53<br>Med: 58.35<br>Low: 54.72													Men:<br>High: 2.88<br>Med: 3.22<br>Low: 2.43<br><br>Women:<br>High: 1.78<br>Med: 1.53<br>Low: 1.15
	65		High: 16.81<br>Med: 15.29<br>Low: 14.08	High: 21.00<br>Med: 20.08<br>Low: 18.26	High: 19.29<br>Med: 17.53<br>Low: 15.76	High: 22.64<br>Med: 21.49<br>Low: 19.38													Men:<br>High: 2.48<br>Med: 2.24<br>Low: 1.68<br><br>Women:<br>High: 1.64<br>Med: 1.41<br>Low: 1.12
Remund 2019	30	T1: 1990/94<br>T2: 1995/99<br>T3: 2000/04<br>T4: 2005/09<br>T5: 2010/14	**data only reported in graphs, unable to extract**																Men: 5.02<br>Women: 3.09

For peer review only

For peer review only - http://bmjopen.bmj.com/site/about/guidelines.xhtml

Study	At age	Time points	T1		T2		T3		T4		T5		T6		T7		T8		Change between first and last time point
			M	F	M	F	M	F	M	F	M	F	M	F	M	F	M	F	
Renard 2019[b]	25	T1: 2001<br>T2: 2011	High: 55.82<br>Med: 53.18<br>Low: 50.63	High: 60.90<br>Med: 59.14<br>Low: 57.14	High: 57.81<br>Med: 54.73<br>Low: 51.74	High: 61.90<br>Med: 60.09<br>Low: 57.32													Men:<br>Low: 1.11<br>Med: 1.55<br>High: 1.99<br><br>WOMEN:<br>Low: 0.18<br>Med: 0.95<br>High: 1.00
Reus-Pons 2017	50	T1: 2001<br>T2: 2011	28.05	32.47	30.87	34.31													Men: 2.82 [d]<br>Women: 1.84 [d]
Storeng 2018	30	T1: 1984/86<br>T2: 1995/97<br>T3: 2006/08	45.1 (44.6–45.5)	50.4 (49.9–50.8)	48.3 (47.8–48.7)	52.9 (52.5–53.4)	52.1 (51.5–52.6)	57.1 (56.6–57.6)											Men: 6.99 (5.27–8.72)<br>Women: 6.75 (5.16–8.34)
			Primary: 47.0 (46.5–47.5)<br>Secondary: 47.9 (47.4–48.5)<br>Tertiary: 51.2 (49.6–52.7)		Primary: 49.7 (49.1–50.2)<br>Secondary: 50.9 (50.4–51.4)<br>Tertiary: 52.0 (50.9–53.1)		Primary: 52.2 (51.0–53.4)<br>Secondary: 54.9 (54.3–55.4)<br>Tertiary: 55.2 (53.1–57.4)												Primary: 5.19 (3.52–6.85)<br>Secondary: 6.95 (5.87–8.02)<br>Tertiary: 3.23 (–0.04–6.51)
Sundberg 2016	77	SWEOLD<br>T1: 1992<br>T2: 2002<br>T3: 2004<br>T4: 2011	8.2	10.7	8.9	10.8	9.3	11.4	9.9	11.8									Men: 1.7 [d]<br>Women: 1.1 [d]
		SHARE:<br>T1: 2004<br>T2: 2011	9.3	11.4	9.9	11.8													Men: 0.6 [d]<br>Women: 0.4 [d]
Yokota 2016	15	T1: 2001<br>T2: 2004<br>T3: 2008	60.5	66.4	61.4	67	62.1	67.4											Men: 1.6 [d]<br>Women: 1.0[d]

[a]95% confidence intervals in brackets where reported; [b] Trends reported by high, medium and low education; [c]Trends not reported by sex; [d]Change estimate not reported, and thus calculated by us (T^2-T^1)

For peer review only - http://bmjopen.bmj.com/site/about/guidelines.xhtml

Table 5b. Summary of healthy life expectancy trends data for studies from Europe[a]

Study	Trend	At age	Time points	T1		T2		T3		T4		T5		T6		T7		T8		Change between first and last time point
				M	F	M	F	M	F	M	F	M	F	M	F	M	F	M	F	
Deeg 2018	% life in good physical health	65	T1: 1993<br>T2: 1996<br>T3: 1999<br>T4: 2002<br>T5: 2006<br>T6: 2009<br>T7: 2012<br>T8: 2016	46.6	28.7	37.6	26.0	33.6	24.5	32.4	22.7	32.7	21.2	30.5	20.2	22.8	14.9	25.0	18.9	Men: -21.6<br>Women: -9.8
	HLE: Physical (years)			6.9	5.5													4.7	4.0	Men: -2.2[c]<br>Women: -1.5[c]
Gheorghe 2016[b]	Quality Adjusted Life Expectancy	25	T1: 2001<br>T2: 2011	High: 44.08<br>Med: 40.82<br>Low: 36.71	High: 45.79<br>Med: 44.2<br>Low: 39.51	High:46.93<br>Med: 43.83<br>Low: 38.84	High: 48.43<br>Med: 45.77<br>Low: 41.31													MEN:<br>High 2.85<br>Med: 3.01<br>Low: 2.13<br><br>WOMEN:<br>High: 2.64<br>Med: 1.57<br>Low: 1.80
		65		High: 13.42<br>Med: 12.06<br>Low: 10.73	High: 15.66<br>Med: 15.12<br>Low: 13.09	High: 15.59<br>Med: 13.97<br>Low: 12.10	High: 17.40<br>Med: 16.33<br>Low: 14.23													MEN:<br>High: 2.17<br>Med: 1.91<br>Low: 1.37<br><br>WOMEN:<br>High: 1.74<br>Med: 1.21<br>Low: 1.14
Remund 2019	HLE (years)	30	T1: 1990/94<br>T2: 1995/99<br>T3: 2000/04<br>T4: 2005/09	**data only reported in graphs, unable to extract**																Men: 4.52<br>Women: 3.09

For peer review only - http://bmjopen.bmj.com/site/about/guidelines.xhtml

Study	Trend	At age	Time points	T1		T2		T3		T4		T5		T6		T7		T8		Change between first and last time point
				M	F	M	F	M	F	M	F	M	F	M	F	M	F	M	F	
	HLE (years), educational gap between primary and tertiary attainment		T5: 2010/14	7.6	3.3	**not reported**						8.8	5.0							Change in educational gap (between primary and tertiary): Men: 1.2[c] Women: 1.7[c]
Reus-Pons 2017	HLE (years)	50	T1: 2001 T2: 2011	18.62 (18.40 - 18.84)	19.43 (19.19 - 19.68)	20.83 (20.55 - 21.10)	20.68 (20.37 - 20.99)													Men: 2.21 Women: 1.25
Storeng 2018[d]	HLE (years)	30	T1: 1984/86 T2: 1995/97 T3: 2006/08	31.6 (31.2–31.9)	32.6 (32.3–33.0)	34.1 (33.7–34.5)	33.9 (33.6–34.3)	38.5 (38.0–39.0)	38.0 (37.5–38.5)											Men: 6.90 (6.08–7.73) Women: 5.40 (4.56–6.25)
				Primary:29.5 (29.2–29.9) Secondary:34.0 (33.6–34.5) Tertiary: 40.1 (38.8–41.4)		Primary:29.8 (29.4–30.3) Secondary: 35.7 (35.3–36.2) Tertiary: 40.3 (39.3–41.3)		Primary: 31.5 (30.6–32.4) Secondary:38.8 (38.3–39.4) Tertiary: 42.8 (41.9–43.8)												Primary: 1.94 (0.68–3.20) Secondary: 4.80 (3.85–5.75) Tertiary: 2.76 (0.49–5.02) Change in gap between primary and tertiary education: 0.7[c]

[a]95% confidence intervals in brackets where reported; [b]Trends reported by high, medium and low education; [c]Change estimate not reported, and thus calculated by us (T^{2}-T^{1}); [d]Trends reported by primary, secondary and tertiary education;

For peer review only - http://bmjopen.bmj.com/site/about/guidelines.xhtml

Table 5c. Summary of disability-free life expectancy trends data for studies from Europe (years)[a]

Study	Trend	At age	Time points	T1		T2		T3		T4		Change between first and last time point
				Male	Female	Male	Female	Male	Female	Male	Female	
Bronnum-Hansen 2017[b]	DFLE	65	T1: 2006-2007<br>T2: 2010-2011<br>T3: 2013-2014	High: 10.6 (9.2–12.1)<br>Med: 9.4 (8.3–10.5)<br>Low: 7.4 (5.9–8.9)	High: 12.5 (10.5–14.5)<br>Med: 9.5 (8.1–10.9)<br>Low: 8.8 (7.7–9.9)	High: 10.6 (9.2–12.0)<br>Med: 9.3 (8.2–10.4)<br>Low: 7.0 (5.3–8.8)	High: 13.2 (10.9–15.6)<br>Med: 10.4 (8.9–11.8)<br>Low: 7.9 (6.6–9.3)	High: 10.9 (9.9–12.0)<br>Med: 9.6 (8.8–10.5)<br>Low: 8.0 (6.7–9.3)	High: 12.9 (11.2–14.6)<br>Med: 10.2 (9.0–11.4)<br>Low: 9.5 (8.5–10.5)			Difference in change in DFLE between those with the lowest and highest education<br>Men: -0.3<br>Women: -0.3
Lagergren 2017	Added years of life without ADL limitations	65+	T1: 1980/85<br>T2: 1994/99<br>T3: 2006/11	**data not reported**								Men: 4.0<br>Women: 3.14
	Added years of life without mobility limitations			**data not reported**								Men: 4.21<br>Women: 3.69
Renard 2019	DFLE	25	T1: 2001<br>T2: 2011	Low: 36.34<br>Med: 41.40<br>High: 42.85	Low: 39.32<br>Med: 43.37<br>High: 48.62	Low:37.02<br>Med: 42.05<br>High: 47.49	Low:35.54<br>Med:42.98<br>High: 48.98					Difference in change in DFLE between levels of education (ref high education) change over time<br>Men:<br>Low: 3.96<br>Med: 3.99

For peer review only - http://bmjopen.bmj.com/site/about/guidelines.xhtml

Study	Trend	At age	Time points	T1		T2		T3		T4		Change between first and last time point
				Male	Female	Male	Female	Male	Female	Male	Female	
												Women: Low: 4.14 Med: 0.76
Storeng 2018[e]	DFLE	30	T1: 1984/86 T2: 1995/97 T3: 2006/08	25.9 (25.6–26.2)	28.8 (28.6–29.1)	28.5 (28.1–28.8)	29.4 (29.0–29.7)	28.6 (28.2–29.0)	29.2 (28.8–29.6)			Men: 2.71 (2.01–3.42) Women: 0.33 (–0.40–1.06)
				Primary: 25.0 (24.7–25.3) Secondary: 28.8 (28.5–29.2) Tertiary: 33.6 (32.5–34.7)		Primary: 25.3 (24.9–25.7) Secondary: 30.1 (29.7–30.5) Tertiary: 33.0 (32.3–33.8)		Primary: 22.5 (21.7–23.2) Secondary: 29.1 (28.6–29.5) Tertiary: 33.2 (32.4–34.1)				Primary: –2.53 (–3.61–1.45) Secondary: 0.23 (–0.57–1.03) Tertiary: –0.32 (–2.23–1.60)
Sundberg 2016	DFLE	77	SWEOLD T1: 1992 T2: 2002 T3: 2004 T4: 2011	6.7 (6.3–7.1)	6.9 (6.4–7.4)	6.9 (6.4–7.3	7.0 (6.5–7.5)	7.4 (7.0–7.9)	7.9 (7.4–8.4)	7.8 (7.2–8.3)	8.5 (8.0–9.0	Men: 1.1 Women: 1.6
			SHARE: T1: 2004 T2: 2011	7.5 (7.0–8.0	7.9 (7.3–8.6	7.6 (7.0–8.1)	9.2 (8.5–9.8)					Men: 0.1 Women: 1.3
Yokota 2019	DFLE	15	T1: 2001 T2: 2004 T3: 2008	54.1	56.0	54.9	56.2	54.8	55.3			Men: 0.7 Women: -0.7

[a]95% confidence intervals in brackets where reported; [b]Stratified by high, medium and low education; [c]Not stratified by sex; [d]Change estimate not reported, and thus calculated by us (T^{2}-T^{1}) [e]Second row stratified by primary, secondary and tertiary education for men and women combined

For peer review only

Table 5d. Summary of disability prevalence trends data for studies from Europe

Study	Trend	Time points	Change between first and last time point[a]
Lagergren 2017	Proportion reporting dependency in activities of daily living, 65-69	T1:1980/85<br>T2: 1994/95<br>T3: 2006/11	Men:-1.5<br>Women:-3.7
	Proportion reporting dependency in activities of daily living, 70-74		Men:-5.6<br>Women:-3.5
	Proportion reporting dependency in activities of daily living, 75-79		Men:-10.7<br>Women:-7.1
	Proportion reporting dependency in activities of daily living, 80-84		Men: -11.5<br>Women: -14.0
	Proportion reporting dependency in activities of daily living, 85+		Men:-20.3<br>Women: -10.5
	Proportion reporting mobility limitations, 65-69		Men:-6.5<br>Women: -8.3
	Proportion reporting mobility limitations, 70-74		Men: -10.9<br>Women: -8.5
	Proportion reporting mobility limitations, 75-79		Men: -15.7<br>Women: -14.8
	Proportion reporting mobility limitations, 80-84		Men: -15.1<br>Women: -19.4
	Proportion reporting mobility limitations, 85+		Men: -16.1<br>Women: -17.5

[a]Change estimate not reported, and thus calculated by us (T^2-T^1)

For peer review only - http://bmjopen.bmj.com/site/about/guidelines.xhtml

Table 6a. Summary of life expectancy trends data for studies from Japan and Korea (years)[a]

Study	At age	Time points	T1		T2		T3		T4		T5		T6		T7		T8		Change between first and last time point
			M	F	M	F	M	F	M	F	M	F	M	F	M	F	M	F	
Jo 2019	0	T1: 2005<br>T2: 2007<br>T3: 2008<br>T4: 2009<br>T5: 2010<br>T6: 2011<br>T7: 2012	75.13	81.87	76.13	82.71	76.54	83.27	76.99	83.74	77.19	84.04	77.64	84.42	77.94	84.61	78.51	85.02	Men: 3.38[b]<br>Women: 3.15[b]
Lee 2016	0	T1: 2005<br>T2: 2008<br>T3: 2011	75.1	81.8	76.5	83.2	77.6	84.4											Men: 2.5[b]<br>Women: 2.6[b]
	65		15.8	19.8	16.6	21.0	17.4	21.8											Men: 1.6[b]<br>Women: 2.0[b]
	85		5.1	6.2	5.2	6.5	5.4	6.9											Men: 0.3[b]<br>Women: 0.7[b]
Sugawara 2016	0	T1: 2000<br>T2: 2010	77.6	84.7	79.5	86.4													Men: 1.9<br>Women:1.7
Tokudome 2016	0	T1: 1990<br>T2: 1995<br>T3: 2000<br>T4: 2005<br>T5: 2010<br>T6: 2013	76.04 (75.98–76.10)	81.96 (81.86–82.05)	76.45 (76.14–76.57)	82.84 (82.62–82.94)	77.55 (77.53–77.58)	84.32 (84.29–84.35)	78.66 (78.60–78.71)	85.48 (85.41–85.54)	79.34 (79.31–79.36)	85.09 (86.06–86.11)	80.05 (79.26–80.84)	86.39 (85.74–87.12)					Men: 4.01[b]<br>Women: 4.43[b]

[a]95% confidence intervals in brackets where reported; [b]Change estimate not reported, and thus calculated by us (T^2-T^1)

For peer review only

For peer review only - http://bmjopen.bmj.com/site/about/guidelines.xhtml

Table 6b. Summary of healthy life expectancy trends data for studies from Japan and Korea (years)[a]

Study	At age	Time points	T1		T2		T3		T4		T5		T6		T7		T8		Change between first and last time point
			M	F	M	F	M	F	M	F	M	F	M	F	M	F	M	F	
Jo 2019	0	T1: 2005<br>T2: 2007<br>T3: 2008<br>T4: 2009<br>T5: 2010<br>T6: 2011<br>T7: 2012	70.8	74.7	71.86	73.92	72.62	75.18	73.4	76.65	73.87	77.21	74.33	76.53	74.71	78.3	74.83	78.14	Men: 4.03<br>Women: 3.44
Lee 2016	0	T1: 2005<br>T2: 2008<br>T3: 2011	64.4	67.7	64.0	67.3	65.8	68.9											Men: 1.4[b]<br>Women: 1.2[b]
	65		9.5	11.5	10.4	12.8	11.7	14.1											Men: 2.2[b]<br>Women: 2.6[b]
	85		2.6	3.8	3.3	4.7	4.0	5.2											Men: 1.4[b]<br>Women: 1.4[b]
Tokudome 2016	0	T1: 1990<br>T2: 1995<br>T3: 2000<br>T4: 2005<br>T5: 2010<br>T6: 2013	68.09 (65.83–70.11)	72.24 (69.38–74.77)	68.44 (66.07–70.58)	72.92 (70.05–75.49)	69.08 (66.67–71.24)	73.95 (70.94–76.55)	69.89 (67.31–72.12)	74.77 (71.66–77.46)	70.78 (68.20–73.06)	75.41 (72.34–78.23)	71.11 (68.50–73.57))	75.56 (72.46–78.42)					Men: 3.02[b]<br>Women: 3.32[b]

[a]95% confidence intervals in brackets where reported; [b]Change estimate not reported, and thus calculated by us (T^{2}-T^{1})

For peer review only

For peer review only - http://bmjopen.bmj.com/site/about/guidelines.xhtml

Table 6c. Summary of disability-free life expectancy trends data for studies from Japan[a]

Study	Trend	At age	Time points	T1		T2		Change between first and last time point
				Male	Female	Male	Female	
Sugawara 2016	DFLE (years)	0	T1: 2000<br>T2: 2010	68.0	71.7	69.0	72.1	Men: 1.0<br>Women: 0.4
	% of life disability-free			87.6	84.6	86.8	83.4	Men: -0.8<br>Women: -1.2

[a]95% confidence intervals in brackets where reported;

For peer review only

Table 7a. Summary of life expectancy trends data high income countries combined from the GBD studies (years)[a]

Study author and date	At age	Time points	T1		T2		Change between first and last time point
			Male	Female	Male	Female	
GBD 2016	0	T1: 2005<br>T2: 2015	76·35 (76·33–76·37)	82·09 (82·07–82·11)	78·10 (78·05–78·15)	83·42 (83·38–83·46)	Men: 1.75[b]<br>Women: 1.33[b]
GBD 2017	0	T1: 1990<br>T2: 2016	72·64 (72·54–72·75)	79·34 (79·24–79·43)	78·27 (78·04–78·49)	83·48 (83·28–83·67)	Men: 5.63[b]<br>Women: 4.14[b]
	65		14·96 (14·90–15·01)	18·77 (18·71–18·84)	18·47 (18·32–18·60)	21·80 (21·66–21·94)	Men: 3.51[b]<br>Women: 3.03[b]
GBD 2018	0	T1: 1990<br>T2: 2017	72·8 (72·7–72·8)	79·4 (79·4–79·4)	78·4 (78·2–78·6)	83·6 (83·4–83·7)	Men: 5.6[b]<br>Women: 4.2[b]

[a]95% confidence intervals in brackets where reported; [b]Change estimate not reported in publication, and calculated by us (T^2-T^1);

For peer review only

Table 7b. Summary of healthy life expectancy trends data for high income countries combined from the GBD studies[a]

Study	Trend	At age	Time points	T1		T2		Change between first and last time point
				Male	Female	Male	Female	
GBD 2016	HLE (years)	0	T1: 2005 T2: 2015	67.48 (64.90–69.75)	71.13 (67.95–73.87)	68.91 (66.27–71.25)	72.21 (68.97–75.02)	Men: 1.43[b] Women: 1.08[b]
GBD 2017	HLE (years)	0	T1: 1990 T2: 2016	64.15 (61.70–66.33)	68.40 (65.23–71.19)	68.58 (65.82–71.06)	71.61 (68.12–74.66)	Men: 4.43[b] Women: 3.21[b]
		65		11.0 (10.45–12.27)	14.28 (13.06–15.39)	14.00 (12.79–15.11)	16.55 (15.09–17.85)	Men: 2.60[b] Women: 2.27[b]
GBD 2018	HLE (years)	0	T1: 1990 T2: 2017	64.0 (61.4–66.1)	68.0 (64.7–70.8)	68.2 (65.3–70.7)	71.0 (67.5–74.0)	Men: 3.0[b] Women: 4.2[b]

[a]95% confidence intervals in brackets where reported; [b]Change estimate not reported in publication, and calculated by us (T^2-T^1);

For peer review only - http://bmjopen.bmj.com/site/about/guidelines.xhtml

Search strategy as applied to Medline

Database(s): **Ovid MEDLINE(R) and In-Process & Other Non-Indexed Citations** 1946 to September 13, 2019

#	Searches	Results
1	Life Expectancy/	16893
2	Longevity/	20149
3	Needs assessment/	28915
4	((expectancy or Dependen* or disabilit*) adj2 years).ti,ab.	968
5	(expectanc* adj5 disabilit*).ti,ab.	406
6	(Care need* adj2 years).ti,ab.	13
7	(healthy life years or "years of healthy life").ti,ab,kw.	260
8	or/1-7	65417
9	exp Age Factors/ or Race Factors/ or Sex Factors/	631787
10	Time Factors/	1159988
11	exp Forecasting/	82762
12	Life Tables/	6387
13	td.fs.	367819
14	or/9-13	2116951
15	8 and 14	14752
16	Life Expectancy/td [Trends]	2146

For peer review only - http://bmjopen.bmj.com/site/about/guidelines.xhtml

17	Needs Assessment/td [Trends]	249
18	((life expectancy or expected years or care need*) adj2 (trend* or direction* or factor* or forecast*)).ti,ab.	334
19	or/16-18	2649
20	15 or 19	14962
21	limit 20 to (humans and yr="2016 -Current")	1661
22	letter/	1034636
23	Editorial/	498100
24	News/	196781
25	exp Historical Article/	390531
26	Anecdotes as Topic/	4730
27	Comment/	799622
28	(letter or comment$).ti.	138712
29	or/22-28	2365158
30	21 not 29	1502

For peer review only

Supplementary materials Figure 1. Expansion and compression of poor health/disability by country and time period

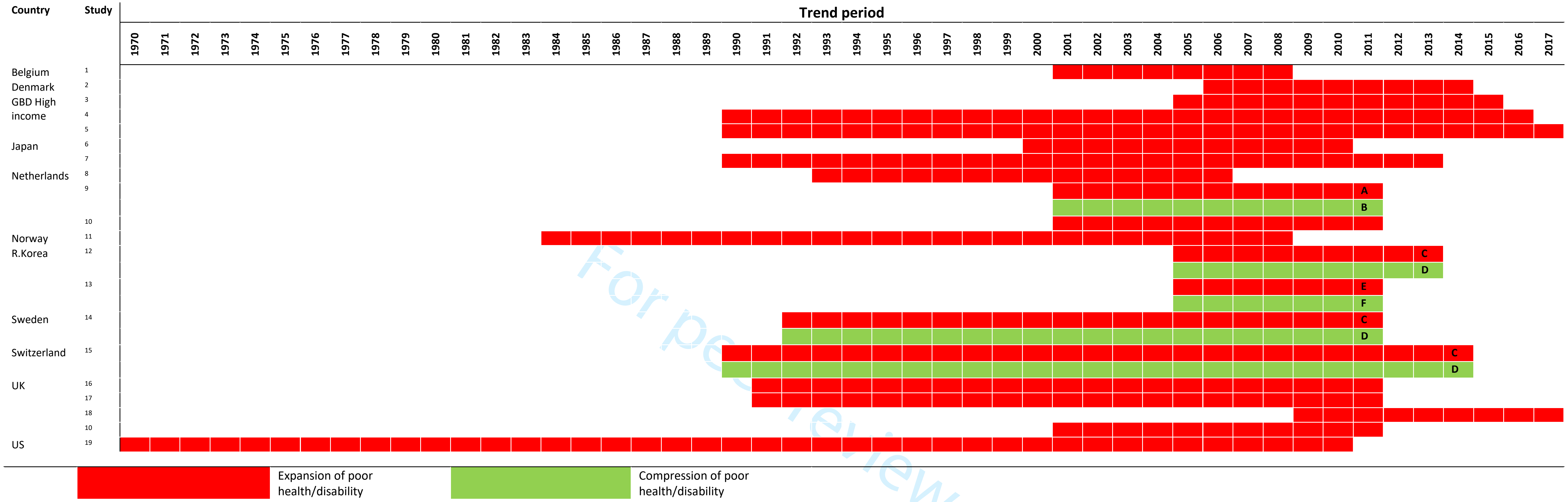

<u>**Where multiple studies in countries or conclusions differ between sub-groups:**</u> [a]Men, 25 and 65, all levels of education; [b]Women (age 25, all levels of education; age 65, high and low education); [c]Men; [d]Women; [e]At birth; [f]Age 65 and 86

For peer review only

PRISMA 2009 Checklist

Section/topic	#	Checklist item	Reported on page #
**TITLE**			
Title	1	Identify the report as a systematic review, meta-analysis, or both.	1
**ABSTRACT**			
Structured summary	2	Provide a structured summary including, as applicable: background; objectives; data sources; study eligibility criteria, participants, and interventions; study appraisal and synthesis methods; results; limitations; conclusions and implications of key findings; systematic review registration number.	2
**INTRODUCTION**			
Rationale	3	Describe the rationale for the review in the context of what is already known.	3-4
Objectives	4	Provide an explicit statement of questions being addressed with reference to participants, interventions, comparisons, outcomes, and study design (PICOS).	4
**METHODS**			
Protocol and registration	5	Indicate if a review protocol exists, if and where it can be accessed (e.g., Web address), and, if available, provide registration information including registration number.	NA
Eligibility criteria	6	Specify study characteristics (e.g., PICOS, length of follow-up) and report characteristics (e.g., years considered, language, publication status) used as criteria for eligibility, giving rationale.	4-5
Information sources	7	Describe all information sources (e.g., databases with dates of coverage, contact with study authors to identify additional studies) in the search and date last searched.	4
Search	8	Present full electronic search strategy for at least one database, including any limits used, such that it could be repeated.	4
Study selection	9	State the process for selecting studies (i.e., screening, eligibility, included in systematic review, and, if applicable, included in the meta-analysis).	5-6
Data collection process	10	Describe method of data extraction from reports (e.g., piloted forms, independently, in duplicate) and any processes for obtaining and confirming data from investigators.	7
Data items	11	List and define all variables for which data were sought (e.g., PICOS, funding sources) and any assumptions and simplifications made.	4-5
Risk of bias in individual studies	12	Describe methods used for assessing risk of bias of individual studies (including specification of whether this was done at the study or outcome level), and how this information is to be used in any data synthesis.	6-7
Summary measures	13	State the principal summary measures (e.g., risk ratio, difference in means).	7-8
Synthesis of results	14	Describe the methods of handling data and combining results of studies, if done, including measures of consistency (e.g., I^2) for each meta-analysis.	7-8

For peer review only - http://bmjopen.bmj.com/site/about/guidelines.xhtml

PRISMA 2009 Checklist

Section/topic	#	Checklist item	Reported on page #
Risk of bias across studies	15	Specify any assessment of risk of bias that may affect the cumulative evidence (e.g., publication bias, selective reporting within studies).	NA
Additional analyses	16	Describe methods of additional analyses (e.g., sensitivity or subgroup analyses, meta-regression), if done, indicating which were pre-specified.	NA
**RESULTS**			
Study selection	17	Give numbers of studies screened, assessed for eligibility, and included in the review, with reasons for exclusions at each stage, ideally with a flow diagram.	8, and figure 1
Study characteristics	18	For each study, present characteristics for which data were extracted (e.g., study size, PICOS, follow-up period) and provide the citations.	Supplement materials table 1
Risk of bias within studies	19	Present data on risk of bias of each study and, if available, any outcome level assessment (see item 12).	Supplementary materials table 2
Results of individual studies	20	For all outcomes considered (benefits or harms), present, for each study: (a) simple summary data for each intervention group (b) effect estimates and confidence intervals, ideally with a forest plot.	Table 3, Supplementary materials tables 3-7
Synthesis of results	21	Present results of each meta-analysis done, including confidence intervals and measures of consistency.	NA – no meta analysis
Risk of bias across studies	22	Present results of any assessment of risk of bias across studies (see Item 15).	Supplementary materials table 2
Additional analysis	23	Give results of additional analyses, if done (e.g., sensitivity or subgroup analyses, meta-regression [see Item 16]).	NA
**DISCUSSION**			
Summary of evidence	24	Summarize the main findings including the strength of evidence for each main outcome; consider their relevance to key groups (e.g., healthcare providers, users, and policy makers).	12-15
Limitations	25	Discuss limitations at study and outcome level (e.g., risk of bias), and at review-level (e.g., incomplete retrieval of identified research, reporting bias).	13-14
Conclusions	26	Provide a general interpretation of the results in the context of other evidence, and implications for future	12-15

For peer review only

For peer review only - http://bmjopen.bmj.com/site/about/guidelines.xhtml

PRISMA 2009 Checklist

		research.	
**FUNDING**			
Funding	27	Describe sources of funding for the systematic review and other support (e.g., supply of data); role of funders for the systematic review.	Title page

From: Moher D, Liberati A, Tetzlaff J, Altman DG, The PRISMA Group (2009). Preferred Reporting Items for Systematic Reviews and Meta-Analyses: The PRISMA Statement. PLoS Med 6(7): e1000097. doi:10.1371/journal.pmed1000097

For more information, visit: www.prisma-statement.org.

For peer review only

For peer review only - http://bmjopen.bmj.com/site/about/guidelines.xhtml
